# Trends in the Incidence and Mortality of Tobacco-Related Cancers Among Adults in the United States

**DOI:** 10.3390/cancers17030534

**Published:** 2025-02-05

**Authors:** Nandika Mansingka, Victor Adekanmbi, Christine D. Hsu, Thao N. Hoang, Jacques G. Baillargeon, Abbey B. Berenson, Fangjian Guo

**Affiliations:** 1 School of Public and Population Health, The University of Texas Medical Branch at Galveston, Galveston, TX 77555, USA; namansin@utmb.edu; 2 Department of Obstetrics & Gynecology, The University of Texas Medical Branch at Galveston, Galveston, TX 77555, USA; viadekan@utmb.edu (V.A.); cdhsu@utmb.edu (C.D.H.); thnhoang@utmb.edu (T.N.H.); abberens@utmb.edu (A.B.B.); 3 Center for Interdisciplinary Research in Women’s Health, The University of Texas Medical Branch at Galveston, Galveston, TX 77555, USA; 4 Department of Epidemiology, School of Public and Population Health, The University of Texas Medical Branch at Galveston, Galveston, TX 77555, USA; jbaillar@utmb.edu

**Keywords:** tobacco, cancer, incidence, mortality, trend

## Abstract

Background: Tobacco use remains a global challenge to public health, accounting for almost eight million deaths per year worldwide, with a significant portion attributable to tobacco-related cancers. Examining the epidemiology of tobacco-related cancers and assessing the trends in the incidence and mortality will allow for more effective prevention, treatment, and targeted strategies. Methods: We assessed the trends in the incidence and mortality of tobacco-related cancers among adults in the United States using data from United States Cancer Statistics (USCS) 2001–2021 and mortality data 1975–2022 from the National Center for Health Statistics (NCHS). The incidence and mortality rates of tobacco-related cancers were calculated as cases per 1,000,000 persons and age-adjusted to the 2000 United States standard population. Results: There was a recent overall decreasing trend in both the incidence (2001–2021) and mortality rate (2001–2022). Among adults 20–49 years old, there was an increasing trend from 2001 to 2021 in the incidence among non-Hispanic American Indians/Alaska Natives (APC 2.6, 95% CI 2.1–3.0) and those in the West (APC 0.2, 95% CI 0.0–0.4); in Hispanics, the incidence rate increased most recently from 2013 to 2021 (APC 1.7, 95% CI 1.0–3.0). The mortality rate first increased from 1975 to 1990 among females 50–64 years old and males 65+ years old and from 1975 to 2000 among females 65+ years old, and then decreased thereafter. Conclusions: The rising incidence in some younger groups highlights the need for targeted public health interventions to address disparities and improve cancer prevention in these vulnerable populations.

## Simple Summary

The aim of this study was to assess the trends in the incidence and mortality of tobacco-related cancers among adults in the United States using data from United States Cancer Statistics (USCS) 2001–2021 and mortality data 1975–2022 from the National Center for Health Statistics (NCHS). There was a recent overall decreasing trend in both the incidence (2001–2021) and mortality rate (2001–2022). Among adults 20–49 years old, there was an increasing trend from 2001 to 2021 in the incidence among non-Hispanic American Indians/Alaska Natives and those in the West, and most recently from 2013 to 2021 in Hispanics. The mortality rate first increased from 1975 to 1990 among females 50–64 years old and males 65+ years old and from 1975 to 2000 among females 65+ years old, and then decreased thereafter. The rising incidence in some younger groups highlights the need for targeted public health interventions to address these disparities.

## 1. Introduction

Tobacco use remains a global challenge to public health, accounting for almost eight million deaths per year, with a significant portion attributable to tobacco-related cancers [1,2,3,4]. Approximately one-fourth of all deaths caused by tobacco use worldwide are cancer related due to the involvement of more than 60 well-known carcinogens. Smoking and chewing are the most common ways to consume tobacco and can lead to more than 10 types of cancers, including lung, larynx, mouth, esophagus, and bladder cancers [1,2,3,4]. While the link between tobacco use and cancer is well-established, this relationship is continuously evolving through changes in common smoking practices, the implementation of new medical treatments, and the enactment of public health initiatives to decrease tobacco use.

It is estimated that 1.2 billion people worldwide use tobacco products regularly in 2020, with the number of male smokers being five times of female smokers [5]. While global tobacco consumption has declined in some regions due to public health initiatives, low- and middle-income countries made little progress as tobacco control measures in these countries are less stringent [5]. In the United States, tobacco use has significantly decreased over the past few decades due to widespread public health campaigns, smoking bans, and increased awareness of its health risks [6,7,8,9]. However, tobacco-related cancers continue to impact millions of people, and there are disparities across age, gender, and racial/ethnic groups [1,2]. Identifying the trends in the incidence and mortality of tobacco-related cancers can provide insights into key patterns and disparities that could inform future interventions to reduce the burden of these diseases. Examining the epidemiology of tobacco-related cancers and assessing these trends will also allow for more effective prevention, treatment, and targeted strategies. There is a lack of comprehensive studies analyzing these trends in the United States [1,2]. In this study, we closely examined the trends in the incidence and mortality of tobacco-related cancers among adults in the United States by age group, sex, race/ethnicity, and region of residence. We also examined patterns in smoking rates and insurance coverage among adults in the United States.

## 2. Materials and Methods

Data used for incidence of tobacco-related cancers are from United States Cancer Statistics (USCS) [10]. The USCS 2001–2021 database is a broad registry of data from the Centers for Disease Control and Prevention (CDC)’s National Program for Cancer Registries (NPCR) and the National Cancer Institute’s (NCI’s) Surveillance, Epidemiology, and End Results (SEER) Program. Hospitals, physicians, and laboratories nationwide report data on demographic characteristics and tumor characteristics to central cancer registries supported by the CDC and NCI. Cancer incidence and population data for all 50 states and the District of Columbia are included in USCS data. The NPCR and SEER Incidence–USCS Public Use Database (2001–2021 database) essentially covered the entire United States adult population between 2001 and 2021. The underlying mortality data related to tobacco-related cancers are from the National Center for Health Statistics (NCHS) [11,12,13,14]. We included mortality data from 1975 to 2022. This study was exempt from full board review by the Institutional Review Board at The University of Texas Medical Branch at Galveston, (Galveston, TX, USA) as data used in this study were deidentified. This report follows the STROBE reporting guideline [15].

Each case of tobacco-related cancers includes demographic characteristics and date of cancer diagnosis. Tobacco-related cancers [16] included primary cancers in the following sites: lip, oral cavity, and pharynx (International Classification of Diseases for Oncology, Third Edition [ICD-O-3] site codes C00.0–14.8); esophagus (ICD-O-3 site codes C15.0–15.9); stomach (ICD-O-3 site codes C16.0–16.9); colon and rectum (ICD-O-3 site codes C18.0–20.9, C26.0); liver (ICD-O-3 site codes C22.0); pancreas (ICD-O-3 site codes C25.0–25.9); larynx (ICD-O-3 site codes C32.0–32.9); trachea, lung, and bronchus (ICD-O-3 site codes C33.9–34.9); cervix uteri (ICD-O-3 site codes C53.0–53.9); kidney and renal pelvis (ICD-O-3 site codes C64.9–65.9); urinary bladder (ICD-O-3 site codes C67.0–67.9); and acute myeloid leukemia. We also separately analyzed the following group of tobacco-related cancers: lung, oral cavity, pharynx, larynx, bladder, and esophagus. We excluded cases that were identified by autopsy or death certificate only. Only cases defined as malignant under International Classification of Diseases for Oncology, Third Edition (ICD-O-3) were included in this study. We included the following ICD-O-3 histology codes for these cancer sites: 8000–9049, 9056–9139, and 9141–9589. For acute myeloid leukemia specifically, ICD-O-3 histology codes 9840, 9861, 9865–9869, 9871–9874, 9895–9898, 9910–9911, and 9920 were included. Stages at diagnosis included localized, regional, and distant (metastasized).

Data were grouped into the following age categories: 20–49 years old, 50–64 years old, and 65+ years old. We included four regions of residence: Northeast, Midwest, South, and West. The analyses included information about ethnicity (Hispanic or non-Hispanic) and race (non-Hispanic White, non-Hispanic Black, non-Hispanic American Indian/Alaska Native, and non-Hispanic Asian or Pacific Islander). Hispanic ethnicity for all cancer cases was identified by the North American Association of Central Cancer Registries (NAACCR) Hispanic/Latino Identification Algorithm (NHIA) [17]. We included in the analyses information about ethnicity (Hispanic or non-Hispanic) and race (non-Hispanic White, non-Hispanic Black, non-Hispanic American Indian/Alaska Native, and non-Hispanic Asian or Pacific Islander).

We analyzed data from the National Health Interview Survey (NHIS) to assess the prevalence of tobacco smoking and health insurance coverage. Through in-person household surveys, the NHIS collected health information annually. However, this shifted to mainly phone interviews from March to December 2020 due to the COVID-19 pandemic. The survey sampled a representative cross-sectional, non-institutionalized population to assess the health status and behaviors of U.S. adults, employing a complex, stratified, multistage sample design to ensure nationally representative data. Verbal consent for participation was obtained from each participant.

We used data from NHIS 1983–2021 to assess prevalence of tobacco-smoking among adults 18+ years old. Current cigarette smoking was defined as having smoked at least 100 cigarettes in a lifetime and currently smoking every day or on some days. Current electronic cigarette use was defined as using electronic cigarettes or other vaping products daily or on some days, with data available from 2016 to 2021.

To assess health insurance coverage, we analyzed NHIS data from 2000 to 2021 for adults aged 18 years and older. Being insured was defined as having private insurance (including comprehensive plans obtained through employers, through local/community programs, purchased directly, or via the Health Insurance Marketplace or state-based exchanges) and/or public insurance (such as Medicaid, CHIP, Medicare, military plans, or other government-sponsored health plans).

## 3. Statistical Analysis

All analyses were carried out using SAS software version 9.4 (SAS Institute; Cary, NC, USA), the SEER*Stat statistical software package (version 8.4.3), and Joinpoint Regression Analysis program, version 5.3.0.0 [18]. Statistical significances were determined as 2–sided *p* values < 0.05. The incidence and mortality rates of tobacco-related cancers were calculated as cases or deaths per 1,000,000 persons and age-adjusted to the 2000 U.S. standard population. Confidence intervals were calculated using the Tiwari method [19]. Joinpoint regression models [20] were fitted based on annual incidence data of 2001–2021 using the National Cancer Institute’s Joinpoint Regression Analysis program, version 5.2.0 [18]. This analysis program selected the best-fitting log-linear regression model to identify the joinpoints (calendar years) when annual percentage changes (APCs) changed significantly, allowing for the minimum number of joinpoints necessary to fit the data. APC was calculated as (exp[β] − 1) ∗ 100, where the regression coefficient (β) was estimated by fitting a least-squares regression line to the natural logarithm of the rates, using the calendar year as a regressor variable. Tests of the statistical significance of APCs and differences between APCs were based on methods proposed by Kleinbaum [21]. Subgroup analyses were performed in age groups and races/ethnicities. Mortality rates of tobacco-related cancers were calculated for 1975–2022. Statistics were not reported in cases where the count fell below 16, a measure to safeguard patient confidentiality by minimizing the risk of identity disclosure. This practice was also adopted due to the inherent unreliability of rates calculated from a small number of cases [22]. To account for differential probabilities of selection and the complex sample design, all analyses of NHIS data were weighted following NHIS analytics guidelines [23]. Final annual sample weights were applied to all analyses to adjust for non-response and post-stratification. Unreported statistics were handled as missing values.

## 4. Results

During 2001–2021, there were 14,099,363 cases of tobacco-related cancers among adults > 20 years old, including 1,177,367 cases in adults 20–49 years old, 4,167,151 cases in adults 50–64 years old and 8,754,845 cases in adults 65 years and older. In 2021, non-Hispanic Asians or Pacific Islanders had the lowest incidence among all age groups (Table 1). Non-Hispanic American Indians/Alaska Natives had the highest incidence among adults 20–49 years old (346.8 per 1,000,000) and 50–64 years old (2034.8 per 1,000,000), while non-Hispanic Whites had the highest incidence among adults 65 years and older (8821.8 per 1,000,000). People in non-metro areas had higher cancer incidence in all age groups compared to those in metro areas. People in the West had the lowest incidence across all age groups, whereas adults in the South had the highest incidence in the 20–49-year-old and 50-64-year-old groups, and adults in the Midwest had the highest incidence in the 65+ year old group. Among males, the top two sites with highest cancer incidence were colorectum and kidney in the 20–49-year-old group, colorectum and lung in the 50–64-year-old group, and lung and bladder in the 65+ year old group (Table 2). Among females, the top two sites with highest cancer incidence were colorectum and cervix in the 20–49-year-old group compared to lung and colorectum in the 50–64-year-old and 65+-year-old groups.

**Figure 1:**
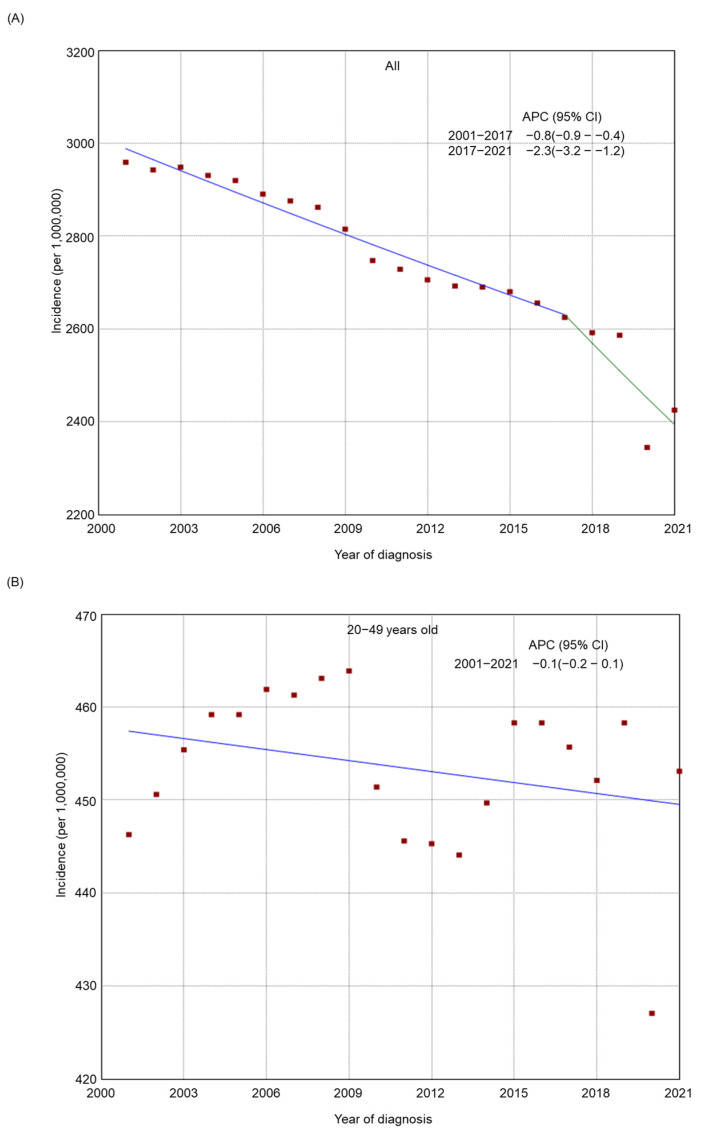

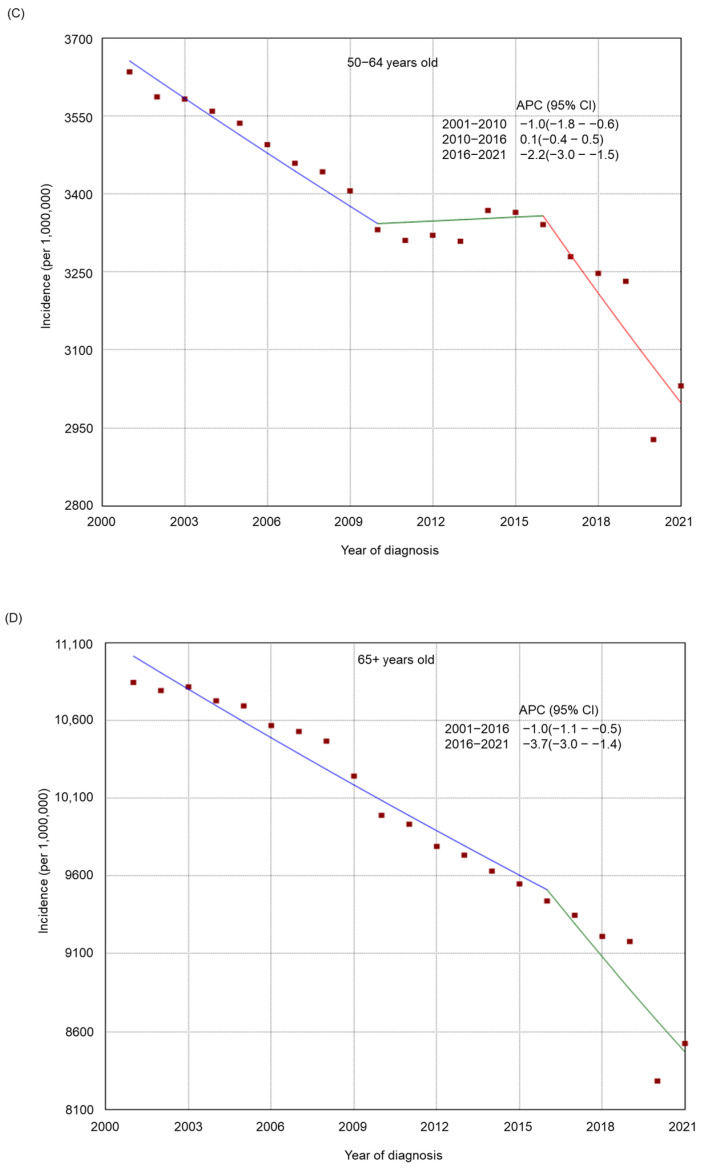
Adjusted incidence rates of tobacco-related cancers among adults in the United States from 2001 to 2021 by age group: (**A**) all; (**B**) 20–49 years old; (**C**) 50–64 years old; (**D**) 65+ years old. Red dots indicate the cancer incidence, while the lines represent trends in cancer incidence over time.

**Figure 2:**
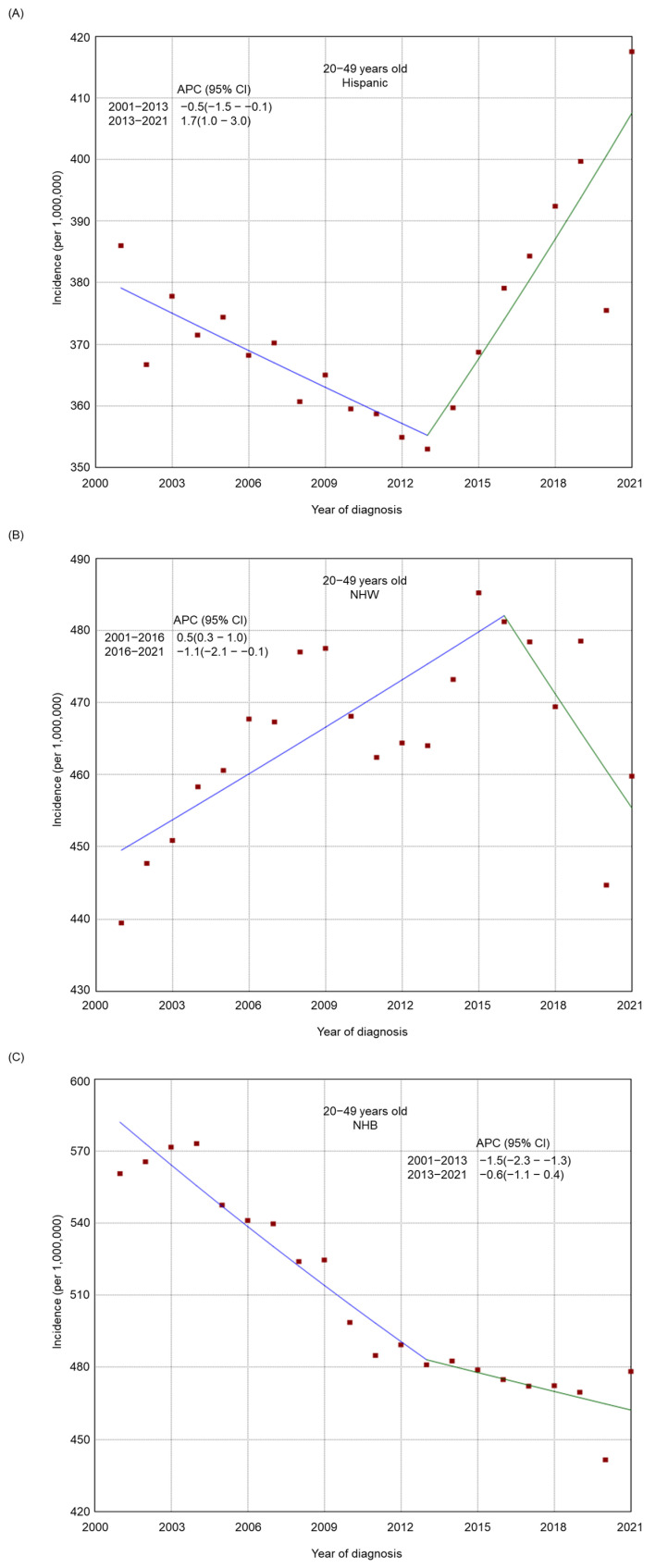

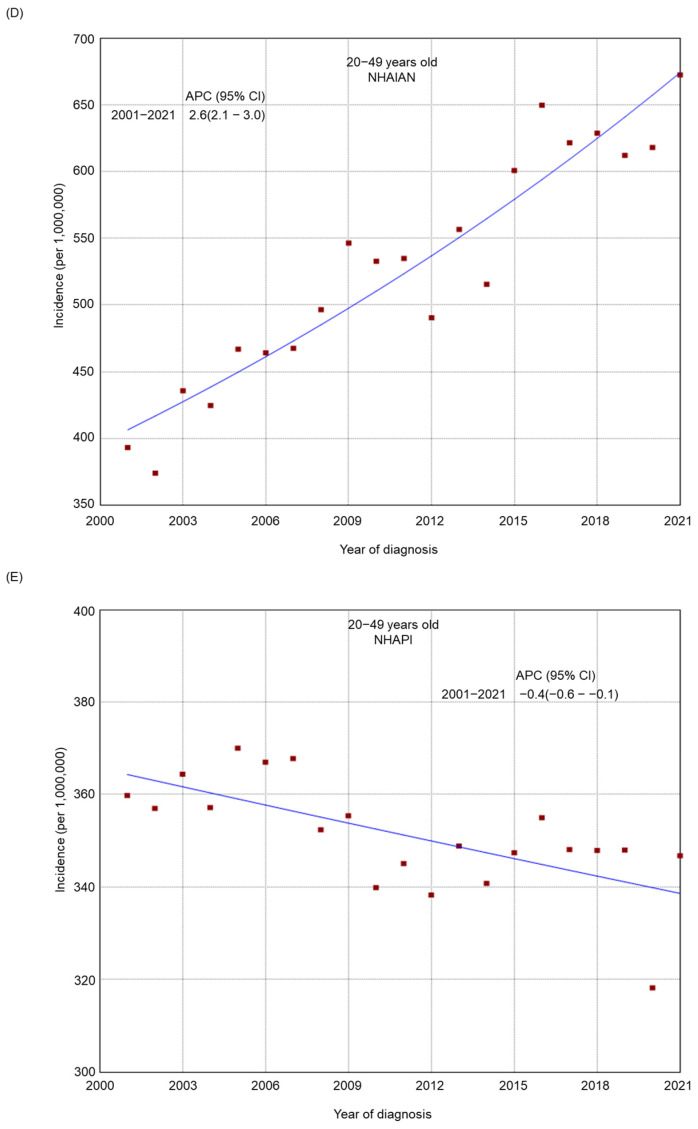
Adjusted incidence rates of tobacco-related cancers among adults 20–49 years old in the United States from 2001 to 2021 by race/ethnicity. (**A**) Hispanic. (**B**) Non-Hispanic White. (**C**) Non-Hispanic Black. (**D**) Non-Hispanic American Indian/Alaska Native. (**E**) Non-Hispanic Asian or Pacific Islander. NHW: Non-Hispanic White, NHB: Non-Hispanic Black, NHAIAN: Non-Hispanic American Indian/Alaska Native, NHAPI: Non-Hispanic Asian or Pacific Islander. Red dots indicate the cancer incidence, while the lines represent trends in cancer incidence over time.

**Figure 3:**
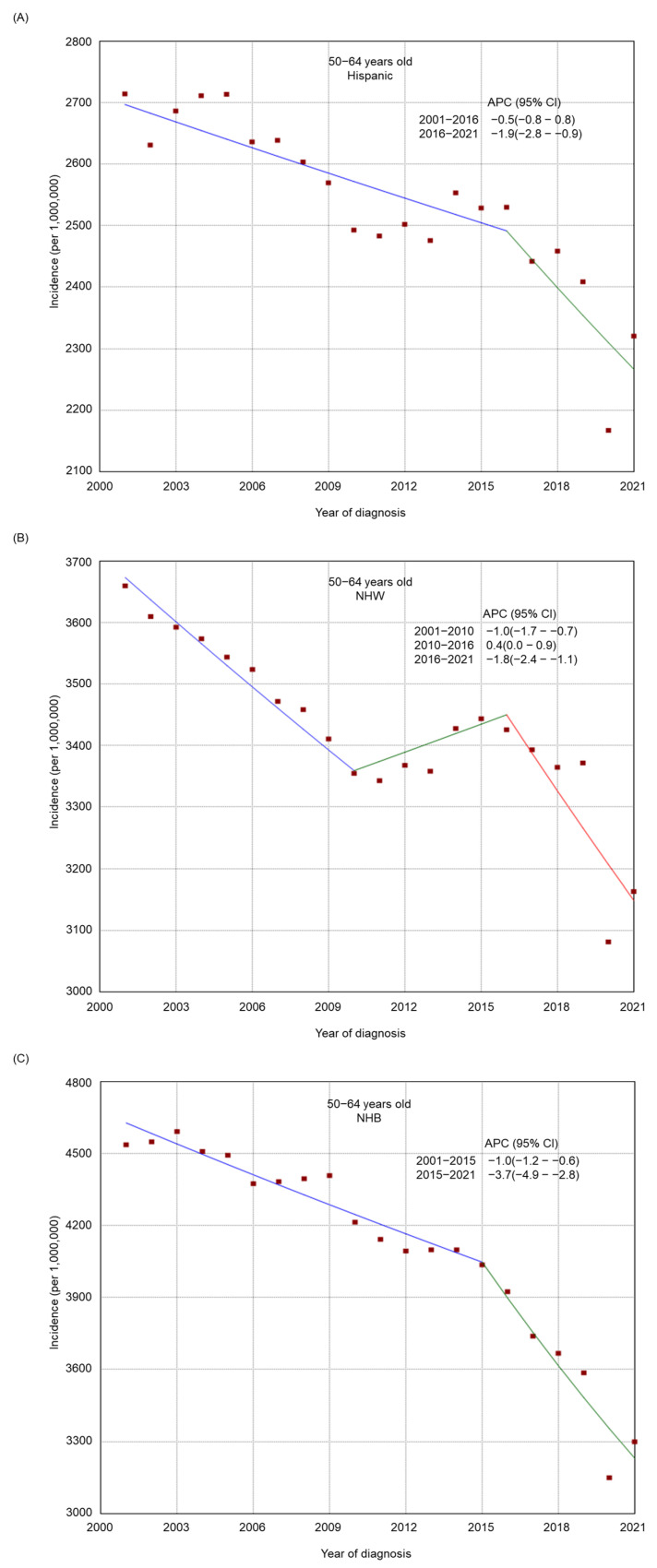

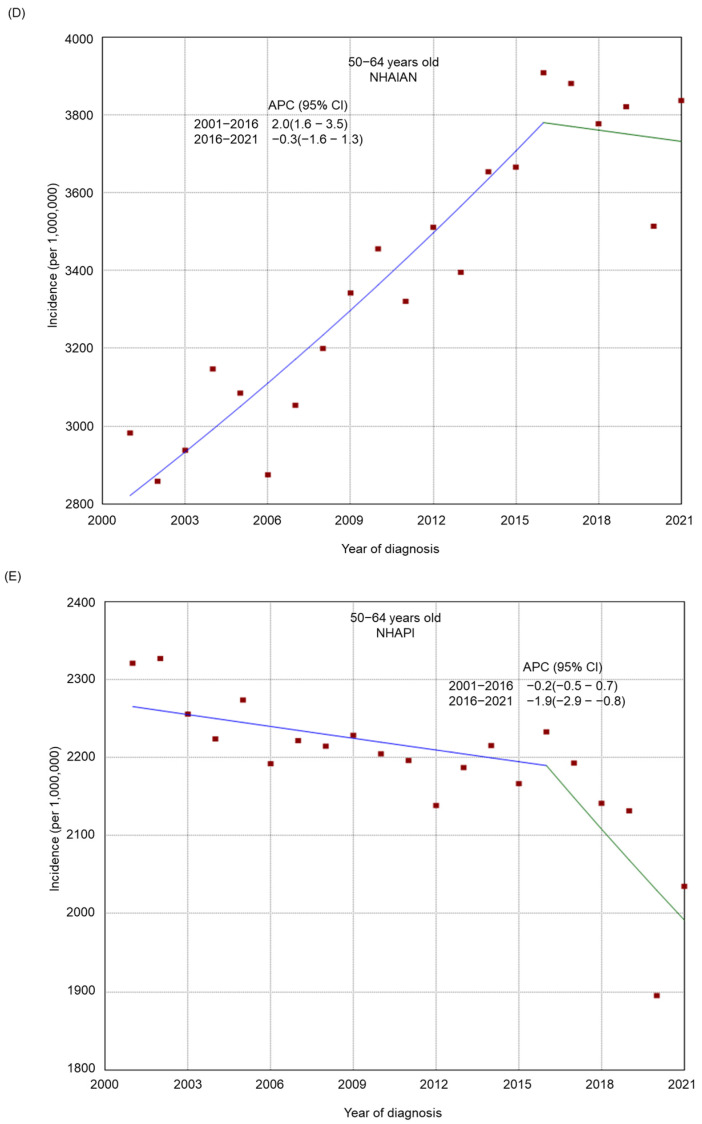
Adjusted incidence rates of tobacco-related cancers among adults 50–64 years old in the United States from 2001 to 2021 by race/ethnicity. (**A**) Hispanic. (**B**) Non-Hispanic White. (**C**) Non-Hispanic Black. (**D**) Non-Hispanic American Indian/Alaska Native. (**E**) Non-Hispanic Asian or Pacific Islander. Red dots indicate the cancer incidence, while the lines represent trends in cancer incidence over time.

**Figure 4:**
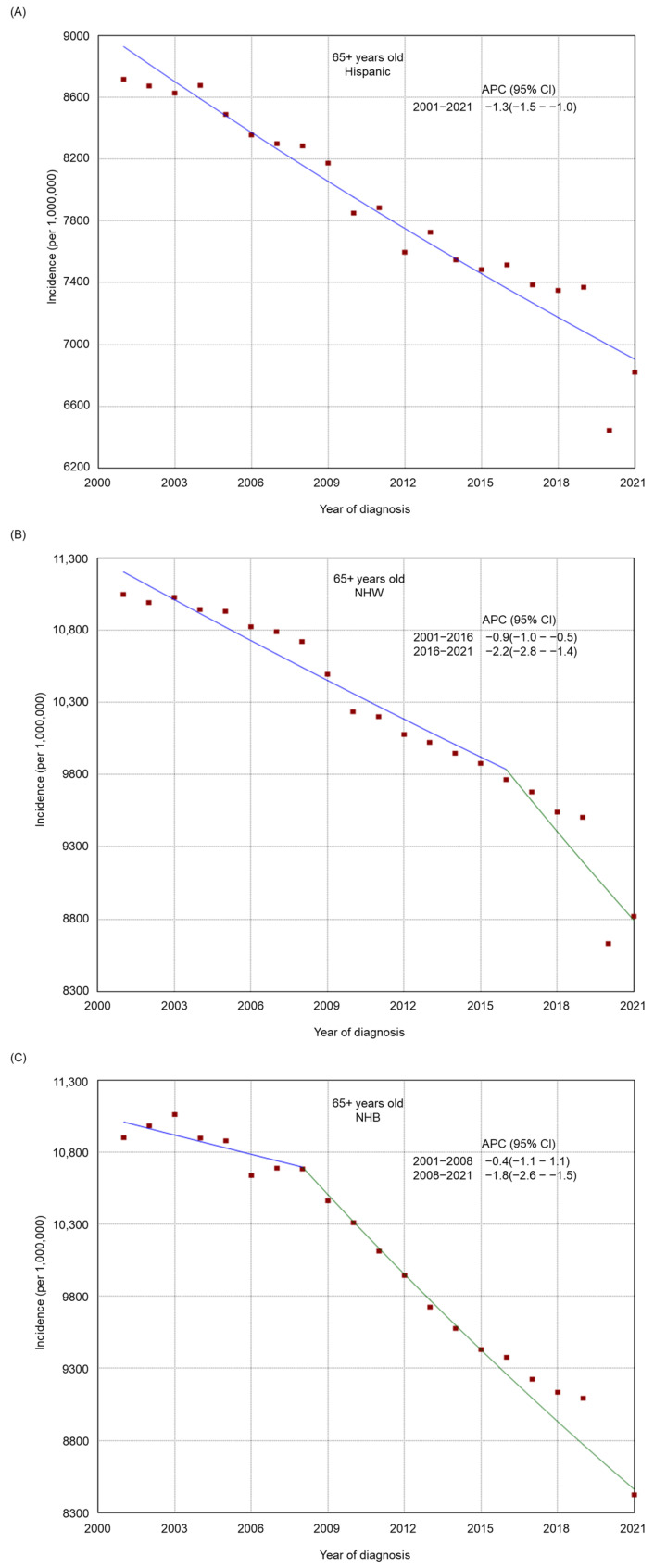

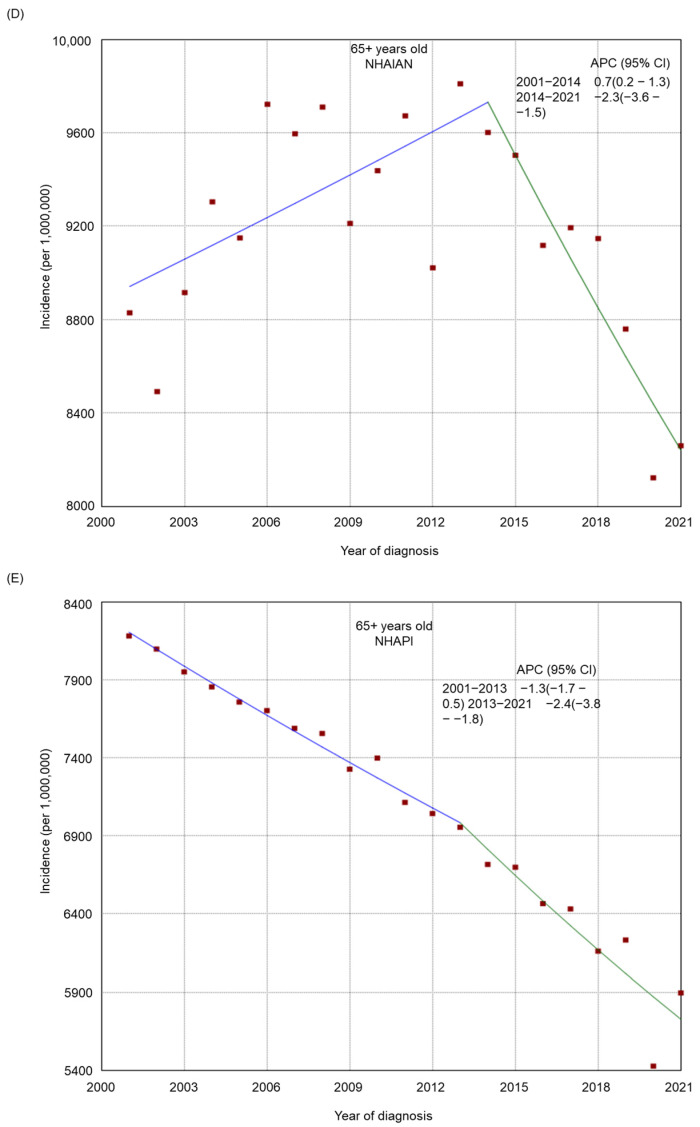
Adjusted incidence rates of tobacco-related cancers among adults 65 years and older in the United States from 2001 to 2021 by race/ethnicity. (**A**) Hispanic. (**B**) Non-Hispanic White. (**C**) Non-Hispanic Black. (**D**) Non-Hispanic American Indian/Alaska Native. (**E**) Non-Hispanic Asian or Pacific Islander. Red dots indicate the cancer incidence, while the lines represent trends in cancer incidence over time.

There was an overall decreasing trend in the incidence of tobacco-related cancers in the 50–64-year-old and 65+-year-old age groups from 2001 to 2021 (Figure 1), whereas the incidence was relatively stable among adults 20–49 years old (from 446.3 per 1,000,000 in 2001 to 453.1 per 1,000,000 in 2021, APC −0.1, 95% CI −0.2–0.1). There was a general declining trend in the incidence among most subgroups by sex (Figure S1), stage at diagnosis (Figure S2), race/ethnicity (Figure S3), residence type (Figure S4), and region of residence (Figure S5). From 2001 to 2021, a decreasing trend was also found in most racial/ethnic groups by age (20–49 years old, Figure 2; 50–64 years old, Figure 3; and 65+ years old, Figure 4), stage at diagnosis groups by age (Figures S6–S8), residence type by age (Figures S9–S11) and region of residence by age (Figures S12–S14). There was an increasing trend in the incidence among 20–49-year-old non-Hispanic American Indians/Alaska Natives (APC 2.6, 95% CI 2.1–3.0) and adults 20–49 years old in the West (APC 0.2, 95% CI 0.0–0.4, *p* = 0.04). Among non-Hispanic American Indians/Alaska Natives, there was initially an increasing trend, followed by a decreasing trend from 2014 to 2021 in the 65+ year old group (from 2001 to 2014, APC 0.7, 95% CI 0.2–1.3; from 2014 to 2021, APC −2.3, 95% CI −3.6–1.5) or a relatively stable incidence trend from 2016 to 2021 in the 50–64 years old (from 2001 to 2016, APC 2.0, 95% CI 1.6–3.5; from 2016 to 2021, APC −0.3, 95% CI −1.6–1.3). Among adults 20–49 years old, there was an upward trend most recently in Hispanics from 2013 to 2021 (from 2001 to 2013, APC −0.5, 95% CI −1.5–0.1; from 2013 to 2021, APC 1.7, 95% CI 1.0–3.0) and in those with regional cancer (from 2001 to 2013, APC −0.5, 95% CI −0.9–0.2; from 2013 to 2021, APC 1.5, 95% CI 0.9–2.3), whereas in those with metastasized cancer, there was first an upward trend followed by a decreasing trend (from 2001 to 2006, APC 3.2, 95% CI 1.9–4.2; from 2006 to 2021, APC −1.3, 95% CI −1.7–1.1).

There were 14,716,987 deaths caused by tobacco-related cancer among adults from 1975 to 2022, including 8,607,833 deaths in males and 6,109,154 in females (Table 3). In 2022, 342,744 deaths were caused by tobacco-related cancers, including 196,342 deaths in males and 146,402 in females. Trends in tobacco-related cancers mortality rates by sex and age group from 1975 to 2022 are shown in Figures S15–S17. There was an overall decrease trend in the 20–49-year-old group, both in males and females, while the mortality rate became stable in recent years in males. The mortality rate was stable in the first decade, then decreased sharply afterward among males 50–64 years old. There was first an increasing trend from 1975 to 1990 among females 50–64 years old and males 65+ years old and from 1975 to 2000 among females 65+ years old, followed by a decreasing trend since that time.

When restricting the analyses to the following group of cancers, lung, oral cavity, pharynx, larynx, bladder, and esophagus, we observed similar patterns of the incidence similar to the combined analyses of all tobacco-related cancers (Figures S18–S21). One exception is that, among 20–49-year-old non-Hispanic American Indians/Alaska Natives, cancer incidence continued to increase although not statistically significant (APC 0.3, 95% CI −0.6–1.3). For deaths caused by this group of cancers, we saw similar trends in the mortality rates to that caused by all tobacco-related cancers (Figures S22–S24). There was a steady decline in the mortality rate among adults 20–49 years old, while rates for those aged 50–64 years and 65+ years initially increased before subsequently decreased.

From 1983 to 2021, the prevalence of current tobacco smokers kept decreasing, especially among young and mid-aged adults (Figure S25). The South and Midwest always had higher prevalence of current smokers. The prevalence of current e-cigarette smokers increased from 2016 to 2021 among young adults, whereas this prevalence was relatively stable among adults 50–64 years old and 65+ years old (Figure S26). The prevalence of current e-cigarette smokers was also higher among non-Hispanic American Indian/Alaska Native individuals. Health insurance coverage remained steady among adults 65+ years old, whereas the coverage increased among young and mid-aged adults following the 2014 implementation of Affordable Care Act (Figure S27). Males, residents of the South and West, and Hispanics consistently had lowest health insurance coverage. However, after 2014, coverage in the West narrowed the gap with the Northeast and Midwest regions.

## 5. Discussion

Using data from USCS 2001–2021 database and mortality data 1975–2022 from NCHS, we assessed the trends in the incidence and mortality rates of tobacco-related cancers among adults in the United States. We found an overall decrease in both the incidence and mortality rate in the early 21st century. However, among adults 20–49 years old, there was an increasing trend in the incidence of tobacco-related cancers among non-Hispanic American Indians/Alaska Natives and those in the West, while the incidence increased most recently among Hispanics and those with regional cancer. The mortality rate became stable in recent years among males 20–49 years old. We saw an increasing trend in the mortality rates from 1975 to 1990 among females 50–64 years old and males 65+ years old and from 1975 to 2000 among females 65+ years old, whereas the mortality rates decreased in recent years in all age-sex groups. Targeted interventions are needed to address the emerging disparities in the incidence of tobacco-related cancers in younger adults, especially among Hispanics, non-Hispanic American Indians/Alaska Natives, and those in the West. Emerging studies indicate that newer tobacco products, such as electronic cigarettes (e-cigarettes), heated tobacco devices, and other vaping products, may be responsible for the increasing incidence of tobacco-related cancers in younger populations. Although marketed as safer substitutes for traditional cigarettes, many of these products contain known carcinogens, such as formaldehyde and acetaldehyde, [24] and are associated with oxidative stress and DNA damage. These mechanisms are implicated in cancer development. The popularity of e-cigarettes and vaping among those aged 18–49 years could contribute to the rising incidence of cancers in this demographic group. Our analyses of NHIS data showed that e-cigarette use among adults aged 18–49 years rose significantly between 2016 and 2021. The prevalence of e-cigarette use was also higher among non-Hispanic American Indian/Alaska Native population. Given that the carcinogenic impact of these products may latently, their role in the observed rise in tobacco-related cancers warrants further investigation.

We found a recent overall decrease in both the incidence and mortality rate of tobacco-related cancers in the United States. By comparison, the current global landscape shows a complex picture regarding trends in tobacco-related cancers. While the death rate of tobacco-related cancers worldwide decreased significantly by 23% from 1990 to 2019, there are stark differences in mortality across regions [3,4]. East Asia, Western Europe, and North America have the highest proportion of all cancer-related deaths attributable to smoking at approximately 30%, compared to Western Sub-Saharan Africa, Andean Latin America, and Eastern Sub-Saharan Africa, which are the lowest, at approximately 6%. In addition, there are gender discrepancies in smoking-related cancer deaths, such as a five-time higher lung cancer mortality rate in males compared to females [3,4]. Significant differences in incidence exist between countries as well [25,26]. For example, the incidence of lung cancer in the United States has declined gradually, while China is still on an upward trend in recent years [25]. This reflects a downward trend in smoking prevalence and increased effectiveness of smoking cessation campaigns [26]. However, in countries like China, with less control over the tobacco industry and difficulty implementing tobacco control policies [27,28,29], incidence rates remain high. Certain types of cancer caused by tobacco use are also more prevalent in different countries. The South Asian region (India, Pakistan, Afghanistan, Bangladesh, Sri Lanka, Bhutan, Nepal, Iran, and Maldives) is disproportionately affected by oral cancer due to comparatively increased consumption of chewing tobacco [30]. Age-adjusted rates of oral cancer in India accounts for almost a third of all cancers in the country and is ranked as one of the top three cancers [31]. In contrast, oropharyngeal cancer incidence rate is comparatively less common among adults in the United States, due to different cultural tobacco consumption practices [11,12].

The findings of the current study show that, despite a substantial decrease in the overall incidence rate of tobacco-related cancers in the United States, there are emerging disparities within specific demographic groups and regions. In particular, younger adults (20–49 years) of non-Hispanic American Indian/Alaskan Natives experienced a rise in incidence rates of tobacco-related cancers. This aligns with previous research showing persistently high smoking prevalence and fundamental barriers to effective tobacco control in this population [32]. These communities are much more susceptible to unique challenges, including cultural reasons for tobacco use, underfunded public health infrastructure, and limited access to tobacco cessation programs due to concentration of non-Hispanic American Indian/Alaskan Native populations in non-metropolitan areas with fewer resources. Addressing these disparities requires culturally tailored interventions and targeted policies prioritizing prevention and cessation efforts for non-Hispanic American Indian/Alaskan Native populations.

The rise in the incidence of tobacco-related cancers among younger Hispanics in the United States may be attributable, in part, to various systemic, socioeconomic, and cultural factors. Many Hispanic individuals face cultural and linguistic challenges; almost 40% of Spanish-speakers reporting lack of proficiency in English, which could create difficulties in access to preventive services or navigation of the U.S. healthcare system [33]. Other significant barriers include high rates of uninsured Hispanic adults, leading to limited access to tobacco cessation services, and mistrust of medical providers due to historically negative interactions, perpetuating health inequities [34]. Interestingly, the rising incidence rates of tobacco-related cancers among younger Hispanic adults can, in part, be attributed to the effects of acculturation. Acculturation is the process of adapting to a new cultural environment and picking up new cultural traits and practices while still retaining their own customs. Research shows that acculturation is associated with increased tobacco use, particularly among U.S.-born Hispanics, due to the adoption of less restrictive smoking norms prevalent in U.S. culture [35]. Furthermore, acculturation increases exposure to tobacco advertising, with the U.S. tobacco industry consistently targeting Hispanic communities disproportionately: culturally relevant advertisements, partnerships with Hispanic-focused events and organizations, and discounts on tobacco products in predominantly Hispanic neighborhoods are major contributors to these adverse health outcomes. The slow disappearance of protective cultural norms and traditional attitudes discouraging smoking underscores the need for culturally sensitive interventions as well as strategies for counter-marketing harmful advertising.

Region-specific trends demonstrated a slight increase in incidence among young adults in the West, which could reflect geographic variations in socioeconomic factors or healthcare access in the United States. We also observed geographic disparities in health insurance coverage. Coverage was consistently lowest among those in the South and West, while the coverage in the West narrowed the gap following the 2014 implementation of the Affordable Care Act. On the other hand, the prevalence of current smokers was consistently higher among those in the South and Midwest. Previous studies have shown that disparities in tobacco use across the United States are partially due to the inequitable implementation of tobacco control policies [6]. These differences may also reflect varying levels of healthcare infrastructure and economic hardship. Awareness of these discrepancies can help to create targeted policy interventions that allow for equal access to resources. Expanding comprehensive tobacco prevention programs and improving access to care in these areas are essential steps toward reducing these inequities.

The mortality analysis from 1975 to 2022 revealed overall declines in tobacco-related cancer deaths, attributed to advancements in medical care, early detection, and reduced smoking prevalence through public health campaigns. Cancer-specific death rate is not easily affected by detection bias results from cancer screening efforts. It can better monitor progress against cancer than cancer incidence and cancer-specific survival [11,12,36]. Among adults aged 50–64 and 65+ years, mortality rates declined sharply. This reflects the impact of tobacco control measures implemented by the Department of Health and Human Services of the United States in the late 20th century, which dramatically reduced the prevalence of adult smoking by almost half [37]. In contrast, mortality rates plateaued for males aged 20–49 in recent years, suggesting that a focus on younger male populations must be re-established, and the effectiveness of current interventions may require re-evaluation. Males, specifically, consistently had higher tobacco-related cancer mortality rates as compared to women, which was in line with previous research on global burdens of cancer attributable to tobacco use, that identified the global age-standardized cancer-related deaths attributable to smoking as much greater in men than among women [4]. In terms of cancer-specific mortality, lung cancer remains the leading cause of cancer over all age groups. However, mortality rates have declined significantly due to the reduction in smoking rates overall and better treatment options. The 2014 implementation of the Affordable Care Act may also partly contribute to decline of mortality rate among adults in recent years [38,39,40,41].

## 6. Strength and Limitations

A major strength of this study is that we used data that covered the entire adult population of the United States, which provided a full picture of disease burden for tobacco-related cancers. We were also able to conduct granular subgroup analyses of the incidence and mortality of tobacco-related cancers using these large datasets. A substantial limitation of this study is that we do not have data on risk factors, particularly tobacco-related behaviors, for these cancers. Moreover, we did not have data on prevention efforts at the individual level for the cancer under study. There were also reporting errors of race and ethnicity in medical records and death certificates, which may affect incidence estimates among racial/ethnic groups. Additionally, this study did not have detailed information correlated to the disparities and increased incidence in the 20–40 age groups. Furthermore, we also lacked data on gender identity and sexual orientation to provide incidence estimates for the LGBTQ+ population who are reported to have higher smoking prevalence [42].

## 7. Conclusions

There was a general decline in the incidence (from 2001 to 2021) and mortality (from 2001 to 2022) of tobacco-rated cancers among adults in the United Sates. However, the rising incidence of these cancers in younger Hispanics, non-Hispanic American Indians/Alaska Natives, and those in the West is concerning and underscores the need for focused public health interventions to address disparities and improve cancer prevention in these vulnerable groups. Our findings reveal generally favorable trends in reduction in both incidence and mortality of tobacco-related cancers in older age groups, while trends among some younger groups and marginalized populations signal areas of concern. These results emphasize the importance of continued tobacco control measures, improved access to preventive care, and tailored public health strategies to address disparities and maintain a downward trend.

## Figures and Tables

**Table 1:** Age adjusted tobacco-related cancers incidence among adults in the United States in 2021 by age group, sex, race/ethnicity, and region of residence.

	20–49		50–64		65+	
	Count	Rate	Count	Rate	Count	Rate
**All**	53,735	453.1(449.2–456.9)	201,570	3031.1(3017.6–3044.6)	456,459	8524.9(8499.9–8550.0)
**Race/ethnicity**						
Hispanic	10,340	417.5(409.5–425.7)	21,094	2320.5(2289.2–2352.0)	32,733	6820.5(6745.2–6896.4)
NHW	31,048	459.8(454.7–465.0)	141,691	3163.0(3146.0–3180.0)	357,618	8821.8(8792.6–8851.0)
NHB	7572	478.3(467.5–489.3)	26,842	3299.2(3259.3–3339.5)	43,387	8424.8(8343.3–8506.9)
NHAIAN	646	672.3(621.1–726.5)	1957	3836.5(3664.9–4014.2)	2881	8260.8(7953.4–8577.2)
NHAPI	3297	346.8(335.0–358.9)	7900	2034.8(1990.0–2080.5)	16,323	5896.6(5805.1–5989.1)
**Stage**						
Localized	20,891	175.3(172.9–177.7)	65,463	991.8(984.1–999.6)	152,884	2852.9(2838.5–2867.4)
Regional	15,587	132.1(130.0–134.2)	58,096	879.5(872.2–886.8)	108,810	2014.2(2002.1–2026.4)
Distant	13,726	115.9(114.0–117.9)	60,000	893.8(886.5–901.1)	129,846	2421.1(2407.8–2434.5)
**Residence type**						
Metro	45,879	441.9(437.9–446.0)	165,842	2923.1(2908.8–2937.5)	375,917	8415.9(8388.7–8443.2)
Non-metro	7844	529.8(518.1–541.7)	35,670	3664.1(3624.9–3703.6)	80,441	9060.4(8997.2–9124.0)
**Region of Residence**						
Northeast	9101	444.7(435.6–454.0)	37,293	2977.4(2946.6–3008.4)	87,564	8744.1(8685.7–8802.9)
Midwest	10,276	469.9(460.9–479.1)	42,359	3249.0(3217.4–3280.9)	95,399	9066.7(9008.4–9125.2)
South	22,506	482.6(476.3–489.0)	84,785	3295.7(3273.1–3318.3)	182,342	8768.8(8728.1–8809.6)
West	11,852	399.7(392.5–407.0)	37,133	2444.9(2419.7–2470.3)	91,154	7470.8(7421.8–7520.0)
**Male**	26,969	453.4(448.0–458.8)	121,562	3693.7(3672.6–3714.9)	265,028	11,224.4(11,180.8–11,268.2)
**Race/ethnicity**						
Hispanic	4765	374.3(363.7–385.2)	12,480	2738.9(2691.0–2787.4)	18,457	8939.5(8806.0–9074.5)
NHW	16,069	469.3(462.1–476.7)	86,498	3859.3(3833.0–3885.9)	209,913	11,565.7(11,515.4–11,616.2)
NHB	3755	498.7(482.8–515.1)	15,391	4042.3(3977.9–4107.5)	23,623	11,382.1(11,228.8–11,536.9)
NHAIAN	310	655.1(583.6–732.5)	1159	4723.2(4450.6–5008.3)	1548	9924.4(9416.3–10,453.3)
NHAPI	1650	362.9(345.5–380.9)	4689	2567.7(2494.4–2642.6)	9282	7760.5(7600.4–7923.1)
**Stage**						
Localized	9534	159.7(156.5–162.9)	38,040	1162.2(1150.3–1174.1)	88,001	3727.4(3702.2–3752.6)
Regional	8041	136.0(133.0–139.0)	36,245	1109.5(1097.9–1121.2)	62,545	2587.5(2566.8–2608.3)
Distant	7385	124.0(121.2–126.9)	35,133	1059.9(1048.6–1071.2)	71,994	3033.9(3011.2–3056.6)
**Residence type**						
Metro	22,983	443.3(437.6–449.1)	99,813	3565.1(3542.7–3587.6)	216,583	11,080.2(11,032.6–11,128.1)
Non-metro	3980	520.3(504.2–536.7)	21,720	4433.7(4373.2–4494.9)	48,383	11,900.6(11,792.3–12,009.6)
**Region of Residence**						
Northeast	4605	452.3(439.3–465.6)	22,293	3611.9(3563.7–3660.6)	49,620	11,498.7(11,395.6–11,602.6)
Midwest	5337	483.7(470.8–496.9)	25,620	3935.2(3886.1–3984.8)	55,422	11,917.9(11,816.3–12,020.2)
South	11,161	481.2(472.3–490.2)	51,150	4055.9(4020.3–4091.8)	106,796	11,659.6(11,588.2–11,731.3)
West	5866	388.8(378.9–398.9)	22,499	2957.5(2918.5–2997.0)	53,190	9708.4(9624.2–9793.0)
**Female**	26,766	453.3(447.9–458.8)	80,008	2387.2(2370.3–2404.2)	191,431	6431.1(6402.0–6460.2)
**Race/ethnicity**						
Hispanic	5575	464.9(452.7–477.3)	8614	1905.0(1864.9–1945.8)	14,276	5249.8(5162.9–5337.9)
NHW	14,979	450.1(442.9–457.3)	55,193	2470.0(2448.7–2491.4)	147,705	6636.6(6602.5–6670.8)
NHB	3817	460.8(446.2–475.8)	11,451	2650.7(2601.6–2700.5)	19,764	6476.1(6384.3–6568.8)
NHAIAN	336	690.2(617.8–768.4)	798	3019.4(2808.5–3242.1)	1333	6954.7(6578.0–7347.4)
NHAPI	1647	332.7(316.8–349.2)	3211	1566.9(1512.8–1622.5)	7041	4484.4(4378.8–4592.0)
**Stage**						
Localized	11,357	191.5(188.0–195.1)	27,423	826.5(816.5–836.6)	64,883	2181.8(2164.9–2198.8)
Regional	7546	128.3(125.5–131.3)	21,851	655.5(646.7–664.5)	46,265	1555.0(1540.7–1569.3)
Distant	6341	107.7(105.1–110.4)	24,867	732.4(723.2–741.8)	57,852	1944.6(1928.7–1960.7)
**Residence type**						
Metro	22,896	441.1(435.4–446.9)	66,029	2302.6(2284.7–2320.6)	159,334	6377.4(6345.8–6409.1)
Non-metro	3864	540.7(523.8–558.1)	13,950	2892.8(2843.2–2943.1)	32,058	6704.2(6630.5–6778.6)
**Region of Residence**						
Northeast	4496	437.8(425.1–450.9)	15,000	2366.7(2328.1–2405.8)	37,944	6705.1(6637.0–6773.6)
Midwest	4939	456.0(443.4–469.0)	16,739	2570.0(2530.1–2610.4)	39,977	6855.6(6787.8–6924.0)
South	11,345	484.6(475.7–493.6)	33,635	2569.4(2541.4–2597.5)	75,546	6527.3(6480.5–6574.4)
West	5986	411.8(401.3–422.4)	14,634	1936.2(1904.4–1968.5)	37,964	5670.6(5613.2–5728.5)

Rate: cases per 1,000,000 persons. Rates were age-adjusted to the 2000 U.S. standard population. NHW: Non-Hispanic White, NHB: Non-Hispanic Black, NHAIAN: Non-Hispanic American Indian/Alaska Native, NHAPI: Non-Hispanic Asian or Pacific Islander.

**Table 2:** Age-adjusted tobacco-related cancers incidence among adults in the United States in 2021 by cancer site.

	20–49		50–64		65+	
	Count	Rate	Count	Rate	Count	Rate
**All**						
Lip, oral cavity, and pharynx	4555	38.4(37.3–39.5)	18,853	286.3(282.1–290.5)	25,652	466.1(460.4–471.9)
Esophagus	832	7.1(6.7–7.6)	5696	84.2(82.0–86.5)	12,106	224.0(220.0–228.0)
Stomach	2783	23.5(22.6–24.4)	7690	116.9(114.3–119.6)	16,488	308.3(303.5–313.1)
Colon and rectum	17,567	148.4(146.2–150.6)	44,695	708.1(701.4–714.9)	79,168	1485.5(1475.1–1496.0)
Liver	1088	9.1(8.6–9.6)	9003	130.0(127.3–132.7)	18,154	328.5(323.7–333.4)
Pancreas	2634	22.3(21.5–23.2)	14,009	208.0(204.5–211.6)	39,000	733.6(726.2–741.0)
Larynx	573	4.9(4.5–5.3)	4421	65.2(63.3–67.2)	6628	120.3(117.3–123.2)
Trachea, lung, and bronchus	4812	41.1(40.0–42.3)	52,948	761.7(755.1–768.3)	151,869	2841.5(2827.1–2856.0)
Cervix uteri	6128	103(100.4–105.6)	3670	118.2(114.3–122.2)	2735	90.1(86.7–93.6)
Kidney and renal pelvis	8722	74.0(72.4–75.6)	22,716	349.4(344.8–354.1)	36,150	661.1(654.2–668.1)
Urinary bladder	2104	17.8(17.0–18.6)	14,769	215.1(211.6–218.7)	58,538	1116.2(1107.1–1125.4)
Acute myeloid leukemia	1937	15.5(14.8–16.2)	3100	46.5(44.9–48.2)	9971	190.2(186.5–194.0)
**Male**						
Lip, oral cavity, and pharynx	3009	50.7(48.9–52.5)	14,248	438(430.7–445.4)	17,792	717.7(707–728.6)
Esophagus	684	11.6(10.8–12.5)	4550	136.4(132.4–140.5)	9474	394.1(386–402.3)
Stomach	1411	23.6(22.4–24.9)	4610	140.6(136.5–144.8)	9895	420.9(412.5–429.5)
Colon and rectum	9224	155(151.8–158.2)	25,841	824.5(814.3–834.8)	39,770	1695.90(1678.90–1713.00)
Liver	703	11.7(10.8–12.6)	7116	208.7(203.8–213.7)	12,986	518.7(509.6–527.9)
Pancreas	1383	23.4(22.2–24.7)	7948	239.7(234.3–245.1)	19,672	839.6(827.6–851.7)
Larynx	396	6.8(6.1–7.5)	3357	100(96.6–103.5)	5422	221.5(215.5–227.6)
Trachea, lung, and bronchus	2352	40(38.4–41.6)	25,974	756.8(747.5–766.2)	76,548	3243.80(3220.40–3267.40)
Kidney and renal pelvis	5357	90.3(87.9–92.8)	15,070	469.1(461.5–476.8)	22,788	930.6(918.3–943.1)
Urinary bladder	1530	25.7(24.4–27)	11,107	327.3(321.2–333.6)	45,005	1991.50(1972.70–2010.30)
Acute myeloid leukemia	920	14.5(13.6–15.5)	1741	52.7(50.2–55.3)	5676	250.1(243.5–256.9)
**Female**						
Lip, oral cavity, and pharynx	1546	26(24.7–27.3)	4605	138.3(134.2–142.4)	7860	260.8(255.1–266.7)
Esophagus	148	2.5(2.2–3)	1146	33.5(31.6–35.6)	2632	88.3(85–91.8)
Stomach	1372	23.3(22.1–24.6)	3080	94(90.6–97.4)	6593	221(215.7–226.5)
Colon and rectum	8343	141.8(138.8–144.9)	18,854	594.5(585.8–603.2)	39,398	1318.70(1305.50–1331.90)
Liver	385	6.4(5.8–7.1)	1887	53.7(51.2–56.2)	5168	172.2(167.5–177)
Pancreas	1251	21.2(20.1–22.5)	6061	177.2(172.7–181.8)	19,328	650.3(641.1–659.7)
Larynx	177	3(2.6–3.5)	1064	31.5(29.6–33.5)	1206	39.5(37.3–41.8)
Trachea, lung, and bronchus	2460	42.3(40.6–44)	26,974	766.9(757.6–776.3)	75,321	2543.30(2525.00–2561.70)
Cervix uteri	6128	103(100.4–105.6)	3670	118.2(114.3–122.2)	2735	90.1(86.7–93.6)
Kidney and renal pelvis	3365	57.4(55.5–59.4)	7646	232.6(227.3–238)	13,362	444.6(437–452.2)
Urinary bladder	574	9.8(9–10.6)	3662	106.3(102.9–109.9)	13,533	457(449.2–464.8)
Acute myeloid leukemia	1017	16.5(15.5–17.5)	1359	40.6(38.4–42.9)	4295	145.2(140.9–149.7)

Rate: cases per 1,000,000 persons. Rates were age-adjusted to the 2000 U.S. standard population.

**Table 3:** Deaths caused by tobacco-related cancers among adults in the United States from 1975 to 2022 by sex and age group.

	Death Count			
**Age group**	20–49	50–64	65+	20+
**1975–2022**				
All	791,015	3,765,025	10,160,947	14,716,987
Males	445,025	2,386,300	5,776,508	8,607,833
Females	345,990	1,378,725	4,384,439	6,109,154
**2022**				
All	12,401	75,988	254,355	342,744
Males	6785	45,814	143,743	196,342
Females	5616	30,174	110,612	146,402

## Data Availability

Data used in this study are United States Cancer Statistics public use databases and mortality data from the National Center for Health Statistics (NCHS), which are publicly available through the Centers for Disease Control and Prevention (CDC) website. It has established procedures for accessing the data. We will direct interested parties to the appropriate contacts to request access. Methods for our analysis would be made available upon request.

## Supplementary Materials

The following supporting information can be downloaded at: https://www.mdpi.com/article/10.3390/cancers17030534/s1, Figure S1. Adjusted incidence rates of tobacco-related cancers among adults in the United States from 2001 to 2021 by sex. A. Male. B. Female. Figure S2. Adjusted incidence rates of tobacco-related cancers among adults in the United States from 2001 to 2021 by stage at diagnosis. A. Localized. B. Regional. C. Distant. Figure S3. Adjusted incidence rates of tobacco-related cancers among adults in the United States from 2001 to 2021 by race/ethnicity. A. Hispanic. B. Non-Hispanic White. C. Non-Hispanic Black. D. Non-Hispanic American Indian/Alaska Native. E. Non-Hispanic Asian or Pacific Islander. NHW: Non-Hispanic White, NHB: Non-Hispanic Black, NHAIAN: Non-Hispanic American Indian/Alaska Native, NHAPI: Non-Hispanic Asian or Pacific Islander. Figure S4. Adjusted incidence rates of tobacco-related cancers among adults in the United States from 2001 to 2021 by residence type. A. Metro. B. Non-metro. Figure S5. Adjusted incidence rates of tobacco-related cancers among adults in the United States from 2001 to 2021 by region of residence. A. Northeast. B. Midwest. C. South. D. West. Figure S6. Adjusted incidence rates of tobacco-related cancers among adults 20–49 years old in the United States from 2001 to 2021 by stage at diagnosis. A. Localized. B. Regional. C. Distant. Figure S7. Adjusted incidence rates of tobacco-related cancers among adults 50–64 years old in the United States from 2001 to 2021 by stage at diagnosis. A. Localized. B. Regional. C. Distant. Figure S8. Adjusted incidence rates of tobacco-related cancers among adults 65 years and older in the United States from 2001 to 2021 by stage at diagnosis. A. Localized. B. Regional. C. Distant. Figure S9. Adjusted incidence rates of tobacco-related cancers among adults 20–49 years old in the United States from 2001 to 2021 by residence type. A. Metro. B. Non-metro. Figure S10. Adjusted incidence rates of tobacco-related cancers among adults 50–64 years old in the United States from 2001 to 2021 by residence type. A. Metro. B. Non-metro. Figure S11. Adjusted incidence rates of tobacco-related cancers among adults 65 years and older in the United States from 2001 to 2021 by residence type. A. Metro. B. Non-metro. Figure S12. Adjusted incidence rates of tobacco-related cancers among adults 20–49 years old in the United States from 2001 to 2021 by region of residence. A. Northeast. B. Midwest. C. South. D. West. Figure S13. Adjusted incidence rates of tobacco-related cancers among adults 50–64 years old in the United States from 2001 to 2021 by region of residence. A. Northeast. B. Midwest. C. South. D. West. Figure S14. Adjusted incidence rates of tobacco-related cancers among adults 65 years and older in the United States from 2001 to 2021 by region of residence. A. Northeast. B. Midwest. C. South. D. West. Figure S15. Mortality rates of tobacco-related cancers among adults 20–49 years old in the United States from 1975 to 2022 by sex. A. All. B. Male. C. Female. Figure S16. Mortality rates of tobacco-related cancers among adults 50–64 years old in the United States from 1975 to 2022 by sex. A. All. B. Male. C. Female. Figure S17. Mortality rates of tobacco-related cancers among adults 65+ years old in the United States from 1975 to 2022 by sex. A. All. B. Male. C. Female. Figure S18. Adjusted incidence rates of cancers of lung, oral cavity, pharynx, larynx, bladder, and esophagus among adults in the United States from 2001 to 2021 by age group. A. All. B. 20–49 years old. C. 50–64 years old. D. 65+ years old. Figure S19. Adjusted incidence rates of cancers of lung, oral cavity, pharynx, larynx, bladder, and esophagus among adults 20-49 years old in the United States from 2001 to 2021 by race/ethnicity. A. Hispanic. B. Non-Hispanic White. C. Non-Hispanic Black. D. Non-Hispanic American Indian/Alaska Native. E. Non-Hispanic Asian or Pacific Islander. NHW: Non-Hispanic White, NHB: Non-Hispanic Black, NHAIAN: Non-Hispanic American Indian/Alaska Native, NHAPI: Non-Hispanic Asian or Pacific Islander. Figure S20. Adjusted incidence rates of cancers of lung, oral cavity, pharynx, larynx, bladder, and esophagus among adults 50–64 years old in the United States from 2001 to 2021 by race/ethnicity. A. Hispanic. B. Non-Hispanic White. C. Non-Hispanic Black. D. Non-Hispanic American Indian/Alaska Native. E. Non-Hispanic Asian or Pacific Islander. Figure S21. Adjusted incidence rates of cancers of lung, oral cavity, pharynx, larynx, bladder, and esophagus among adults 65 years and older in the United States from 2001 to 2021 by race/ethnicity. A. Hispanic. B. Non-Hispanic White. C. Non-Hispanic Black. D. Non-Hispanic American Indian/Alaska Native. E. Non-Hispanic Asian or Pacific Islander. Figure S22. Mortality rates of cancers of lung, oral cavity, pharynx, larynx, bladder, and esophagus among adults 20–49 years old in the United States from 1975 to 2022 by sex. A. All. B. Male. C. Female. Figure S23. Mortality rates of cancers of lung, oral cavity, pharynx, larynx, bladder, and esophagus among adults 50–64 years old in the United States from 1975 to 2022 by sex. A. All. B. Male. C. Female. Figure S24. Mortality rates of cancers of lung, oral cavity, pharynx, larynx, bladder, and esophagus among adults 65+ years old in the United States from 1975 to 2022 by sex. A. All. B. Male. C. Female. Figure S25. Prevalence of current tobacco smokers among adults in the United States from 1983 to 2021 by age, sex, region of residence, and race/ethnicity. A. Age. B. Sex. C. Region of residence. D. Race/ethnicity. NHW: Non-Hispanic White, NHB: Non-Hispanic Black. Figure S26. Prevalence of current e-cigarette smokers among adults in the United States from 2016 to 2021 by age, sex, region of residence, and race/ethnicity. A. Age. B. Sex. C. Region of residence. D. Race/ethnicity. NHW: Non-Hispanic White, NHB: Non-Hispanic Black, NHAIAN: Non-Hispanic American Indian/Alaska Native, NHAPI: Non-Hispanic Asian or Pacific Islander. Figure S27. Health insurance coverage among adults in the United States from 2000 to 2021 by age, sex, region of residence, and race/ethnicity. A. Age. B. Sex. C. Region of residence. D. Race/ethnicity. NHW: Non-Hispanic White, NHB: Non-Hispanic Black.
