# Supplementary material for: Trends in the Incidence and Mortality of Tobacco-Related Cancers Among Adults in the United States
Source: Cancers (Basel). 2025 Feb 5;17(3):534. doi: 10.3390/cancers17030534 (PMC11817473; doi:10.3390/cancers17030534)
Supplement: Supplementary file 1 [file cancers-17-00534-s001.zip › cancers-3399220-supplementary.pdf]

Figure legends.

Figure S1. Adjusted incidence rates of tobacco-related cancers among adults in the United States from 2001 to 2021 by sex.
A. Male. B. Female.

Figure S2. Adjusted incidence rates of tobacco-related cancers among adults in the United States from 2001 to 2021 by stage at diagnosis.
A. Localized. B. Regional. C. Distant.

Figure S3. Adjusted incidence rates of tobacco-related cancers among adults in the United States from 2001 to 2021 by race/ethnicity.
A. Hispanic. B. Non-Hispanic White. C. Non-Hispanic Black. D. Non-Hispanic American Indian/Alaska Native. E. Non-Hispanic Asian or Pacific Islander.
NHW: Non-Hispanic White, NHB: Non-Hispanic Black, NHAIAN: Non-Hispanic American Indian/Alaska Native, NHAPI: Non-Hispanic Asian or Pacific Islander.

Figure S4. Adjusted incidence rates of tobacco-related cancers among adults in the United States from 2001 to 2021 by residence type.
A. Metro. B. Non-metro.

Figure S5. Adjusted incidence rates of tobacco-related cancers among adults in the United States from 2001 to 2021 by region of residence.
A. Northeast. B. Midwest. C. South. D. West.

Figure S6. Adjusted incidence rates of tobacco-related cancers among adults 20-49 years old in the United States from 2001 to 2021 by stage at diagnosis.
A. Localized. B. Regional. C. Distant.

Figure S7. Adjusted incidence rates of tobacco-related cancers among adults 50-64 years old in the United States from 2001 to 2021 by stage at diagnosis.
A. Localized. B. Regional. C. Distant.

Figure S8. Adjusted incidence rates of tobacco-related cancers among adults 65 years and older in the United States from 2001 to 2021 by stage at diagnosis.
A. Localized. B. Regional. C. Distant.

Figure S9. Adjusted incidence rates of tobacco-related cancers among adults 20-49 years old in the United States from 2001 to 2021 by residence type.
A. Metro. B. Non-metro.

Figure S10. Adjusted incidence rates of tobacco-related cancers among adults 50-64 years old in the United States from 2001 to 2021 by residence type.
A. Metro. B. Non-metro.

Figure S11. Adjusted incidence rates of tobacco-related cancers among adults 65 years and older in the United States from 2001 to 2021 by residence type.
A. Metro. B. Non-metro.

Figure S12. Adjusted incidence rates of tobacco-related cancers among adults 20-49 years old in the United States from 2001 to 2021 by region of residence.
A. Northeast. B. Midwest. C. South. D. West.

Figure S13. Adjusted incidence rates of tobacco-related cancers among adults 50-64 years old in the United States from 2001 to 2021 by region of residence.
A. Northeast. B. Midwest. C. South. D. West.

Figure S14. Adjusted incidence rates of tobacco-related cancers among adults 65 years and older in the United States from 2001 to 2021 by region of residence.
A. Northeast. B. Midwest. C. South. D. West.

Figure S15. Mortality rates of tobacco-related cancers among adults 20-49 years old in the United States from 1975 to 2022 by sex.
A. All. B. Male. C. Female.

Figure S16. Mortality rates of tobacco-related cancers among adults 50-64 years old in the United States from 1975 to 2022 by sex.
A. All. B. Male. C. Female.

Figure S17. Mortality rates of tobacco-related cancers among adults 65+ years old in the United States from 1975 to 2022 by sex.
A. All. B. Male. C. Female.

Figure S18. Adjusted incidence rates of cancers of lung, oral cavity, pharynx, larynx, bladder, and esophagus among adults in the United States from 2001 to 2021 by age group.
A. All. B. 20-49 years old. C. 50-64 years old. D. 65+ years old.

Figure S19. Adjusted incidence rates of cancers of lung, oral cavity, pharynx, larynx, bladder, and esophagus among adults 20-49 years old in the United States from 2001 to 2021 by race/ethnicity.
A. Hispanic. B. Non-Hispanic White. C. Non-Hispanic Black. D. Non-Hispanic American Indi-an/Alaska Native. E. Non-Hispanic Asian or Pacific Islander.
NHW: Non-Hispanic White, NHB: Non-Hispanic Black, NHAIAN: Non-Hispanic American Indi-an/Alaska Native, NHAPI: Non-Hispanic Asian or Pacific Islander.

Figure S20. Adjusted incidence rates of cancers of lung, oral cavity, pharynx, larynx, bladder, and esophagus among adults 50-64 years old in the United States from 2001 to 2021 by race/ethnicity.
A. Hispanic. B. Non-Hispanic White. C. Non-Hispanic Black. D. Non-Hispanic American Indi-an/Alaska Native. E. Non-Hispanic Asian or Pacific Islander.

Figure S21. Adjusted incidence rates of cancers of lung, oral cavity, pharynx, larynx, bladder, and esophagus among adults 65 years and older in the United States from 2001 to 2021 by race/ethnicity.
A. Hispanic. B. Non-Hispanic White. C. Non-Hispanic Black. D. Non-Hispanic American Indi-an/Alaska Native. E. Non-Hispanic Asian or Pacific Islander.

Figure S22. Mortality rates of cancers of lung, oral cavity, pharynx, larynx, bladder, and esophagus among adults 20-49 years old in the United States from 1975 to 2022 by sex.
A. All. B. Male. C. Female.

Figure S23. Mortality rates of cancers of lung, oral cavity, pharynx, larynx, bladder, and esophagus among adults 50-64 years old in the United States from 1975 to 2022 by sex.
A. All. B. Male. C. Female.

Figure S24. Mortality rates of cancers of lung, oral cavity, pharynx, larynx, bladder, and esophagus among adults 65+ years old in the United States from 1975 to 2022 by sex.
A. All. B. Male. C. Female.

Figure S25. Prevalence of current tobacco smokers among adults in the United States from 1983 to 2021 by age, sex, region of residence, and race/ethnicity.
A. Age. B. Sex. C. Region of residence. D. Race/ethnicity.
NHW: Non-Hispanic White, NHB: Non-Hispanic Black.

Figure S26. Prevalence of current e-cigarette smokers among adults in the United States from 2016 to 2021 by age, sex, region of residence, and race/ethnicity.
A. Age. B. Sex. C. Region of residence. D. Race/ethnicity.
NHW: Non-Hispanic White, NHB: Non-Hispanic Black, NHAIAN: Non-Hispanic American Indian/Alaska Native, NHAPI: Non-Hispanic Asian or Pacific Islander.

Figure S27. Health insurance coverage among adults in the United States from 2000 to 2021 by age, sex, region of residence, and race/ethnicity.
A. Age. B. Sex. C. Region of residence. D. Race/ethnicity.
NHW: Non-Hispanic White, NHB: Non-Hispanic Black.

Figure S1. (A)

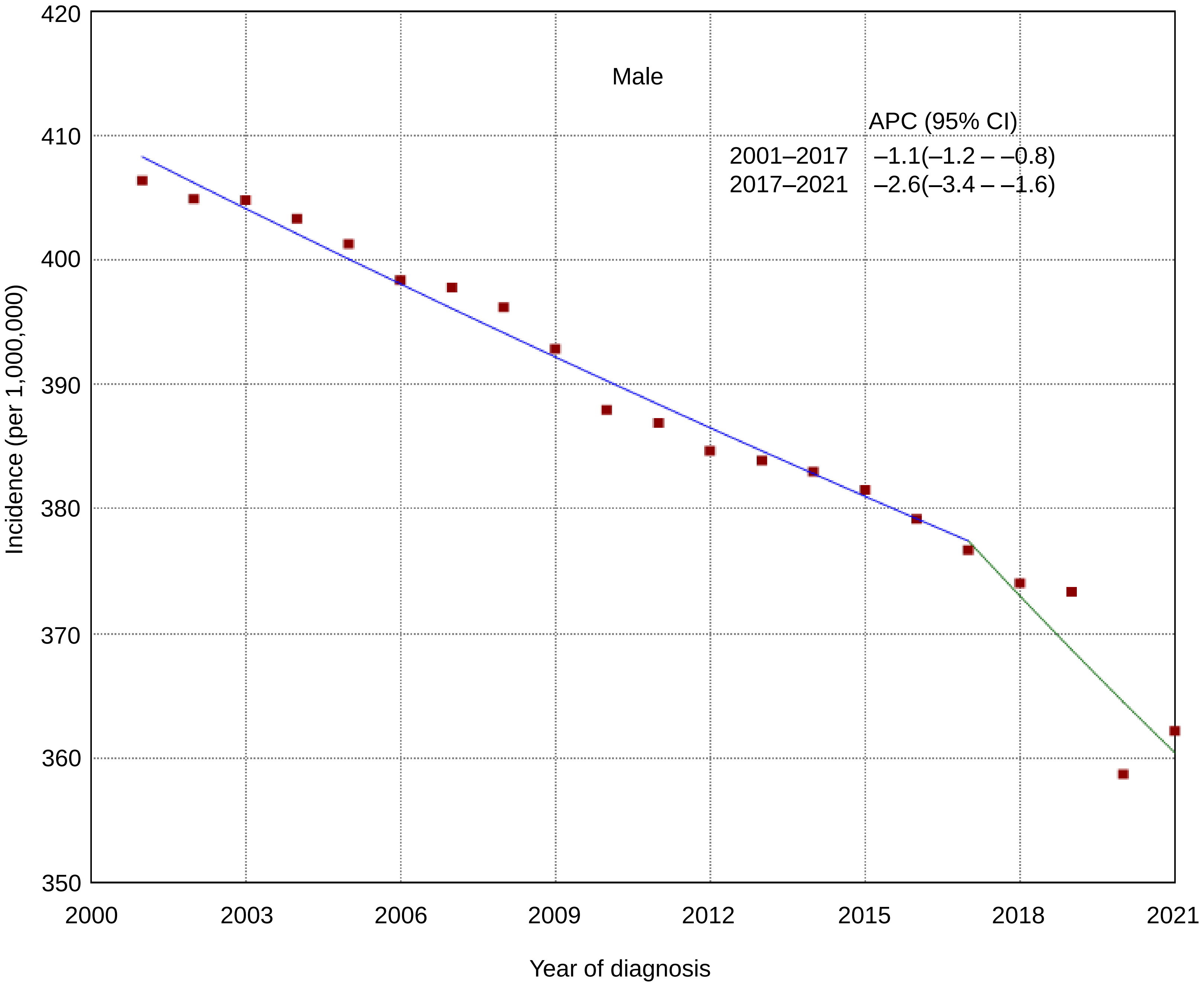

Male
APC (95% CI)
2001–2017 –1.1(–1.2 – –0.8)
2017–2021 –2.6(–3.4 – –1.6)
Incidence (per 1,000,000)
420
410
400
390
380
370
360
350
2000
2003
2006
2009
2012
2015
2018
2021
Year of diagnosis

Figure S1. (B)

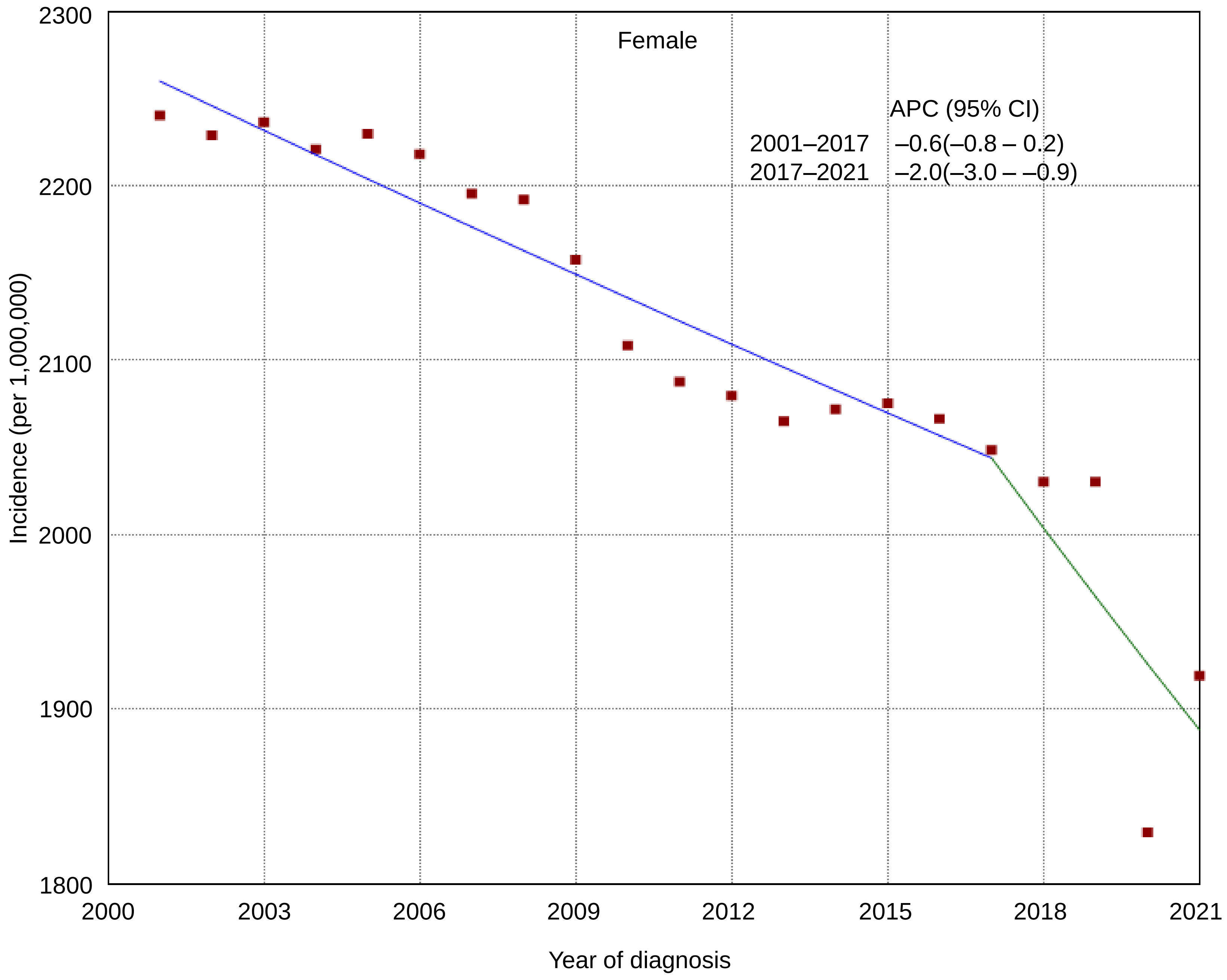

2300
2200
2100
2000
1900
1800
2000
2003
2006
2009
2012
2015
2018
2021
Female
APC (95% CI)
2001–2017 –0.6(–0.8 – 0.2)
2017–2021 –2.0(–3.0 – –0.9)
Incidence (per 1,000,000)
Year of diagnosis

Figure S2. (A)

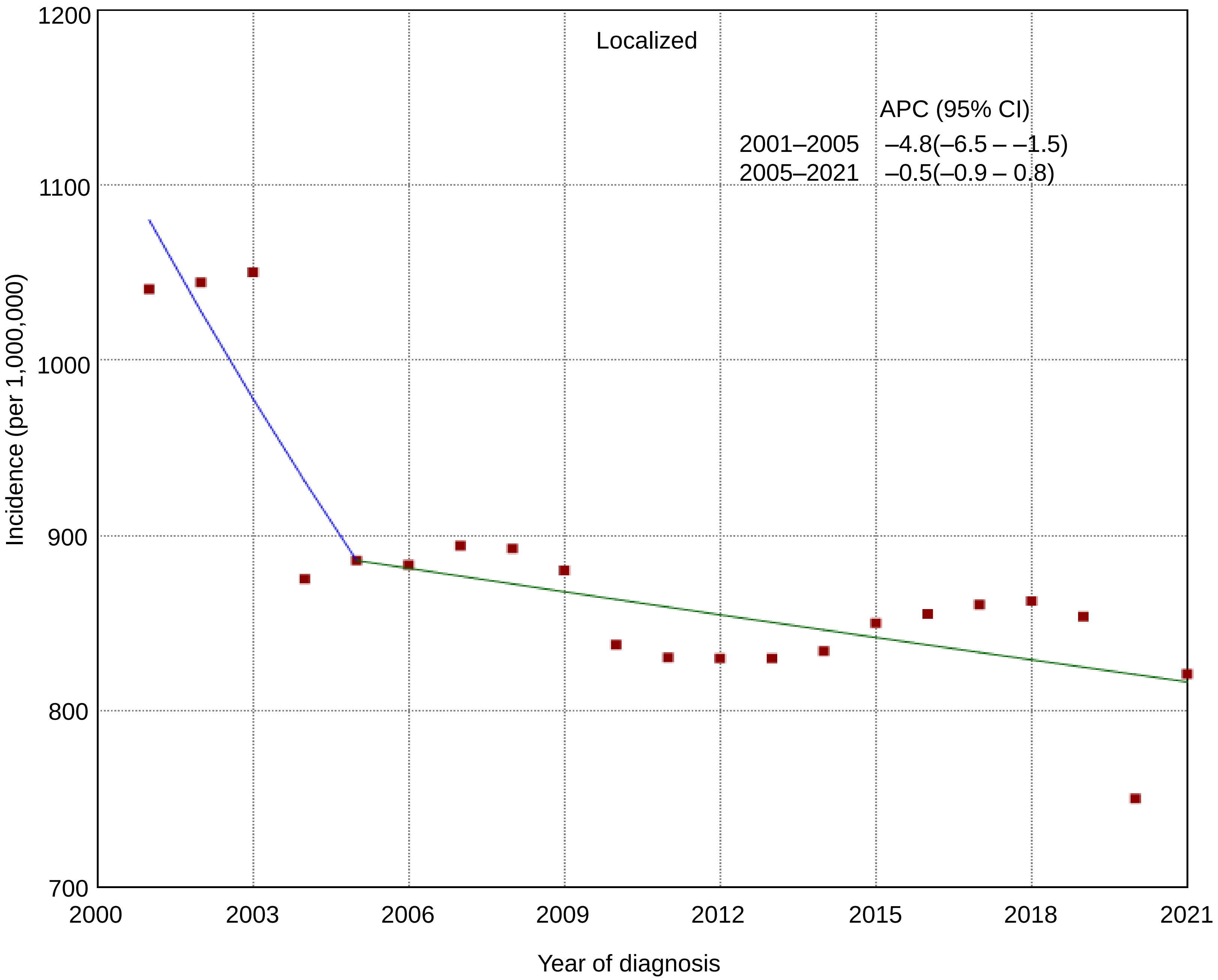

Localized
APC (95% CI)
2001–2005 –4.8(–6.5 – –1.5)
2005–2021 –0.5(–0.9 – 0.8)
Incidence (per 1,000,000)
1200
1100
1000
900
800
700
2000
2003
2006
2009
2012
2015
2018
2021
Year of diagnosis

Figure S2. (B)

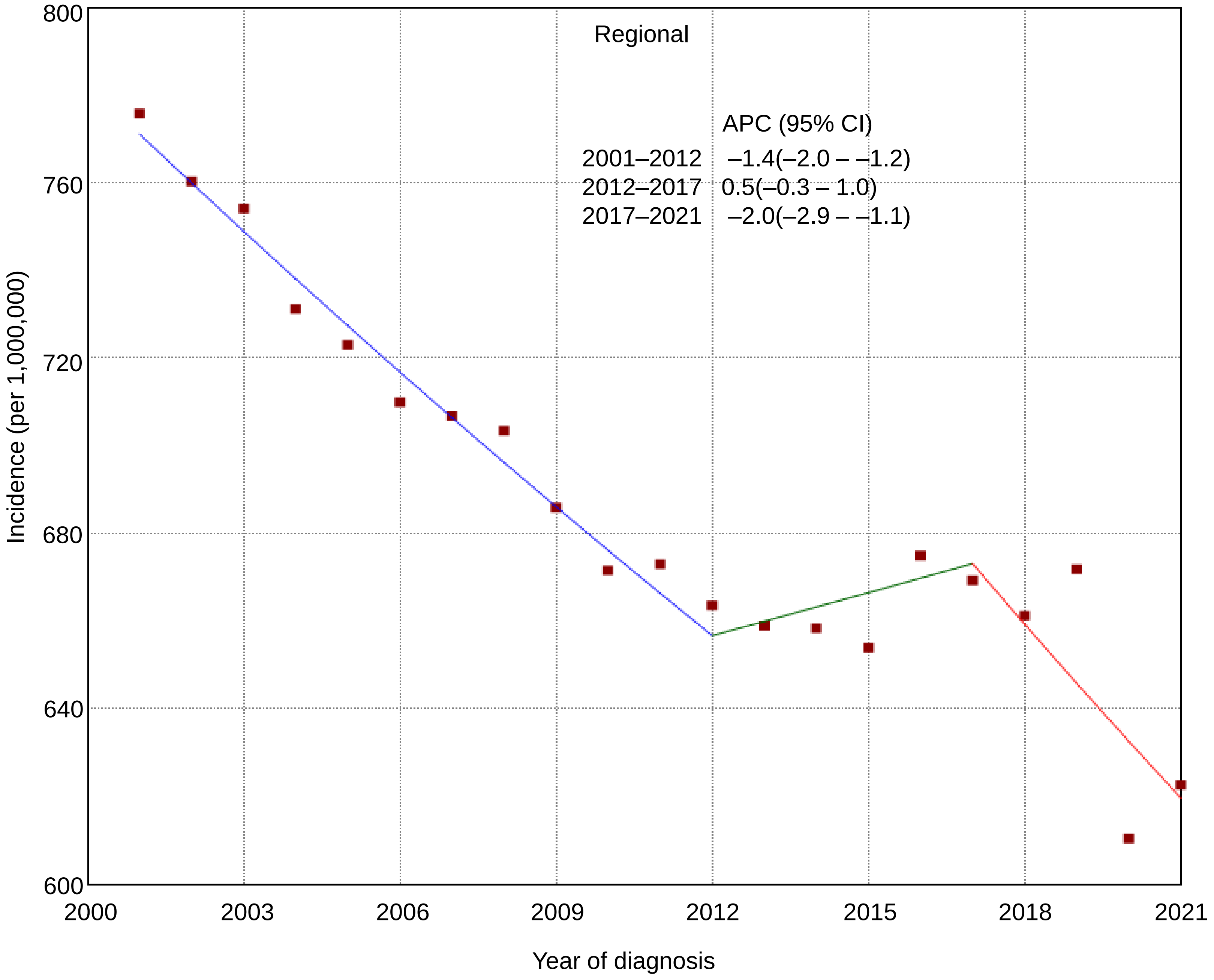

Regional
APC (95% CI)
2001–2012 –1.4(–2.0 – –1.2)
2012–2017 0.5(–0.3 – 1.0)
2017–2021 –2.0(–2.9 – –1.1)
800
760
720
680
640
600
Incidence (per 1,000,000)
2000
2003
2006
2009
2012
2015
2018
2021
Year of diagnosis

Figure S2. (C)

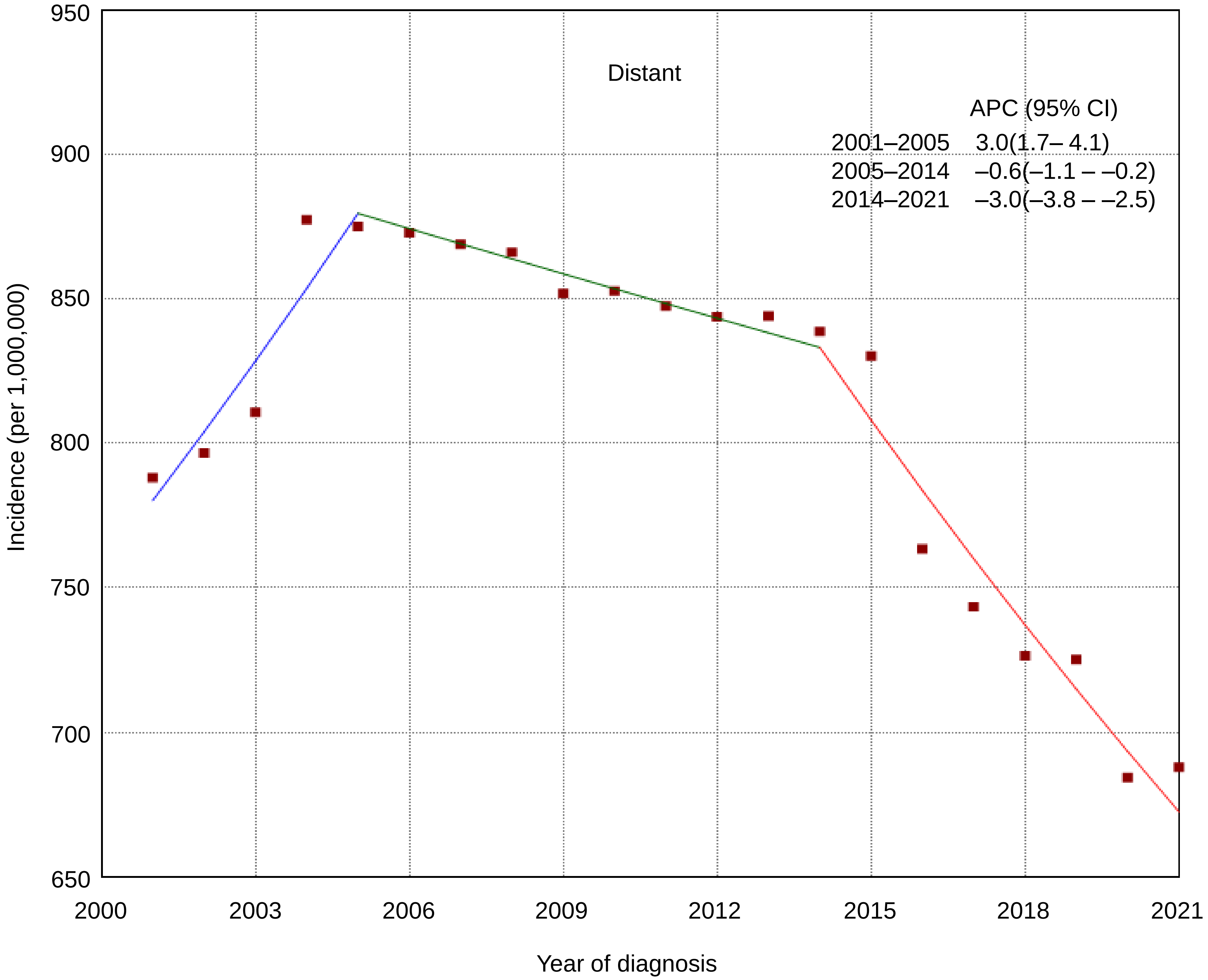

Distant
APC (95% CI)
2001–2005 3.0(1.7– 4.1)
2005–2014 –0.6(–1.1 – –0.2)
2014–2021 –3.0(–3.8 – –2.5)
Incidence (per 1,000,000)
950
900
850
800
750
700
650
2000
2003
2006
2009
2012
2015
2018
2021
Year of diagnosis

Figure S3. (A)

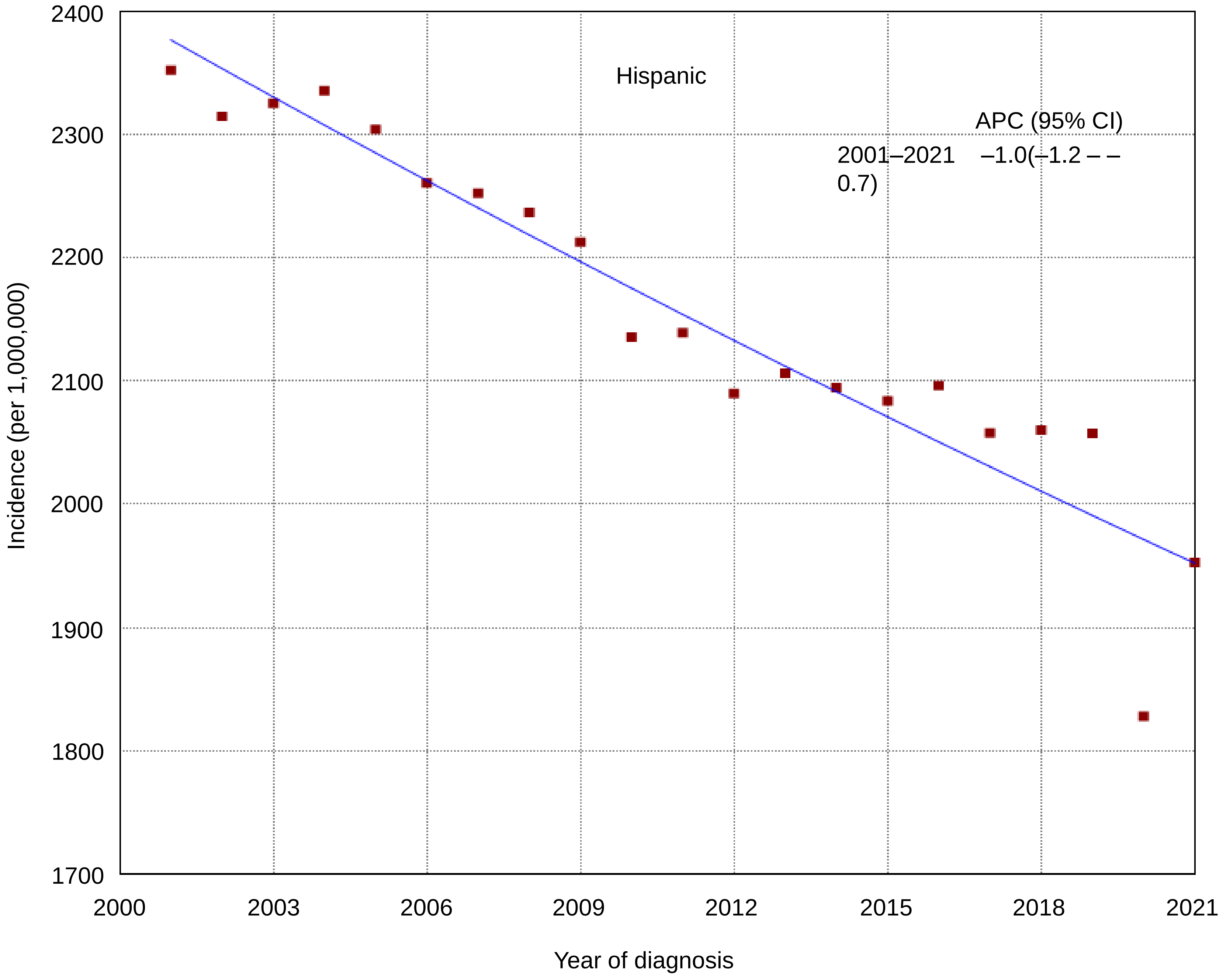
Hispanic
APC (95% CI)
2001–2021 –1.0(–1.2 – –0.7)
Incidence (per 1,000,000)
2400
2300
2200
2100
2000
1900
1800
1700
2000
2003
2006
2009
2012
2015
2018
2021
Year of diagnosis

Figure S3. (B)

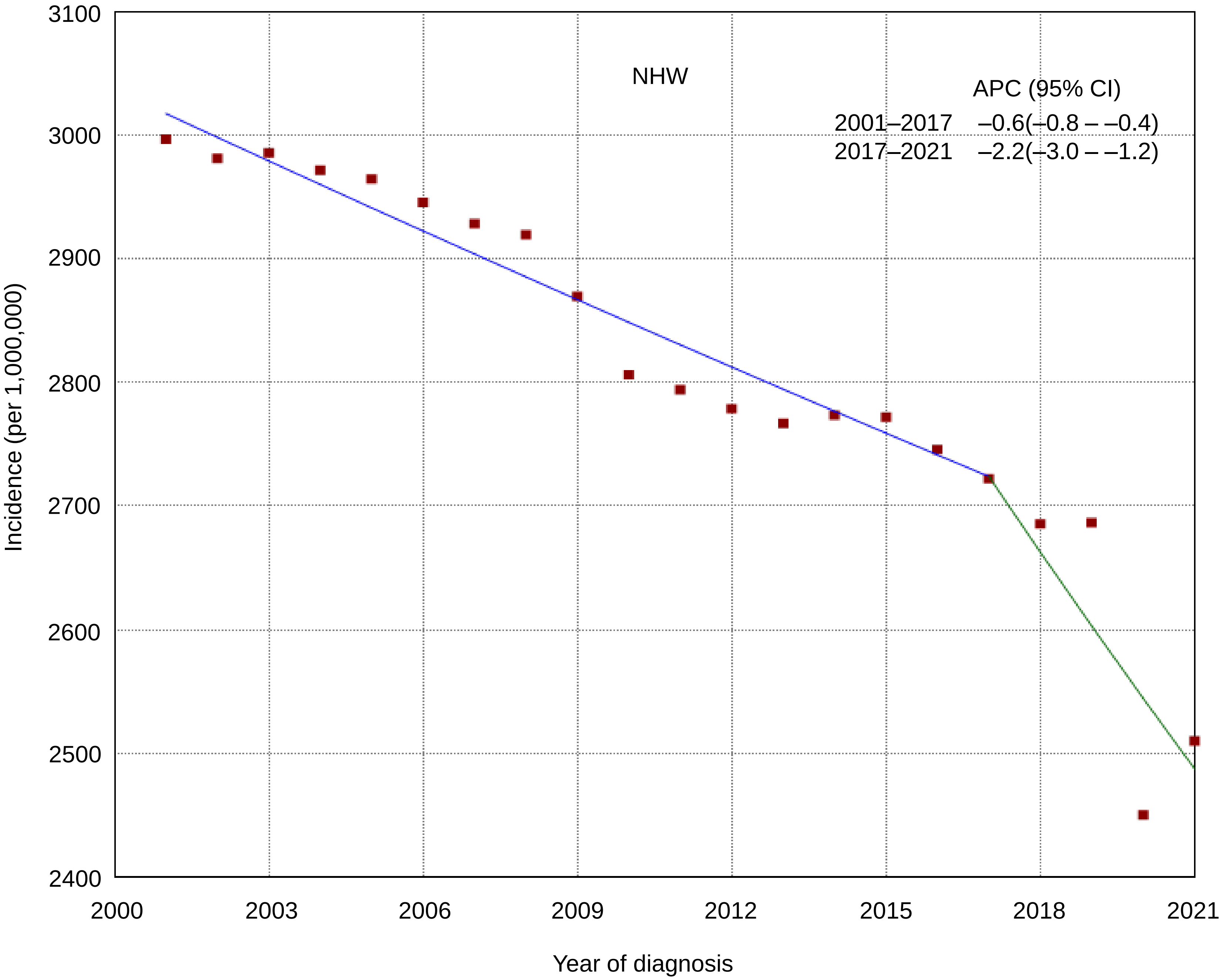

NHW
APC (95% CI)
2001–2017 –0.6(–0.8 – –0.4)
2017–2021 –2.2(–3.0 – –1.2)
Incidence (per 1,000,000)
3100
3000
2900
2800
2700
2600
2500
2400
2000
2003
2006
2009
2012
2015
2018
2021
Year of diagnosis

Figure S3. (C)

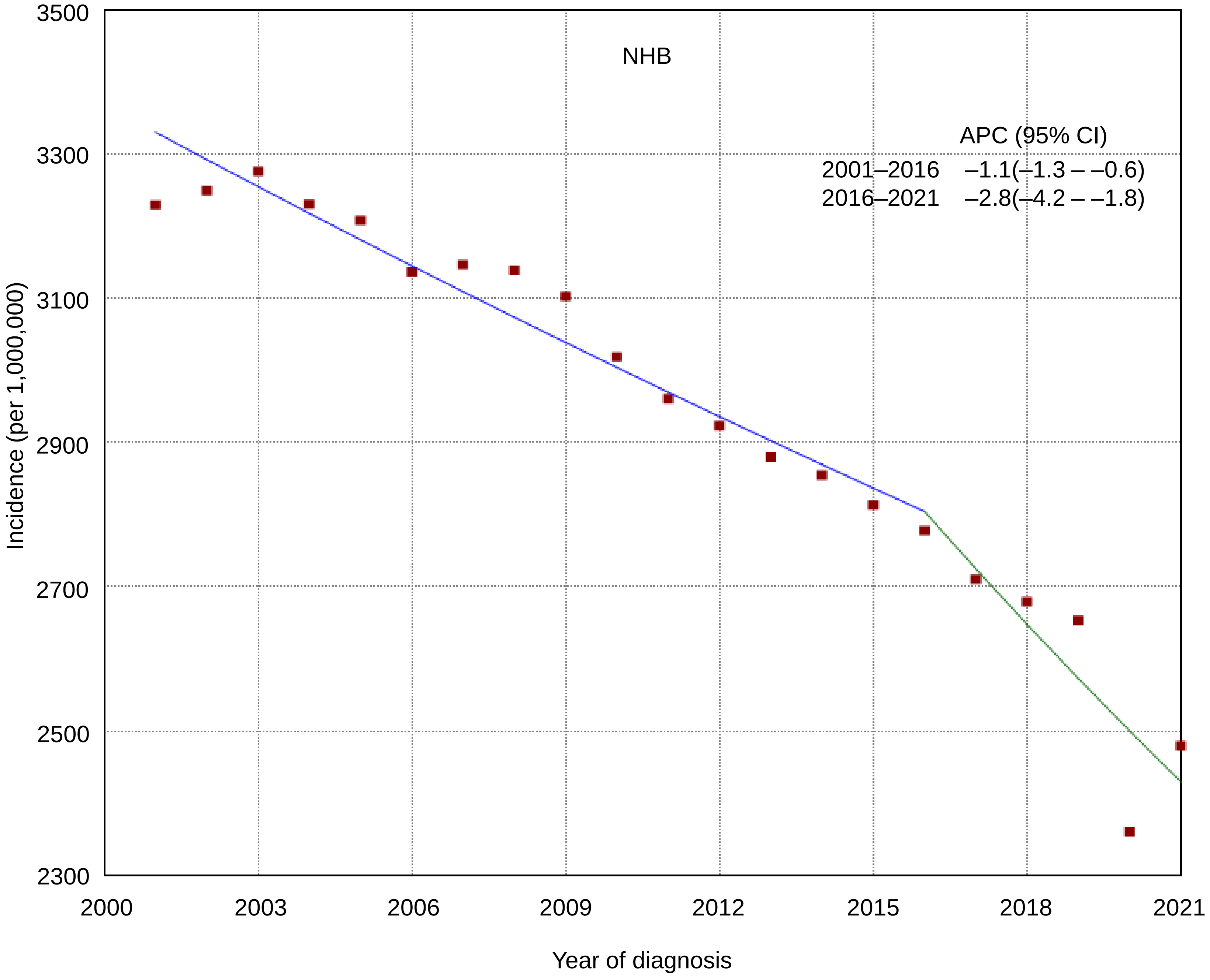

NHB
APC (95% CI)
2001–2016 –1.1(–1.3 – –0.6)
2016–2021 –2.8(–4.2 – –1.8)
Incidence (per 1,000,000)
3500
3300
3100
2900
2700
2500
2300
2000
2003
2006
2009
2012
2015
2018
2021
Year of diagnosis

Figure S3. (D)

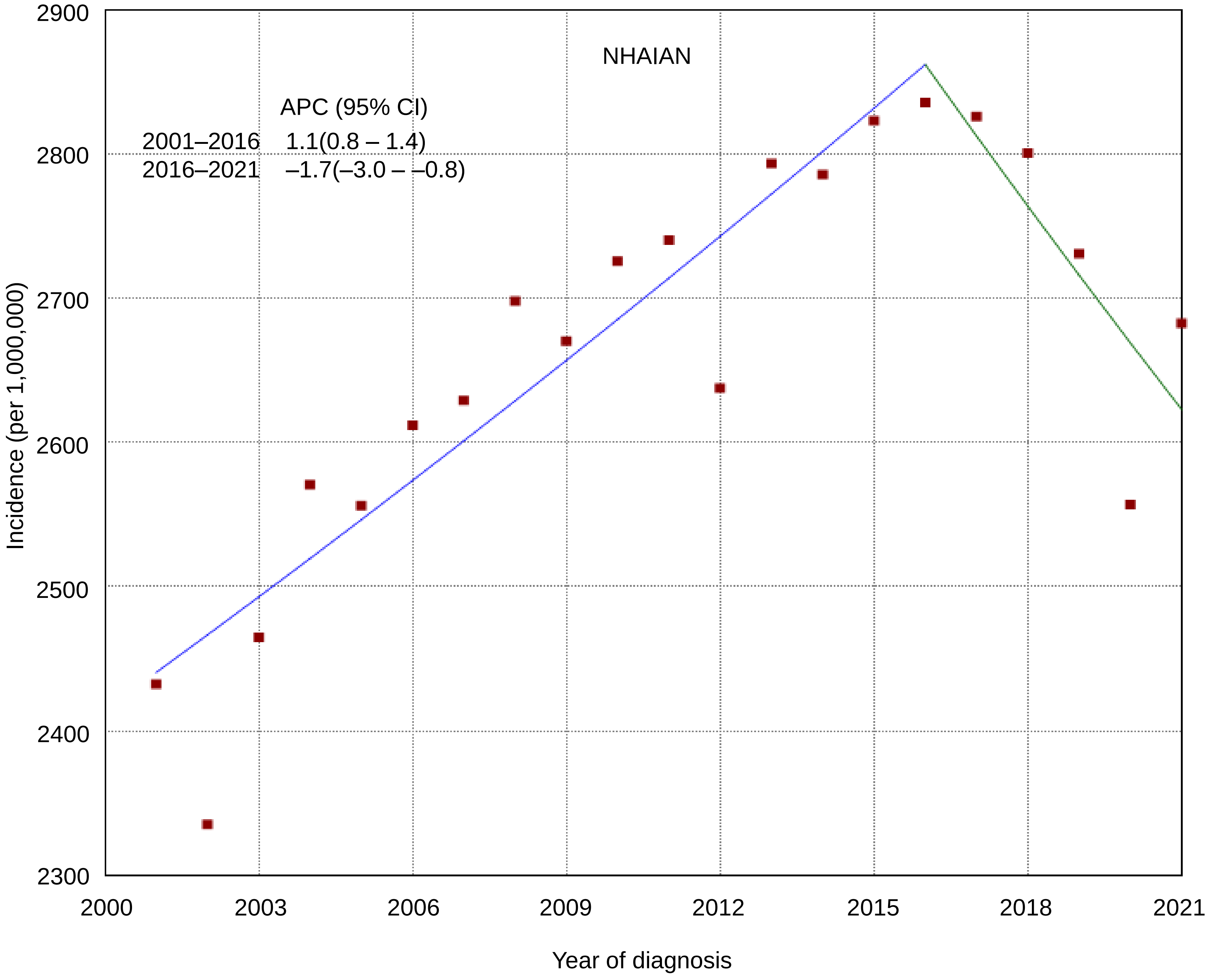
NHAIAN
APC (95% CI)
2001–2016 1.1(0.8 – 1.4)
2016–2021 –1.7(–3.0 – –0.8)
Incidence (per 1,000,000)
2900
2800
2700
2600
2500
2400
2300
2000
2003
2006
2009
2012
2015
2018
2021
Year of diagnosis

Figure S3. (E)

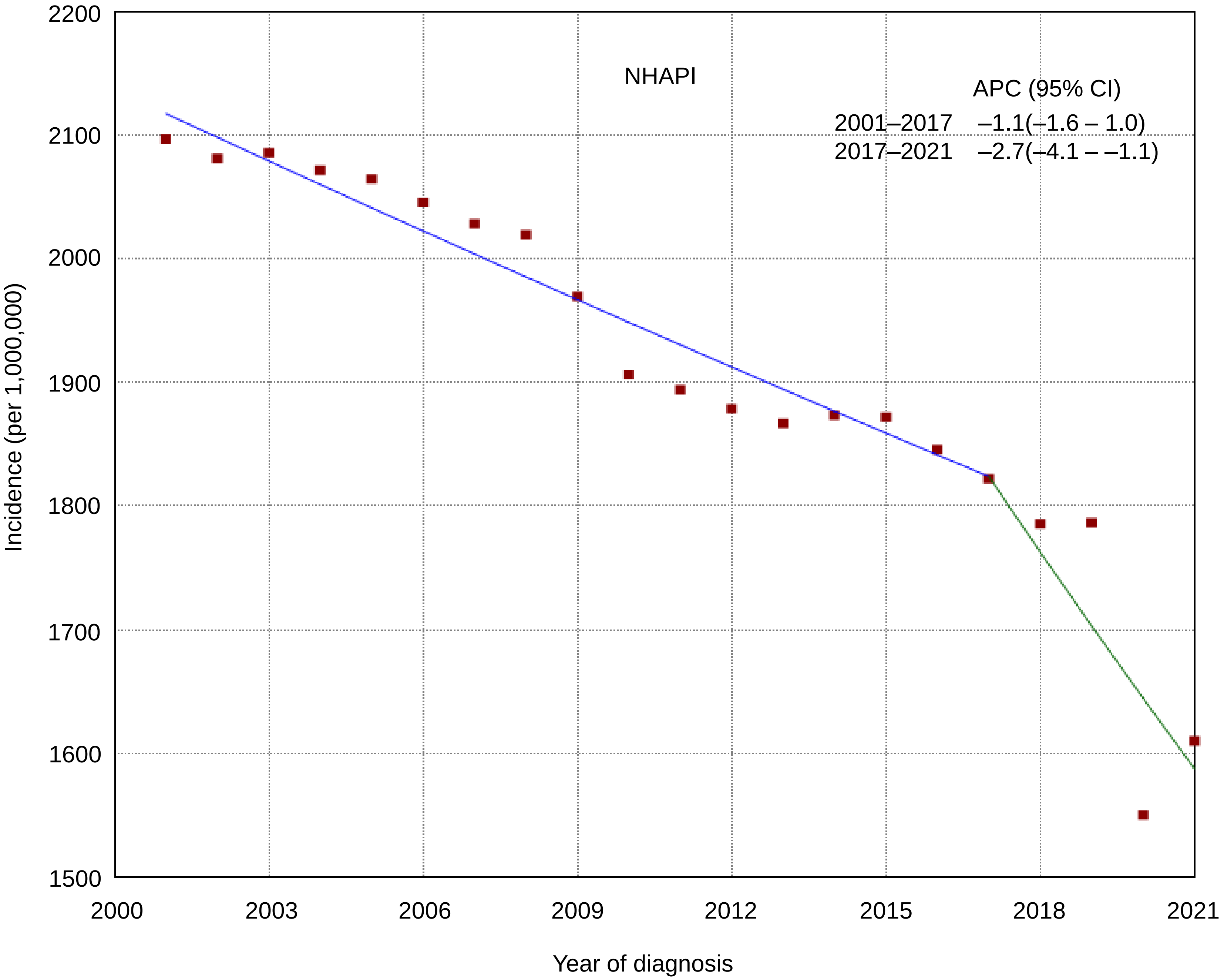
NHAPI
APC (95% CI)
2001–2017 −1.1(−1.6 – 1.0)
2017–2021 −2.7(−4.1 – −1.1)
Incidence (per 1,000,000)
2200
2100
2000
1900
1800
1700
1600
1500
2000
2003
2006
2009
2012
2015
2018
2021
Year of diagnosis

Figure S4. (A)

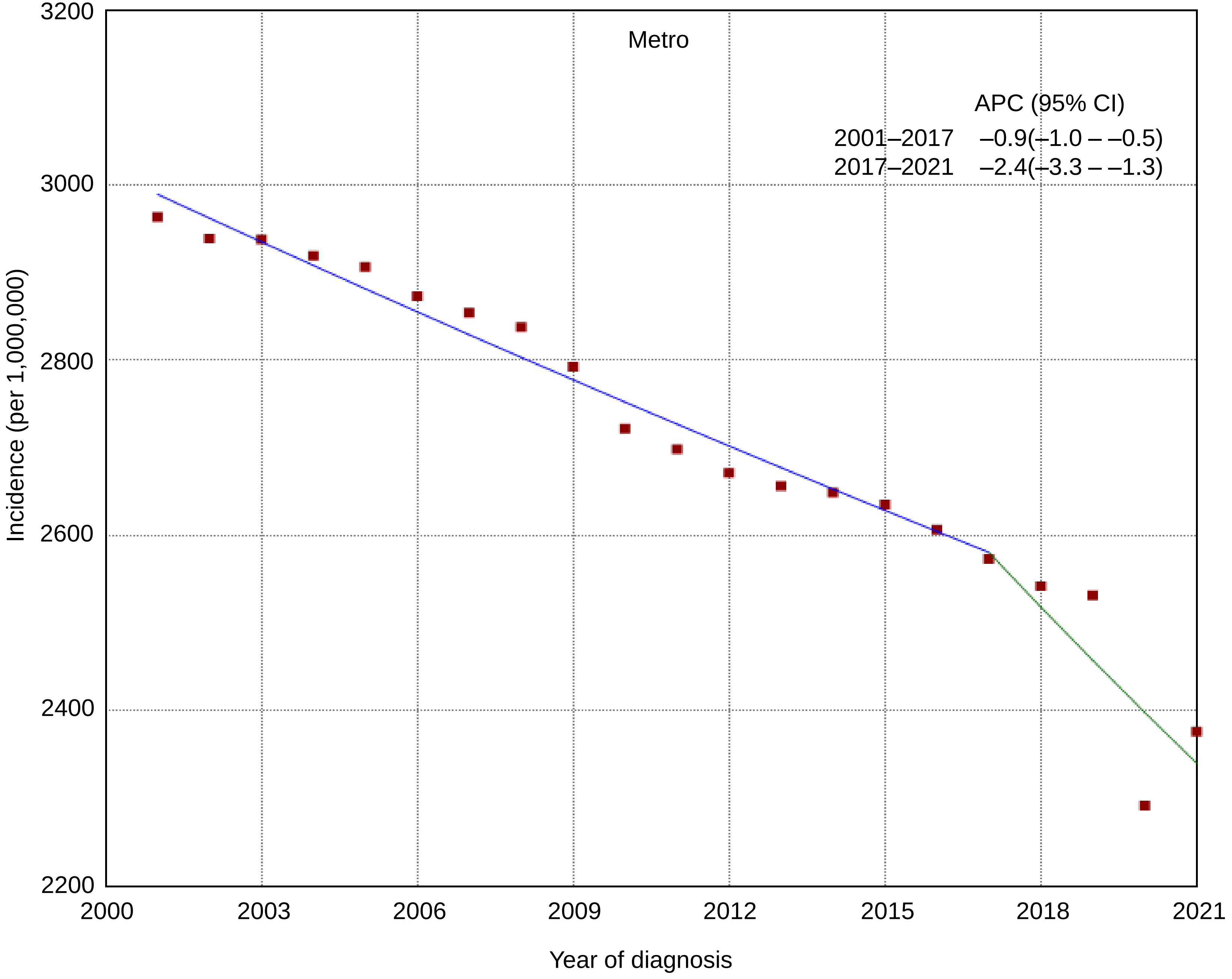

Metro
APC (95% CI)
2001–2017 –0.9(–1.0 – –0.5)
2017–2021 –2.4(–3.3 – –1.3)
3200
3000
2800
2600
2400
2200
Incidence (per 1,000,000)
2000
2003
2006
2009
2012
2015
2018
2021
Year of diagnosis

Figure S4. (B)

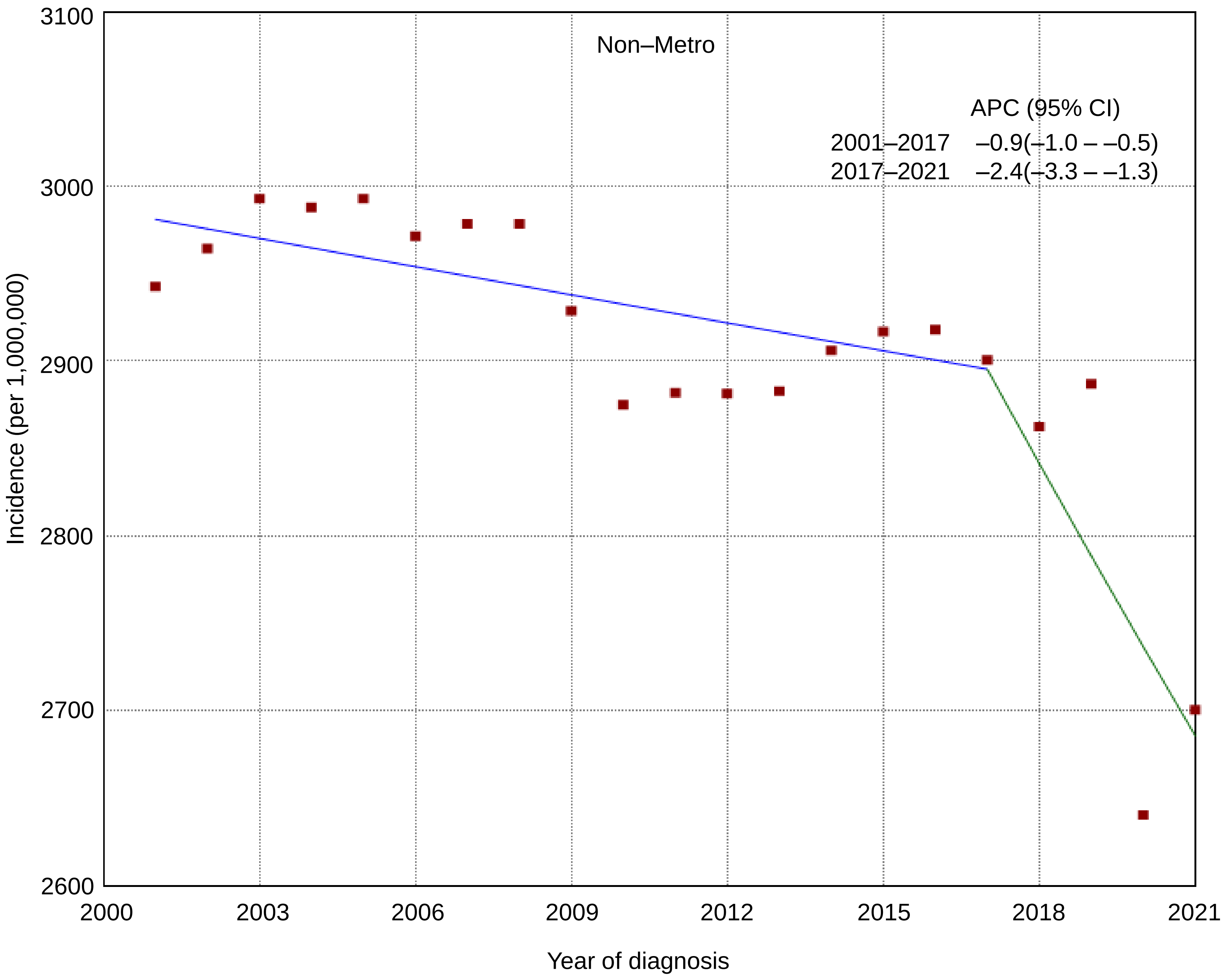

Non–Metro
APC (95% CI)
2001–2017 –0.9(–1.0 – –0.5)
2017–2021 –2.4(–3.3 – –1.3)
3100
3000
2900
2800
2700
2600
Incidence (per 1,000,000)
2000
2003
2006
2009
2012
2015
2018
2021
Year of diagnosis

Figure S5. (A)

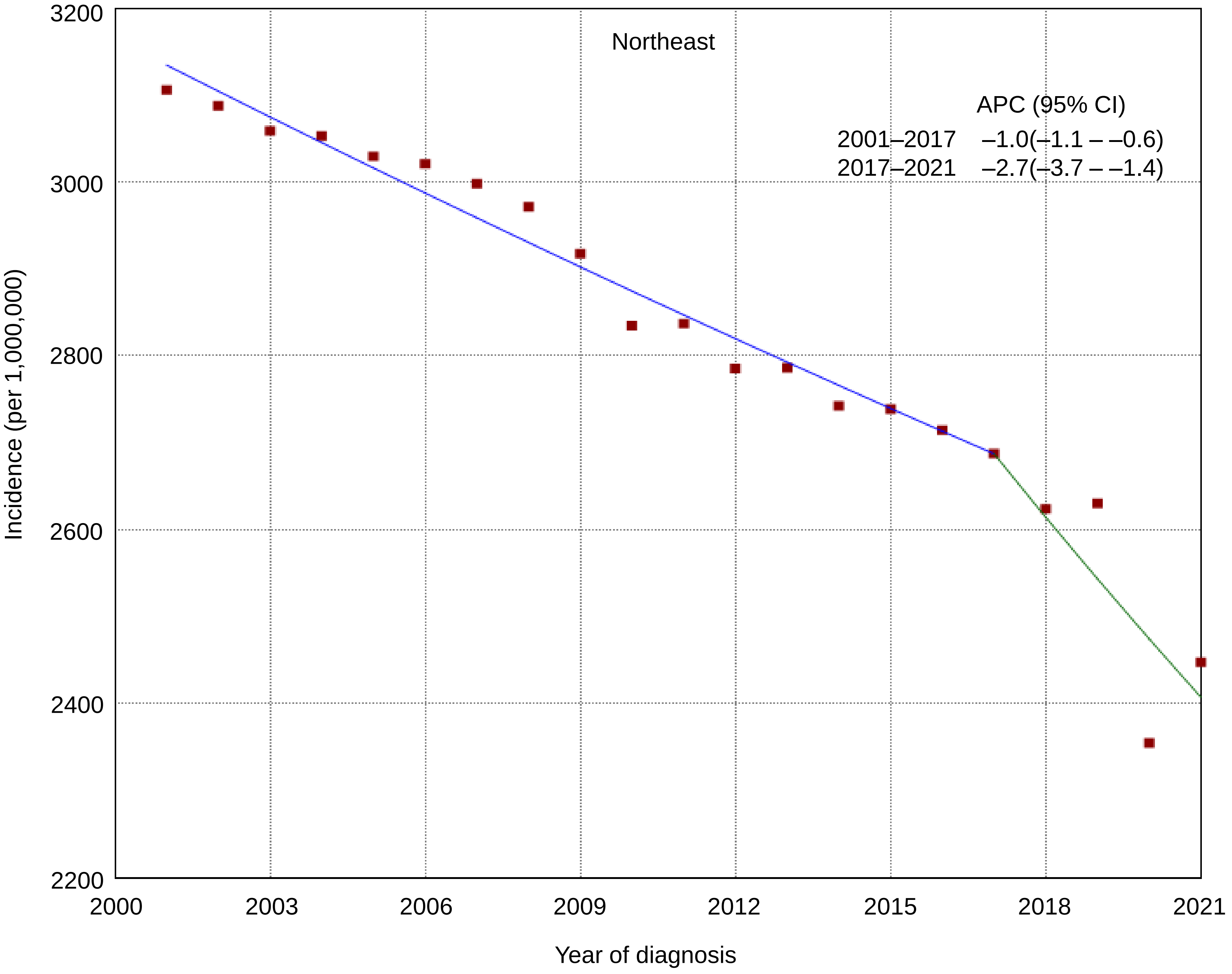

Northeast
APC (95% CI)
2001–2017 −1.0(−1.1 – −0.6)
2017–2021 −2.7(−3.7 – −1.4)
Incidence (per 1,000,000)
3200
3000
2800
2600
2400
2200
2000
2003
2006
2009
2012
2015
2018
2021
Year of diagnosis

Figure S5. (B)

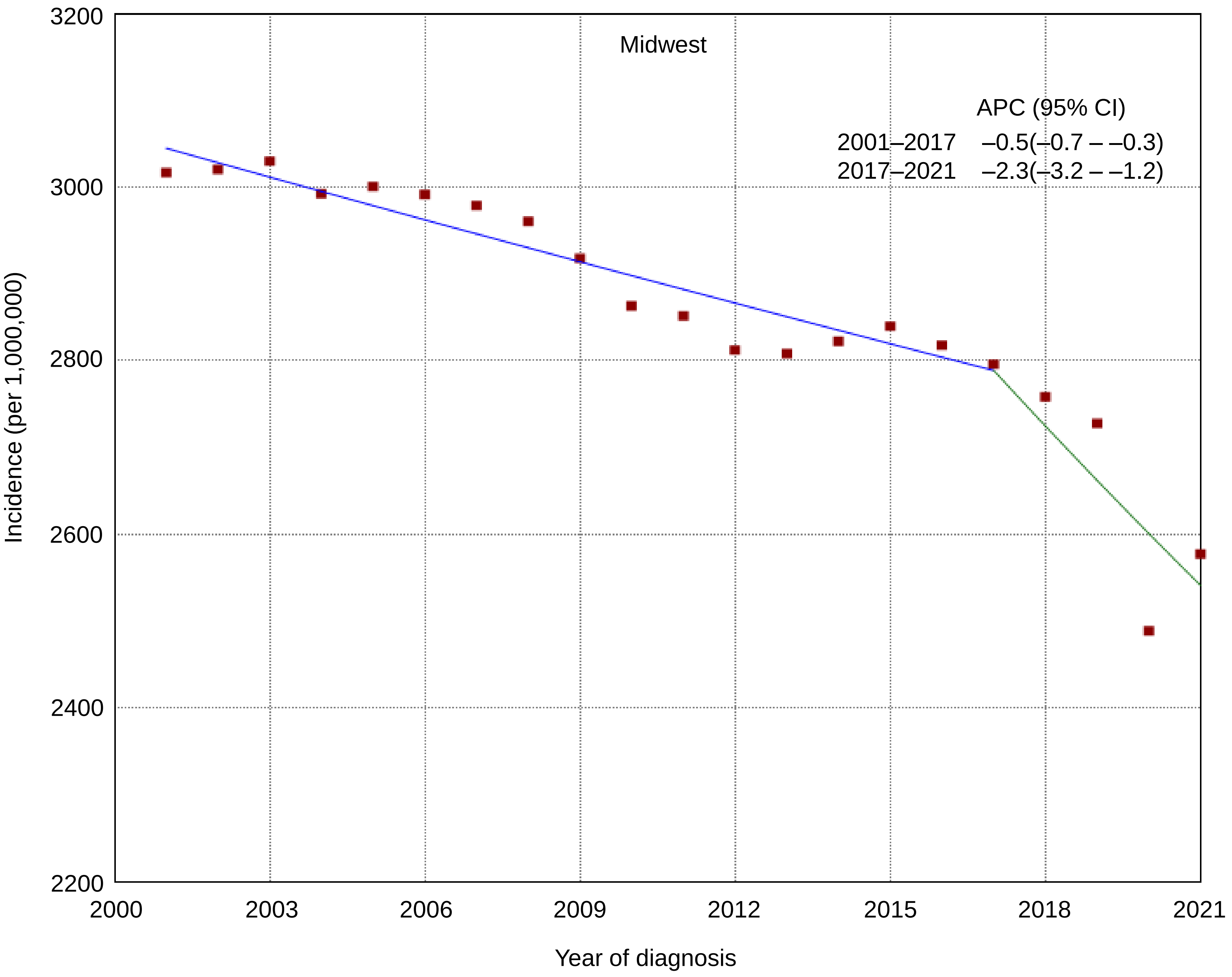

Midwest
APC (95% CI)
2001–2017 –0.5(–0.7 – –0.3)
2017–2021 –2.3(–3.2 – –1.2)
3200
3000
2800
2600
2400
2200
Incidence (per 1,000,000)
2000
2003
2006
2009
2012
2015
2018
2021
Year of diagnosis

Figure S5. (C)

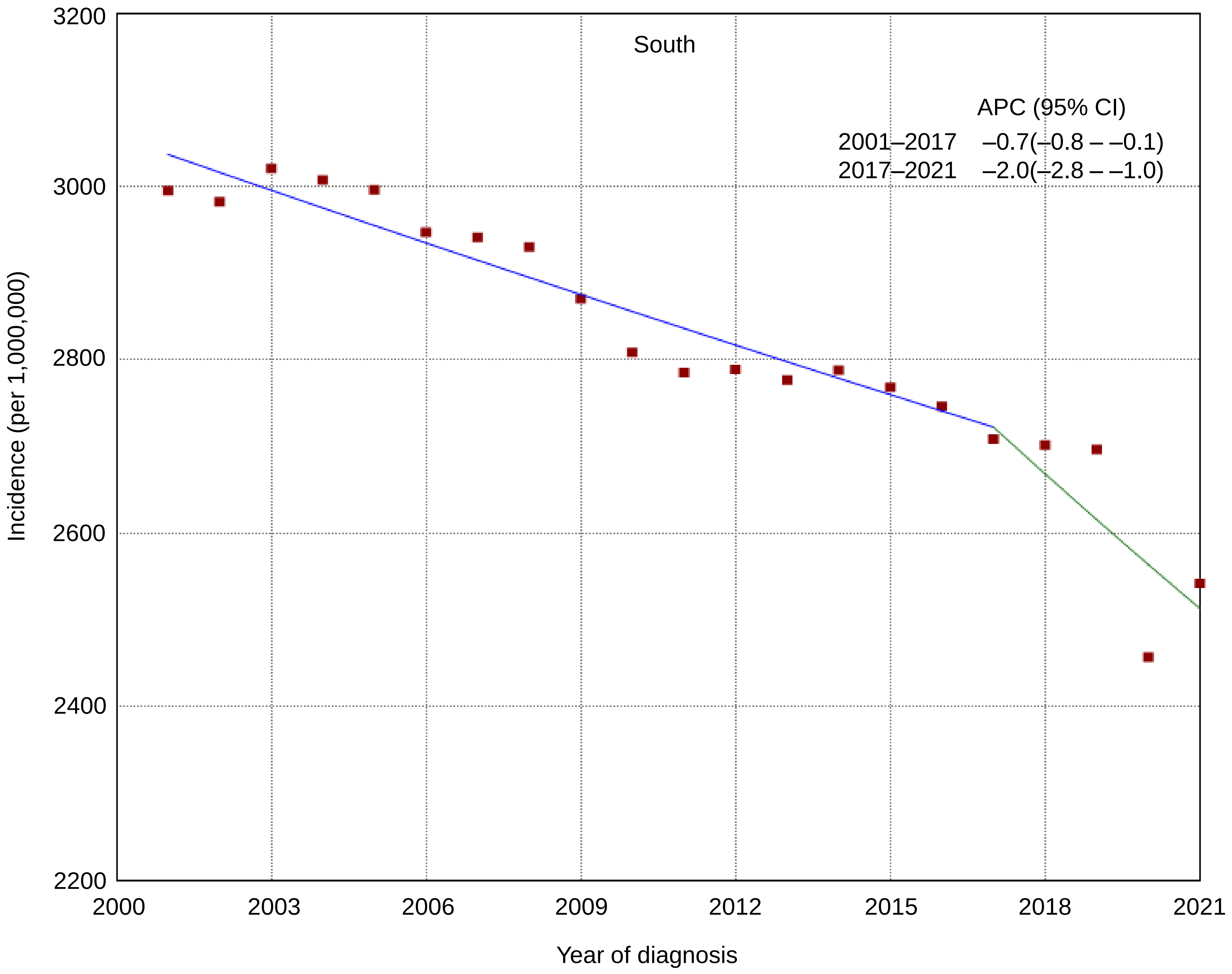
South
APC (95% CI)
2001–2017 –0.7(–0.8 – –0.1)
2017–2021 –2.0(–2.8 – –1.0)
3200
3000
2800
2600
2400
2200
Incidence (per 1,000,000)
2000
2003
2006
2009
2012
2015
2018
2021
Year of diagnosis

Figure S5. (D)

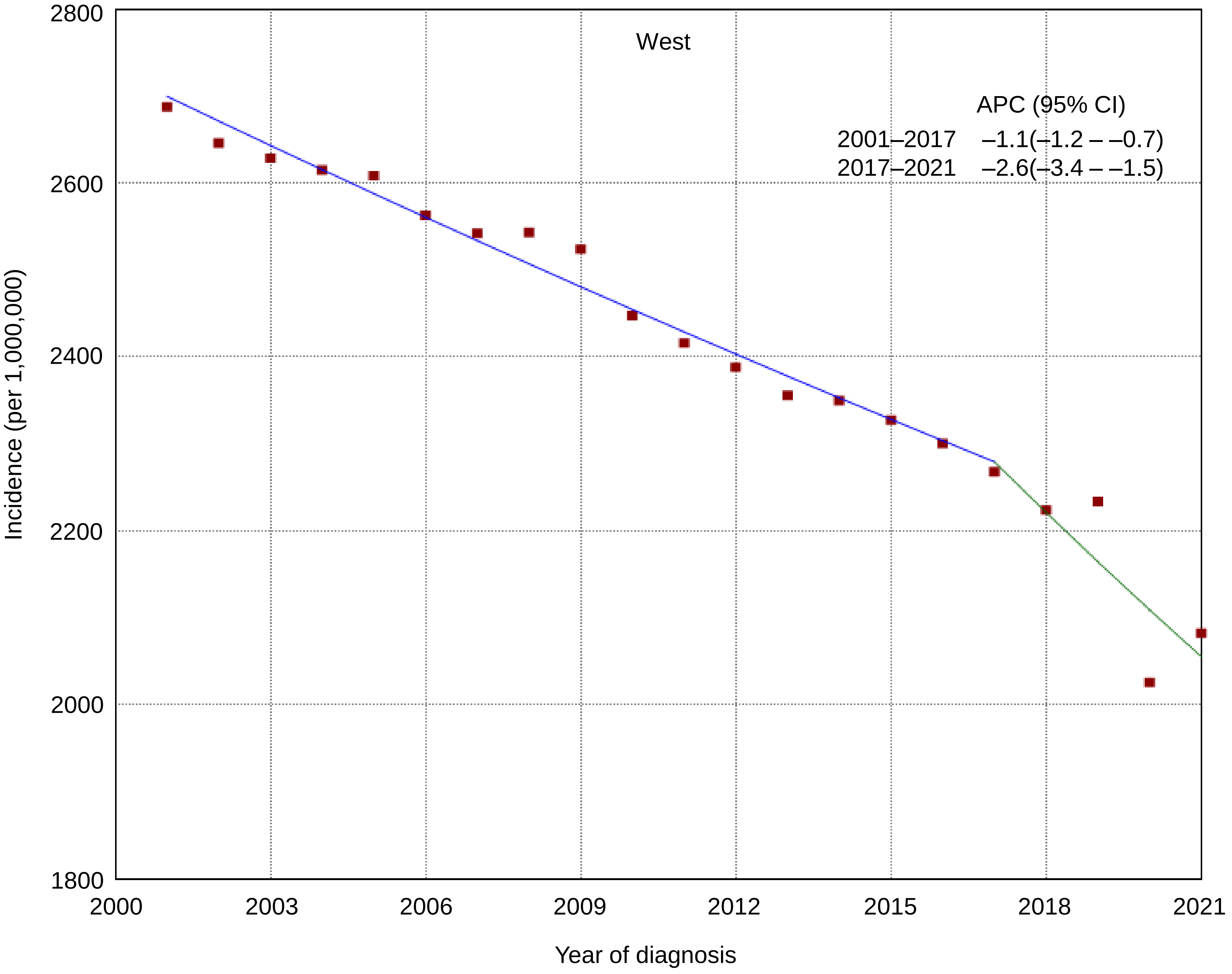
West
APC (95% CI)
2001–2017 –1.1(–1.2 – –0.7)
2017–2021 –2.6(–3.4 – –1.5)
Incidence (per 1,000,000)
2800
2600
2400
2200
2000
1800
2000
2003
2006
2009
2012
2015
2018
2021
Year of diagnosis

Figure S6. (A)

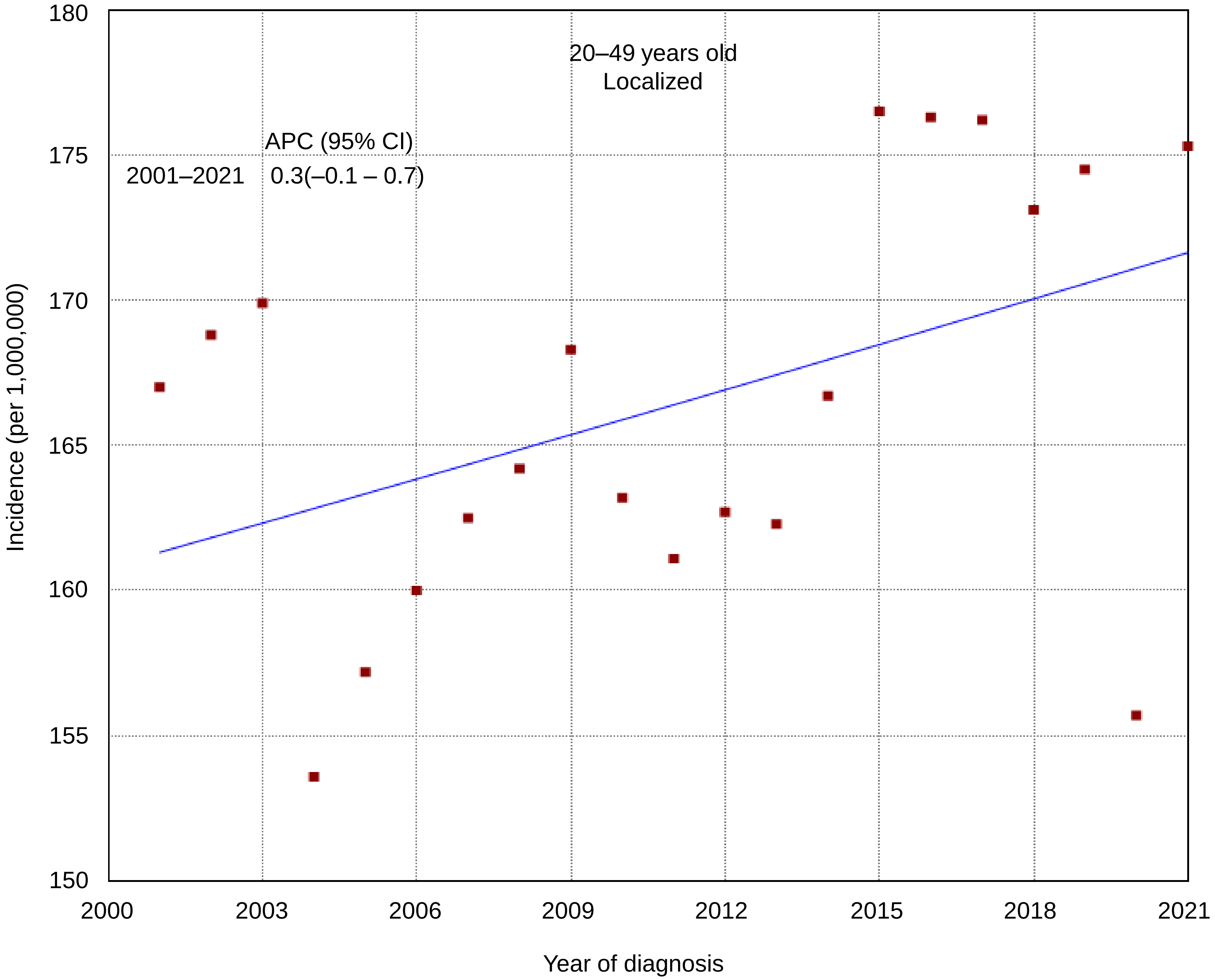

20–49 years old
Localized
APC (95% CI)
2001–2021 0.3(–0.1 – 0.7)
Incidence (per 1,000,000)
180
175
170
165
160
155
150
2000
2003
2006
2009
2012
2015
2018
2021
Year of diagnosis

Figure S6. (B)

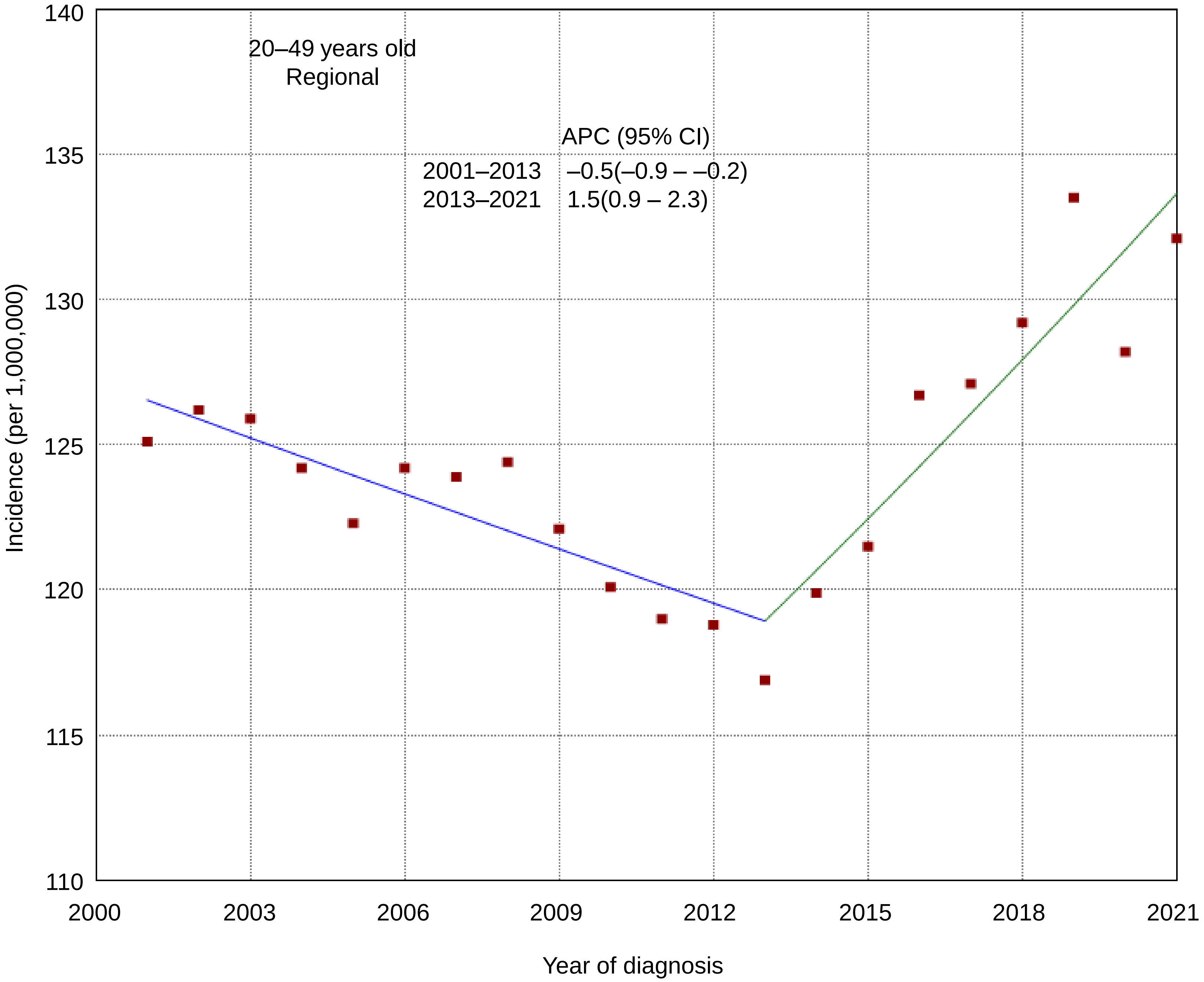
20–49 years old
Regional
APC (95% CI)
2001–2013 –0.5(–0.9 – –0.2)
2013–2021 1.5(0.9 – 2.3)
Incidence (per 1,000,000)
140
135
130
125
120
115
110
2000
2003
2006
2009
2012
2015
2018
2021
Year of diagnosis

Figure S6. (C)

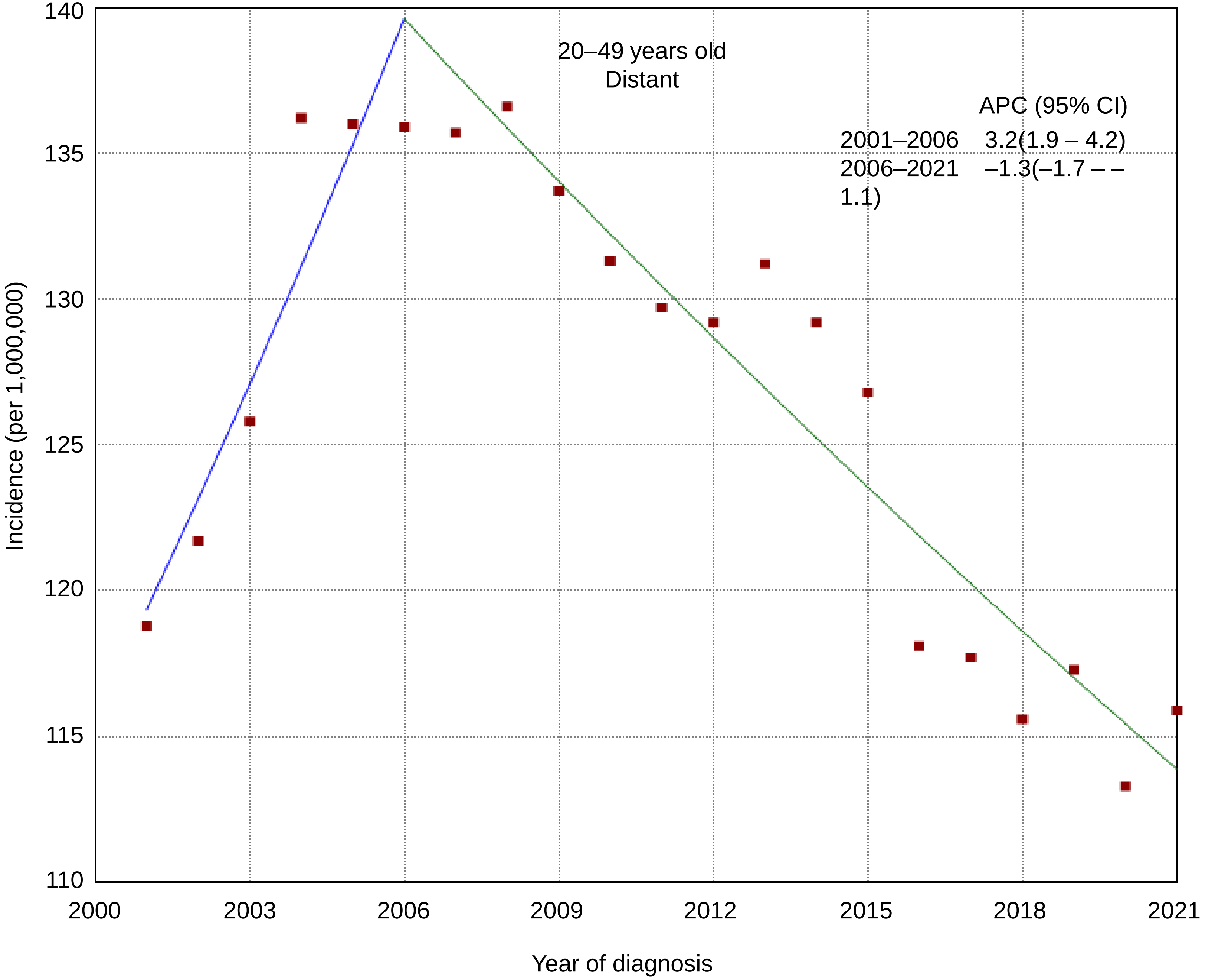
20–49 years old
Distant
APC (95% CI)
2001–2006 3.2(1.9 – 4.2)
2006–2021 –1.3(–1.7 – –1.1)
Incidence (per 1,000,000)
Year of diagnosis
140
135
130
125
120
115
110
2000
2003
2006
2009
2012
2015
2018
2021

Figure S7. (A)

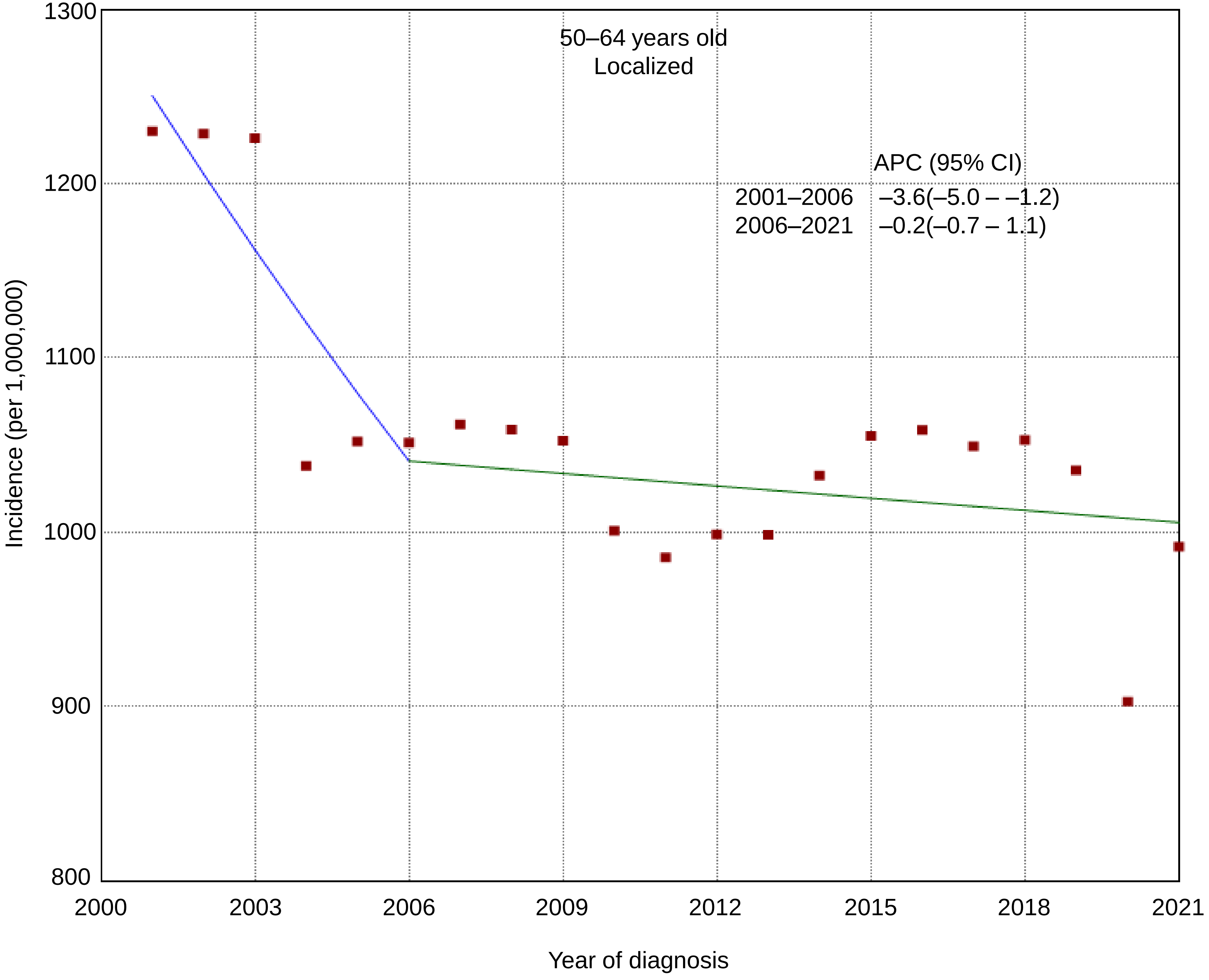

50–64 years old
Localized
APC (95% CI)
2001–2006 –3.6(–5.0 – –1.2)
2006–2021 –0.2(–0.7 – 1.1)
1300
1200
1100
1000
900
800
Incidence (per 1,000,000)
2000
2003
2006
2009
2012
2015
2018
2021
Year of diagnosis

Figure S7. (B)

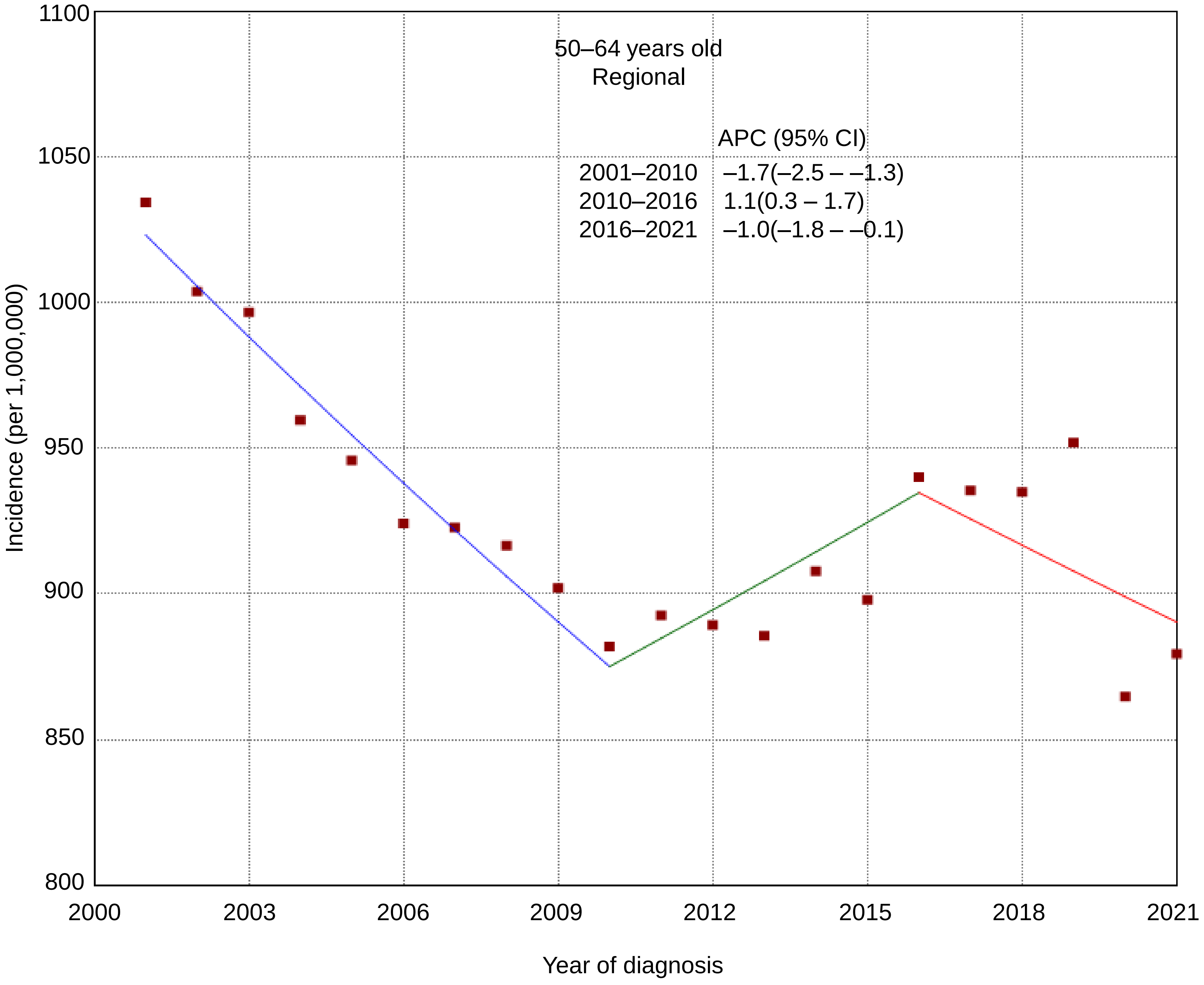

50–64 years old
Regional
APC (95% CI)
2001–2010 –1.7(–2.5 – –1.3)
2010–2016 1.1(0.3 – 1.7)
2016–2021 –1.0(–1.8 – –0.1)
1100
1050
1000
950
900
850
800
Incidence (per 1,000,000)
2000
2003
2006
2009
2012
2015
2018
2021
Year of diagnosis

Figure S7. (C)

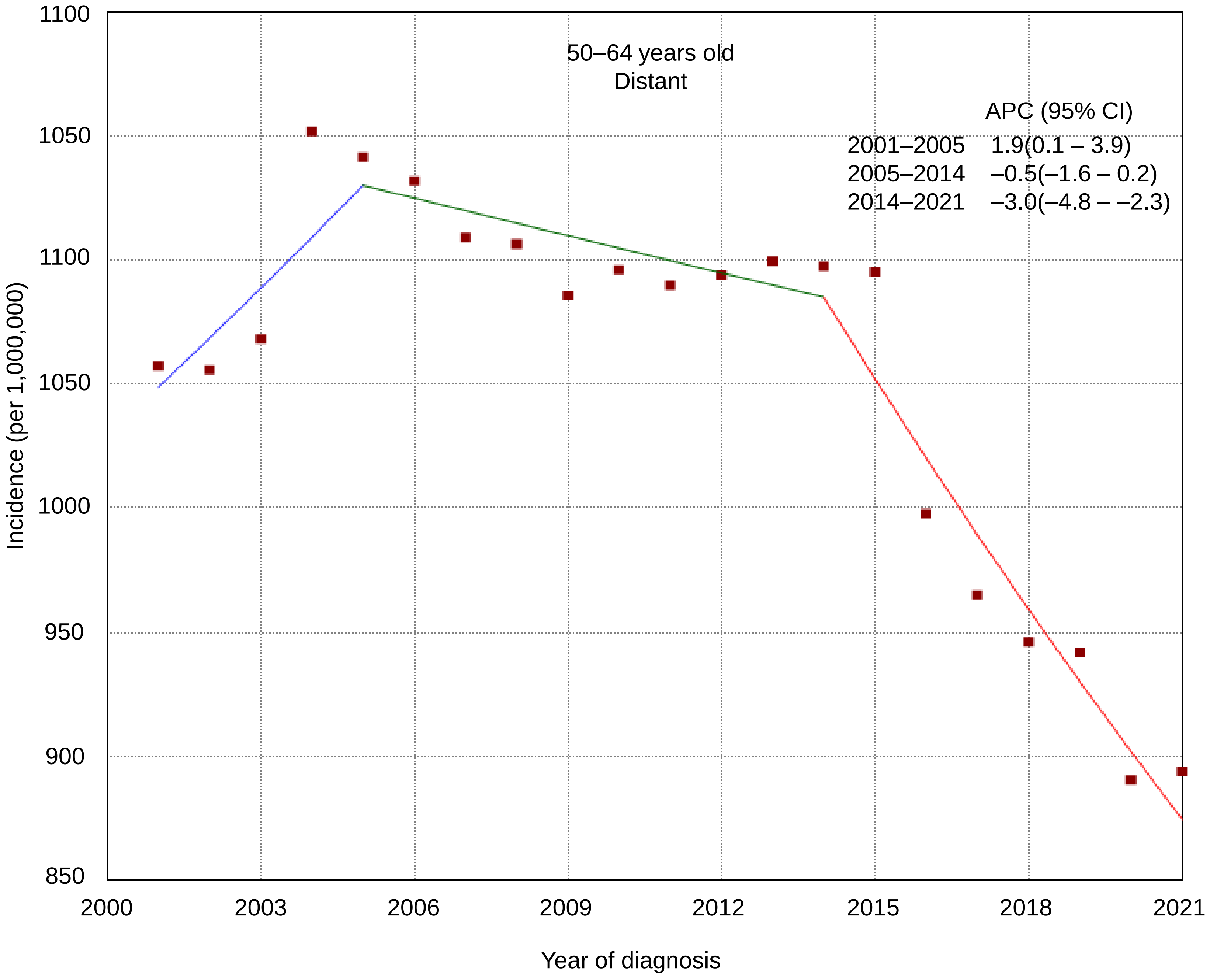

50–64 years old
Distant
APC (95% CI)
2001–2005 1.9(0.1 – 3.9)
2005–2014 –0.5(–1.6 – 0.2)
2014–2021 –3.0(–4.8 – –2.3)
1100
1050
1100
1050
1000
950
900
850
Incidence (per 1,000,000)
2000
2003
2006
2009
2012
2015
2018
2021
Year of diagnosis

Figure S8. (A)

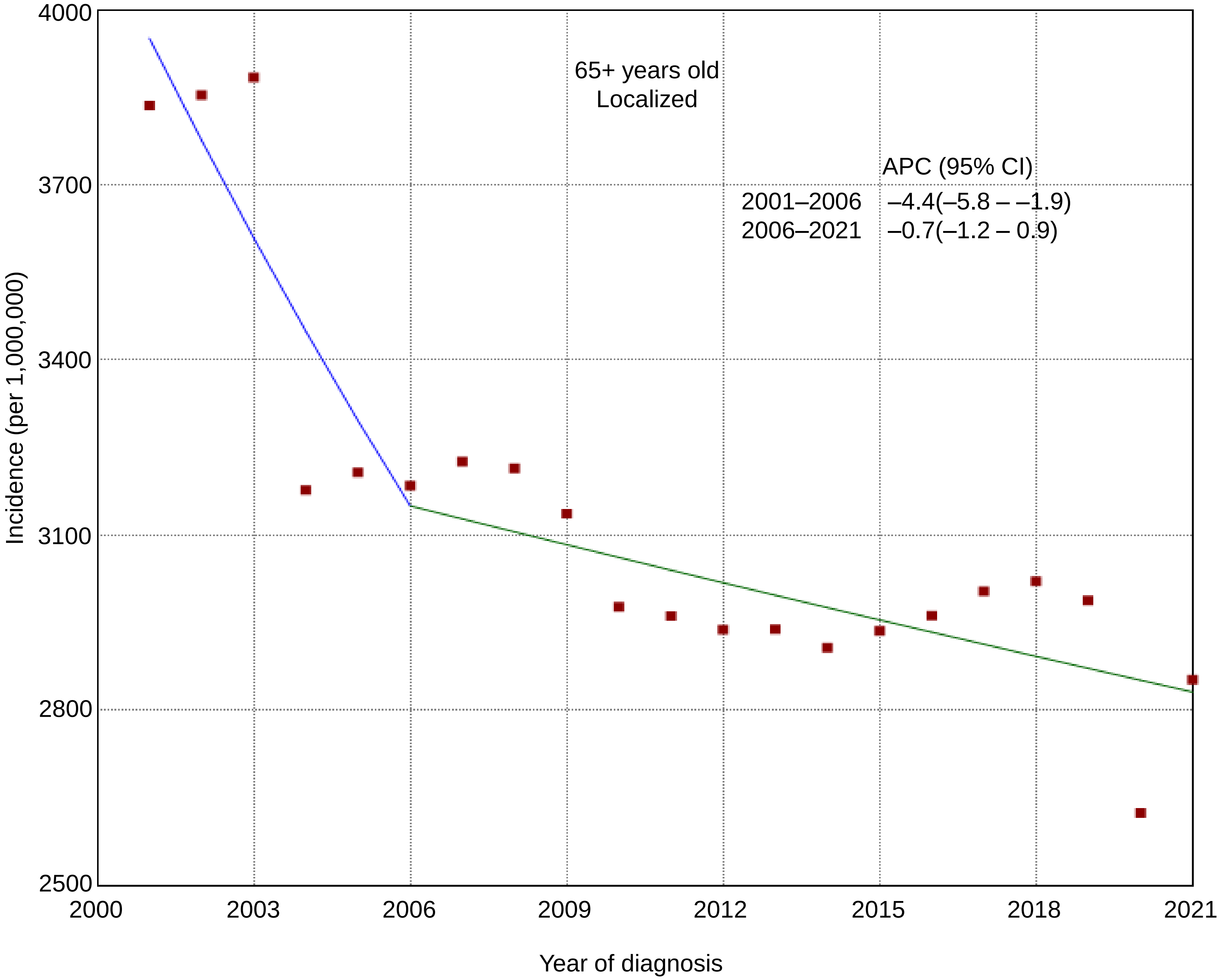

65+ years old
Localized
APC (95% CI)
2001–2006 –4.4(–5.8 – –1.9)
2006–2021 –0.7(–1.2 – 0.9)
Incidence (per 1,000,000)
4000
3700
3400
3100
2800
2500
2000
2003
2006
2009
2012
2015
2018
2021
Year of diagnosis

Figure S8. (B)

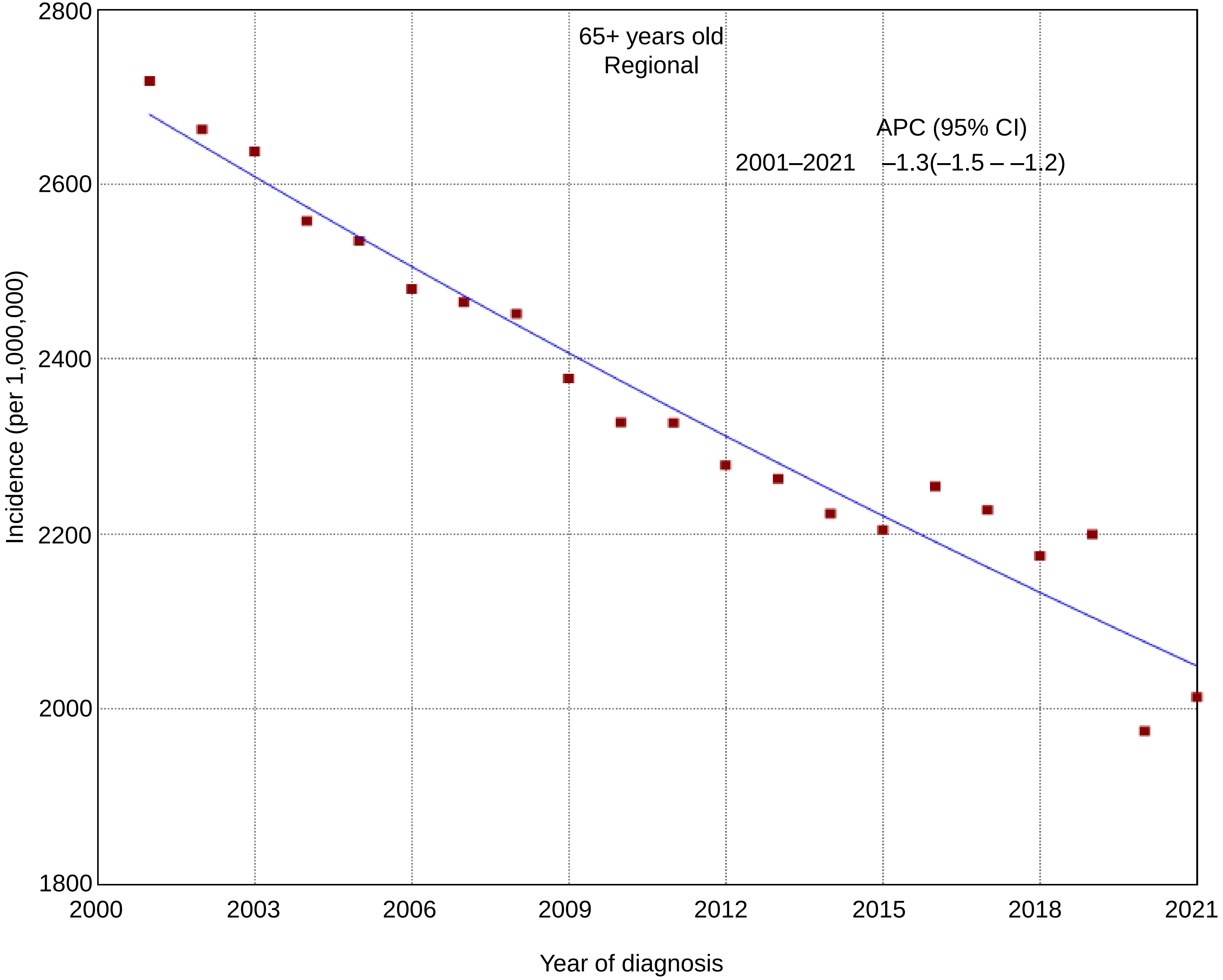
65+ years old
Regional
APC (95% CI)
2001–2021 –1.3(–1.5 – –1.2)
Incidence (per 1,000,000)
2800
2600
2400
2200
2000
1800
2000
2003
2006
2009
2012
2015
2018
2021
Year of diagnosis

Figure S8. (C)

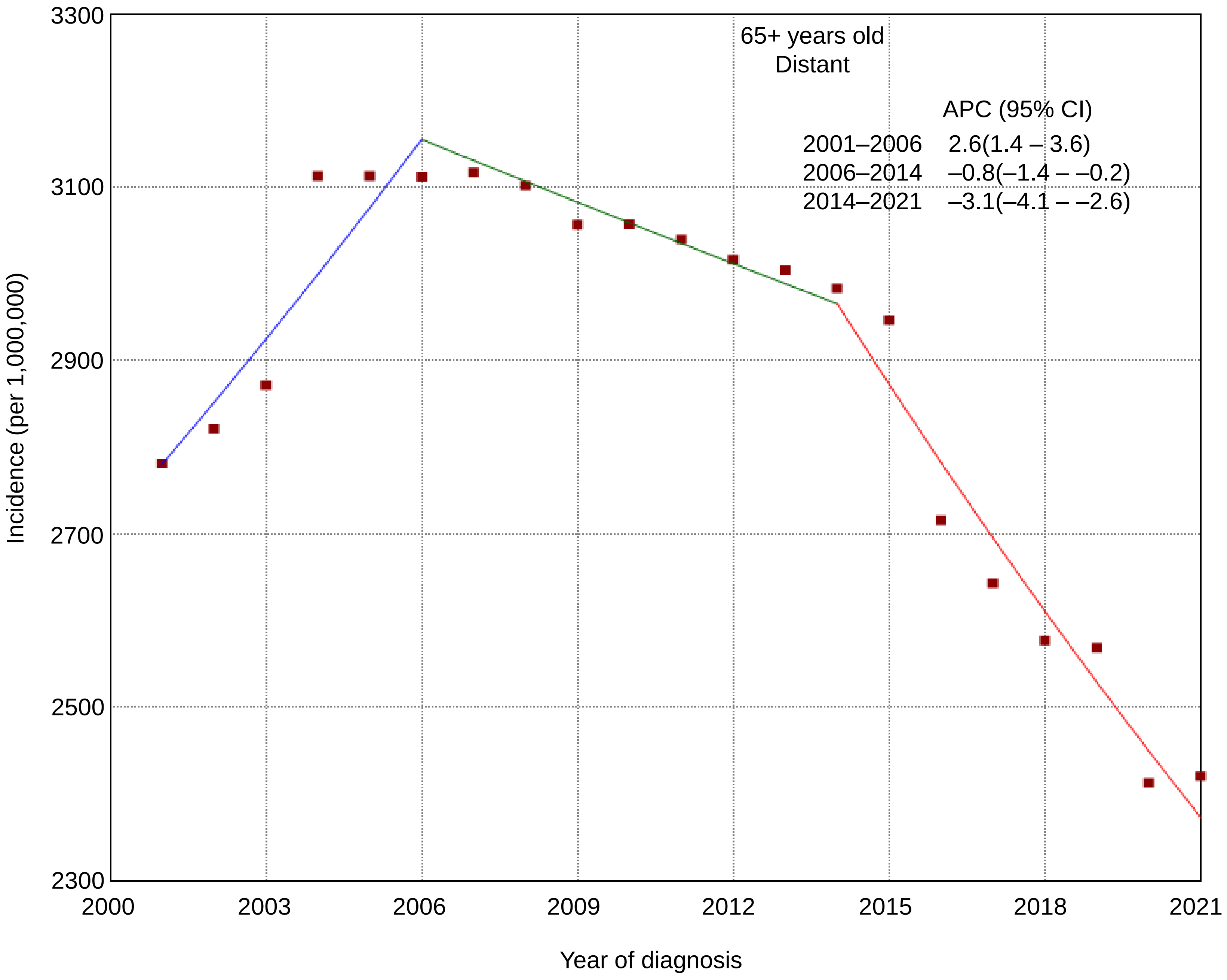

65+ years old
Distant
APC (95% CI)
2001–2006 2.6(1.4 – 3.6)
2006–2014 –0.8(–1.4 – –0.2)
2014–2021 –3.1(–4.1 – –2.6)
3300
3100
2900
2700
2500
2300
Incidence (per 1,000,000)
2000
2003
2006
2009
2012
2015
2018
2021
Year of diagnosis

Figure S9. (A)

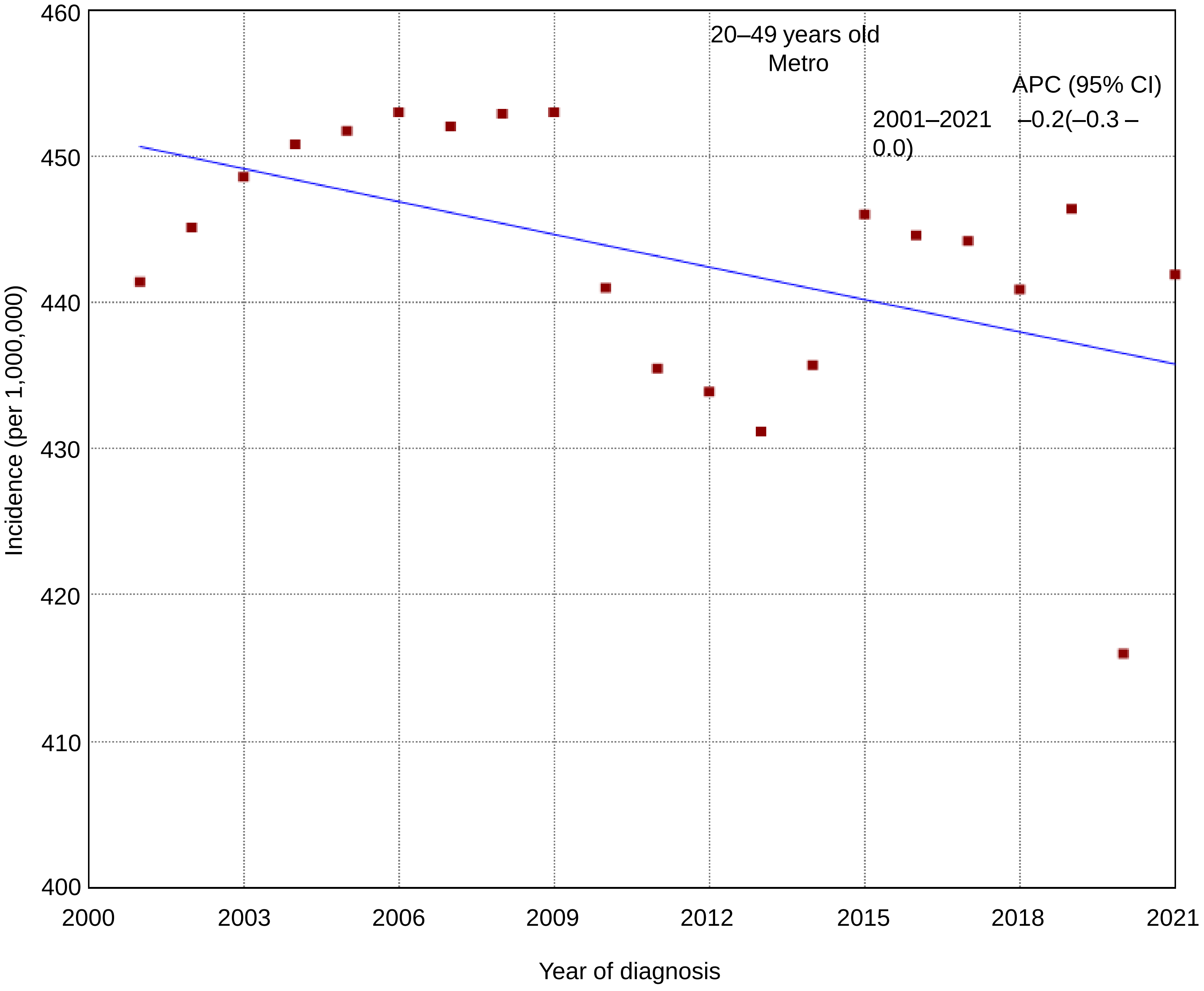
20–49 years old
Metro
APC (95% CI)
2001–2021
–0.2(–0.3 – 0.0)
460
450
440
430
420
410
400
Incidence (per 1,000,000)
2000
2003
2006
2009
2012
2015
2018
2021
Year of diagnosis

Figure S9. (B)

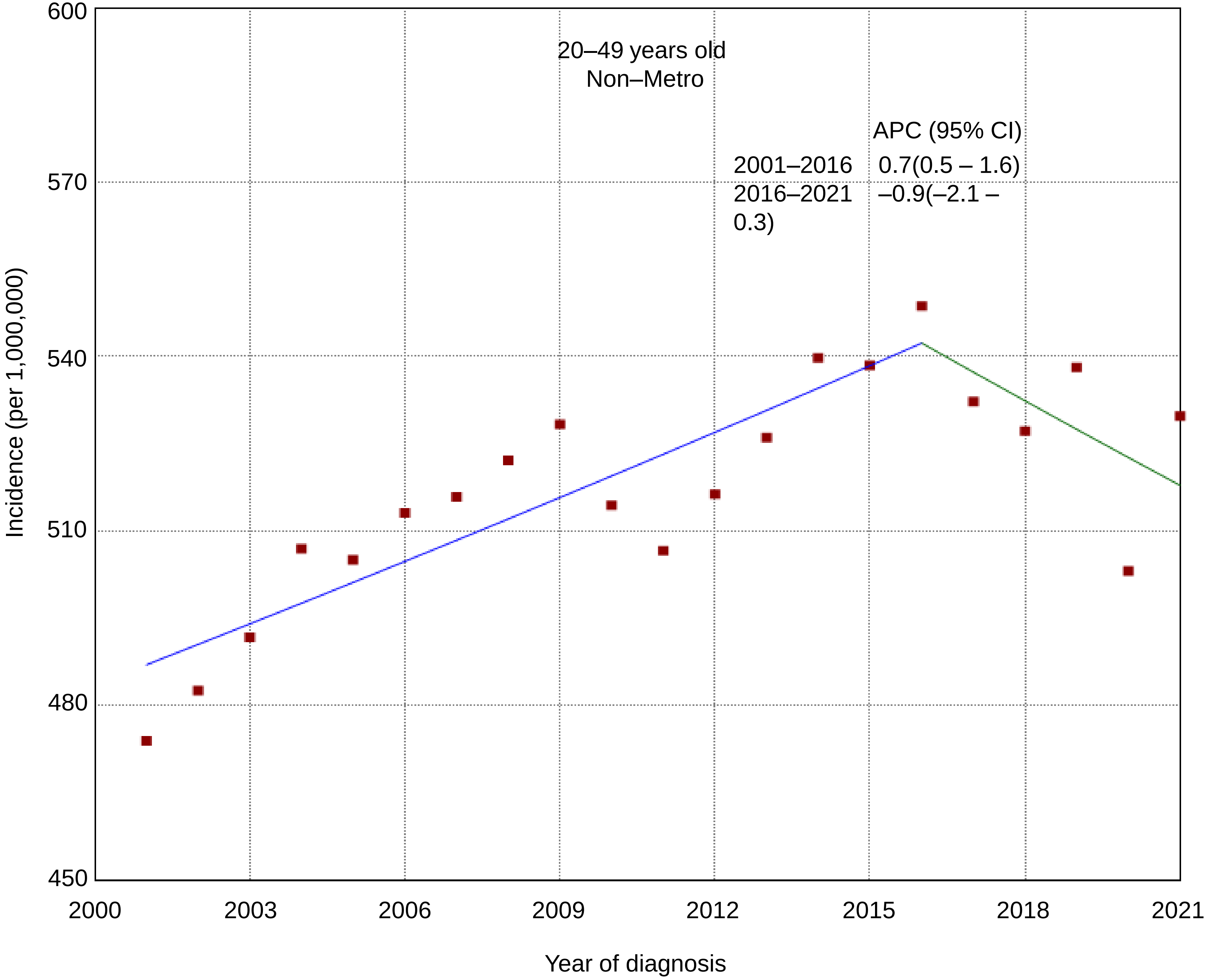

600
570
540
510
480
450
20–49 years old
Non–Metro
APC (95% CI)
2001–2016 0.7(0.5 – 1.6)
2016–2021 –0.9(–2.1 – 0.3)
Incidence (per 1,000,000)
2000
2003
2006
2009
2012
2015
2018
2021
Year of diagnosis

Figure S10. (A)

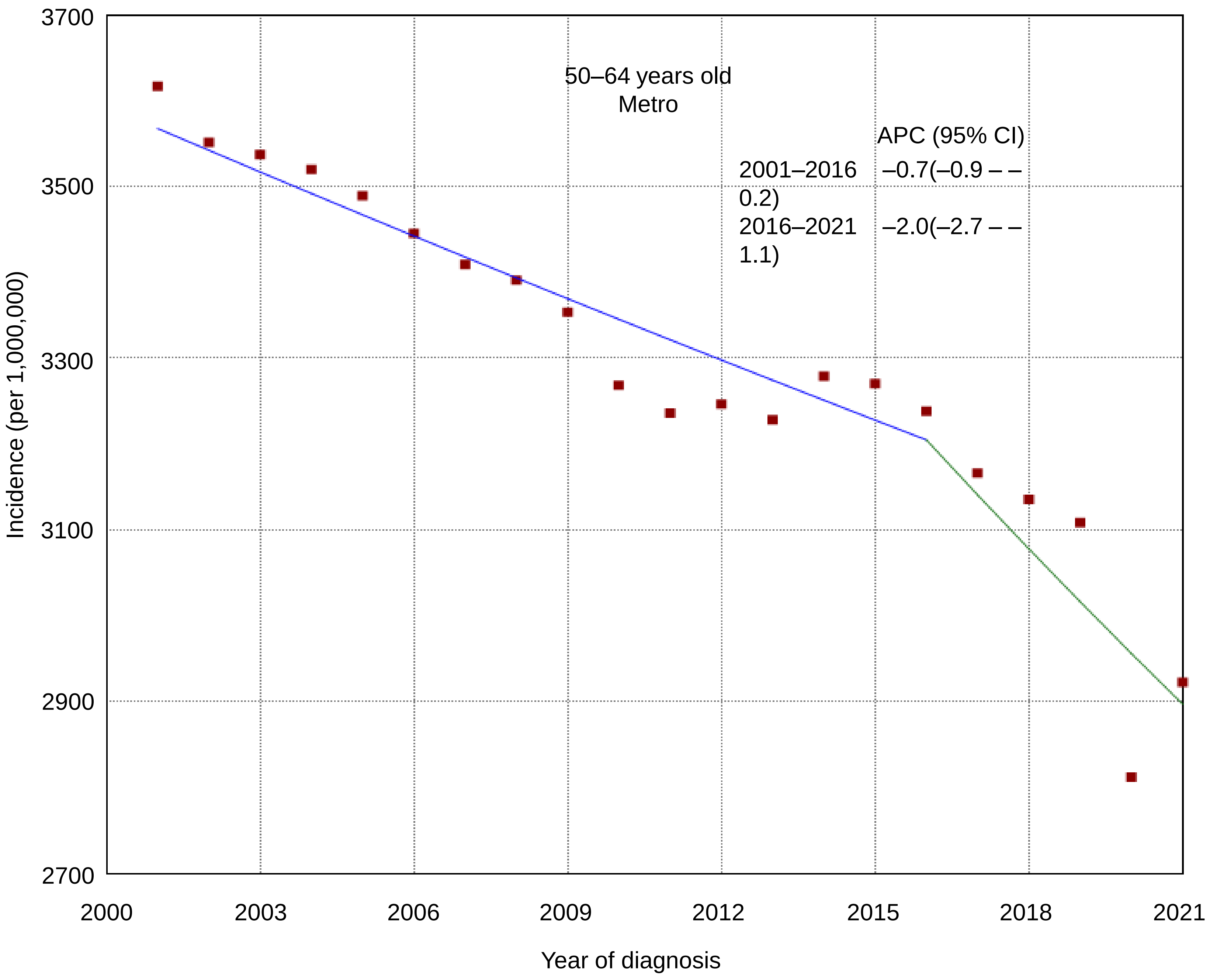
50–64 years old
Metro
APC (95% CI)
2001–2016 –0.7(–0.9 – – 0.2)
2016–2021 –2.0(–2.7 – – 1.1)
3700
3500
3300
3100
2900
2700
Incidence (per 1,000,000)
2000
2003
2006
2009
2012
2015
2018
2021
Year of diagnosis

Figure S10. (B)

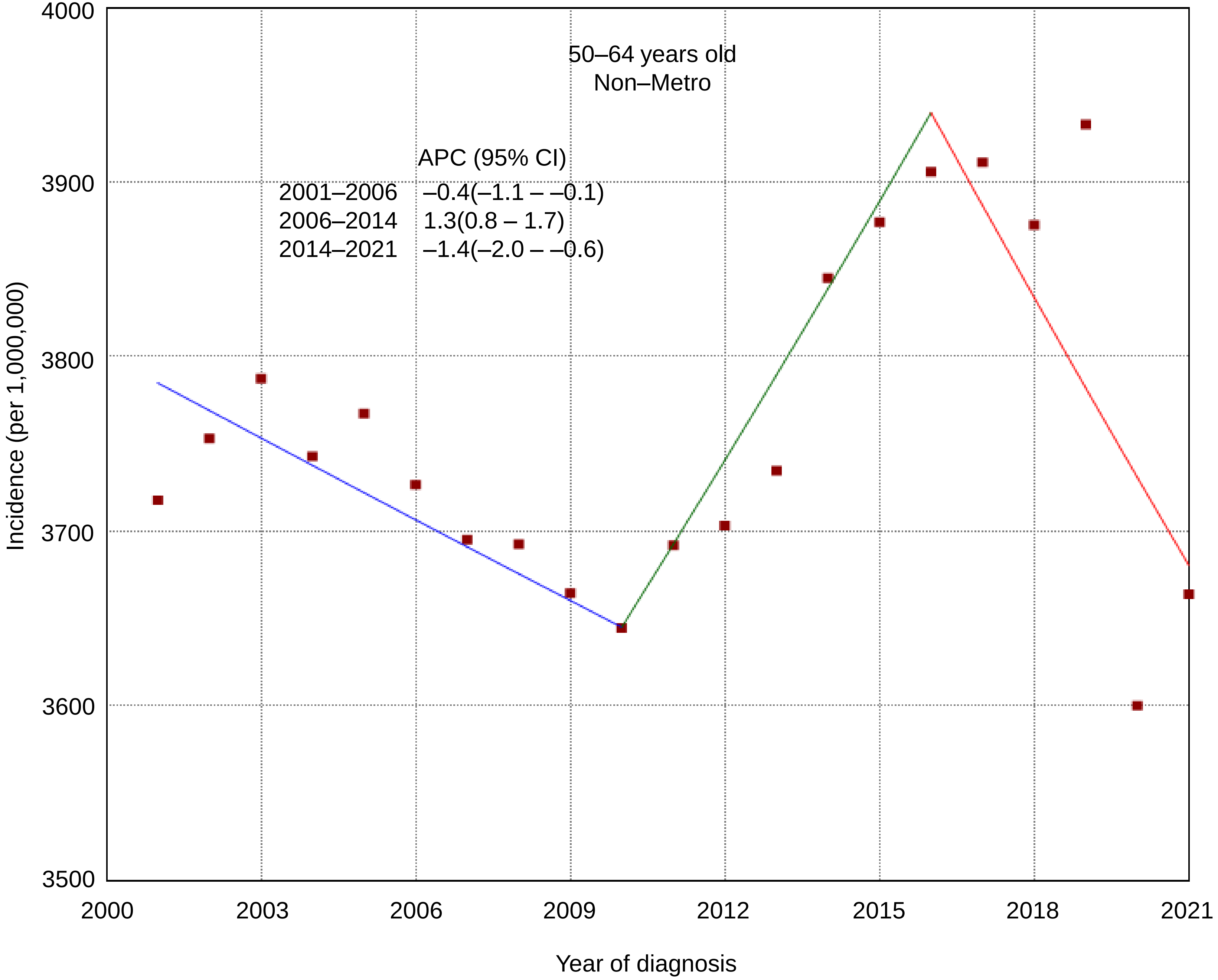

50–64 years old
Non–Metro
APC (95% CI)
2001–2006 –0.4(–1.1 – –0.1)
2006–2014 1.3(0.8 – 1.7)
2014–2021 –1.4(–2.0 – –0.6)
Incidence (per 1,000,000)
4000
3900
3800
3700
3600
3500
2000
2003
2006
2009
2012
2015
2018
2021
Year of diagnosis

Figure S11. (A)

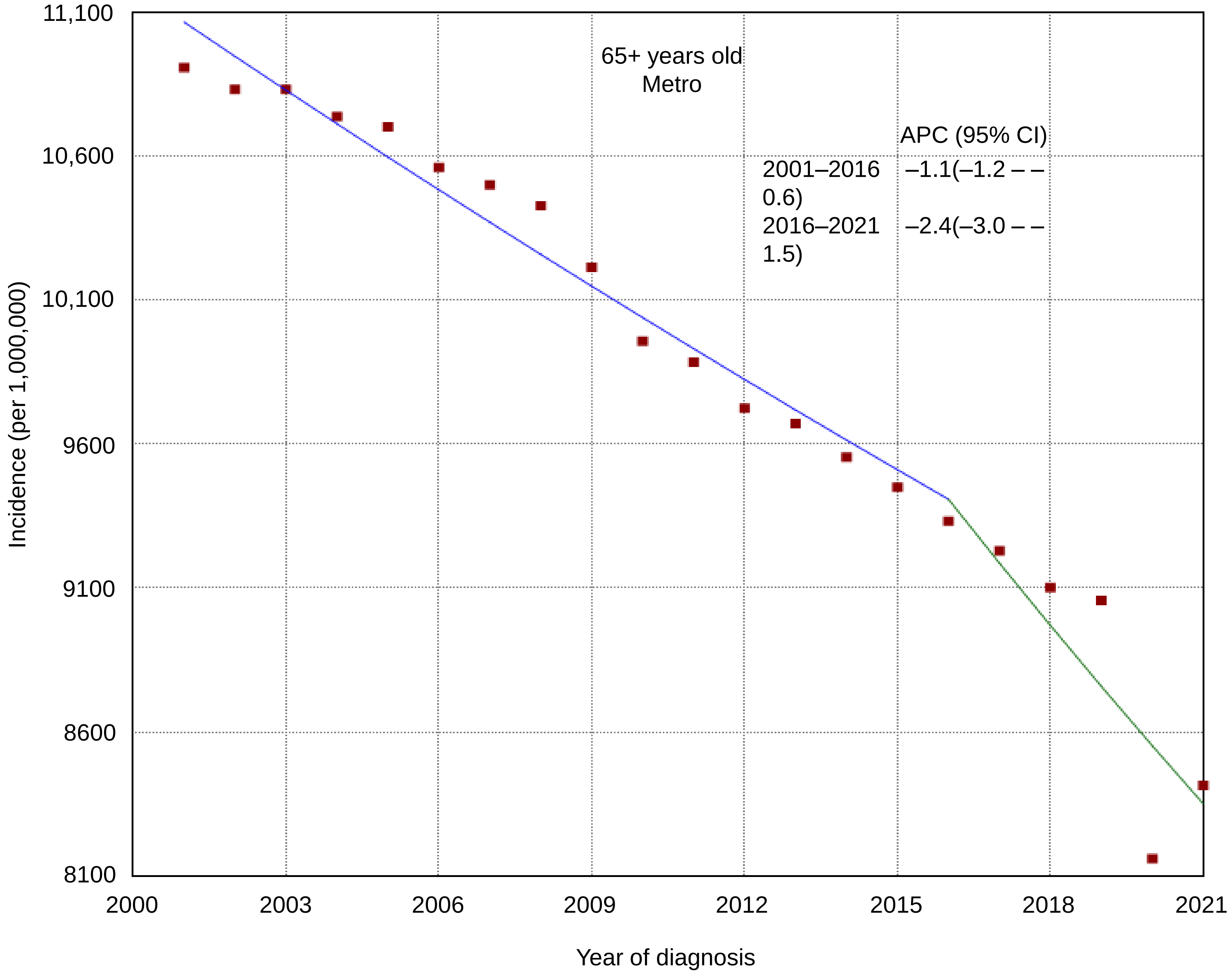

11,100
10,600
10,100
9600
9100
8600
8100
Incidence (per 1,000,000)
2000
2003
2006
2009
2012
2015
2018
2021
Year of diagnosis
65+ years old
Metro
APC (95% CI)
2001–2016 –1.1(–1.2 – – 0.6)
2016–2021 –2.4(–3.0 – – 1.5)

Figure S11. (B)

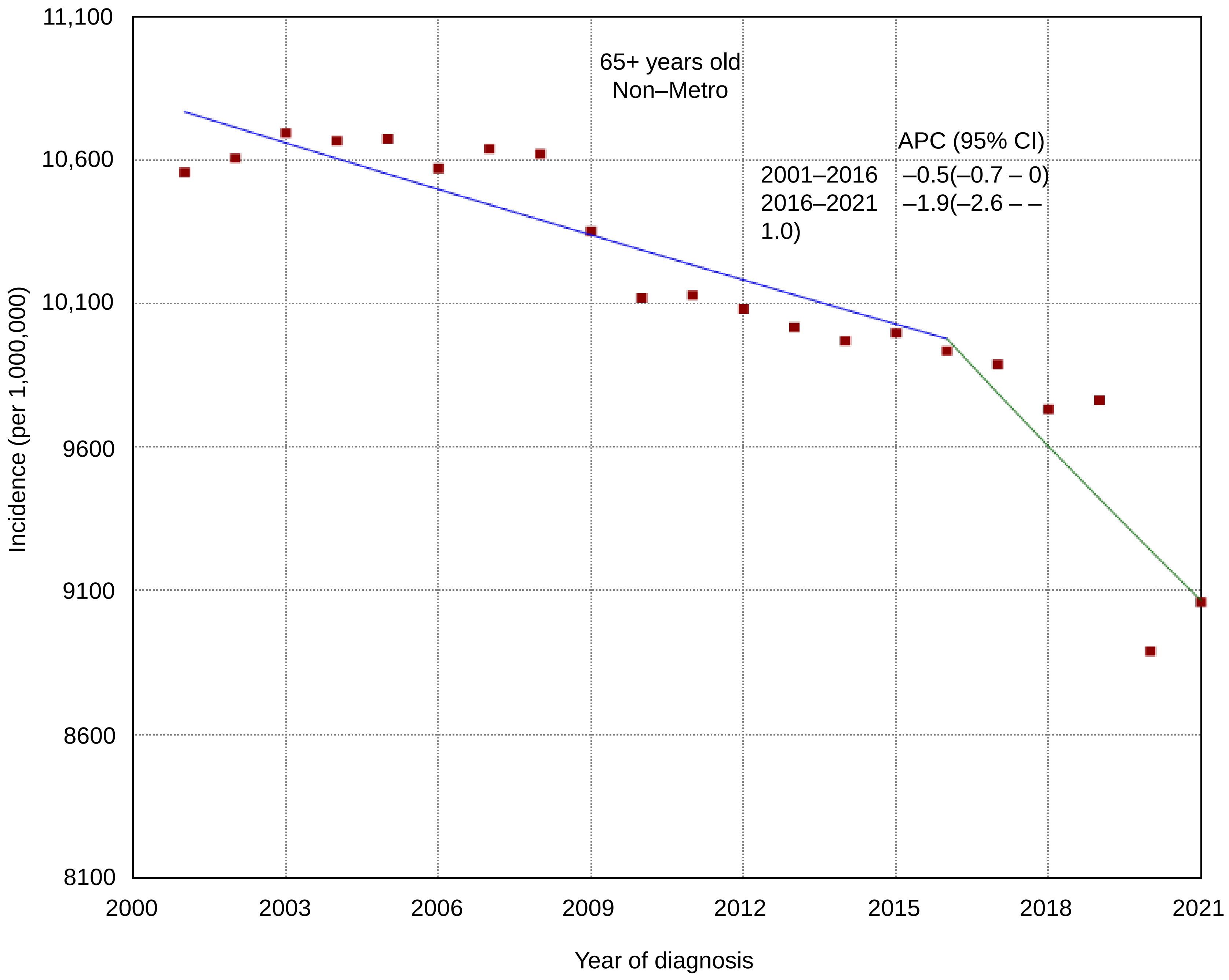
65+ years old
Non–Metro
APC (95% CI)
2001–2016 –0.5(–0.7 – 0)
2016–2021 –1.9(–2.6 – –1.0)
11,100
10,600
10,100
9600
9100
8600
8100
Incidence (per 1,000,000)
2000
2003
2006
2009
2012
2015
2018
2021
Year of diagnosis

Figure S12. (A)

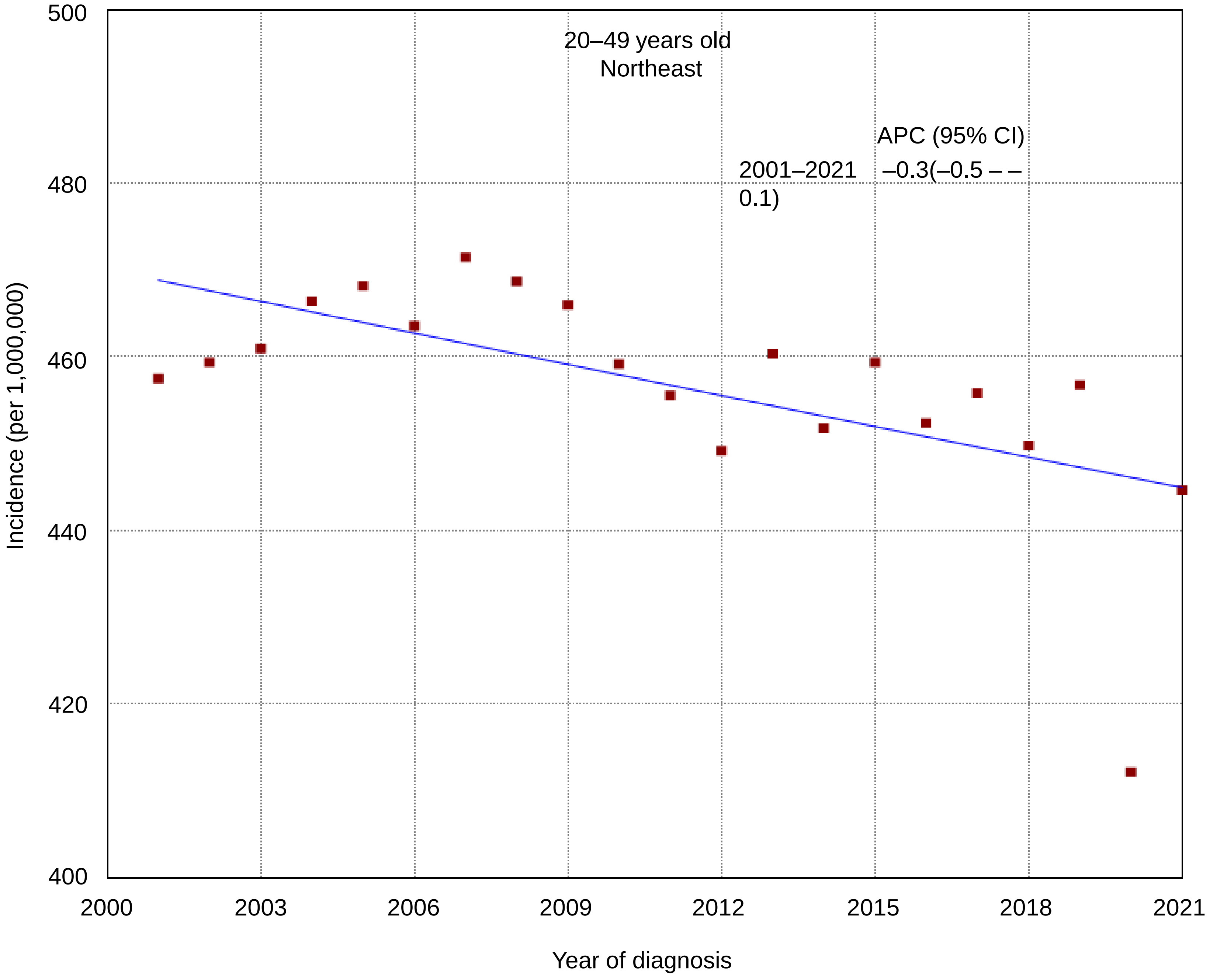

20–49 years old
Northeast
APC (95% CI)
2001–2021
−0.3(−0.5 – –
0.1)
500
480
460
440
420
400
Incidence (per 1,000,000)
2000
2003
2006
2009
2012
2015
2018
2021
Year of diagnosis

Figure S12. (B)

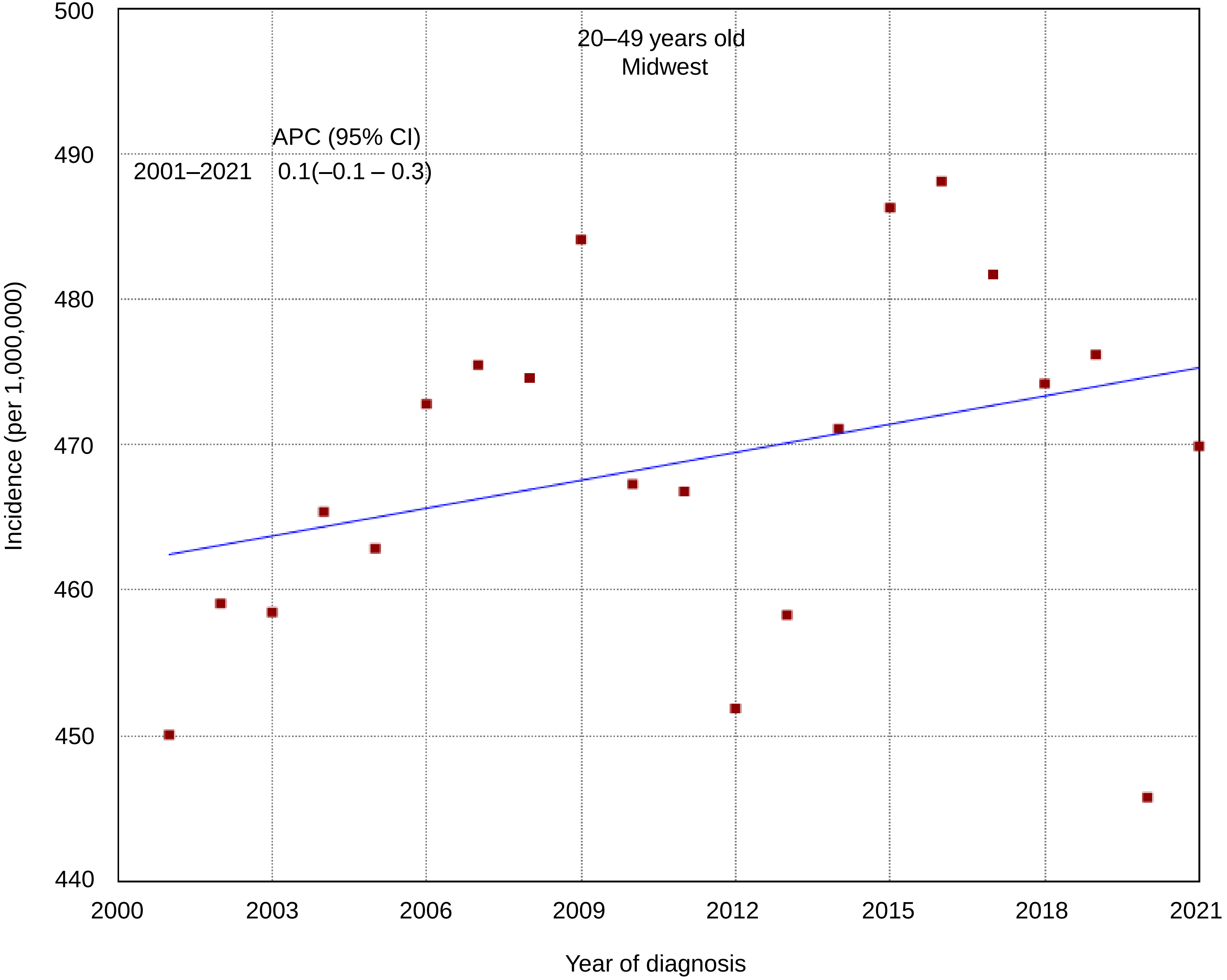
20–49 years old
Midwest
APC (95% CI)
2001–2021 0.1(–0.1 – 0.3)
Incidence (per 1,000,000)
500
490
480
470
460
450
440
2000
2003
2006
2009
2012
2015
2018
2021
Year of diagnosis

Figure S12. (C)

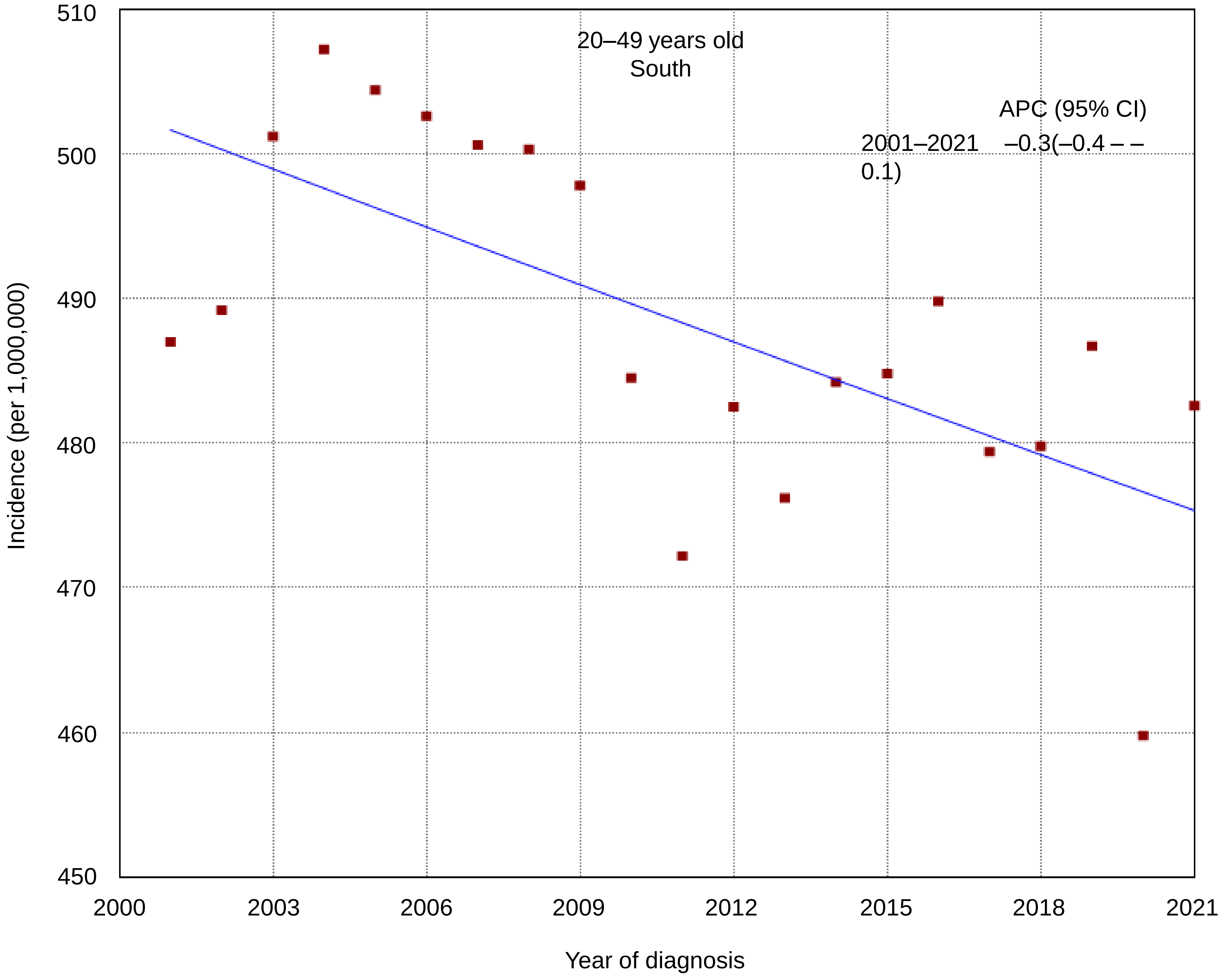

20–49 years old
South
APC (95% CI)
2001–2021 –0.3(–0.4 – –0.1)
510
500
490
480
470
460
450
Incidence (per 1,000,000)
2000
2003
2006
2009
2012
2015
2018
2021
Year of diagnosis

Figure S12. (D)

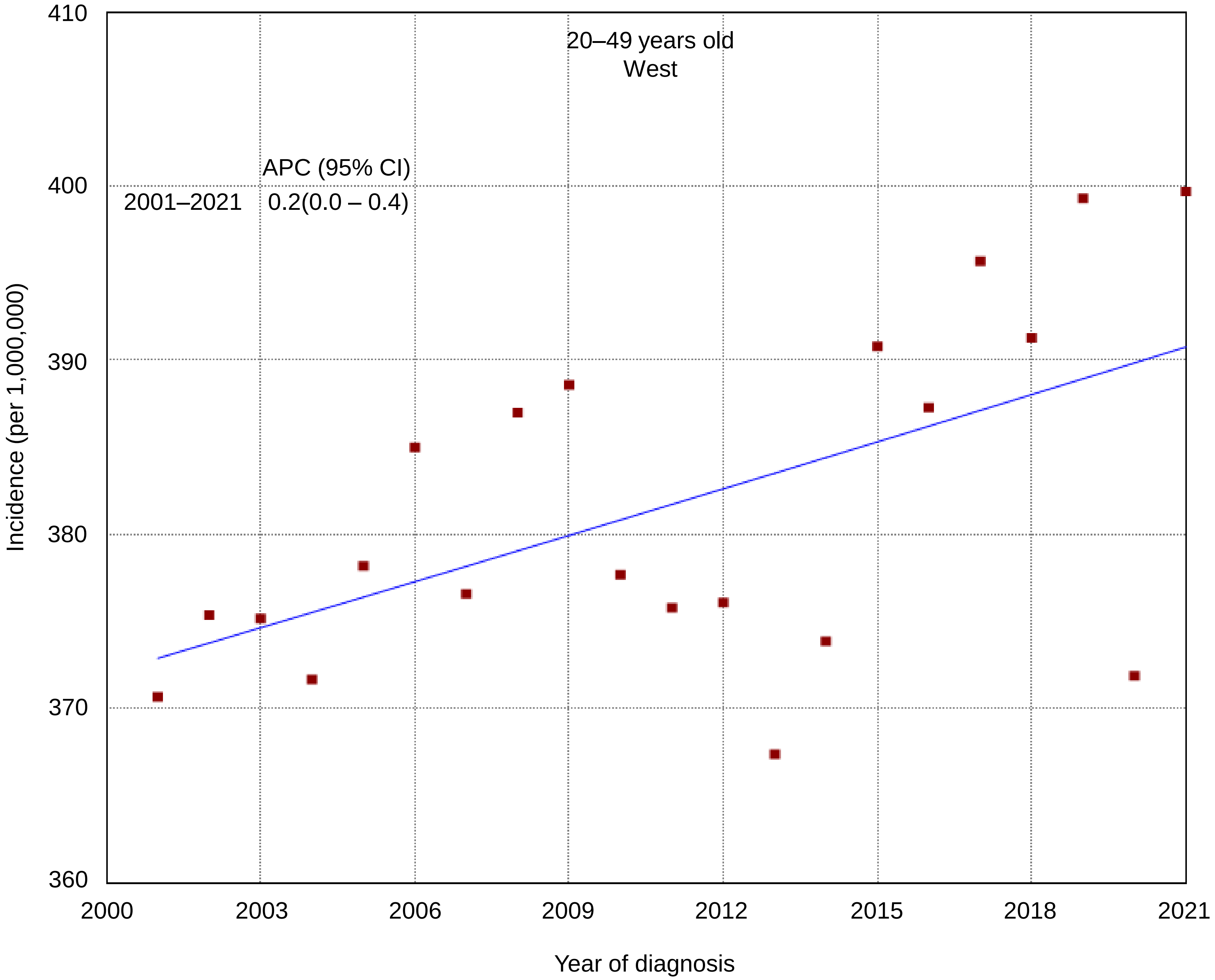

20–49 years old
West
APC (95% CI)
2001–2021
0.2(0.0 – 0.4)
410
400
390
380
370
360
Incidence (per 1,000,000)
2000
2003
2006
2009
2012
2015
2018
2021
Year of diagnosis

Figure S13. (A)

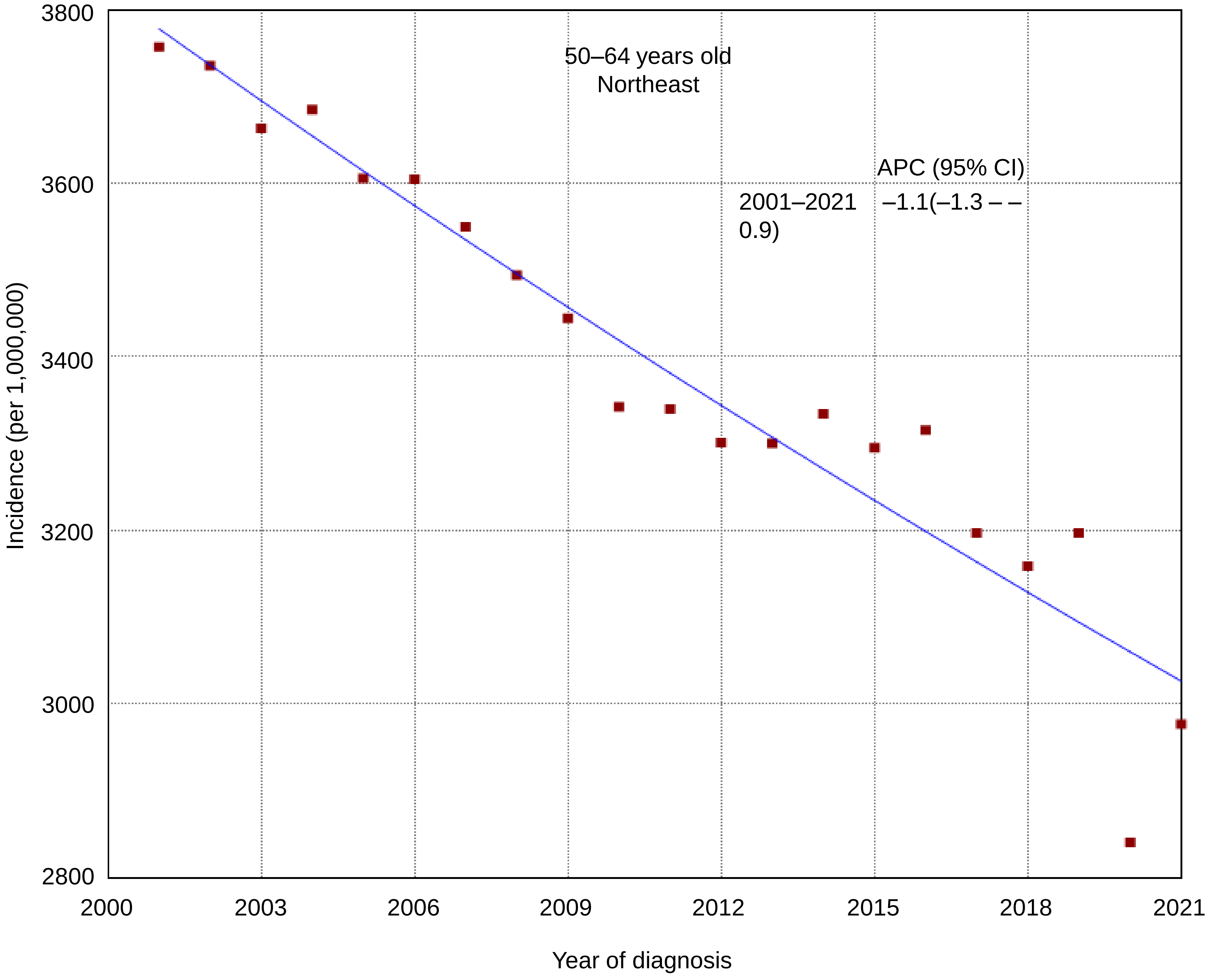

50–64 years old
Northeast
APC (95% CI)
2001–2021
–1.1(–1.3 – –
0.9)
Incidence (per 1,000,000)
3800
3600
3400
3200
3000
2800
2000
2003
2006
2009
2012
2015
2018
2021
Year of diagnosis

Figure S13. (B)

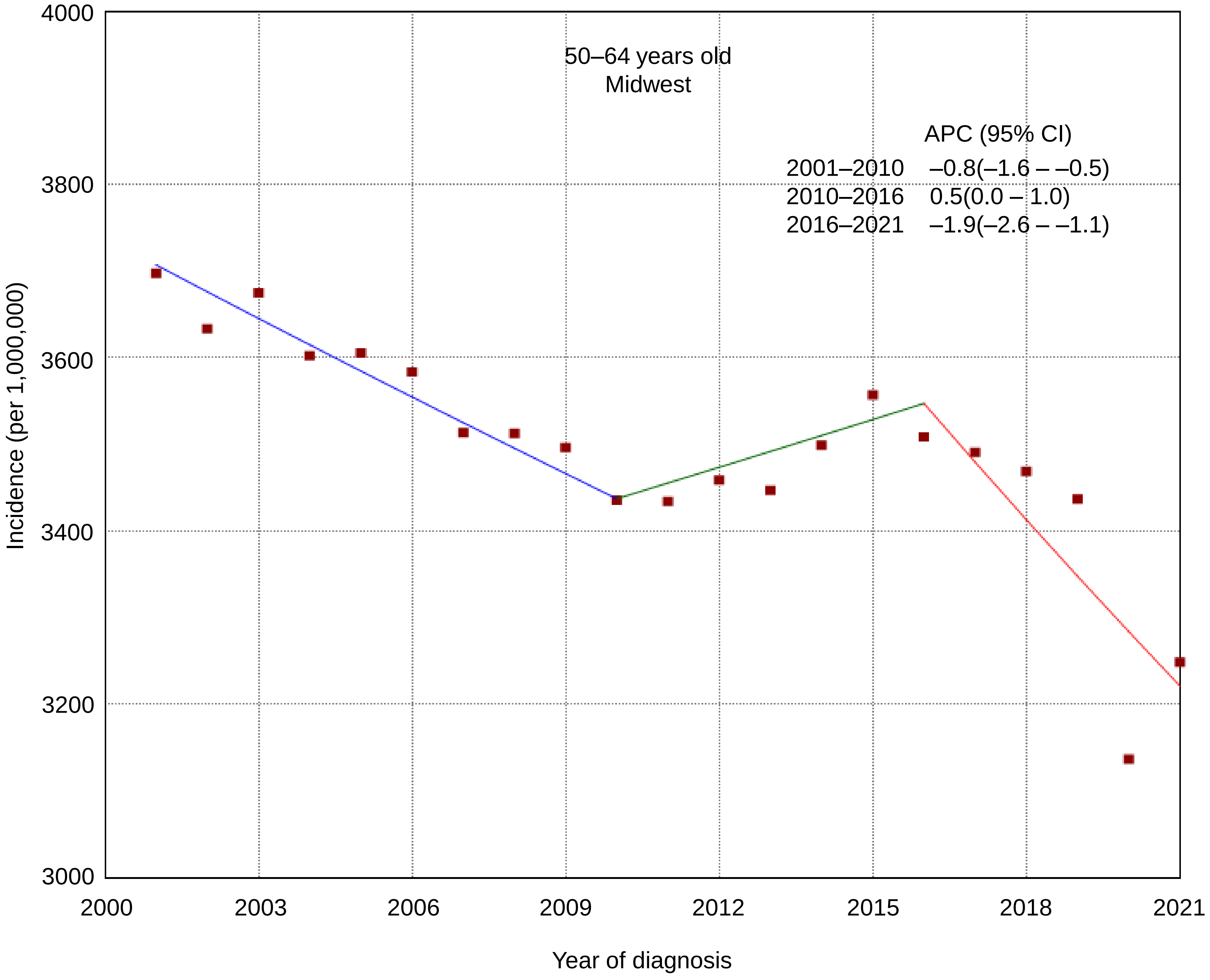
50–64 years old
Midwest
APC (95% CI)
2001–2010 –0.8(–1.6 – –0.5)
2010–2016 0.5(0.0 – 1.0)
2016–2021 –1.9(–2.6 – –1.1)
Incidence (per 1,000,000)
4000
3800
3600
3400
3200
3000
2000
2003
2006
2009
2012
2015
2018
2021
Year of diagnosis

Figure S13. (C)

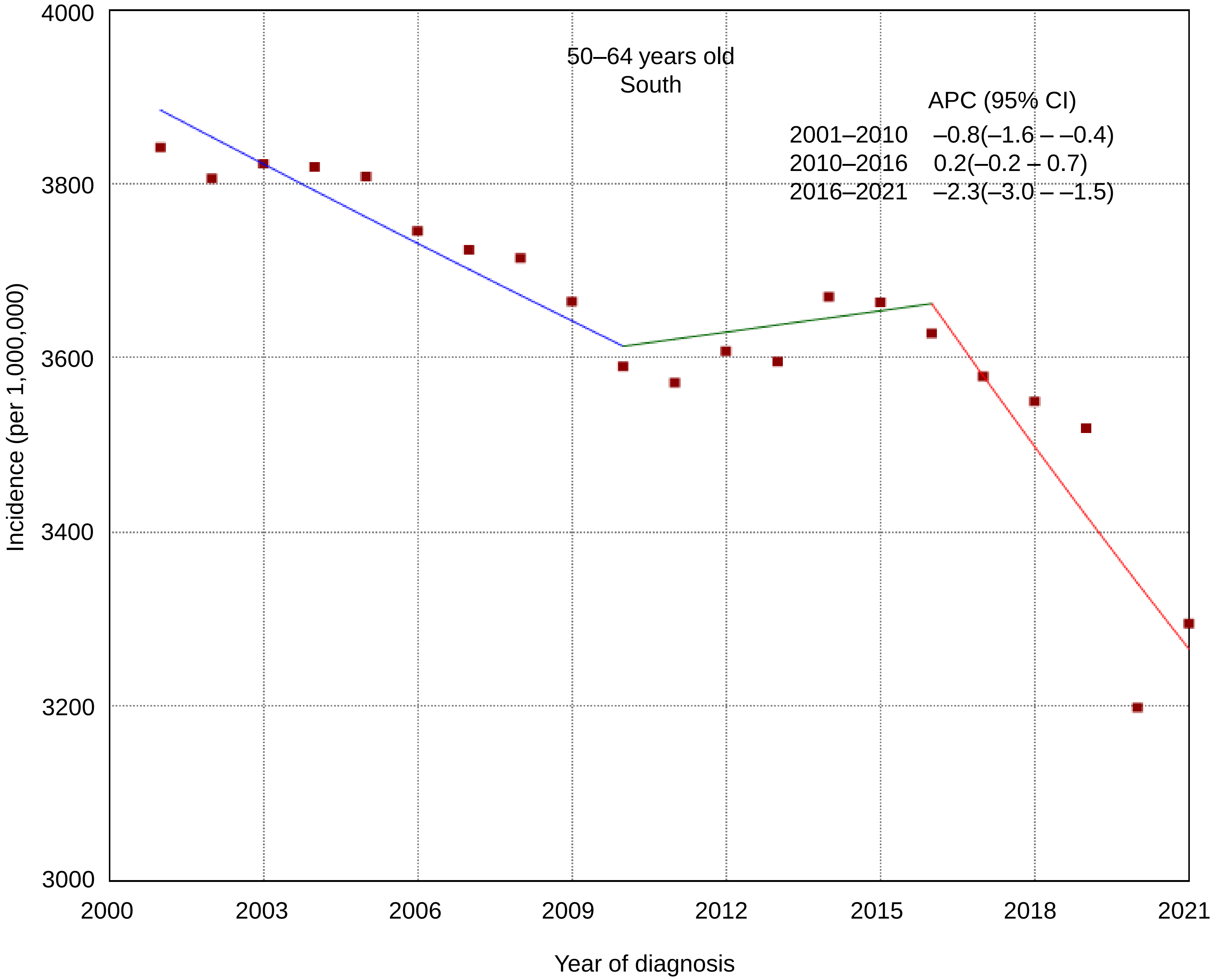
50–64 years old
South
APC (95% CI)
2001–2010 –0.8(–1.6 – –0.4)
2010–2016 0.2(–0.2 – 0.7)
2016–2021 –2.3(–3.0 – –1.5)
Incidence (per 1,000,000)
4000
3800
3600
3400
3200
3000
2000
2003
2006
2009
2012
2015
2018
2021
Year of diagnosis

Figure S13. (D)

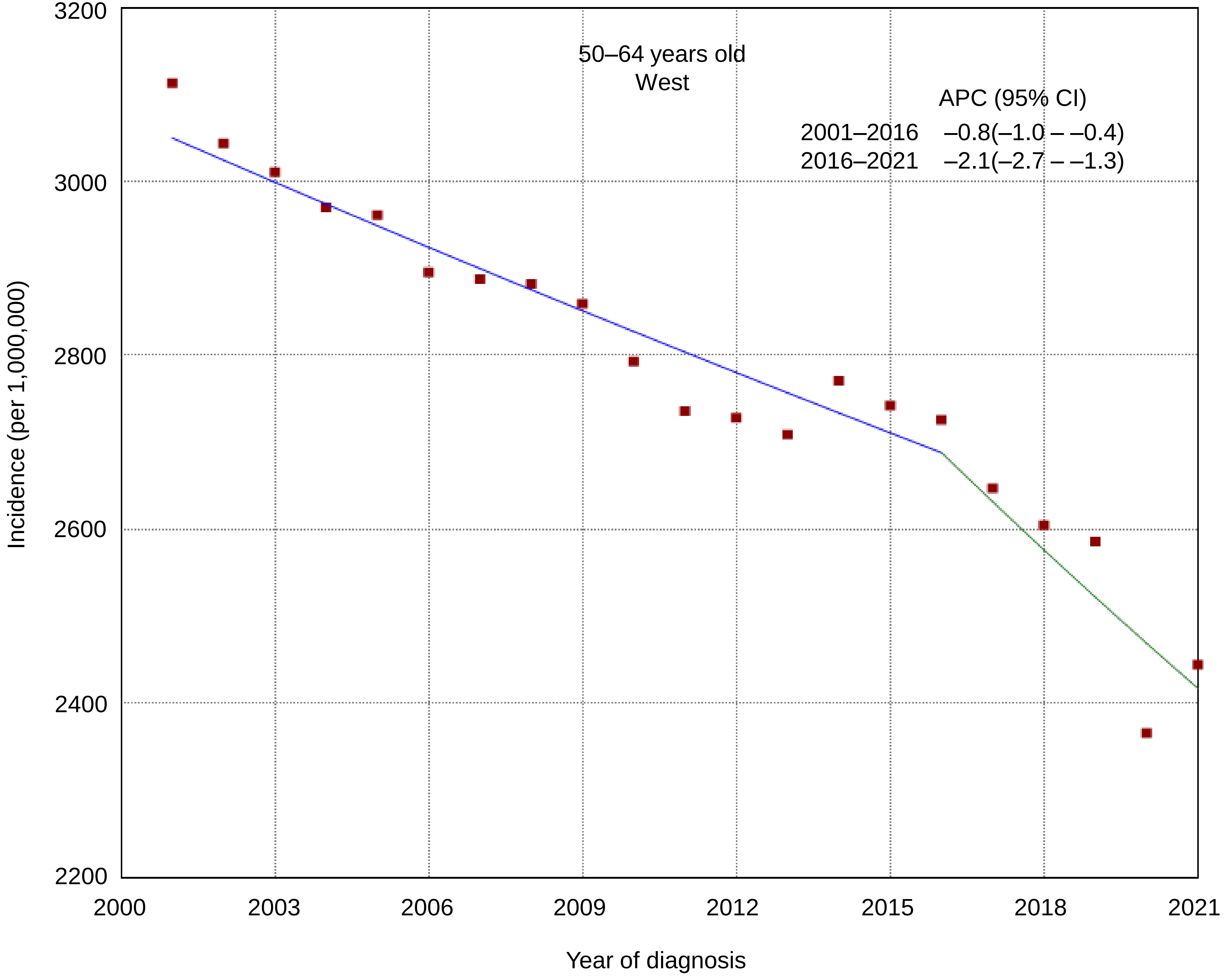

50–64 years old
West
APC (95% CI)
2001–2016 –0.8(–1.0 – –0.4)
2016–2021 –2.1(–2.7 – –1.3)
Incidence (per 1,000,000)
3200
3000
2800
2600
2400
2200
2000
2003
2006
2009
2012
2015
2018
2021
Year of diagnosis

Figure S14. (A)

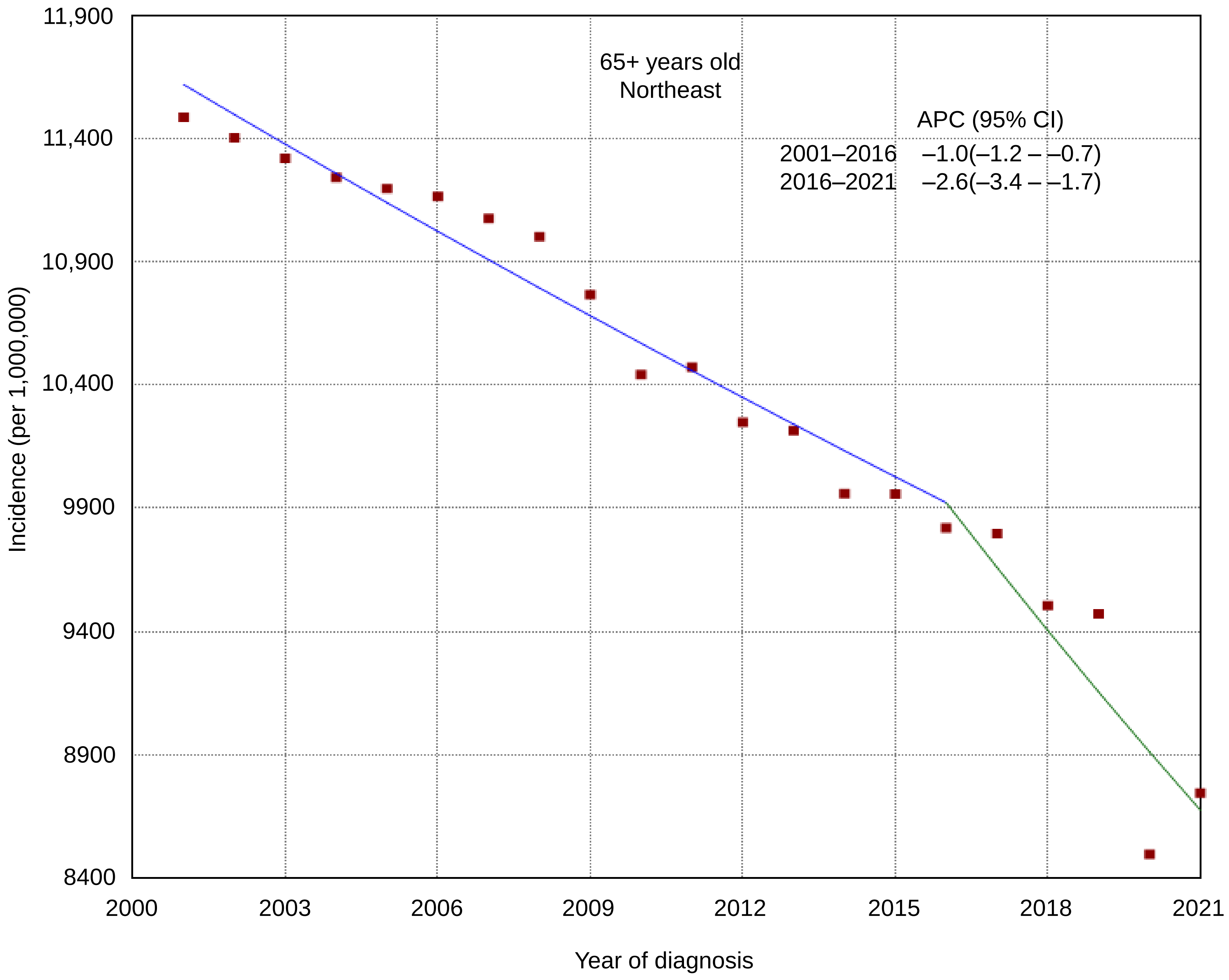
65+ years old
Northeast
APC (95% CI)
2001–2016 −1.0(−1.2 – −0.7)
2016–2021 −2.6(−3.4 – −1.7)
11,900
11,400
10,900
10,400
9900
9400
8900
8400
Incidence (per 1,000,000)
2000
2003
2006
2009
2012
2015
2018
2021
Year of diagnosis

Figure S14. (B)

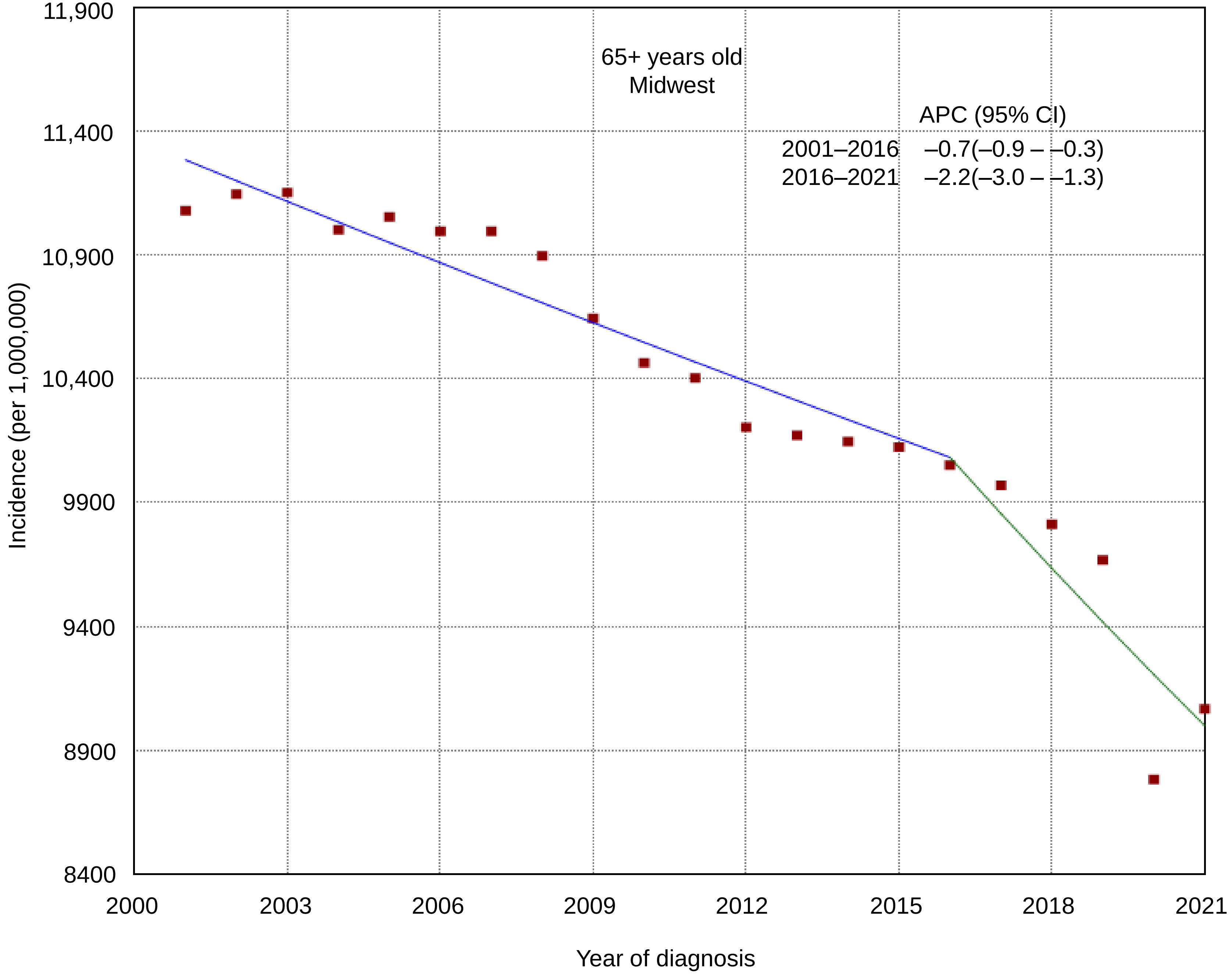
65+ years old
Midwest
APC (95% CI)
2001–2016 –0.7(–0.9 – –0.3)
2016–2021 –2.2(–3.0 – –1.3)
Incidence (per 1,000,000)
11,900
11,400
10,900
10,400
9900
9400
8900
8400
2000
2003
2006
2009
2012
2015
2018
2021
Year of diagnosis

Figure S14. (C)

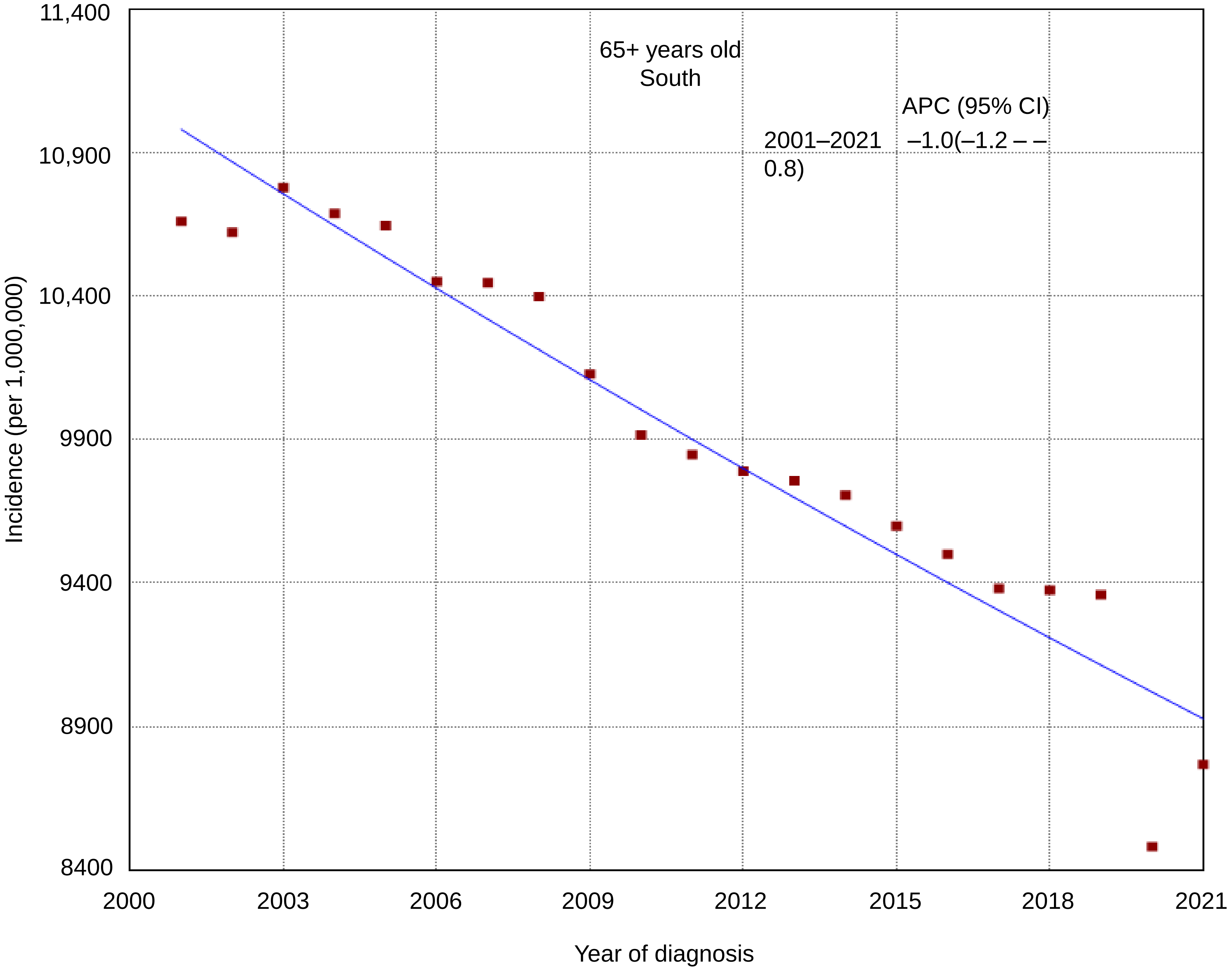

11,400
10,900
10,400
9900
9400
8900
8400
Incidence (per 1,000,000)
65+ years old
South
APC (95% CI)
2001–2021
−1.0(−1.2 – –
0.8)
2000
2003
2006
2009
2012
2015
2018
2021
Year of diagnosis

Figure S14. (D)

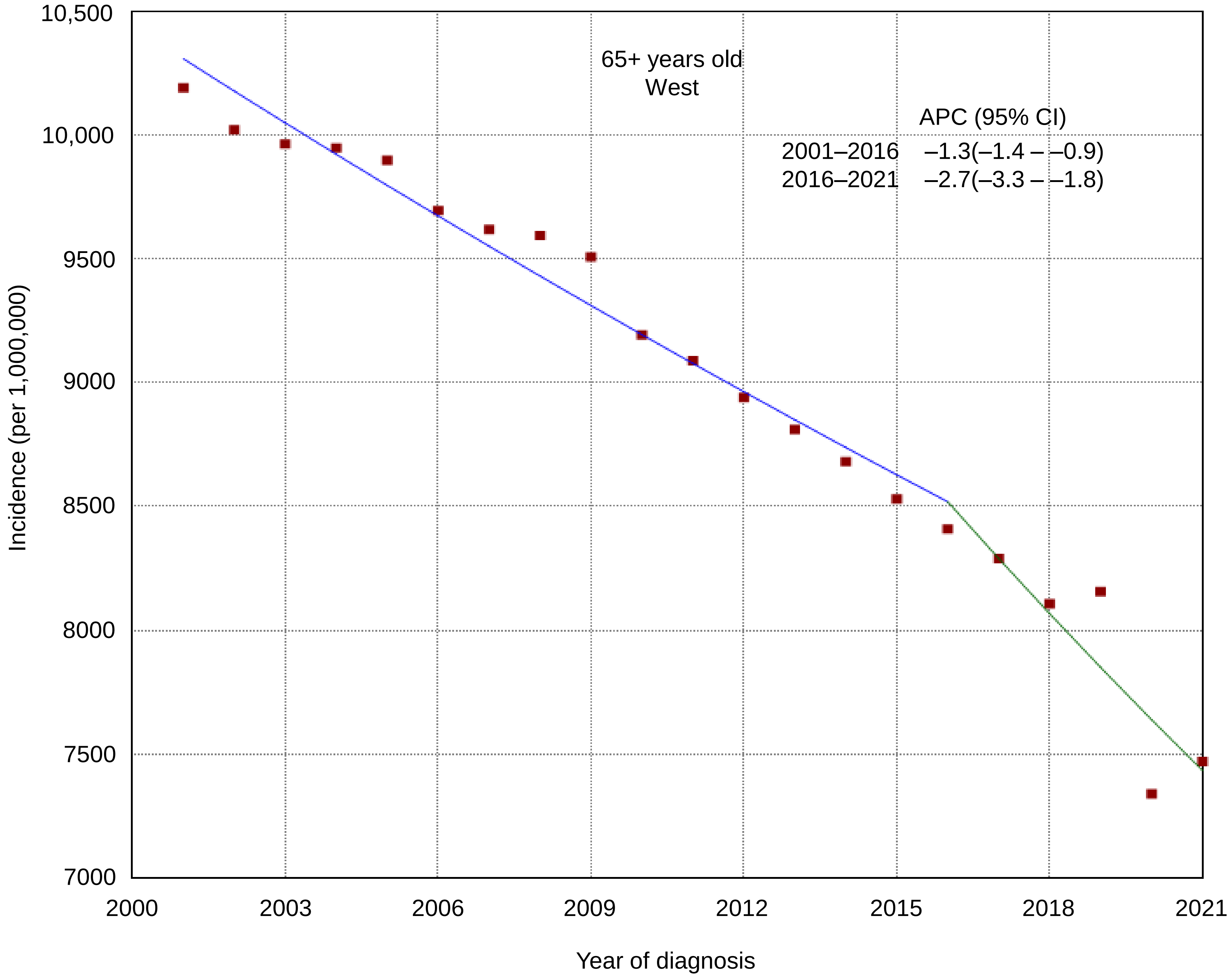
65+ years old
West
APC (95% CI)
2001–2016 –1.3(–1.4 – –0.9)
2016–2021 –2.7(–3.3 – –1.8)
10,500
10,000
9500
9000
8500
8000
7500
7000
Incidence (per 1,000,000)
2000
2003
2006
2009
2012
2015
2018
2021
Year of diagnosis

Figure S15. (A)

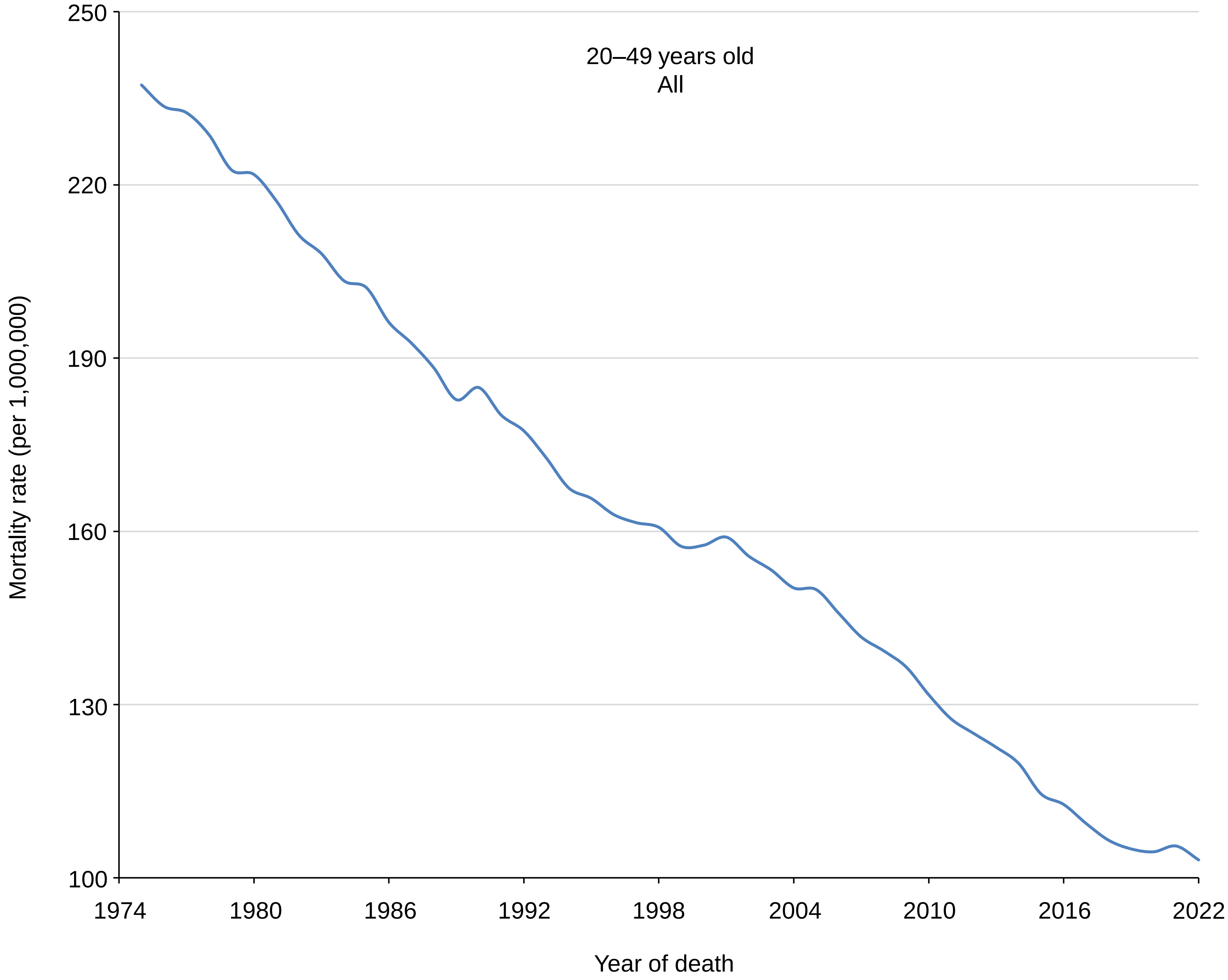

20–49 years old
All
Mortality rate (per 1,000,000)
250
220
190
160
130
100
1974
1980
1986
1992
1998
2004
2010
2016
2022
Year of death

Figure S15. (B)

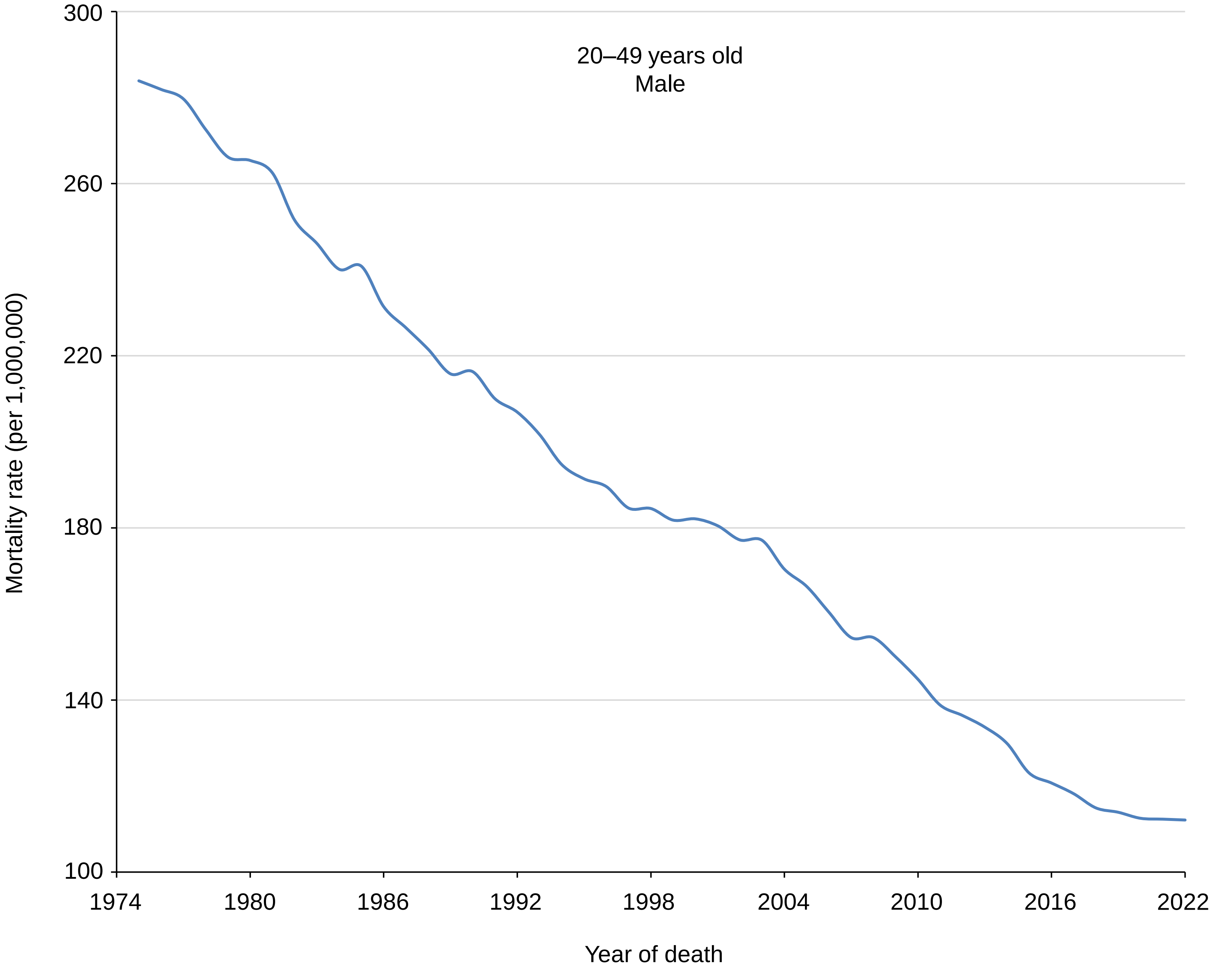

20–49 years old
Male
Mortality rate (per 1,000,000)
300
260
220
180
140
100
1974
1980
1986
1992
1998
2004
2010
2016
2022
Year of death

Figure S15. (C)

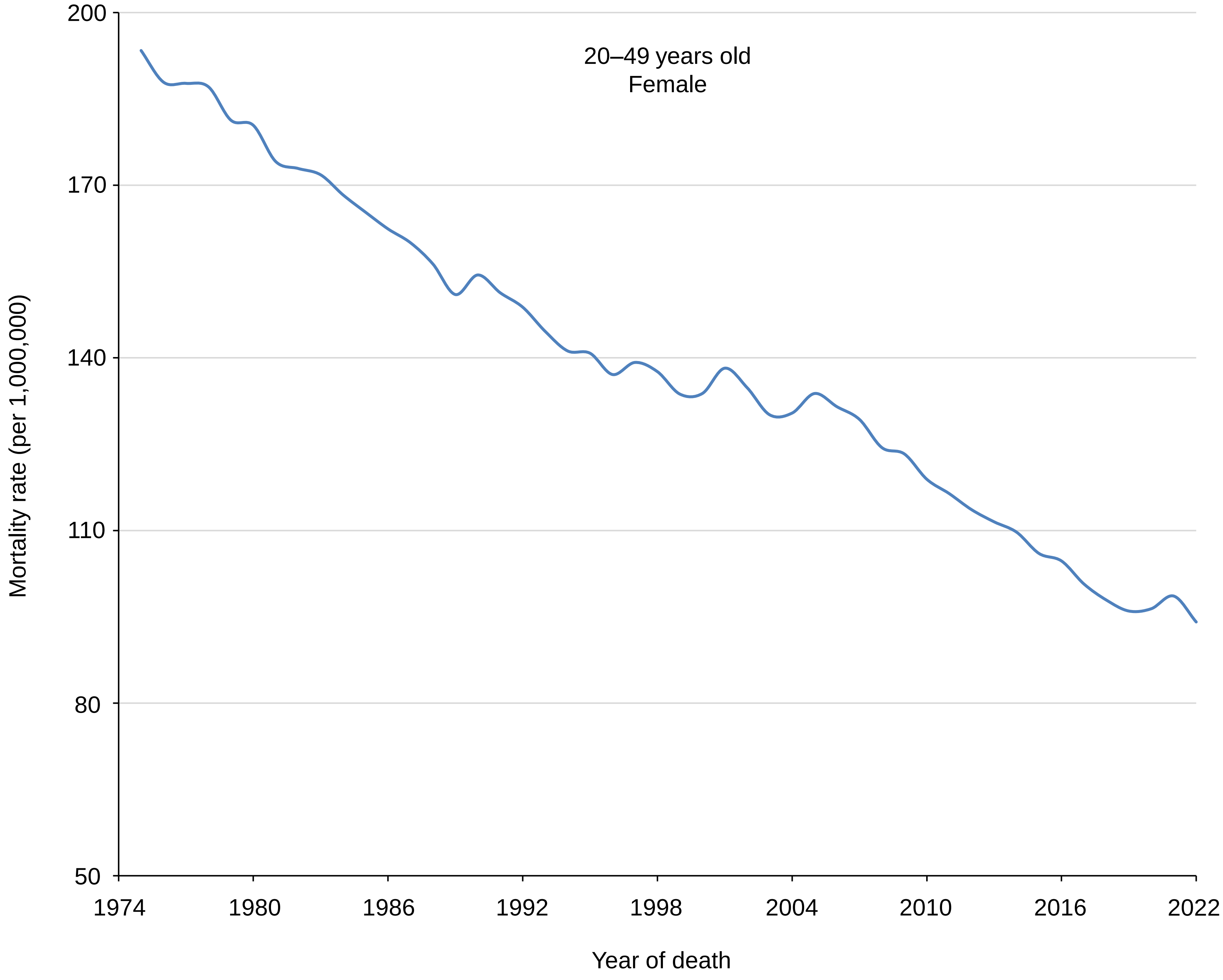

20–49 years old
Female
Mortality rate (per 1,000,000)
200
170
140
110
80
50
1974
1980
1986
1992
1998
2004
2010
2016
2022
Year of death

Figure S16. (A)

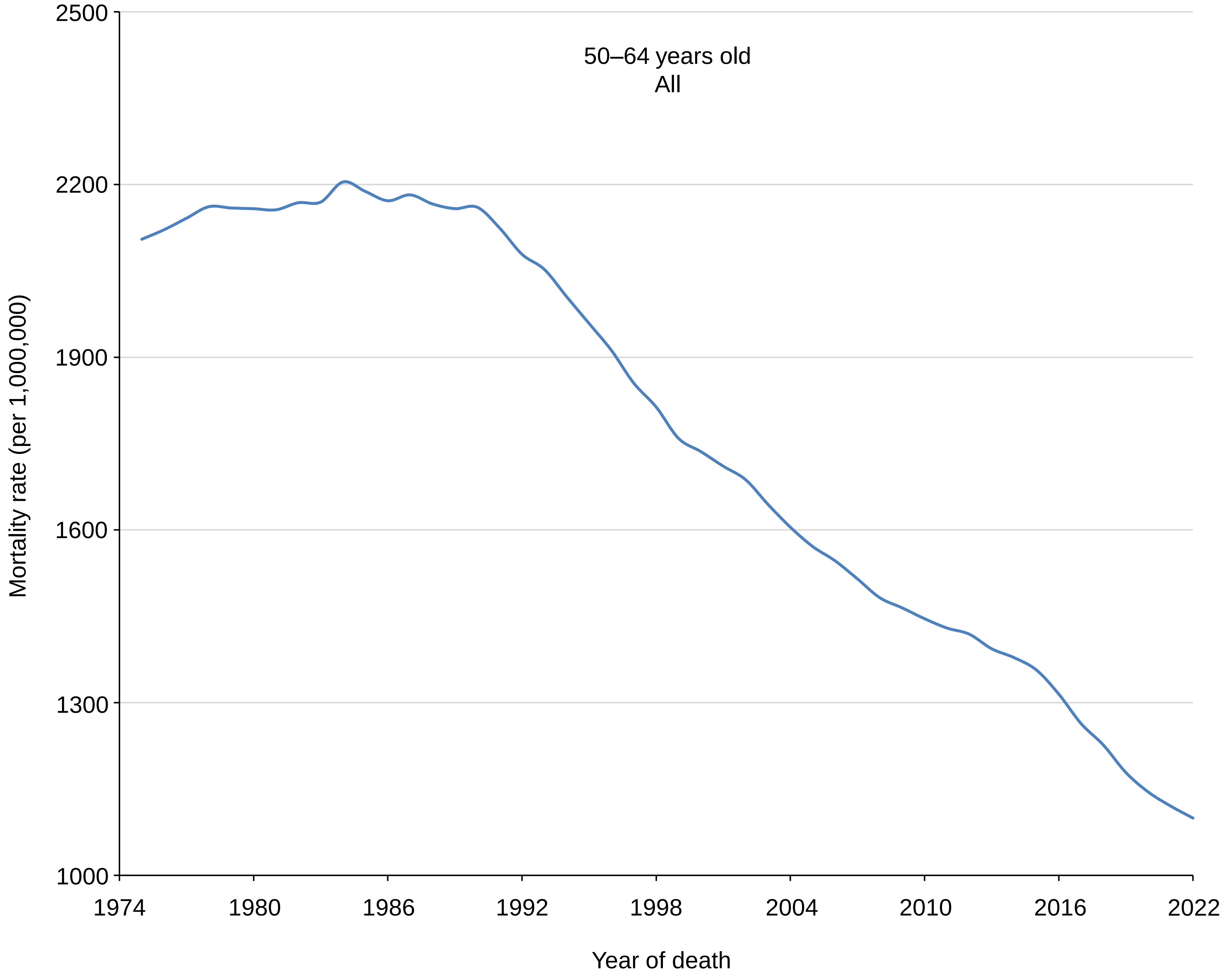

50–64 years old
All
Mortality rate (per 1,000,000)
2500
2200
1900
1600
1300
1000
1974
1980
1986
1992
1998
2004
2010
2016
2022
Year of death

Figure S16. (B)

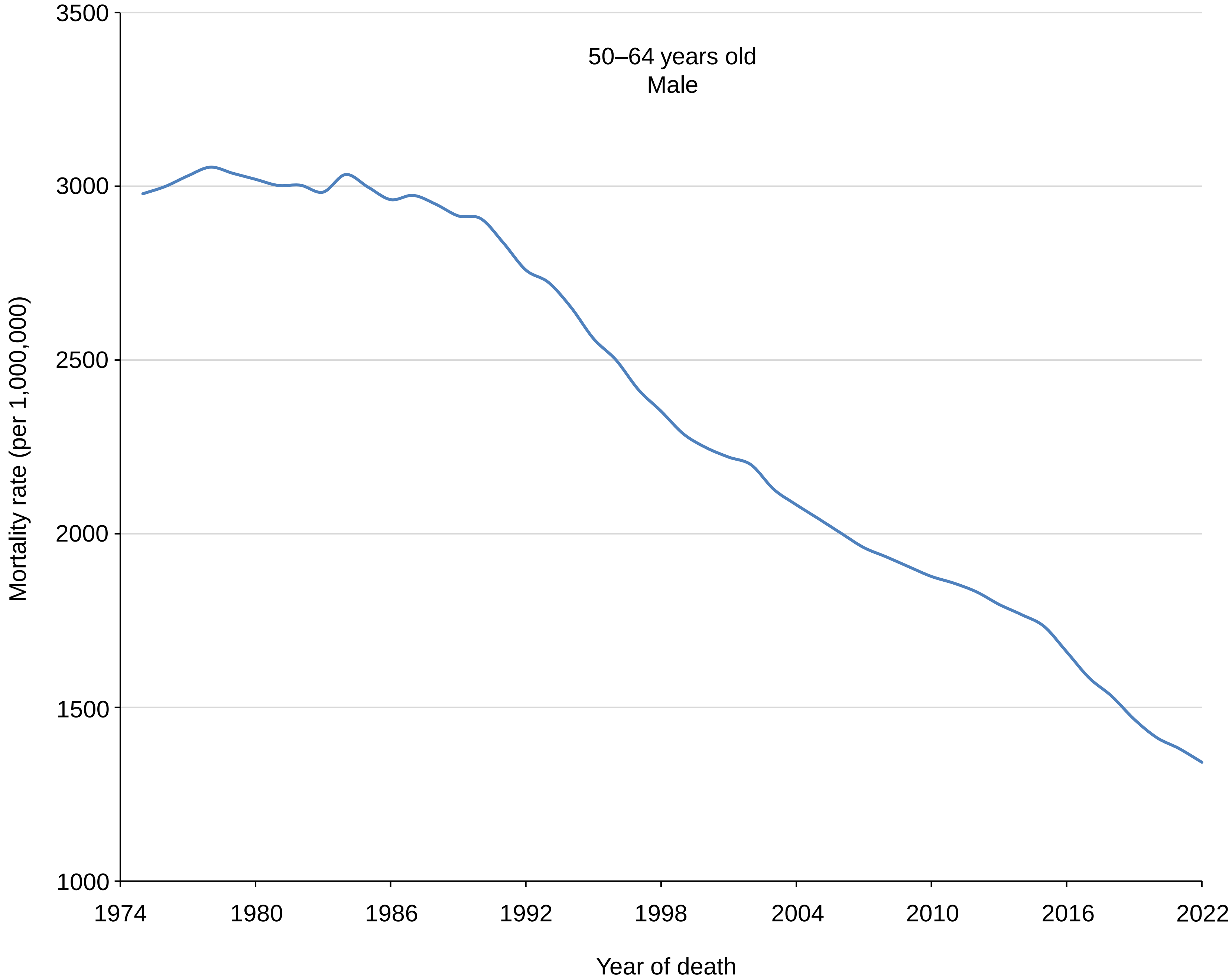

50–64 years old
Male
Mortality rate (per 1,000,000)
3500
3000
2500
2000
1500
1000
1974
1980
1986
1992
1998
2004
2010
2016
2022
Year of death

Figure S16. (C)

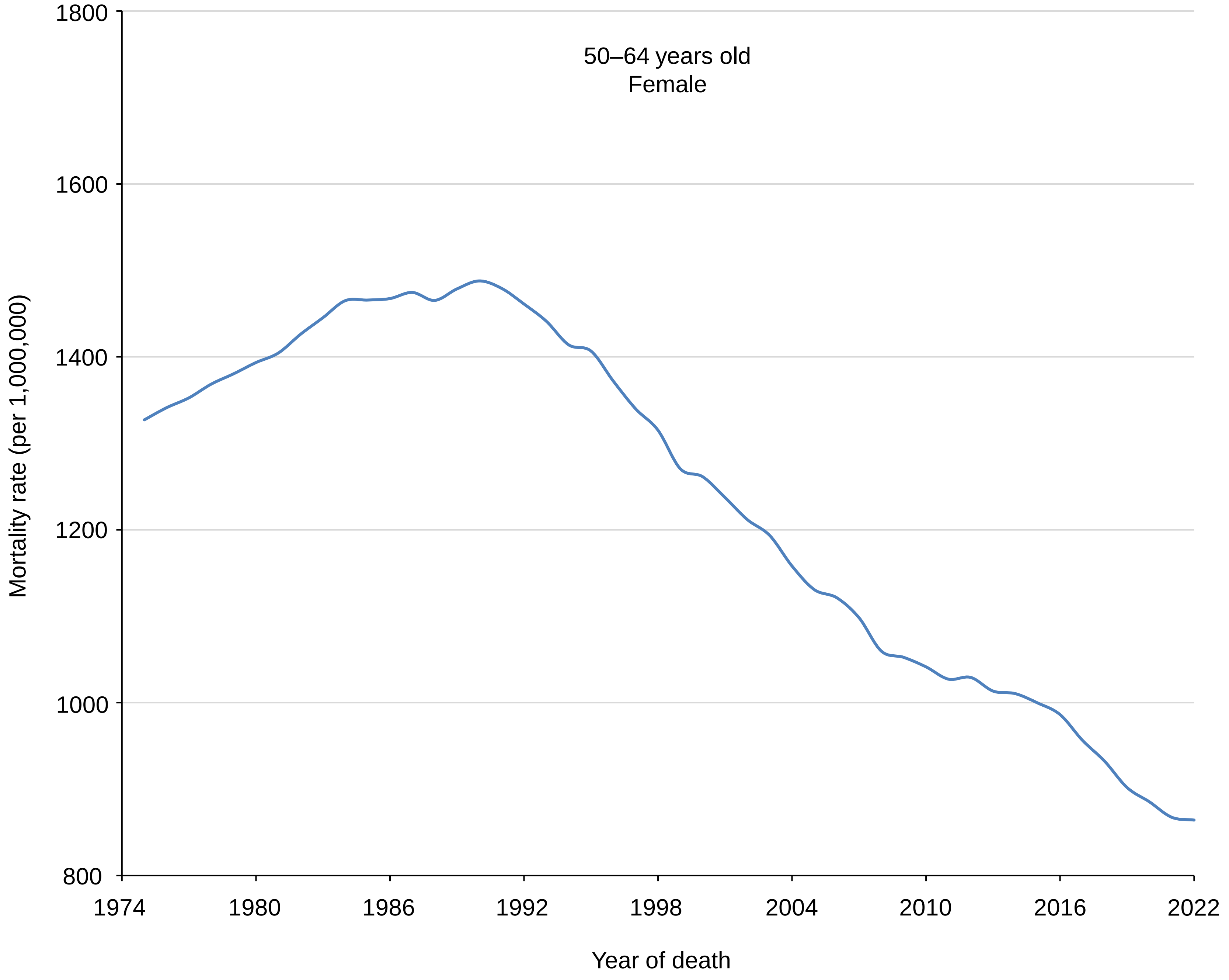

50–64 years old
Female
1800
1600
1400
1200
1000
800
Mortality rate (per 1,000,000)
1974
1980
1986
1992
1998
2004
2010
2016
2022
Year of death

Figure S17. (A)

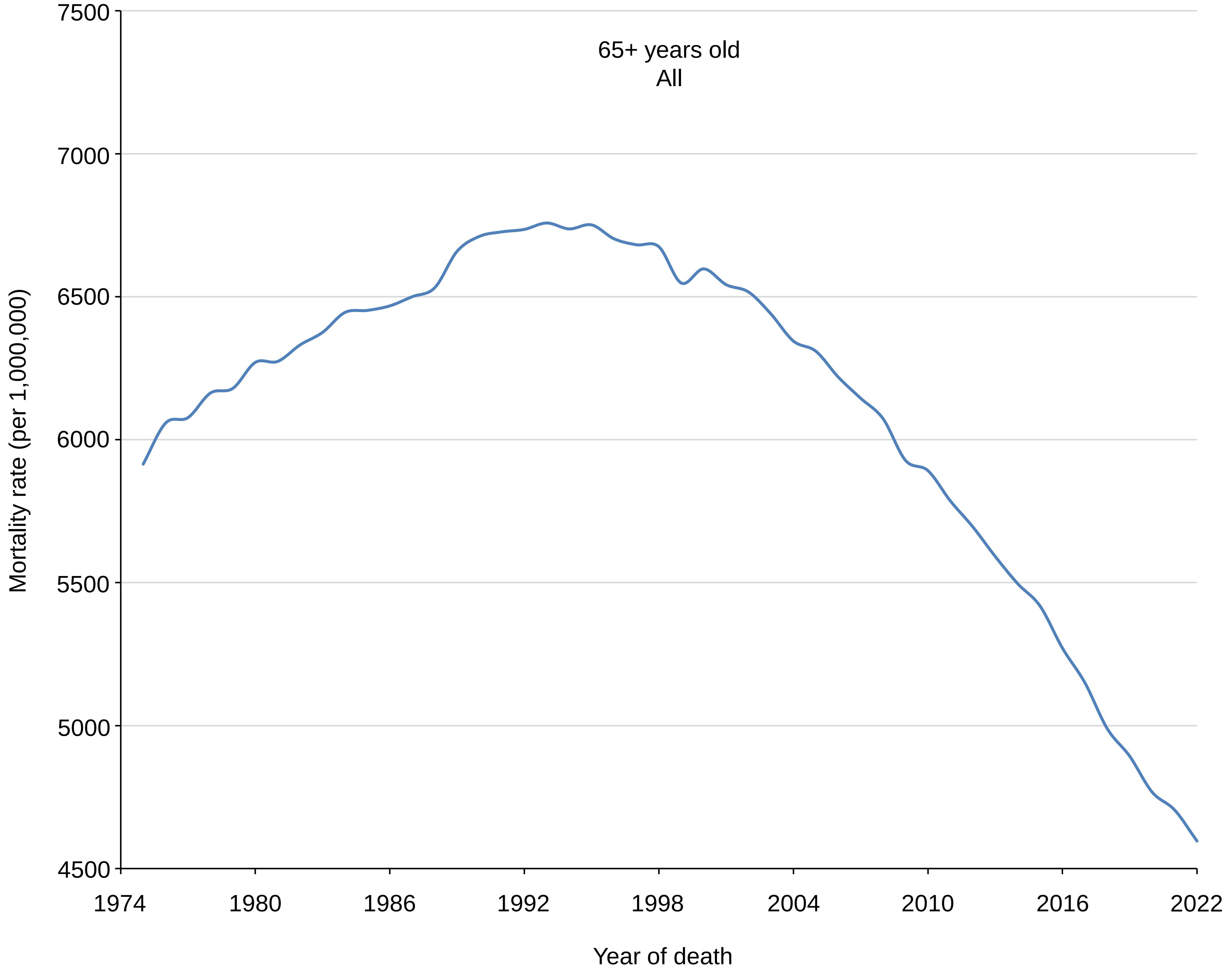

65+ years old
All
Mortality rate (per 1,000,000)
7500
7000
6500
6000
5500
5000
4500
1974
1980
1986
1992
1998
2004
2010
2016
2022
Year of death

Figure S17. (B)

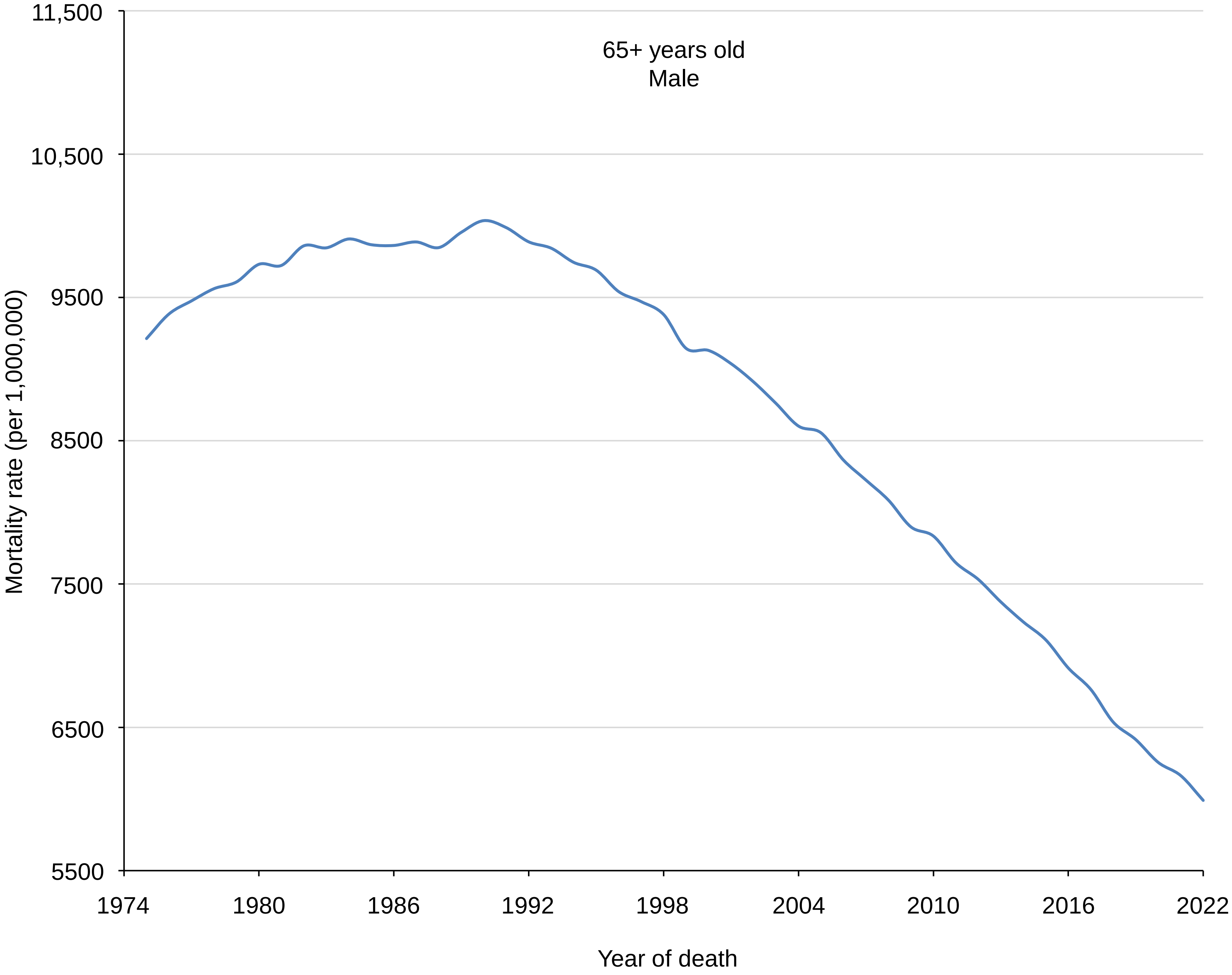

65+ years old
Male
Mortality rate (per 1,000,000)
11,500
10,500
9500
8500
7500
6500
5500
1974
1980
1986
1992
1998
2004
2010
2016
2022
Year of death

Figure S17. (C)

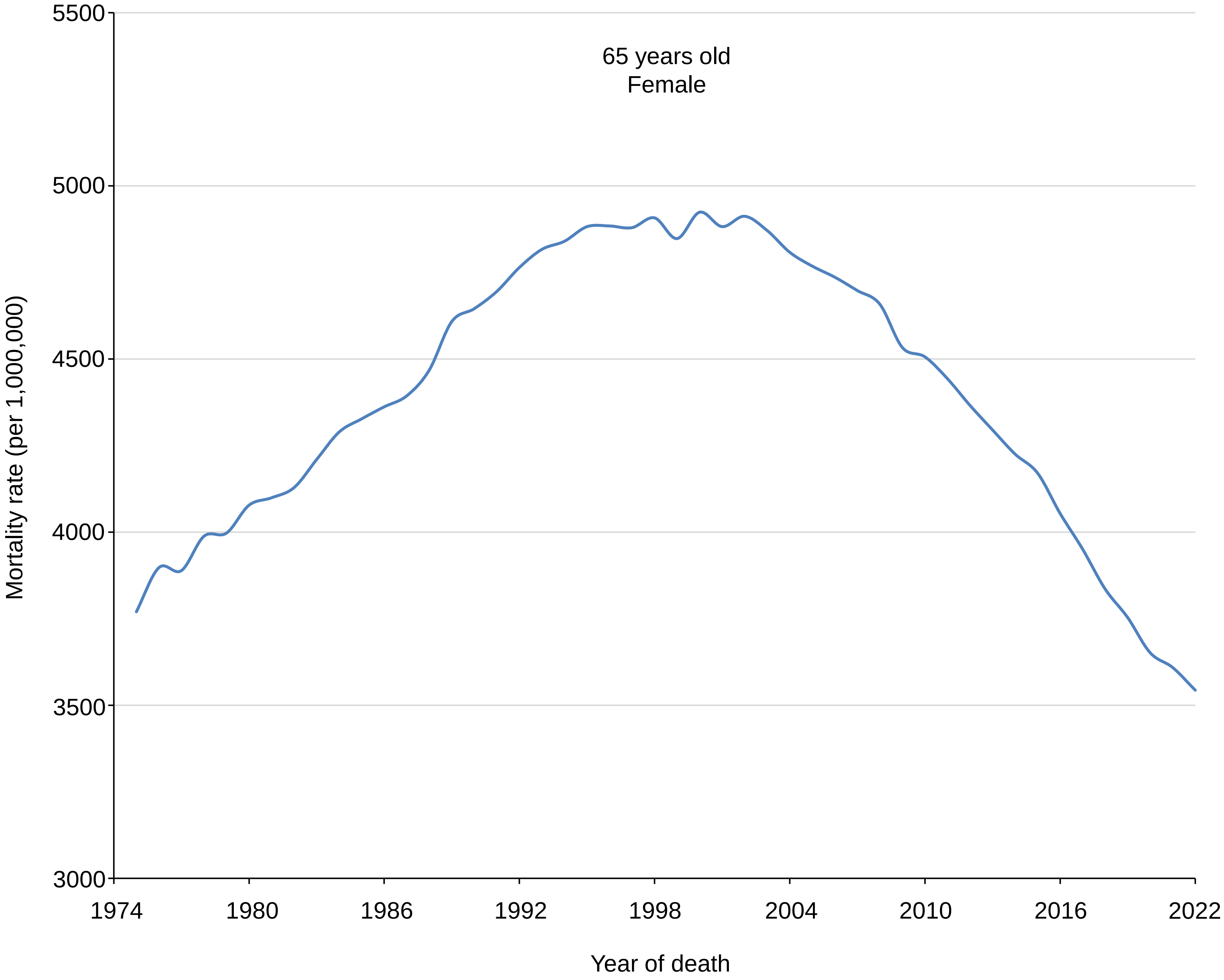
65 years old
Female
5500
5000
4500
4000
3500
3000
Mortality rate (per 1,000,000)
1974
1980
1986
1992
1998
2004
2010
2016
2022
Year of death

Figure S18. (A)

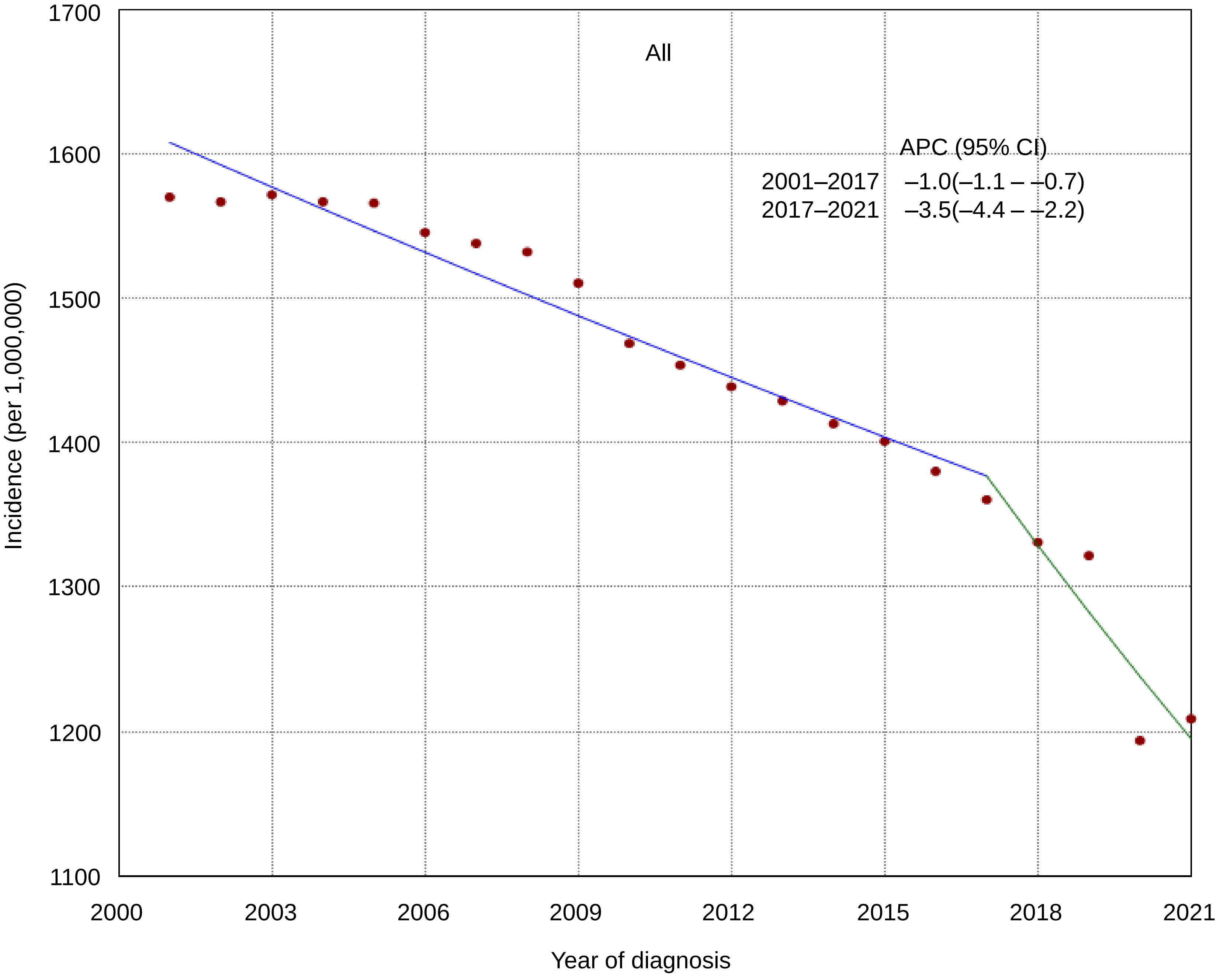

All
APC (95% CI)
2001–2017 –1.0(–1.1 – –0.7)
2017–2021 –3.5(–4.4 – –2.2)
Incidence (per 1,000,000)
1700
1600
1500
1400
1300
1200
1100
2000
2003
2006
2009
2012
2015
2018
2021
Year of diagnosis

Figure S18. (B)

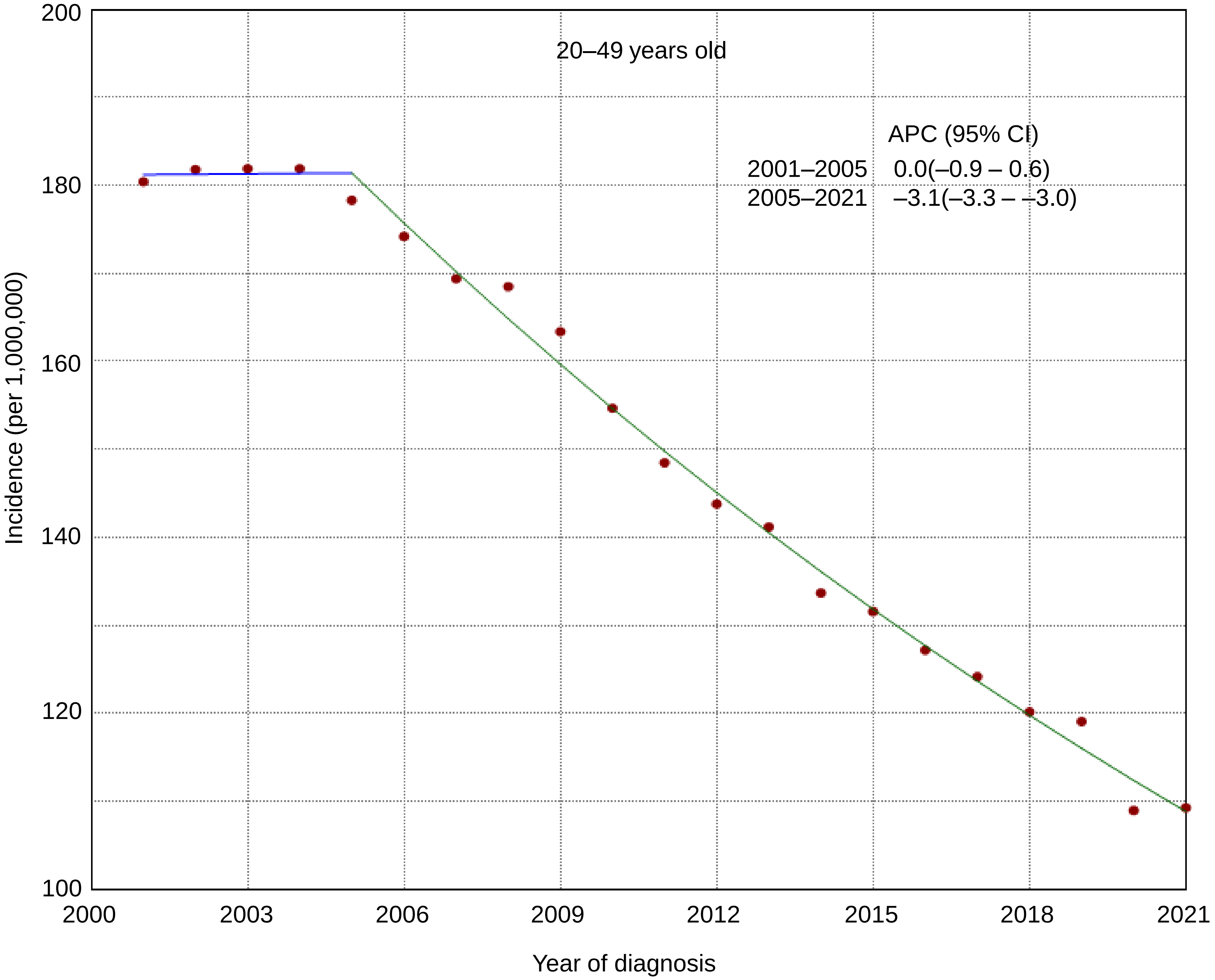

20–49 years old
APC (95% CI)
2001–2005 0.0(–0.9 – 0.6)
2005–2021 –3.1(–3.3 – –3.0)
Incidence (per 1,000,000)
Year of diagnosis
200
180
160
140
120
100
2000
2003
2006
2009
2012
2015
2018
2021

Figure S18. (C)

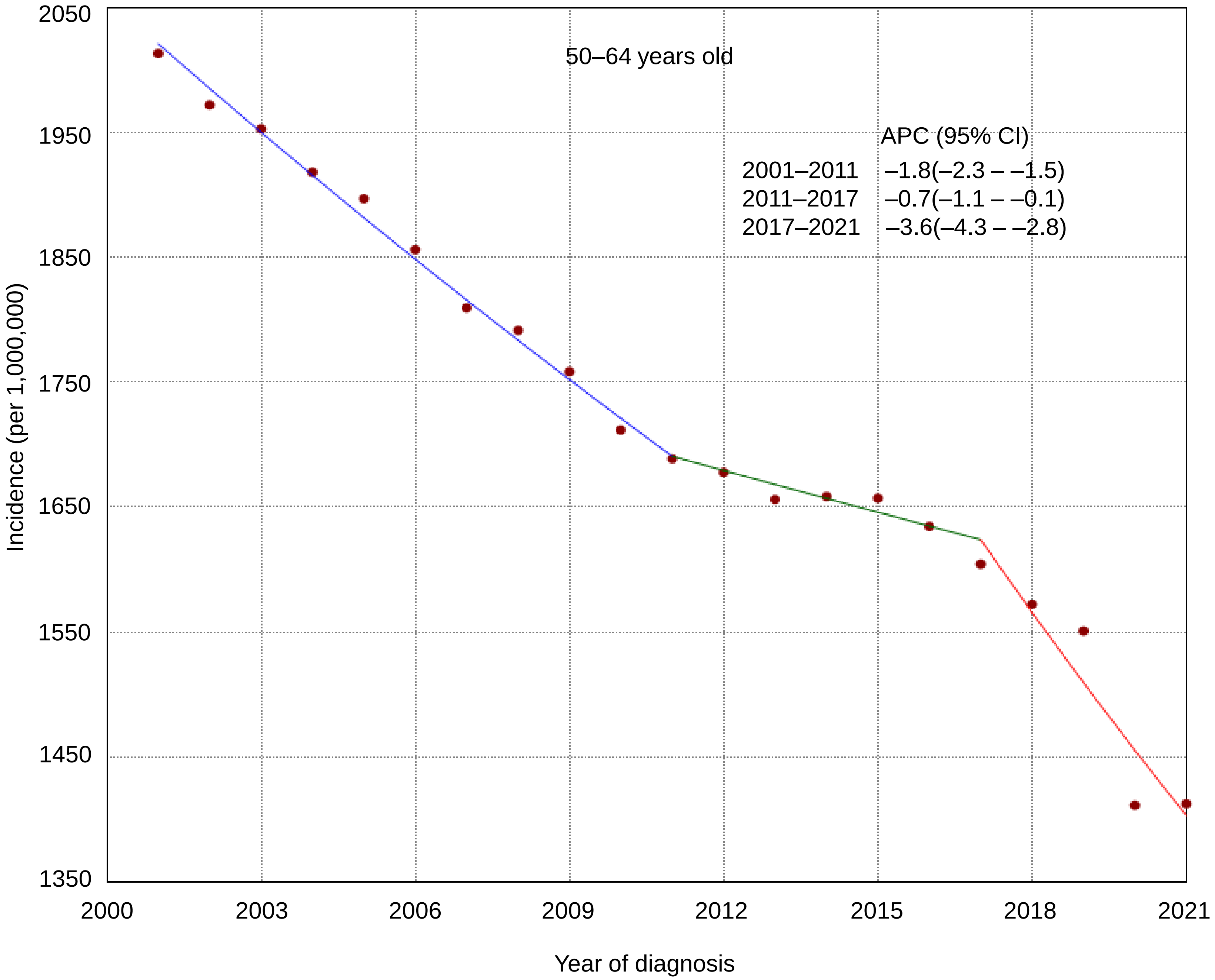

50–64 years old
APC (95% CI)
2001–2011 –1.8(–2.3 – –1.5)
2011–2017 –0.7(–1.1 – –0.1)
2017–2021 –3.6(–4.3 – –2.8)
Incidence (per 1,000,000)
2050
1950
1850
1750
1650
1550
1450
1350
2000
2003
2006
2009
2012
2015
2018
2021
Year of diagnosis

Figure S18. (D)

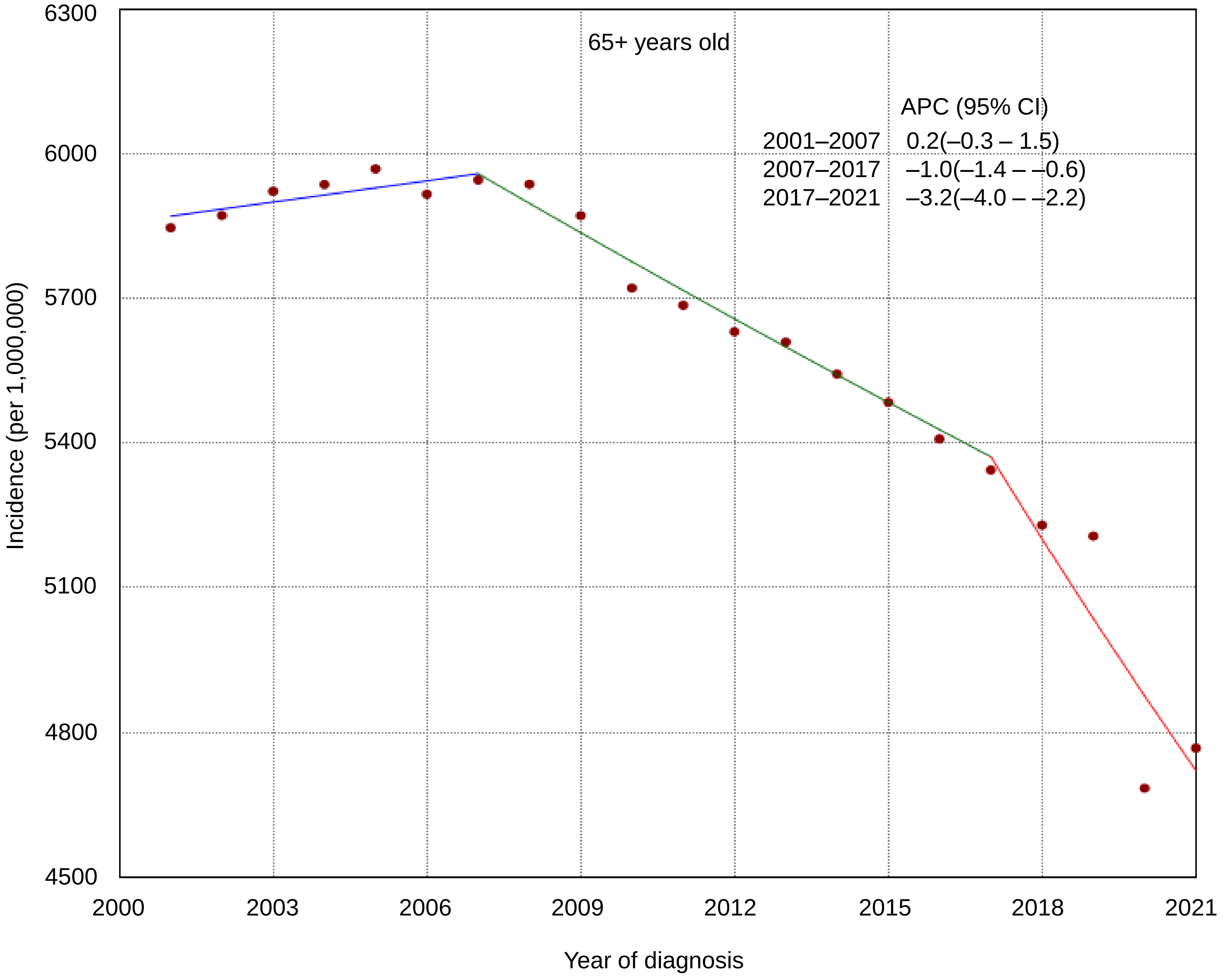

65+ years old
APC (95% CI)
2001–2007 0.2(–0.3 – 1.5)
2007–2017 –1.0(–1.4 – –0.6)
2017–2021 –3.2(–4.0 – –2.2)
Incidence (per 1,000,000)
6300
6000
5700
5400
5100
4800
4500
2000
2003
2006
2009
2012
2015
2018
2021
Year of diagnosis

Figure S19. (A)

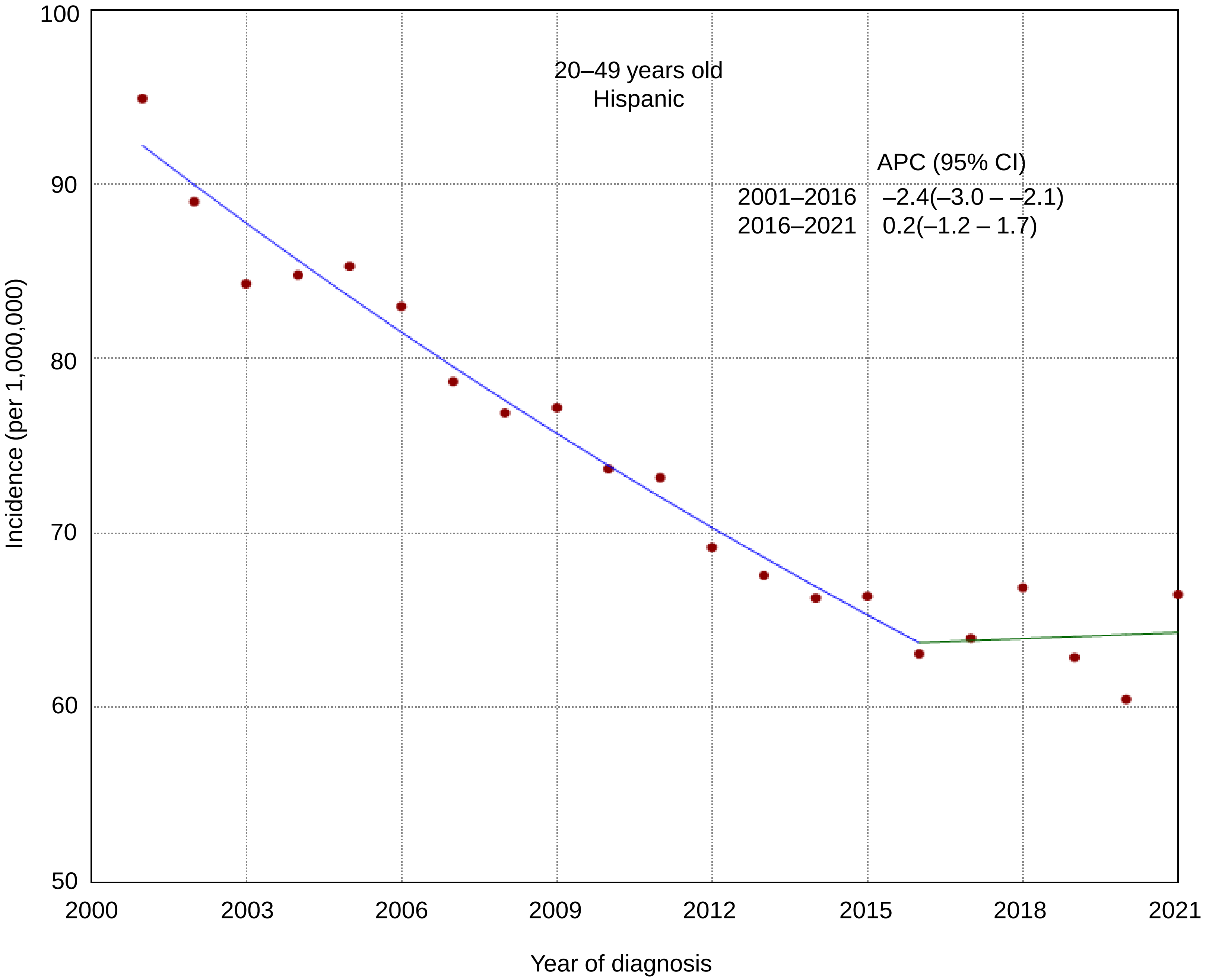
20–49 years old
Hispanic
APC (95% CI)
2001–2016 –2.4(–3.0 – –2.1)
2016–2021 0.2(–1.2 – 1.7)
Incidence (per 1,000,000)
100
90
80
70
60
50
2000
2003
2006
2009
2012
2015
2018
2021
Year of diagnosis

Figure S19. (B)

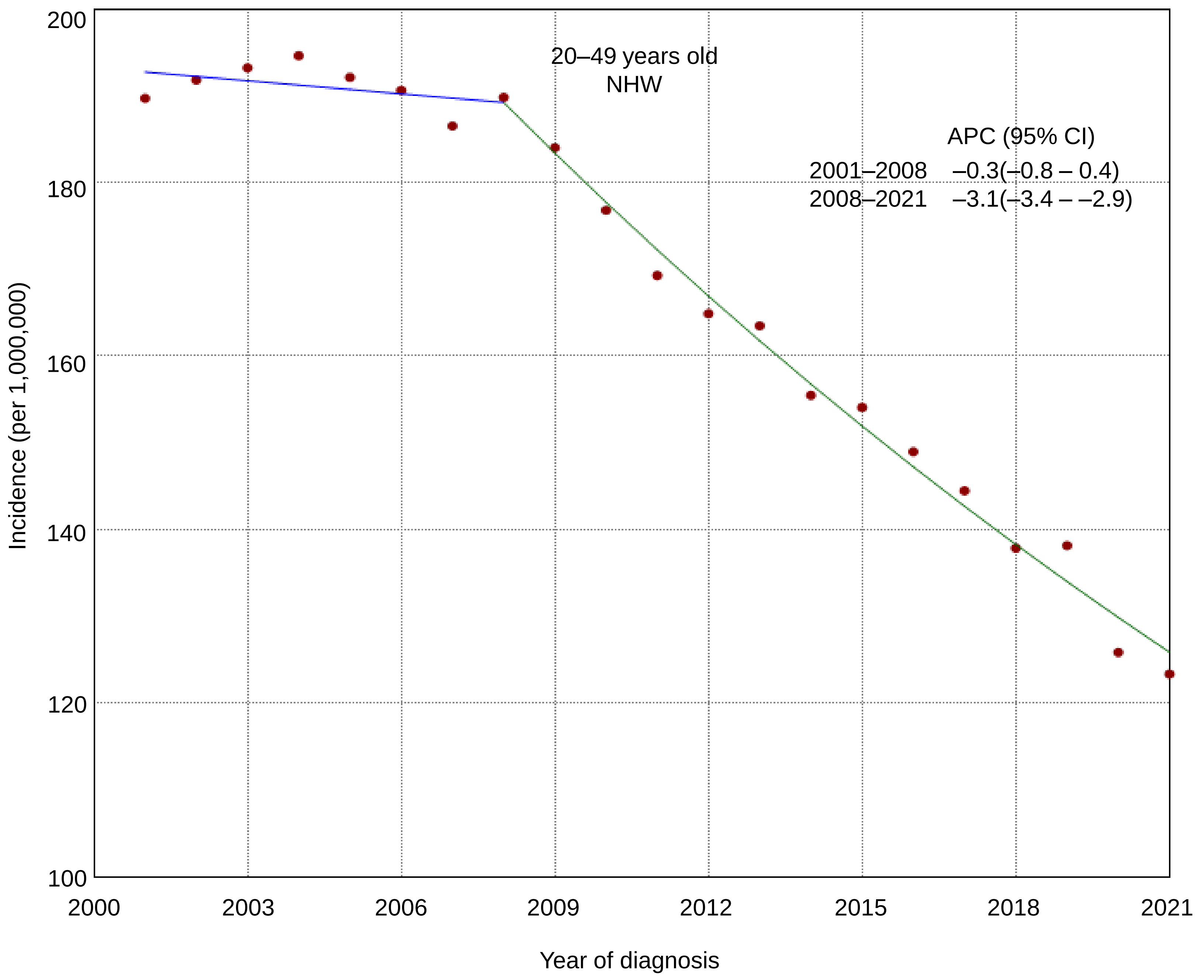
20–49 years old
NHW
APC (95% CI)
2001–2008 −0.3(−0.8 – 0.4)
2008–2021 −3.1(−3.4 – −2.9)
Incidence (per 1,000,000)
200
180
160
140
120
100
2000
2003
2006
2009
2012
2015
2018
2021
Year of diagnosis

Figure S19. (C)

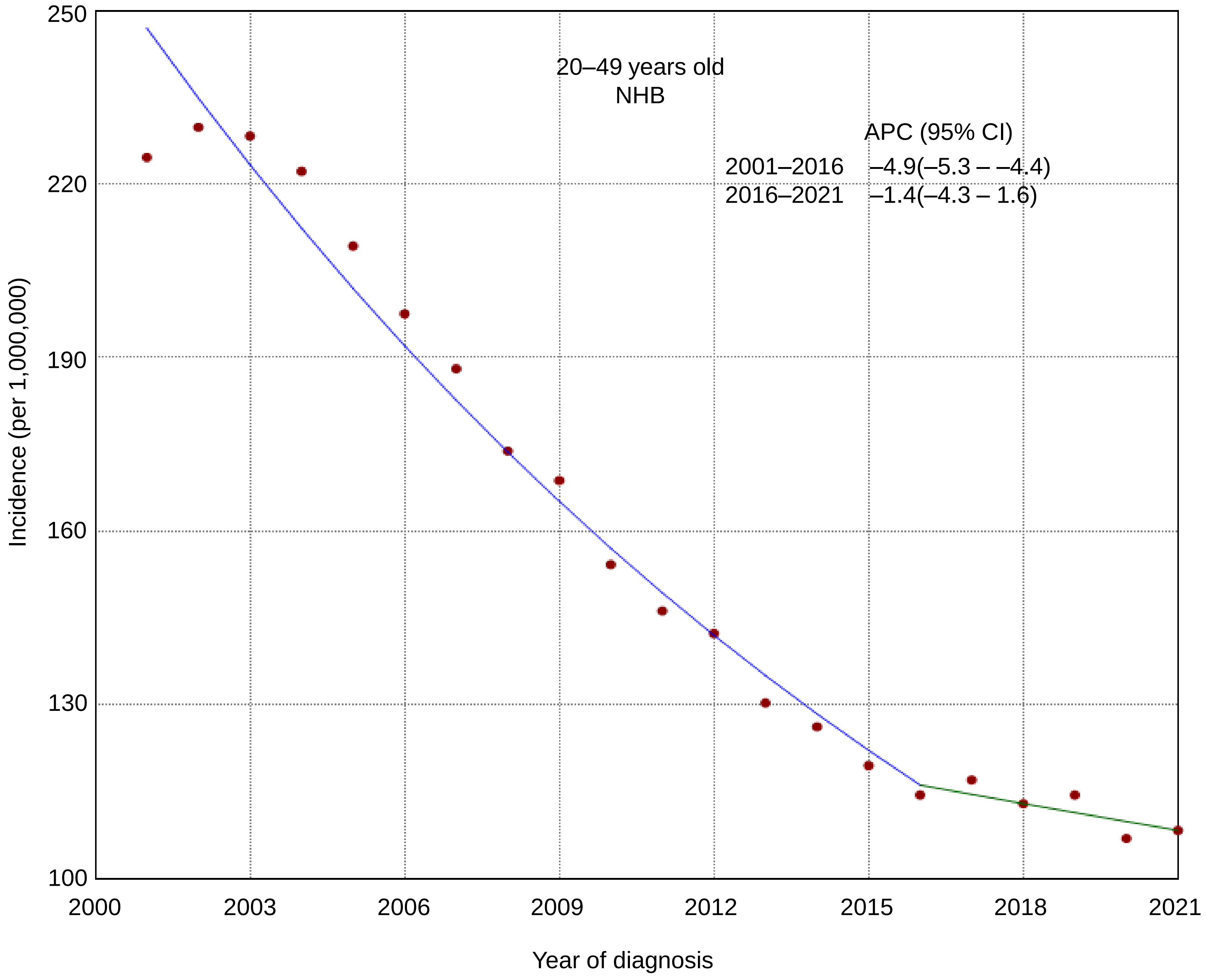
20–49 years old
NHB
APC (95% CI)
2001–2016 –4.9(–5.3 – –4.4)
2016–2021 –1.4(–4.3 – 1.6)
Incidence (per 1,000,000)
250
220
190
160
130
100
2000
2003
2006
2009
2012
2015
2018
2021
Year of diagnosis

Figure S19. (D)

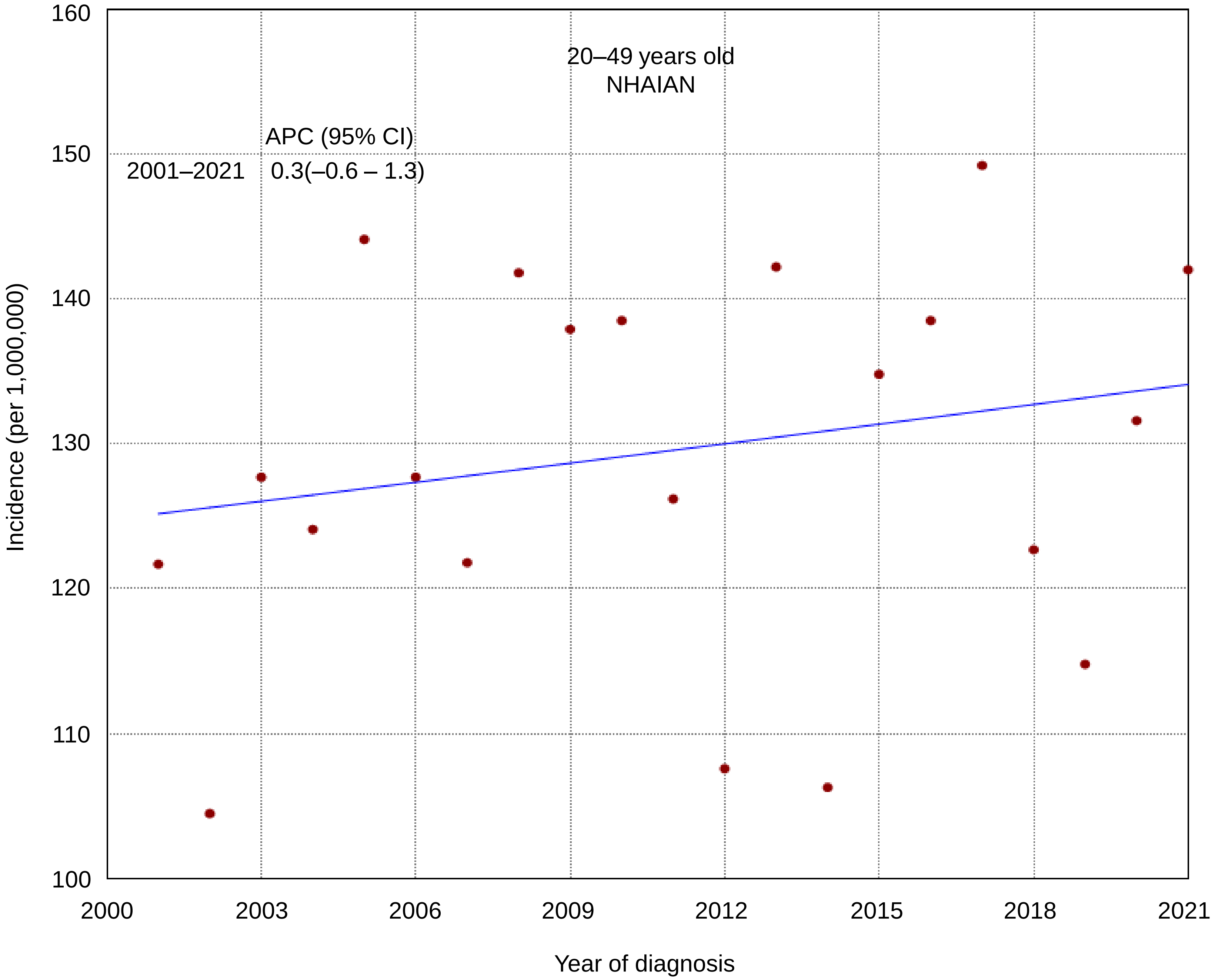
20–49 years old
NHAIAN
APC (95% CI)
2001–2021
0.3(–0.6 – 1.3)
Incidence (per 1,000,000)
160
150
140
130
120
110
100
2000
2003
2006
2009
2012
2015
2018
2021
Year of diagnosis

Figure S19. (E)

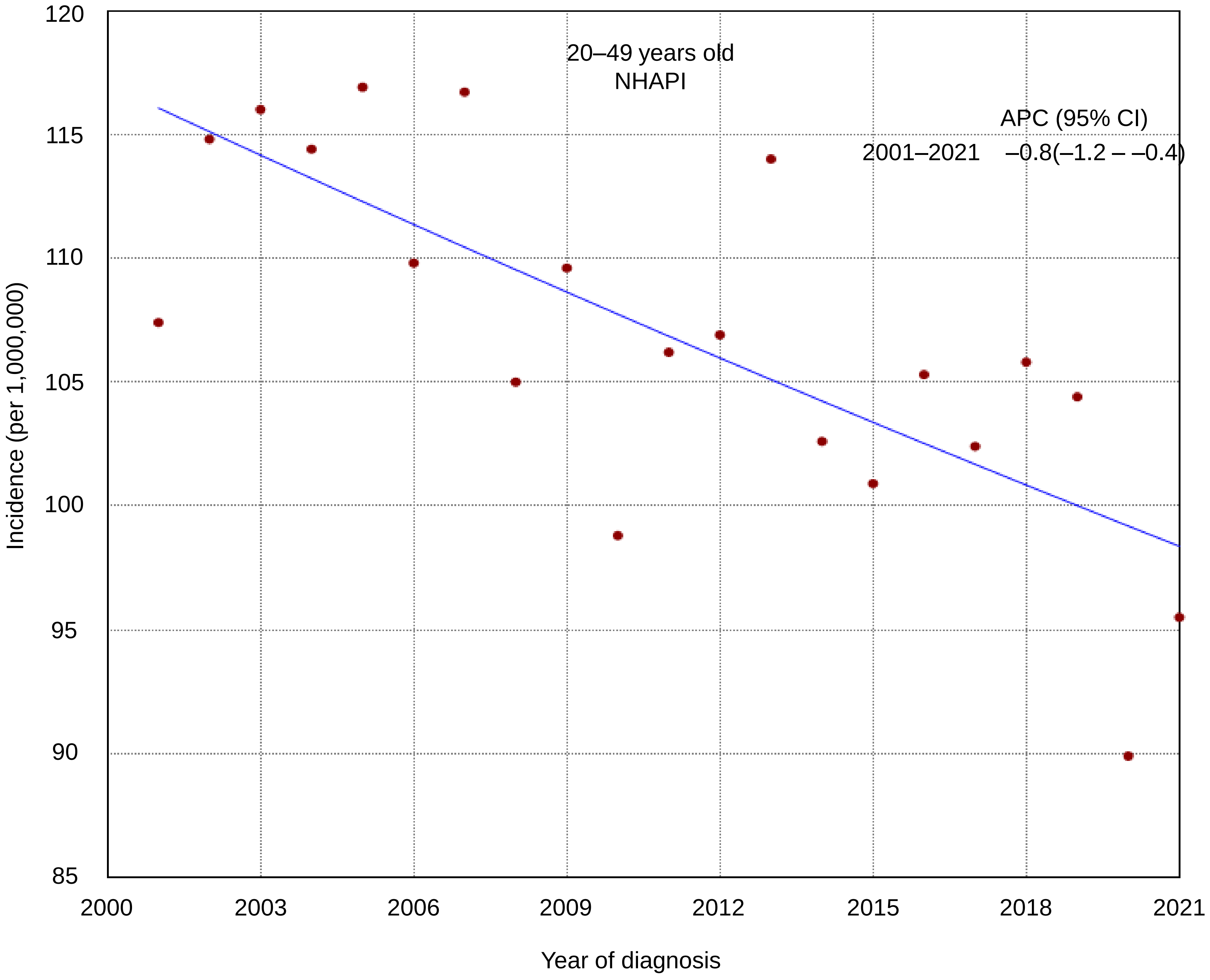
20–49 years old
NHAPI
APC (95% CI)
2001–2021 –0.8(–1.2 – –0.4)
Incidence (per 1,000,000)
120
115
110
105
100
95
90
85
2000
2003
2006
2009
2012
2015
2018
2021
Year of diagnosis

Figure S20. (A)

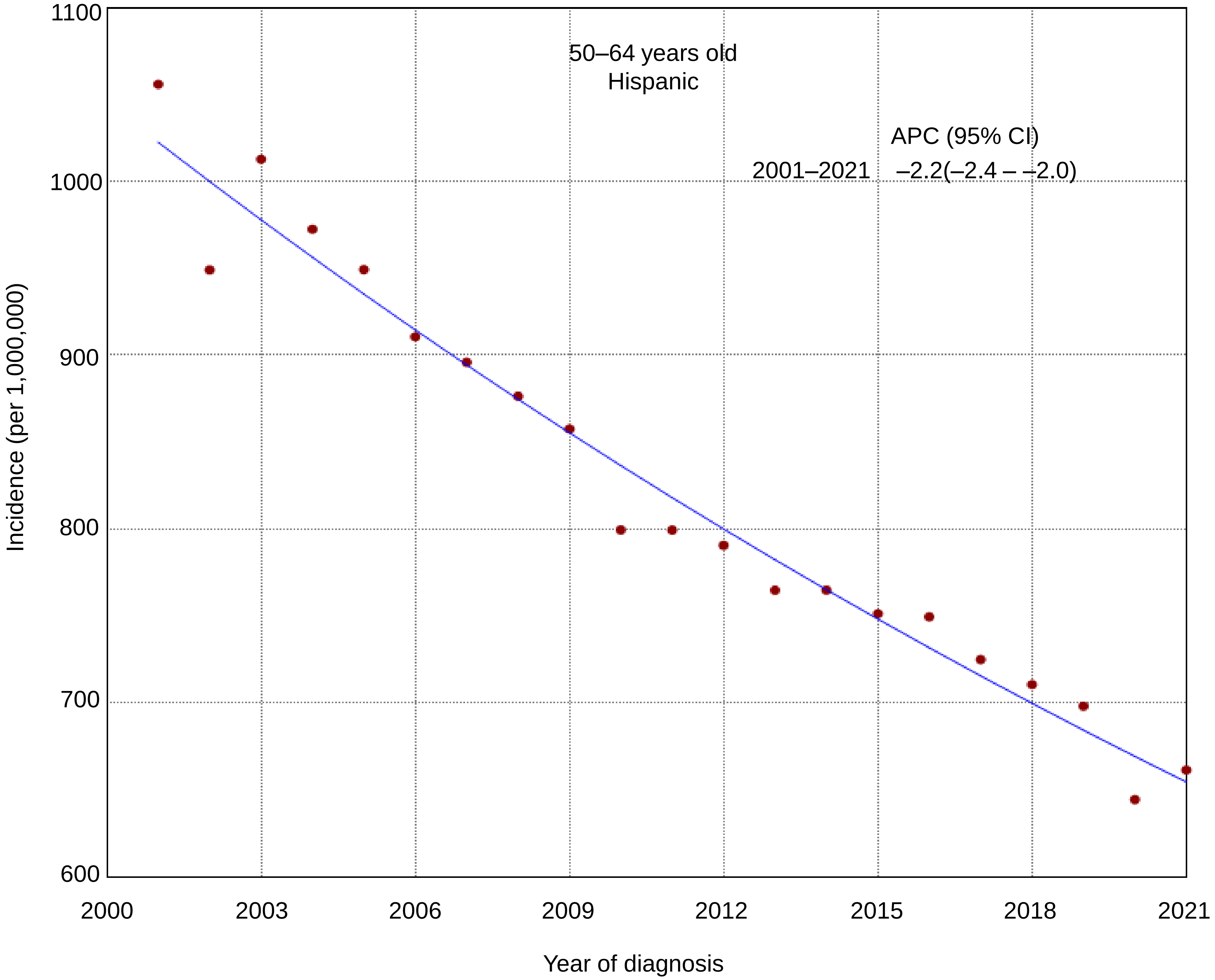
50–64 years old
Hispanic
APC (95% CI)
2001–2021 –2.2(–2.4 – –2.0)
Incidence (per 1,000,000)
1100
1000
900
800
700
600
2000
2003
2006
2009
2012
2015
2018
2021
Year of diagnosis

Figure S20. (B)

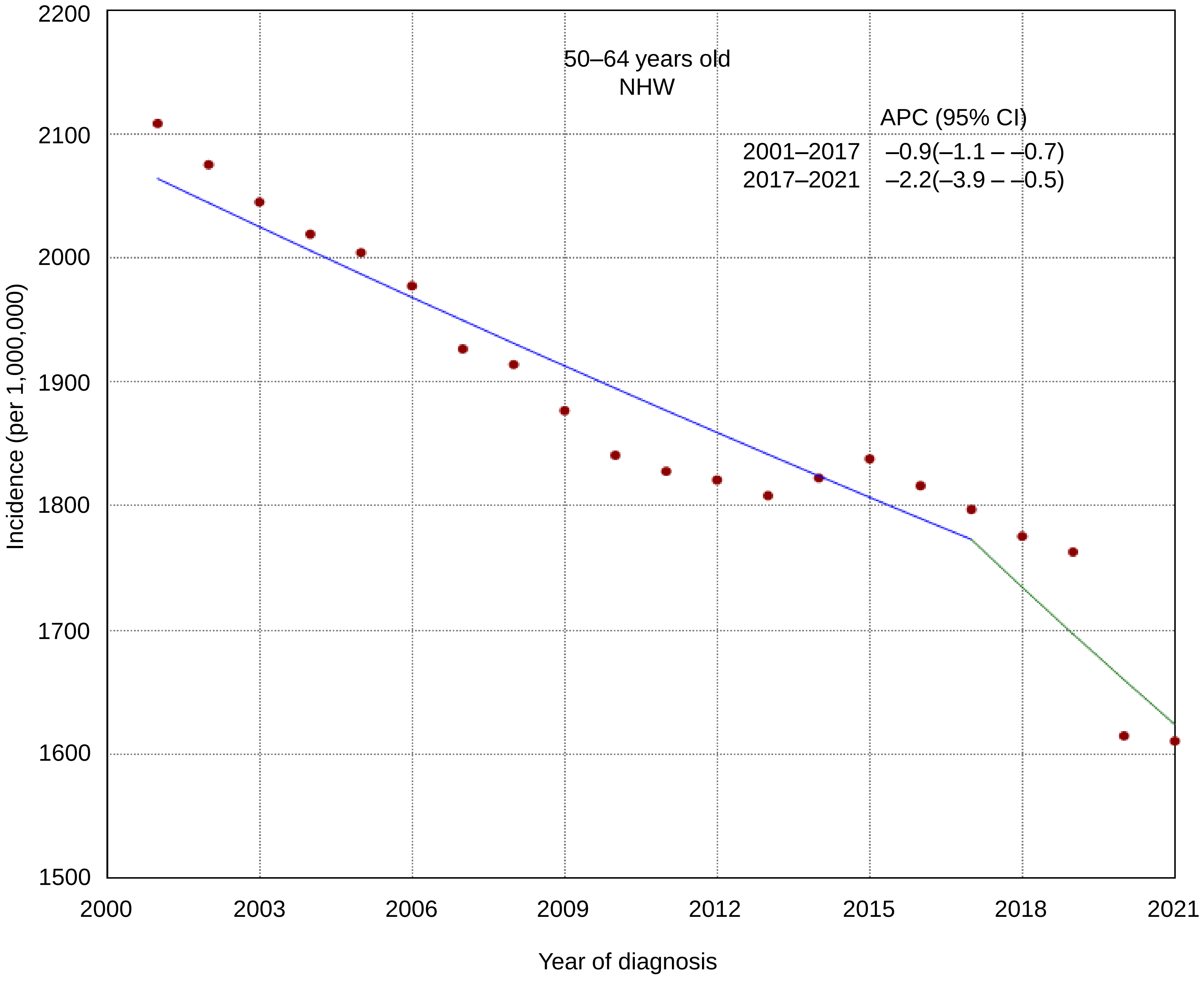

50–64 years old
NHW
APC (95% CI)
2001–2017 –0.9(–1.1 – –0.7)
2017–2021 –2.2(–3.9 – –0.5)
Incidence (per 1,000,000)
2200
2100
2000
1900
1800
1700
1600
1500
2000
2003
2006
2009
2012
2015
2018
2021
Year of diagnosis

Figure S20. (C)

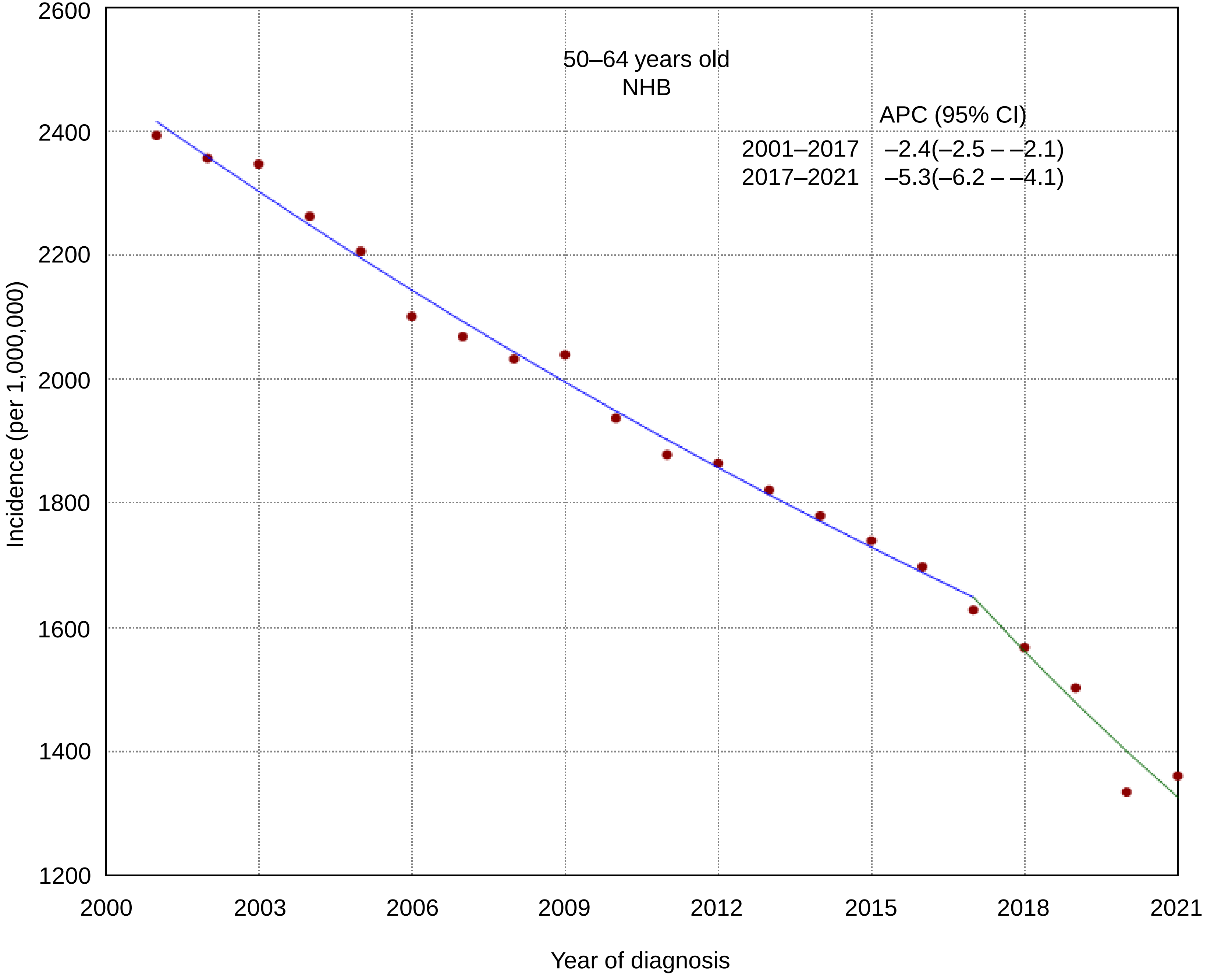
50–64 years old
NHB
APC (95% CI)
2001–2017 –2.4(–2.5 – –2.1)
2017–2021 –5.3(–6.2 – –4.1)
Incidence (per 1,000,000)
Year of diagnosis
2600
2400
2200
2000
1800
1600
1400
1200
2000
2003
2006
2009
2012
2015
2018
2021

Figure S20. (D)

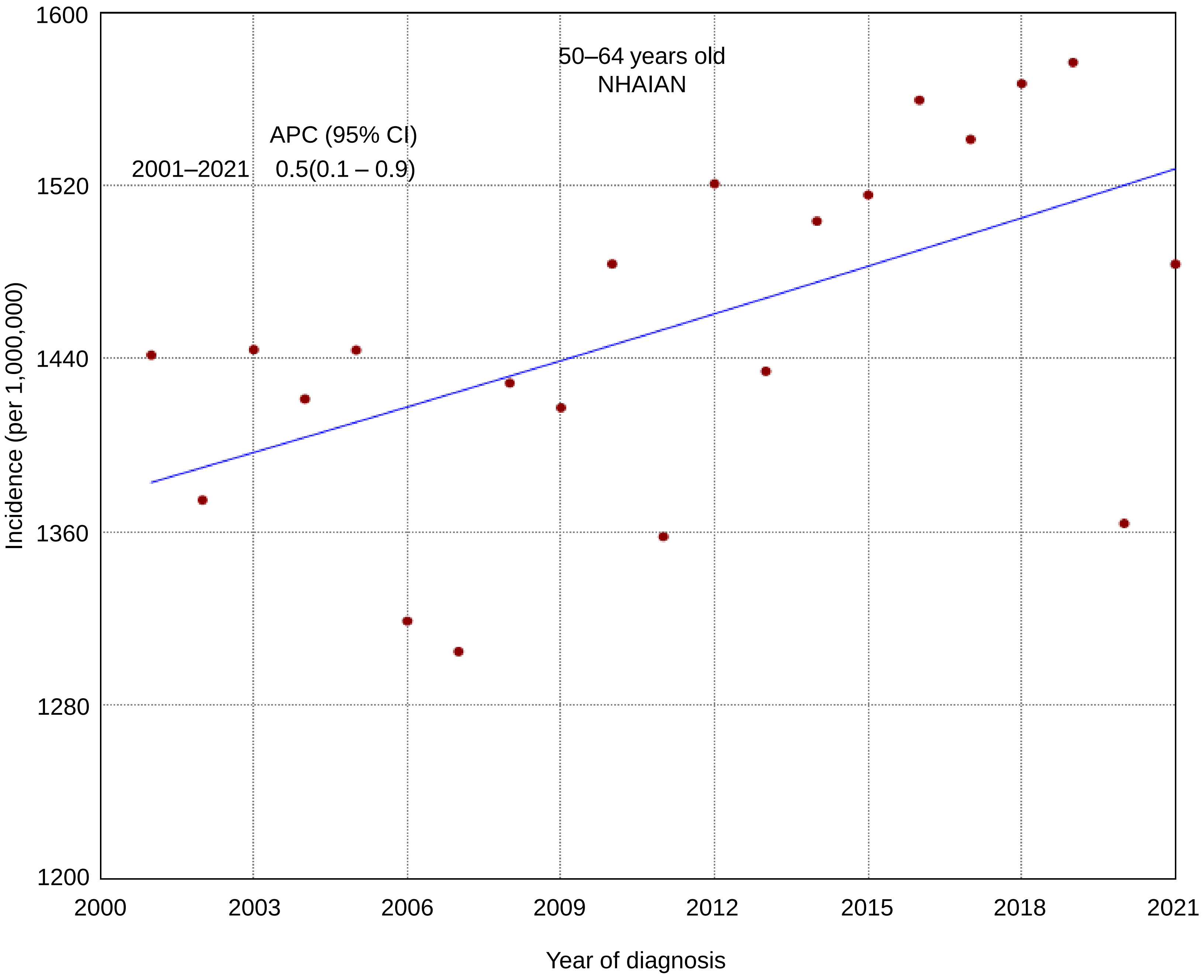

50–64 years old
NHAIAN
APC (95% CI)
2001–2021 0.5(0.1 – 0.9)
Incidence (per 1,000,000)
1600
1520
1440
1360
1280
1200
2000
2003
2006
2009
2012
2015
2018
2021
Year of diagnosis

Figure S20. (E)

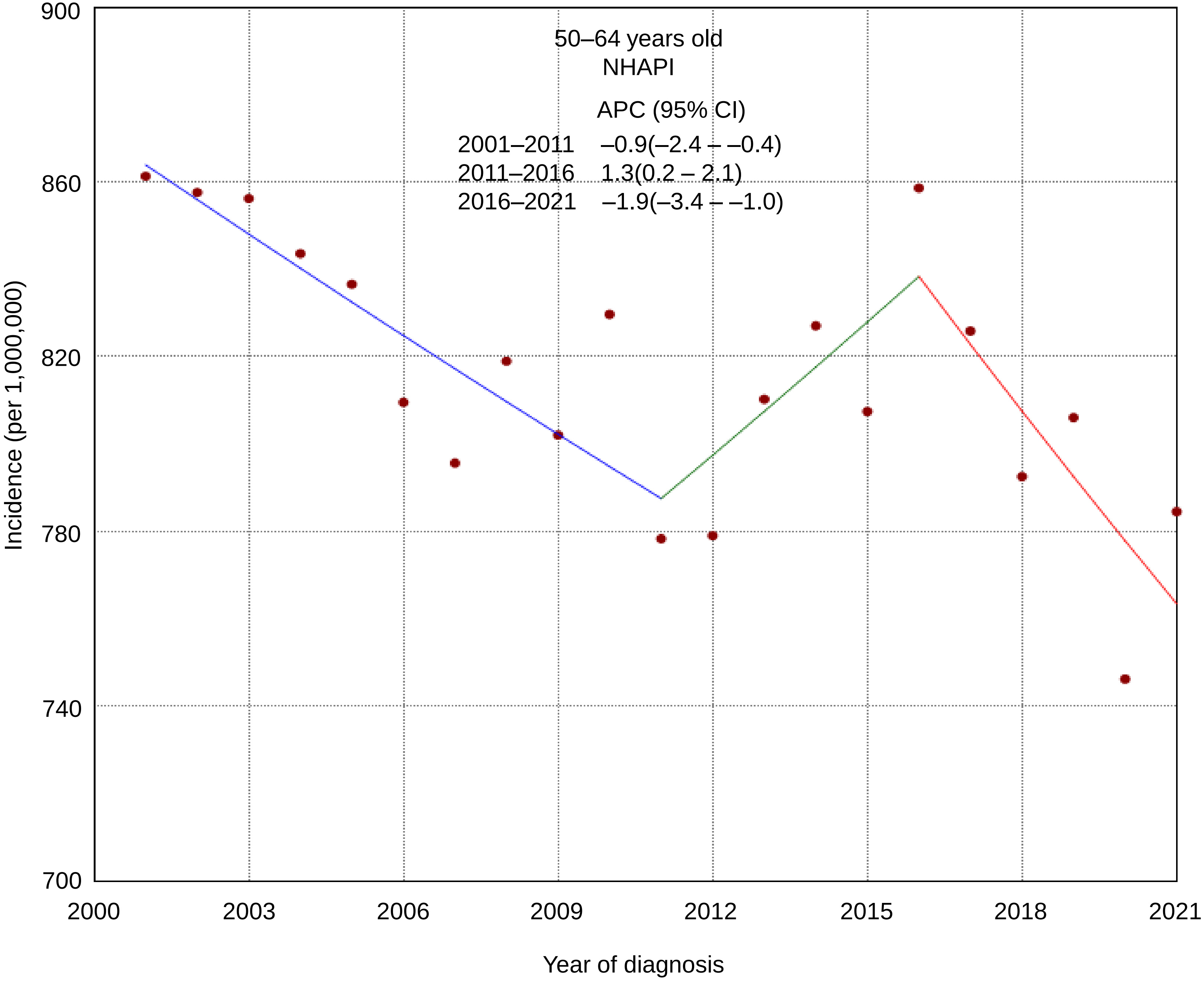

50–64 years old
NHAPI
APC (95% CI)
2001–2011 –0.9(–2.4 – –0.4)
2011–2016 1.3(0.2 – 2.1)
2016–2021 –1.9(–3.4 – –1.0)
Incidence (per 1,000,000)
900
860
820
780
740
700
2000
2003
2006
2009
2012
2015
2018
2021
Year of diagnosis

Figure S21. (A)

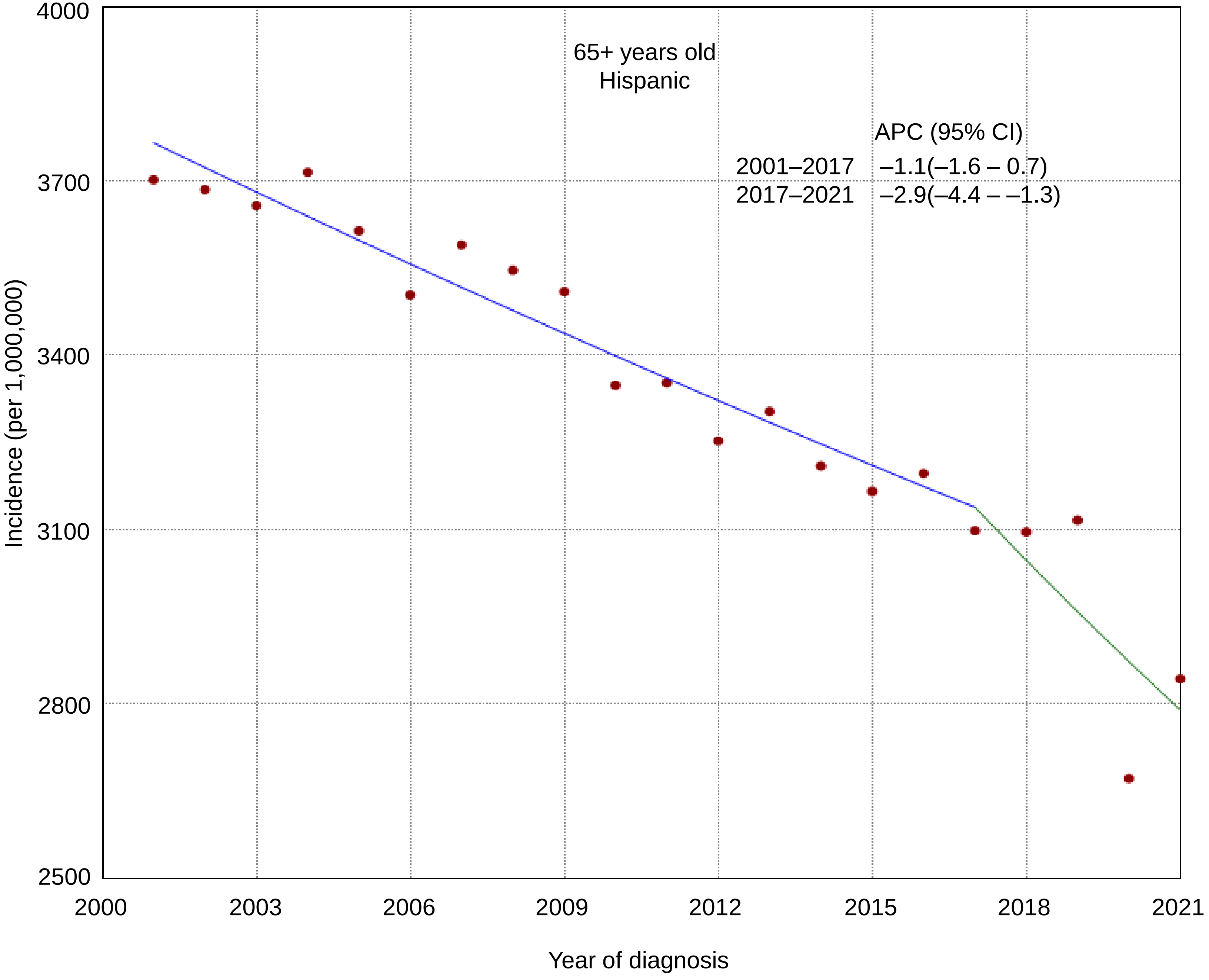
65+ years old
Hispanic
APC (95% CI)
2001–2017 –1.1(–1.6 – 0.7)
2017–2021 –2.9(–4.4 – –1.3)
4000
3700
3400
3100
2800
2500
Incidence (per 1,000,000)
2000
2003
2006
2009
2012
2015
2018
2021
Year of diagnosis

Figure S21. (B)

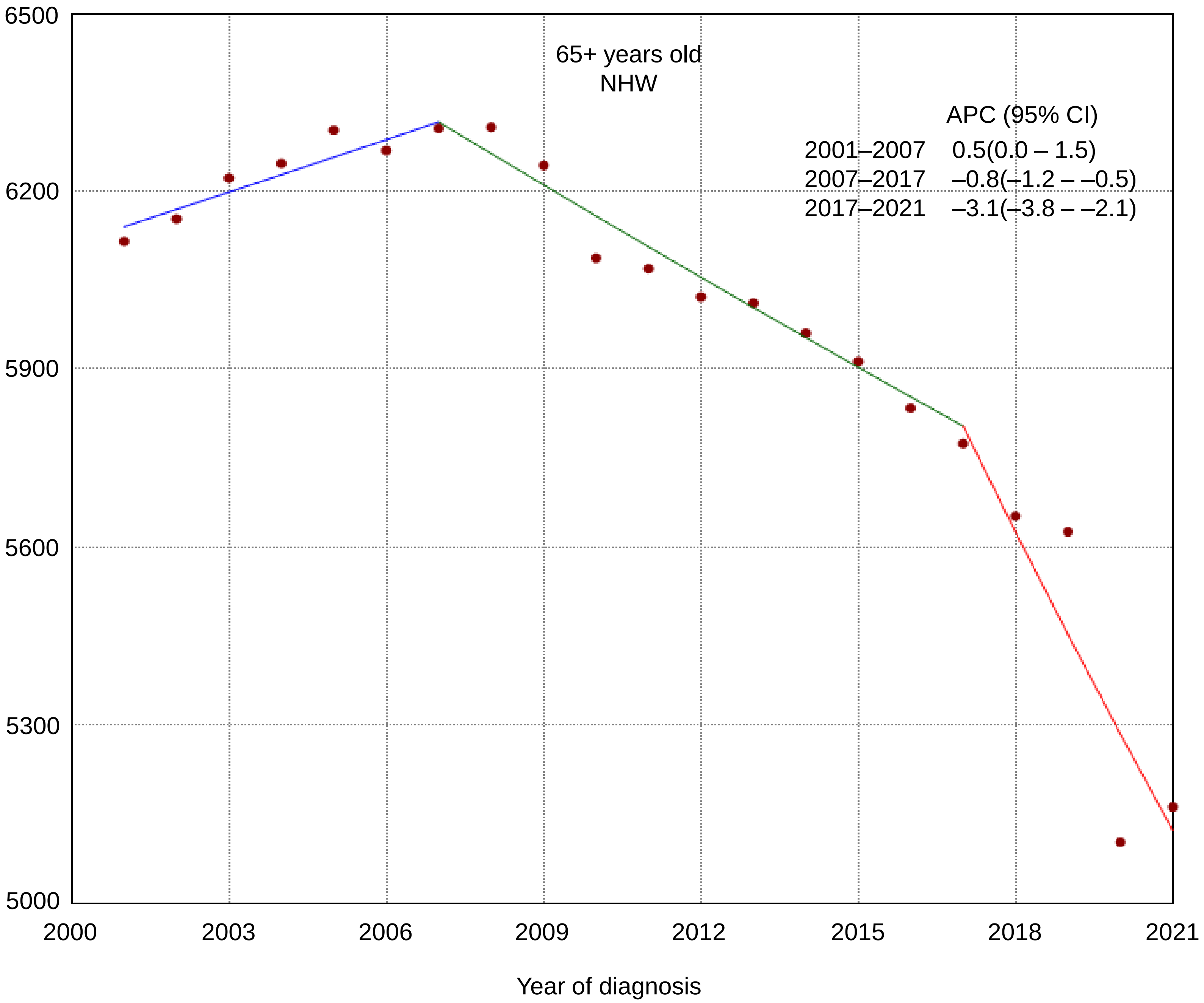
65+ years old
NHW
APC (95% CI)
2001–2007 0.5(0.0 – 1.5)
2007–2017 –0.8(–1.2 – –0.5)
2017–2021 –3.1(–3.8 – –2.1)
6500
6200
5900
5600
5300
5000
2000
2003
2006
2009
2012
2015
2018
2021
Year of diagnosis

Figure S21. (C)

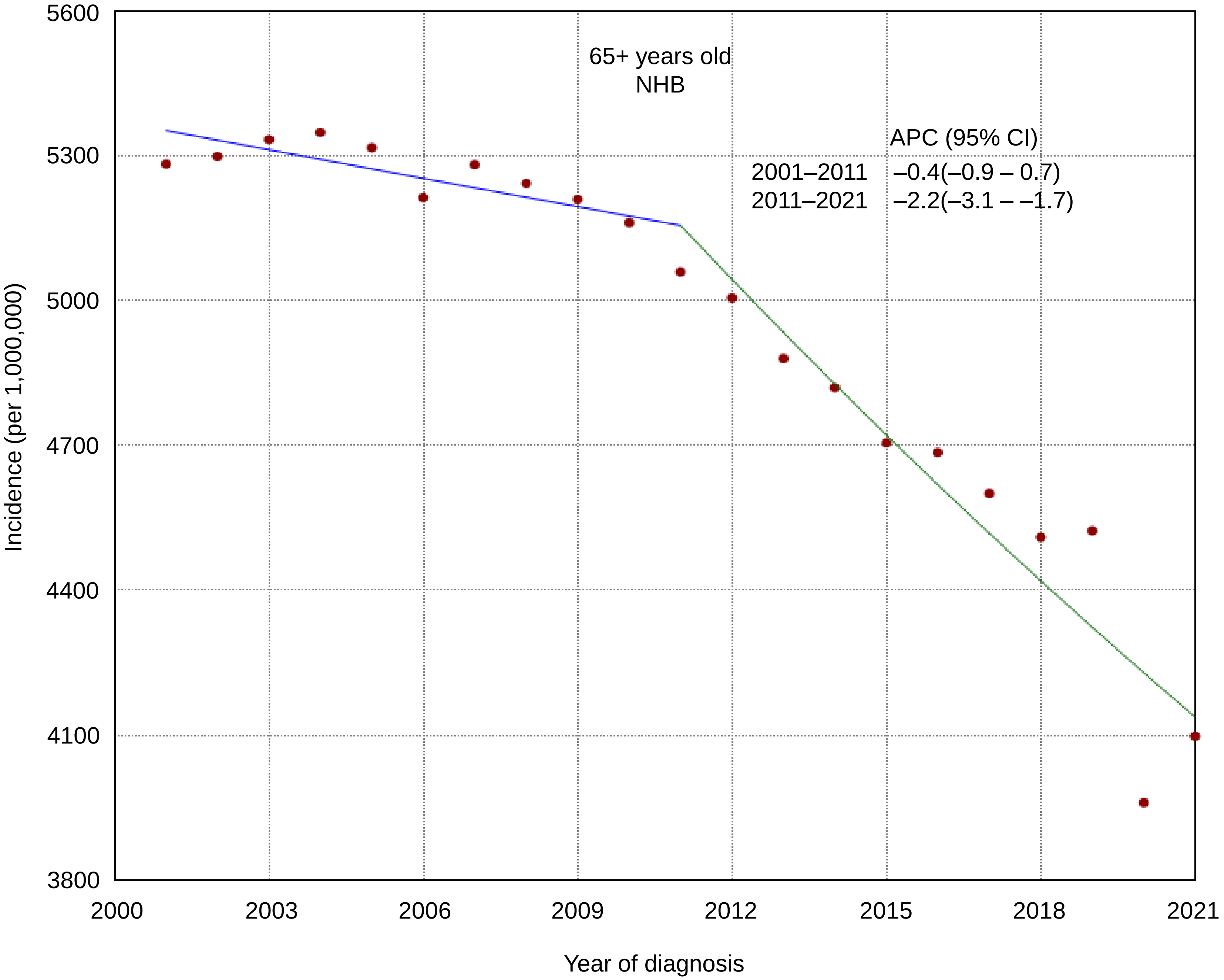

65+ years old
NHB
APC (95% CI)
2001–2011 –0.4(–0.9 – 0.7)
2011–2021 –2.2(–3.1 – –1.7)
Incidence (per 1,000,000)
5600
5300
5000
4700
4400
4100
3800
2000
2003
2006
2009
2012
2015
2018
2021
Year of diagnosis

Figure S21. (D)

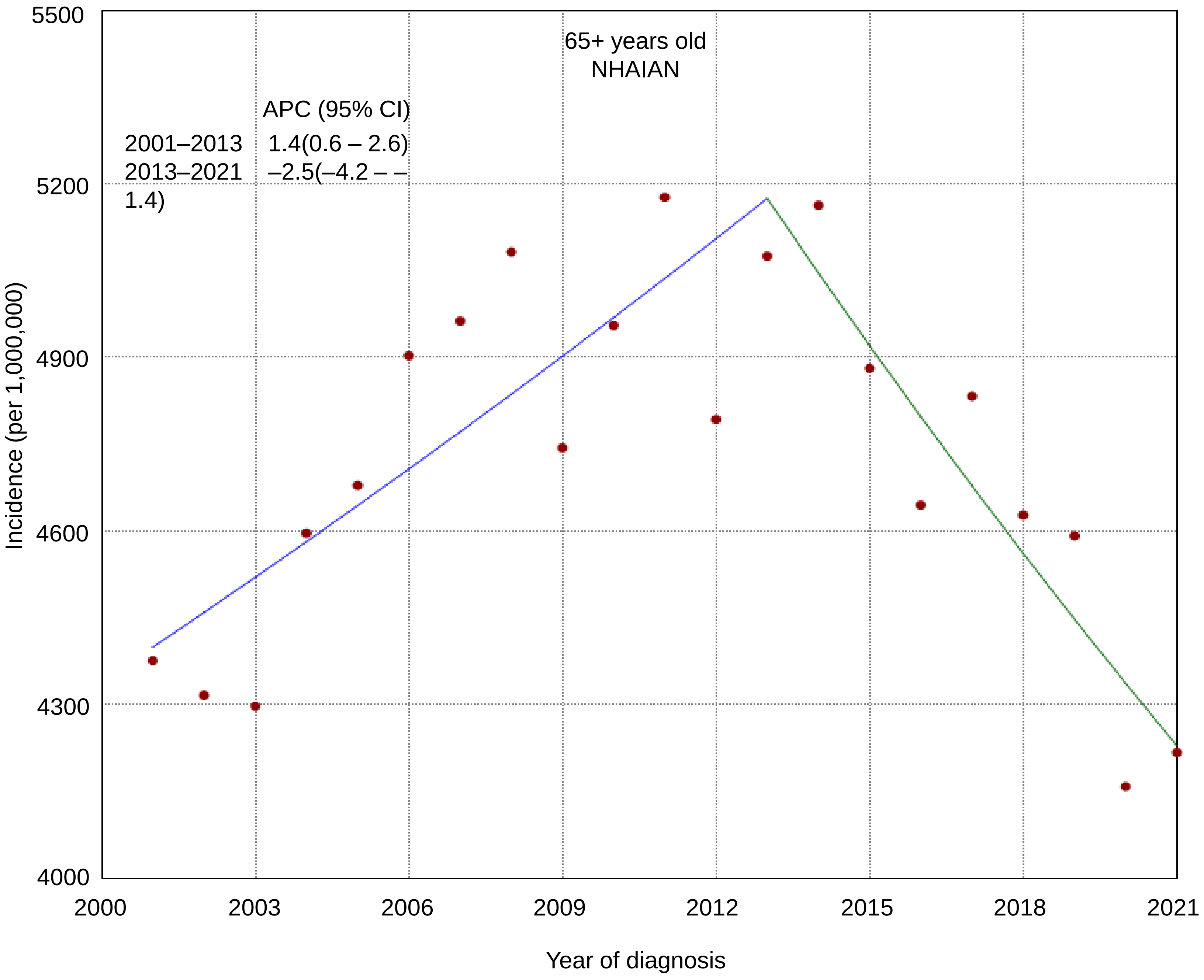

65+ years old
NHAIAN
APC (95% CI)
2001–2013 1.4(0.6 – 2.6)
2013–2021 –2.5(–4.2 – –1.4)
5500
5200
4900
4600
4300
4000
Incidence (per 1,000,000)
2000
2003
2006
2009
2012
2015
2018
2021
Year of diagnosis

Figure S21. (E)

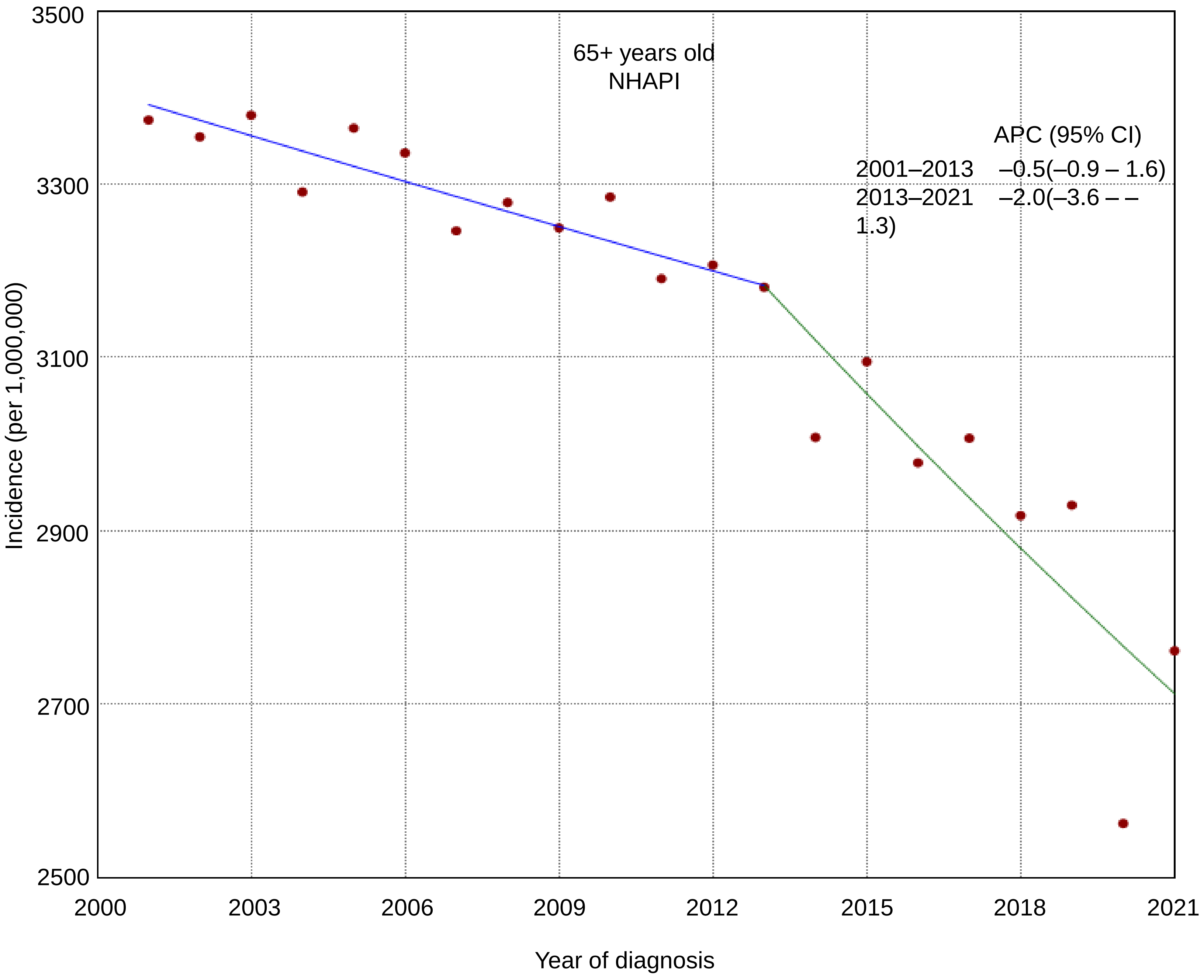

65+ years old
NHAPI
APC (95% CI)
2001–2013 –0.5(–0.9 – 1.6)
2013–2021 –2.0(–3.6 – –1.3)
3500
3300
3100
2900
2700
2500
Incidence (per 1,000,000)
2000
2003
2006
2009
2012
2015
2018
2021
Year of diagnosis

Figure S22. (A)

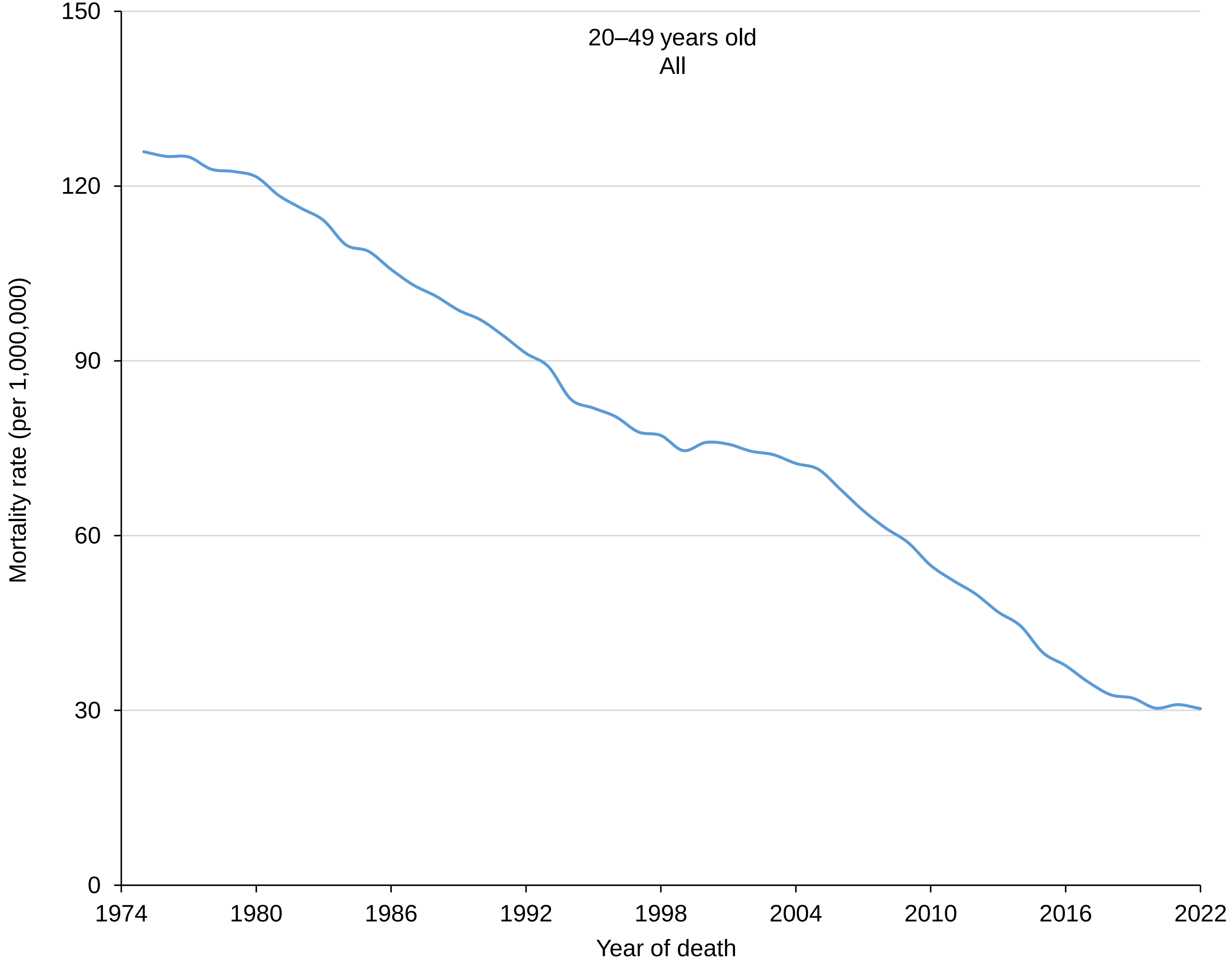

20–49 years old
All
150
120
90
60
30
0
Mortality rate (per 1,000,000)
1974
1980
1986
1992
1998
2004
2010
2016
2022
Year of death

Figure S22. (B)

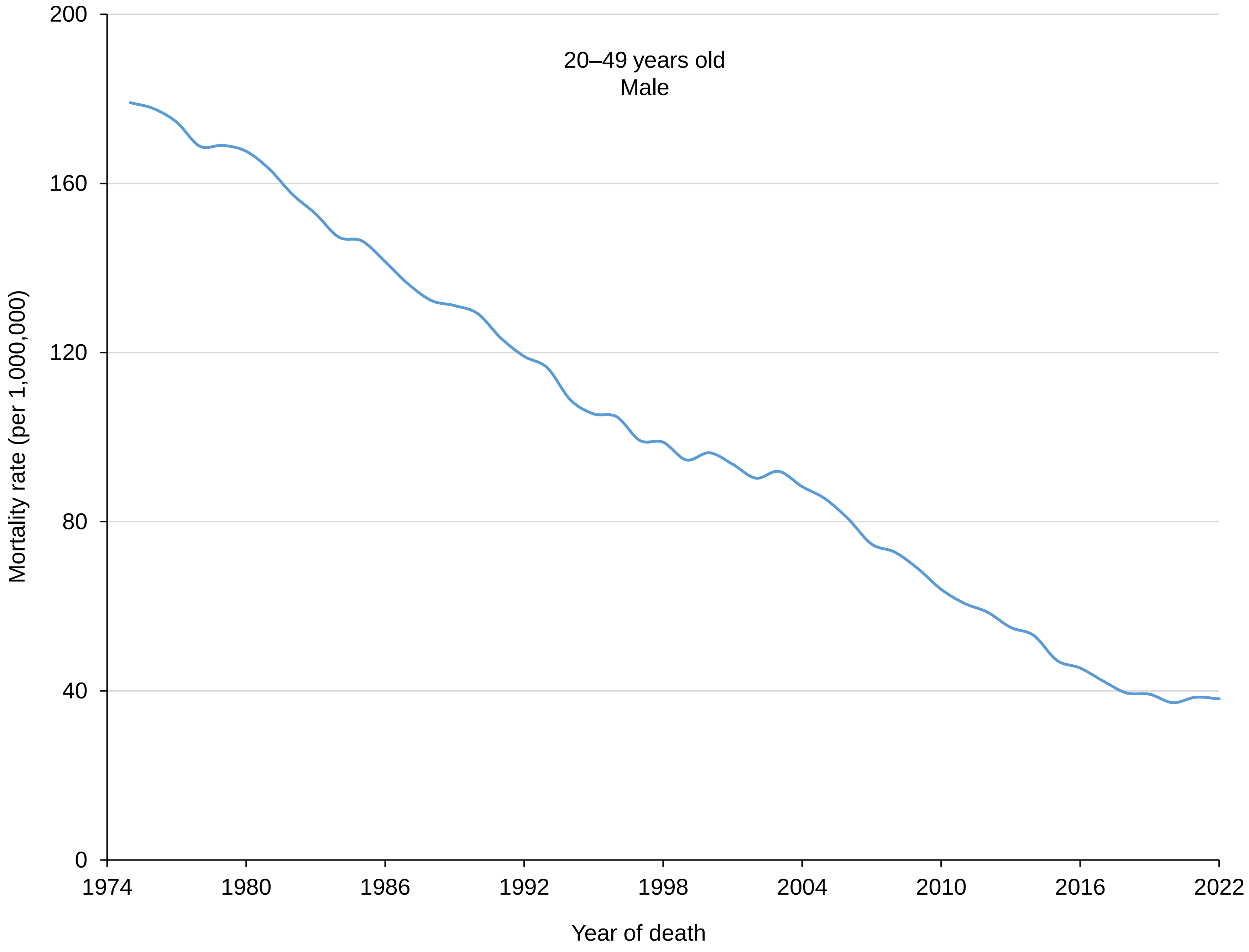
20–49 years old
Male
Mortality rate (per 1,000,000)
200
160
120
80
40
0
1974
1980
1986
1992
1998
2004
2010
2016
2022
Year of death

Figure S22. (C)

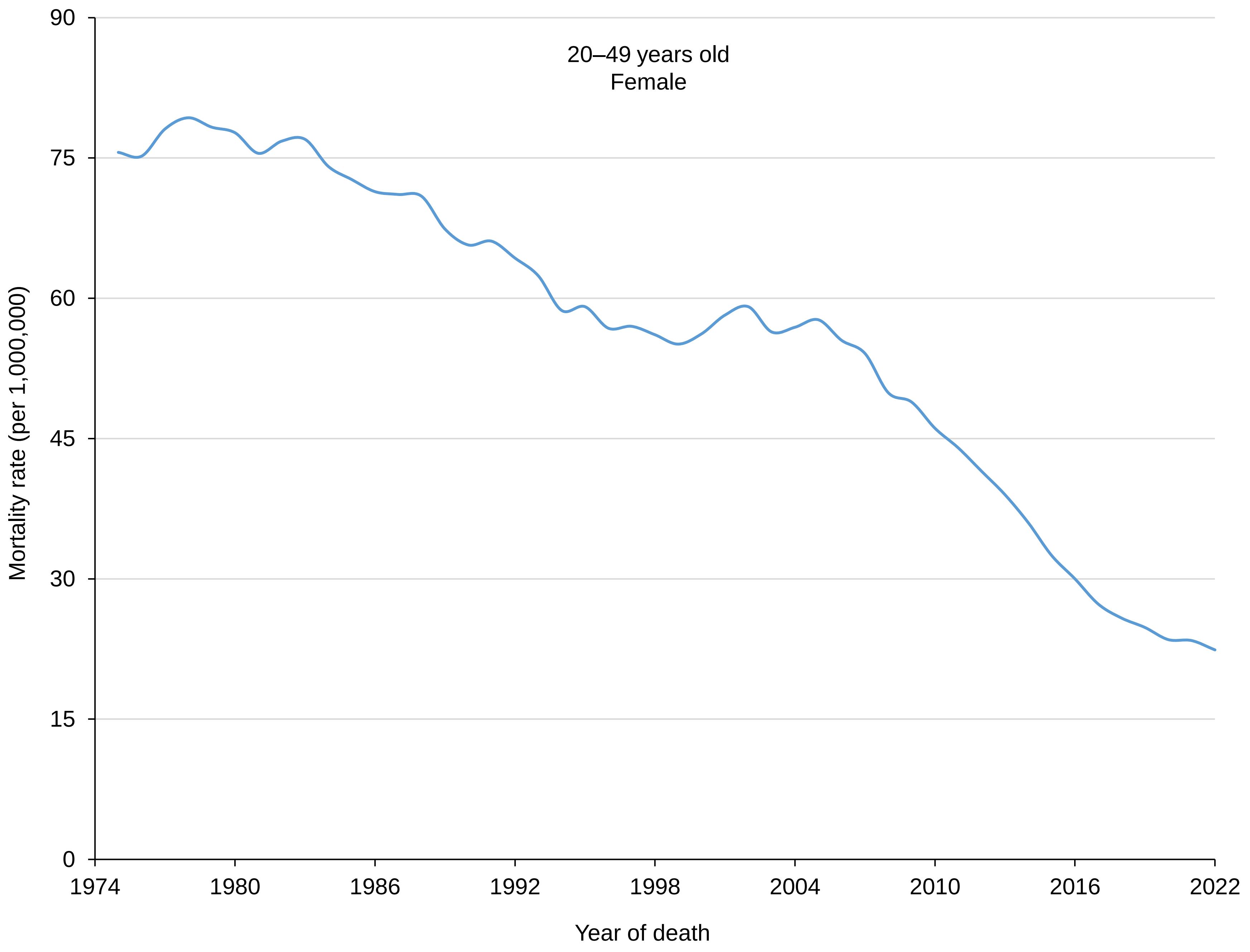
20–49 years old
Female
Mortality rate (per 1,000,000)
90
75
60
45
30
15
0
1974
1980
1986
1992
1998
2004
2010
2016
2022
Year of death

Figure S23. (A)

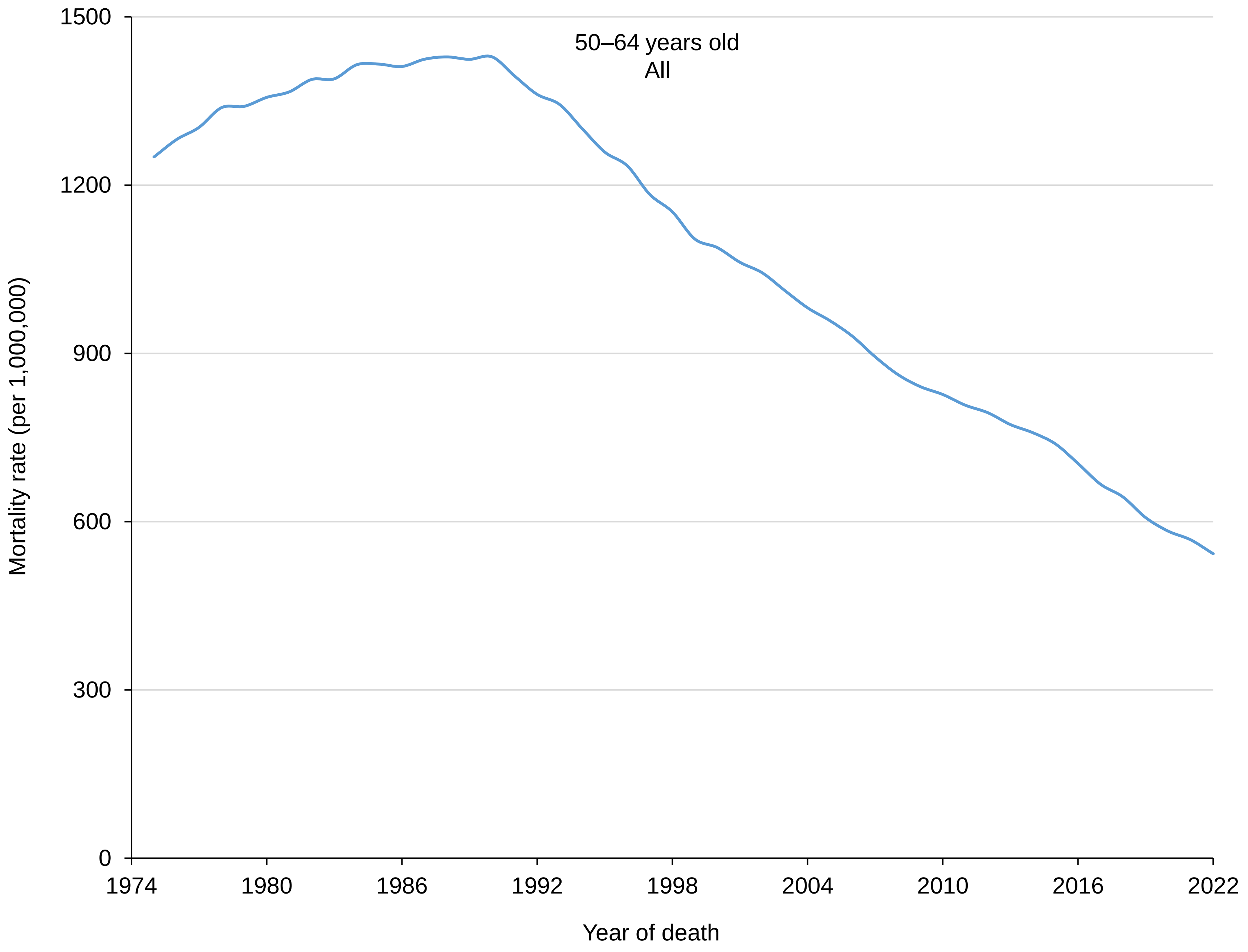

50–64 years old
All
1500
1200
900
600
300
0
Mortality rate (per 1,000,000)
1974
1980
1986
1992
1998
2004
2010
2016
2022
Year of death

Figure S23. (B)

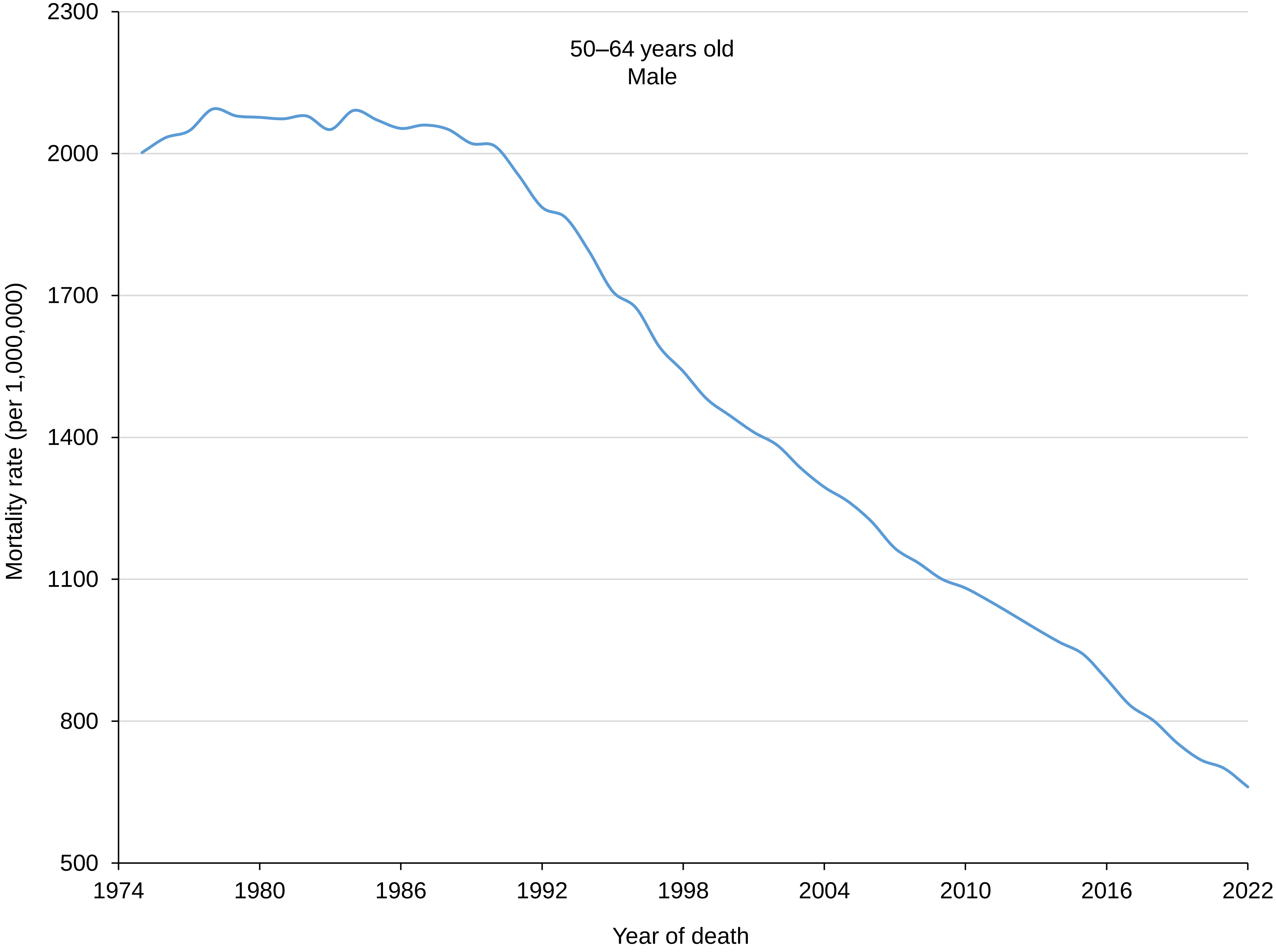
50–64 years old
Male
Mortality rate (per 1,000,000)
2300
2000
1700
1400
1100
800
500
1974
1980
1986
1992
1998
2004
2010
2016
2022
Year of death

Figure S23. (C)

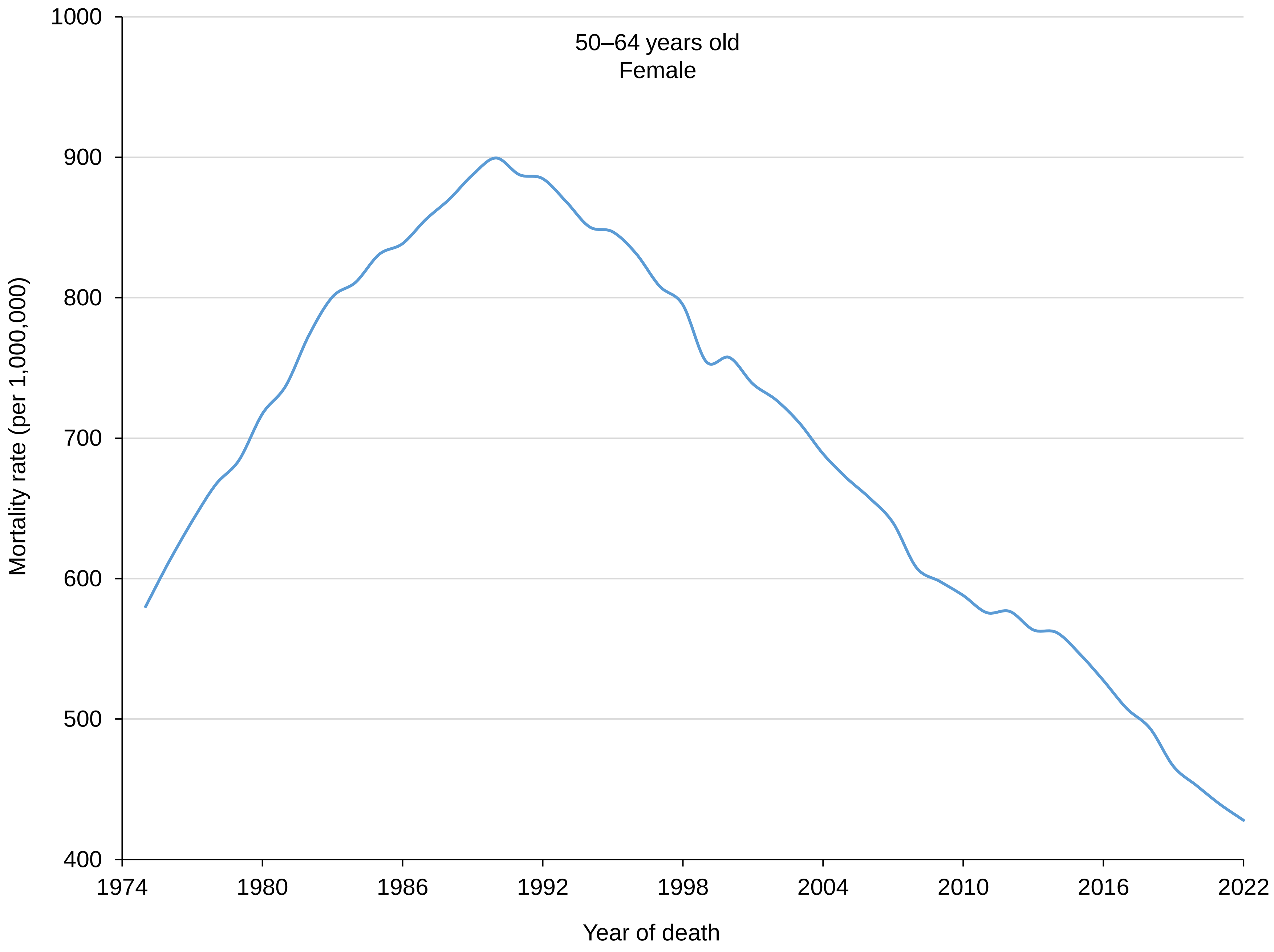
50–64 years old
Female
Mortality rate (per 1,000,000)
1000
900
800
700
600
500
400
1974
1980
1986
1992
1998
2004
2010
2016
2022
Year of death

Figure S24. (A)

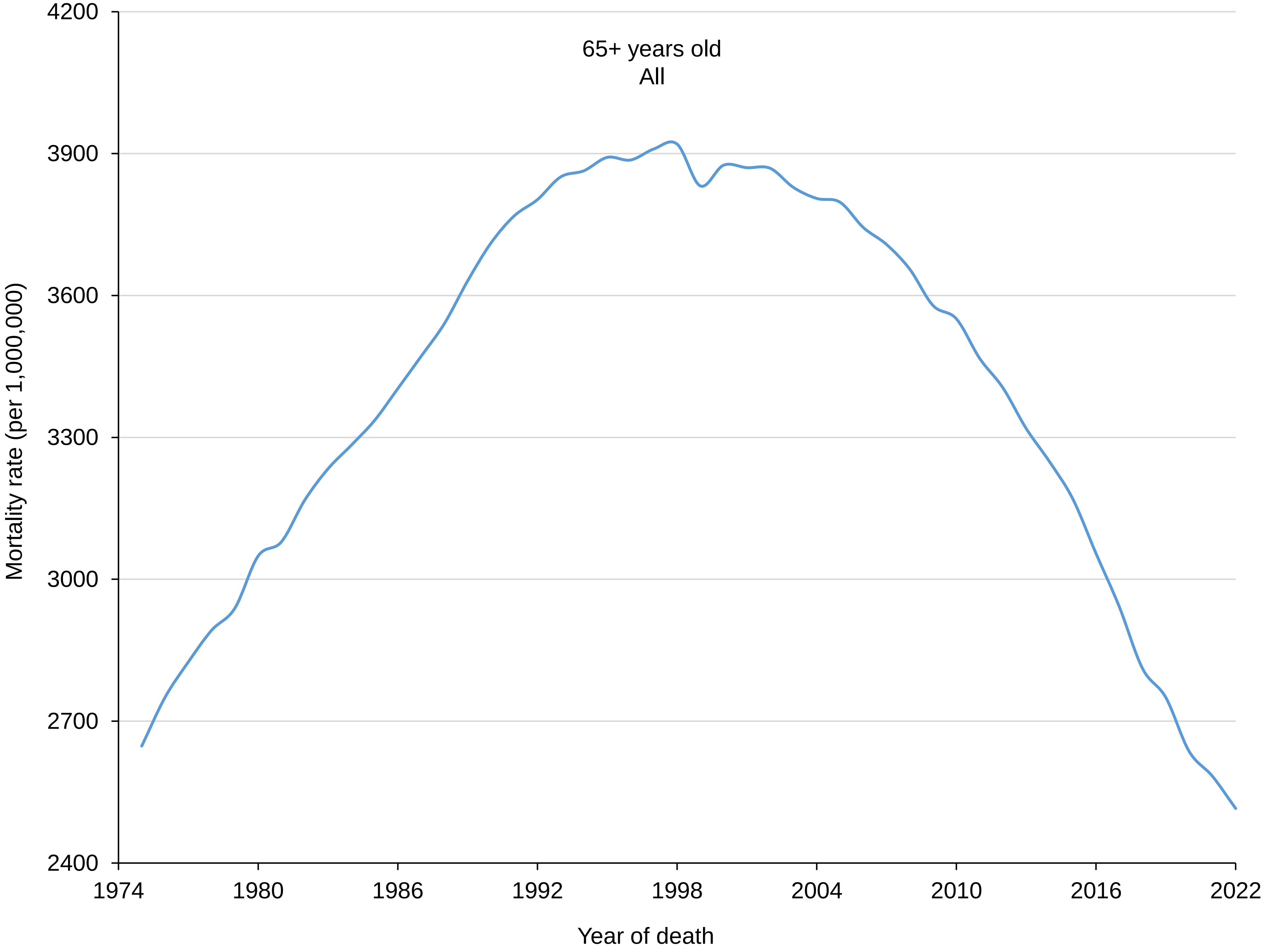
65+ years old
All
Mortality rate (per 1,000,000)
4200
3900
3600
3300
3000
2700
2400
1974
1980
1986
1992
1998
2004
2010
2016
2022
Year of death

Figure S24. (B)

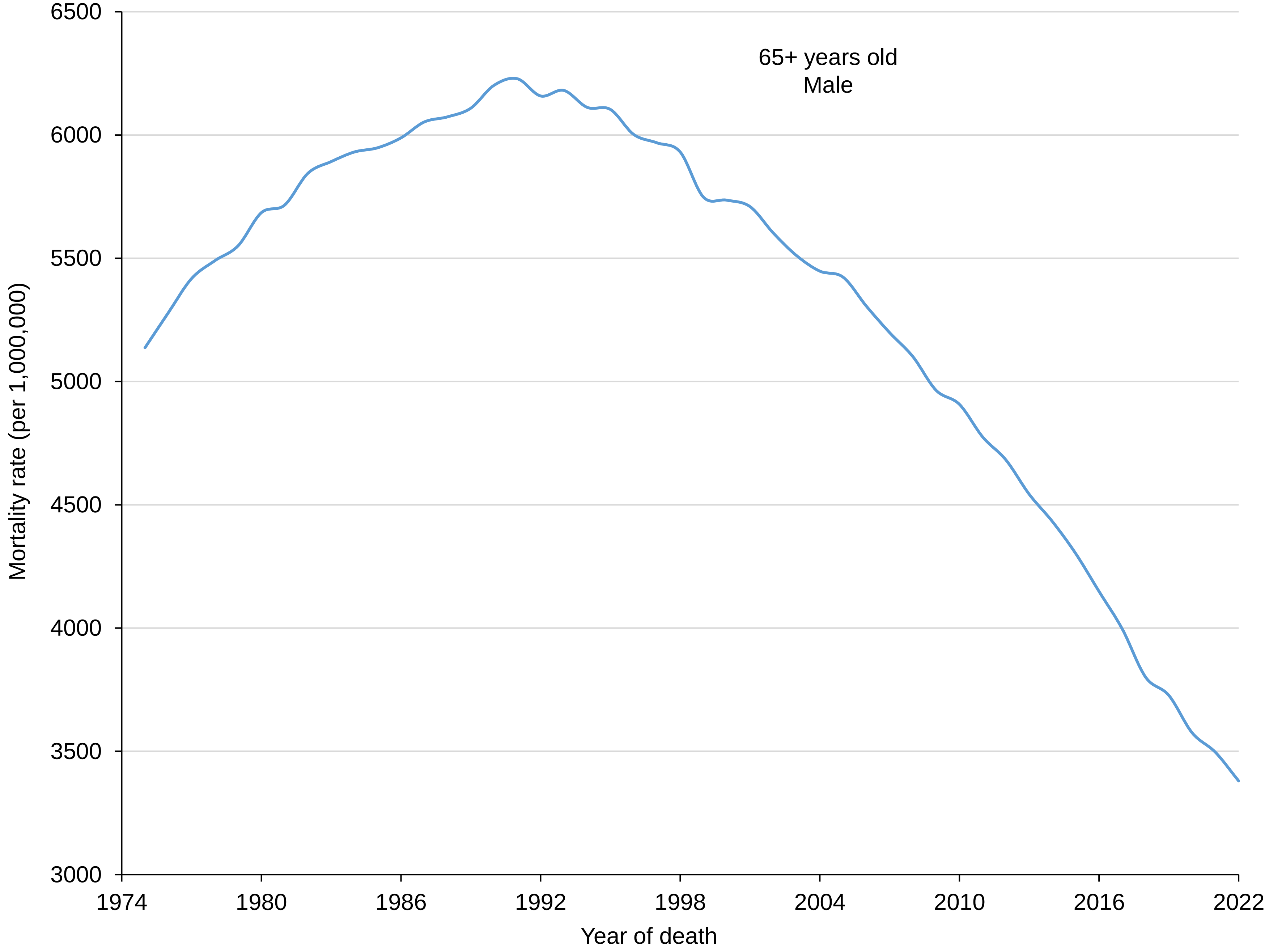
65+ years old
Male
Mortality rate (per 1,000,000)
6500
6000
5500
5000
4500
4000
3500
3000
1974
1980
1986
1992
1998
2004
2010
2016
2022
Year of death

Figure S24. (C)

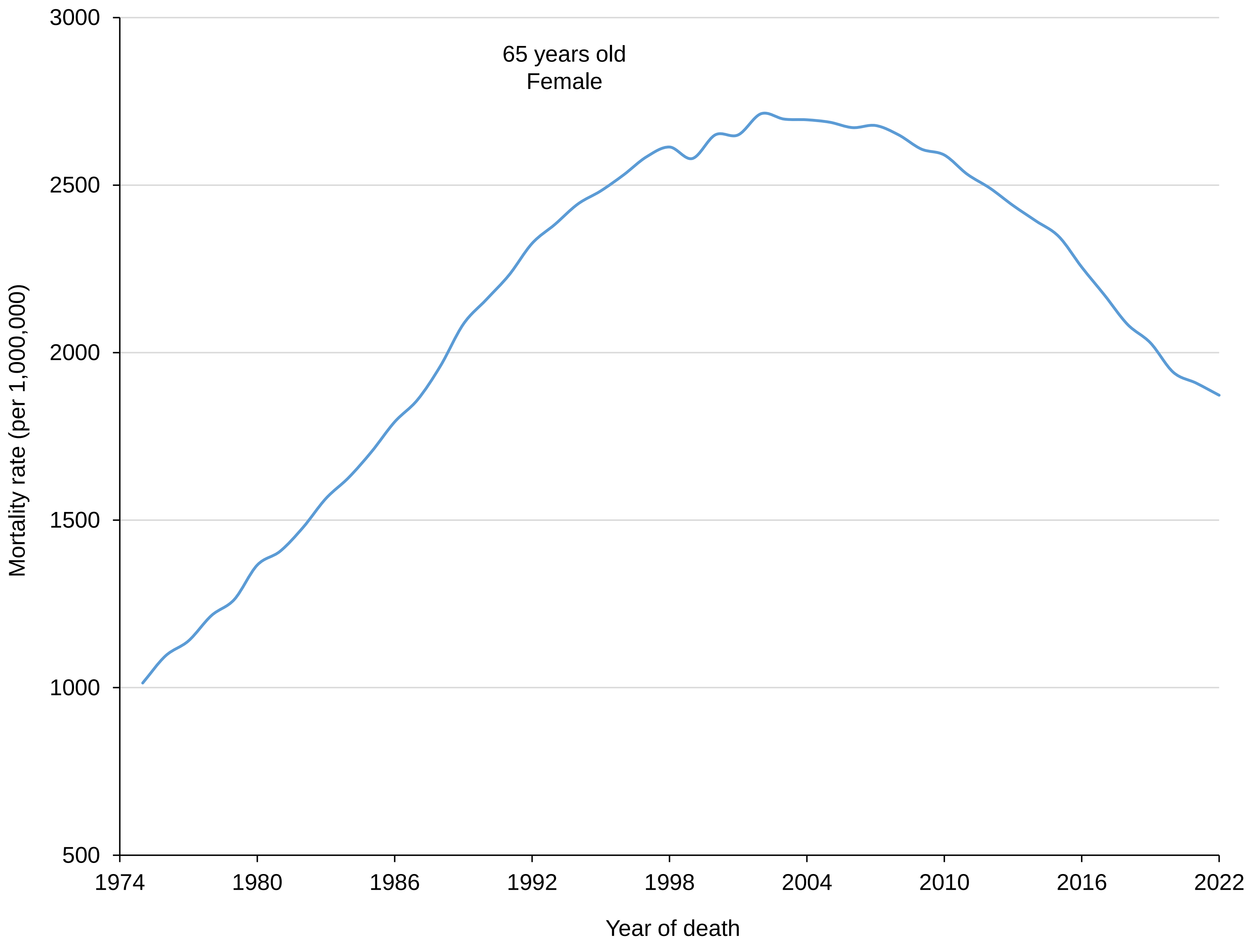
65 years old
Female
3000
2500
2000
1500
1000
500
Mortality rate (per 1,000,000)
1974
1980
1986
1992
1998
2004
2010
2016
2022
Year of death

Figure S25. (A)

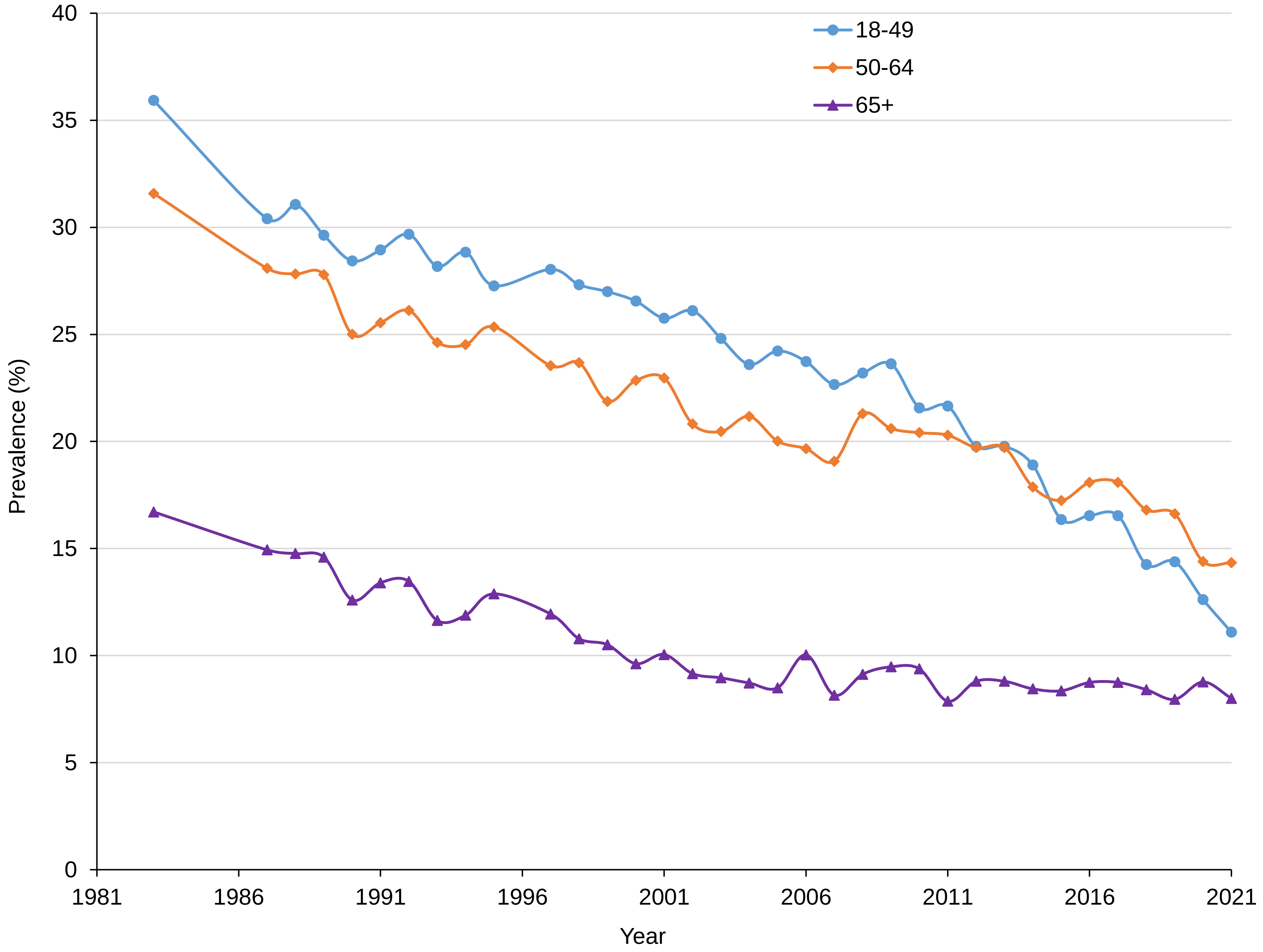

40
35
30
25
20
15
10
5
0
Prevalence (%)
1981
1986
1991
1996
2001
2006
2011
2016
2021
Year
18-49
50-64
65+

Figure S25. (B)

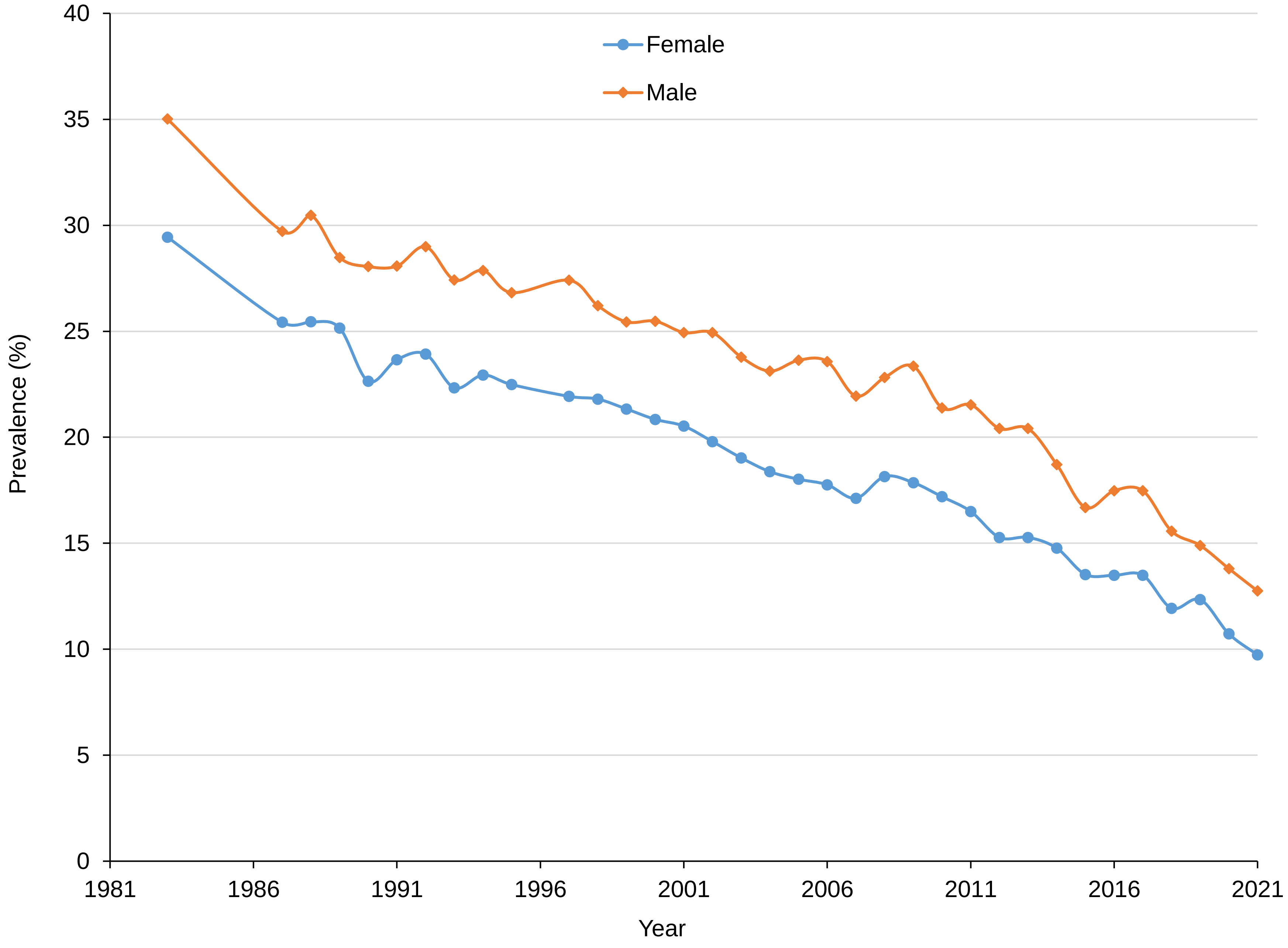

Female
Male
Prevalence (%)
Year
0
5
10
15
20
25
30
35
40
1981
1986
1991
1996
2001
2006
2011
2016
2021

Figure S25. (C)

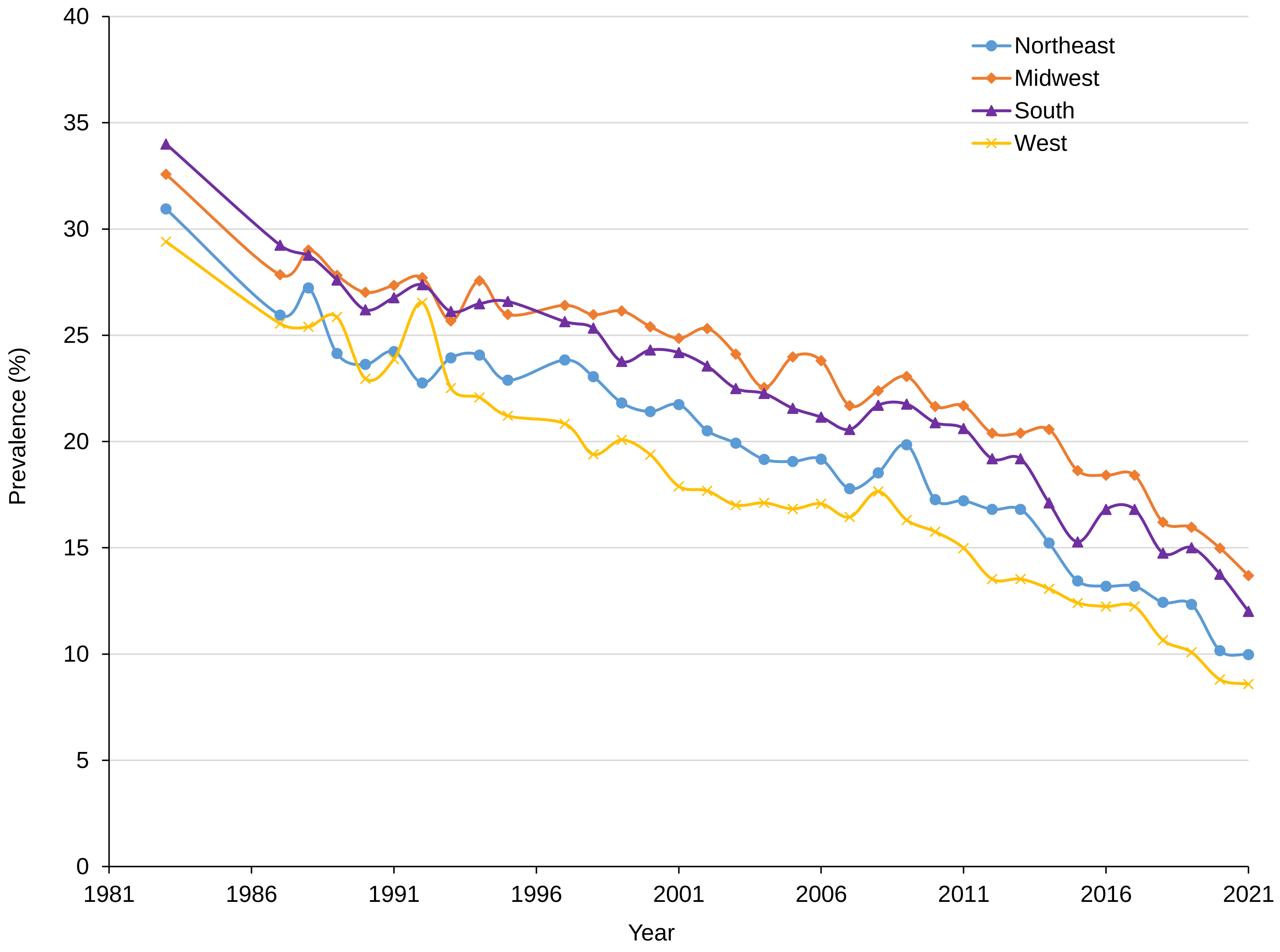

Prevalence (%)
40
35
30
25
20
15
10
5
0
1981
1986
1991
1996
2001
2006
2011
2016
2021
Year
Northeast
Midwest
South
West

Figure S25. (D)

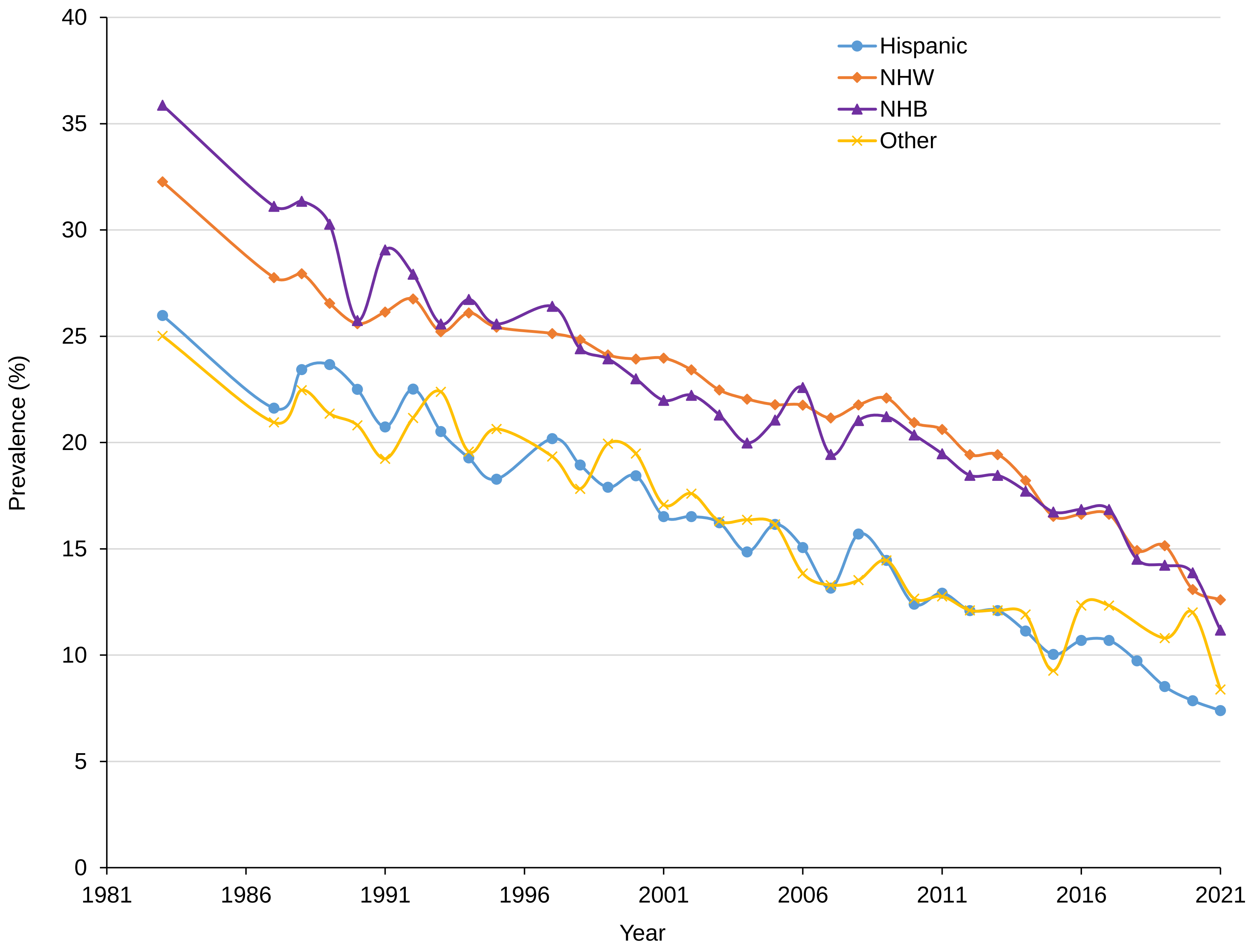
Hispanic
NHW
NHB
Other
Prevalence (%)
Year
0
5
10
15
20
25
30
35
40
1981
1986
1991
1996
2001
2006
2011
2016
2021

Figure S26. (A)

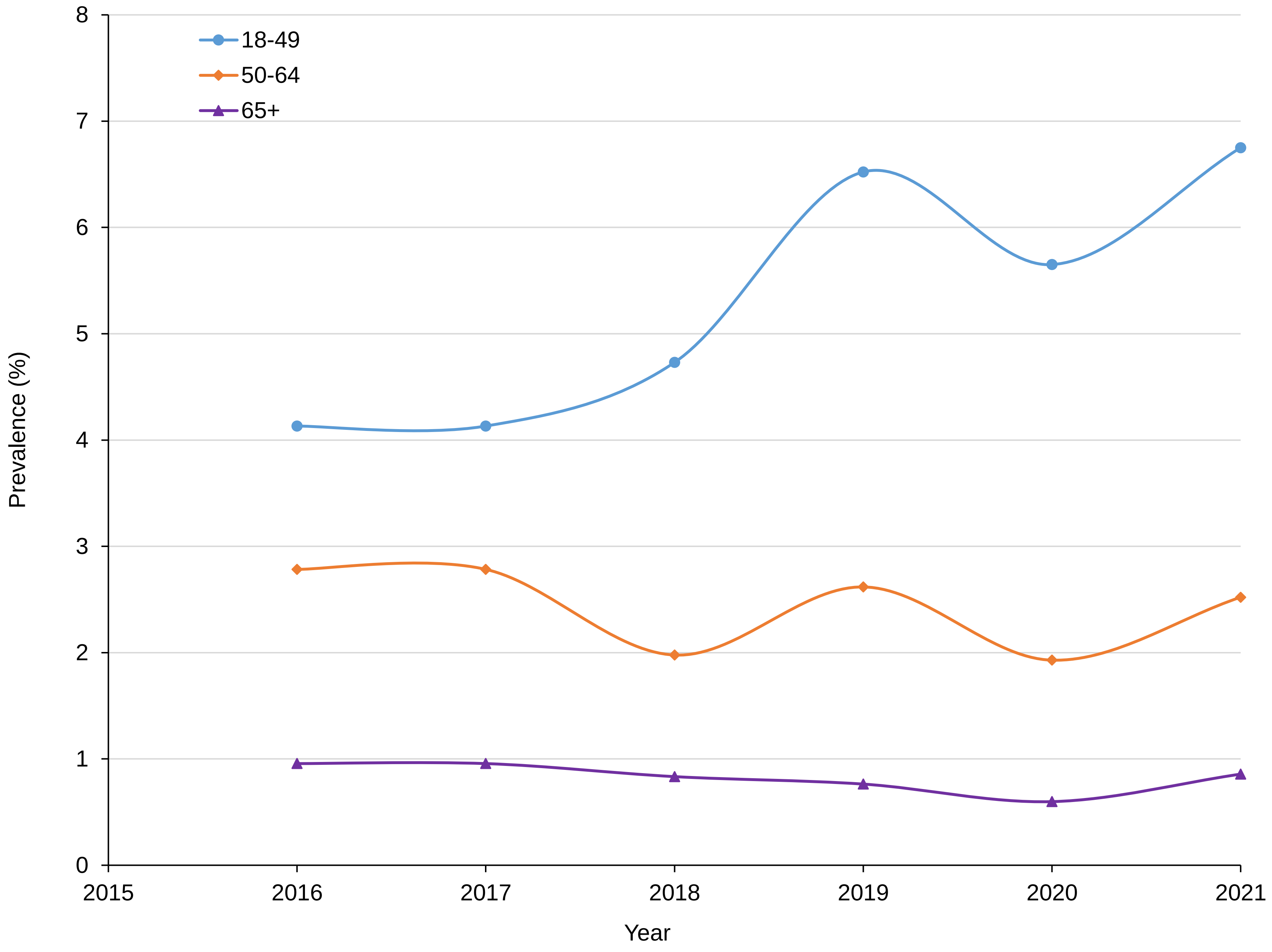
18-49
50-64
65+
Prevalence (%)
0
1
2
3
4
5
6
7
8
2015
2016
2017
2018
2019
2020
2021
Year

Figure S26. (B)

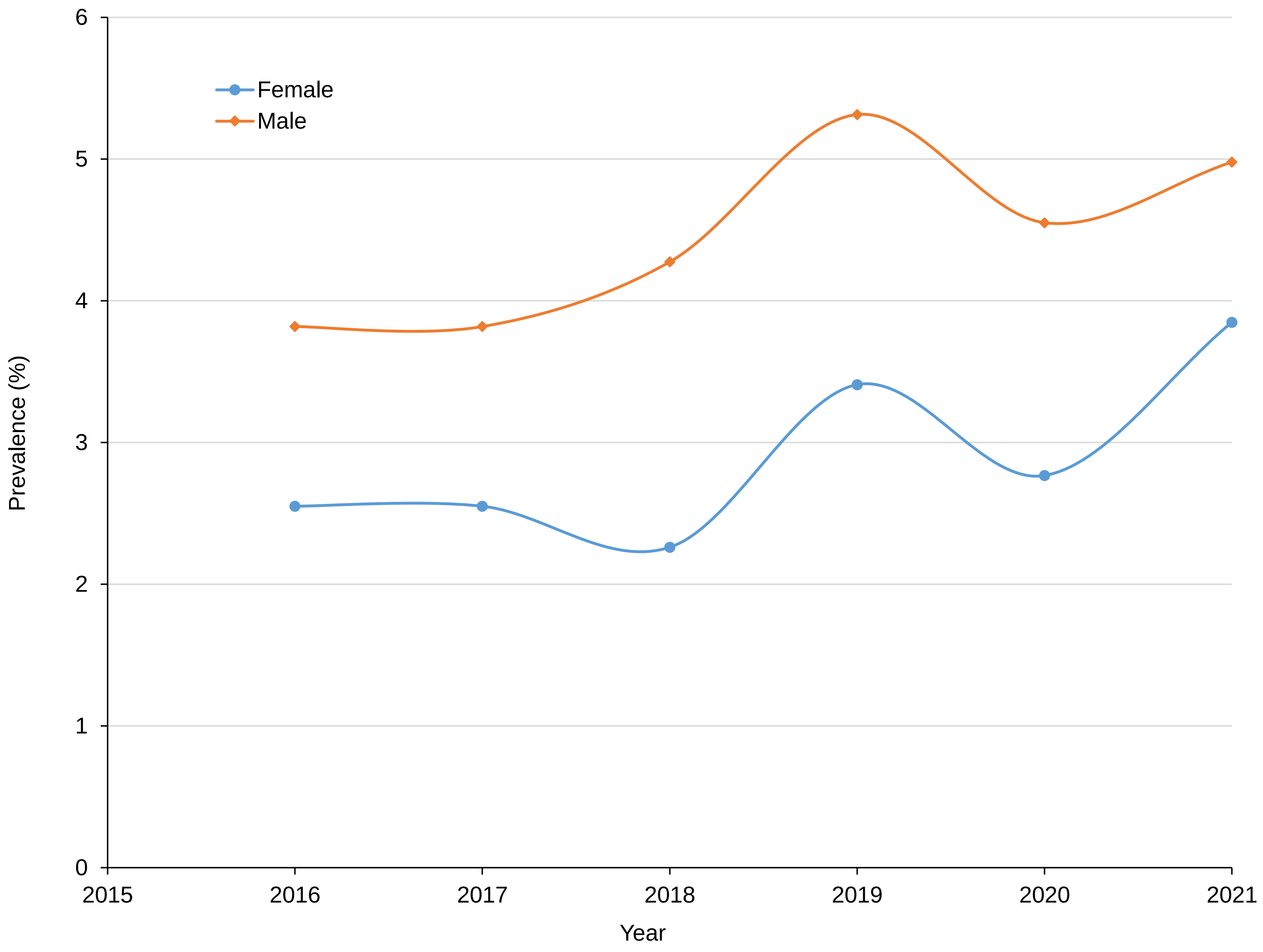

Female
Male
Prevalence (%)
0
1
2
3
4
5
6
2015
2016
2017
2018
2019
2020
2021
Year

Figure S26. (C)

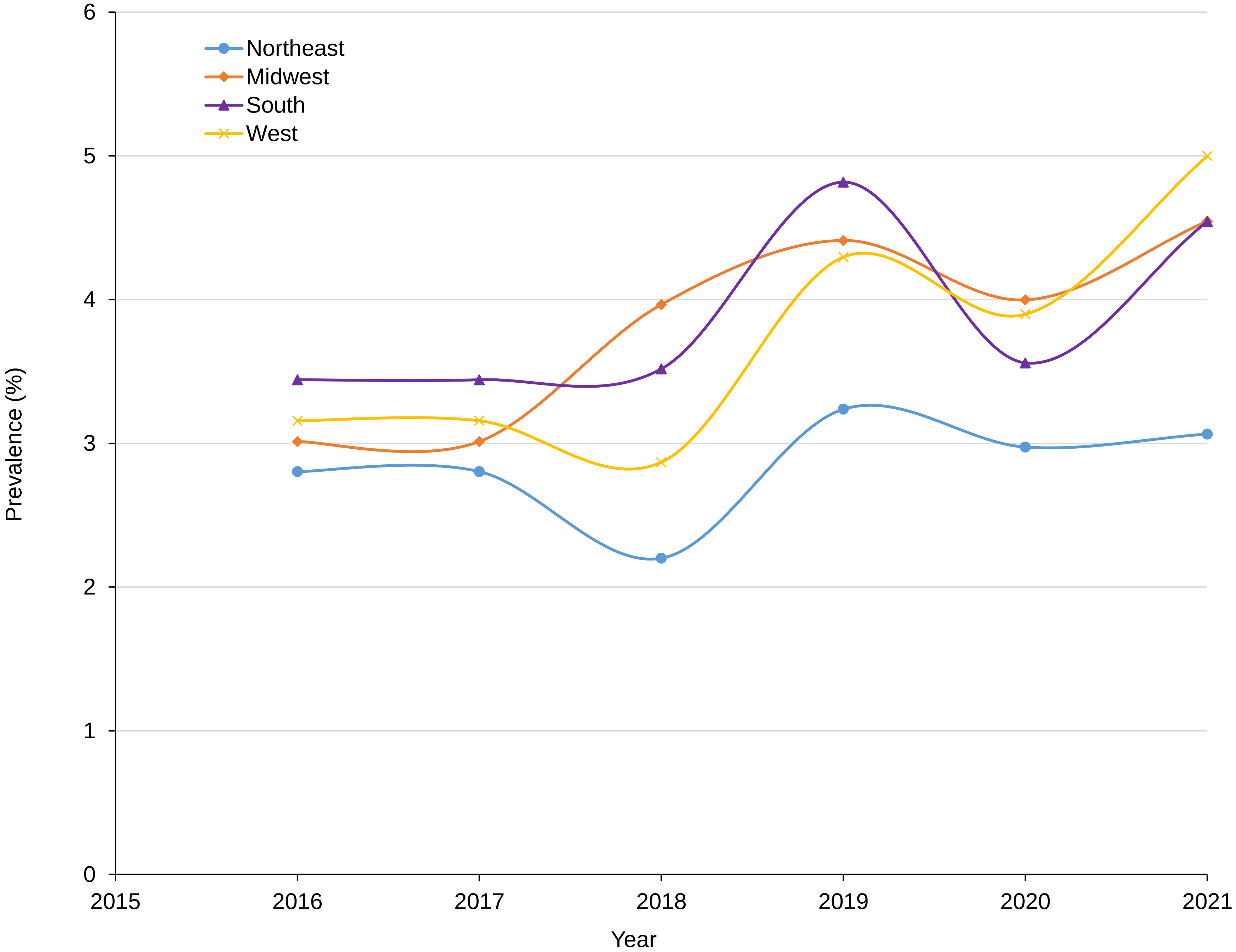

Northeast
Midwest
South
West
Prevalence (%)
Year
0
1
2
3
4
5
6
2015
2016
2017
2018
2019
2020
2021

Figure S26. (D)

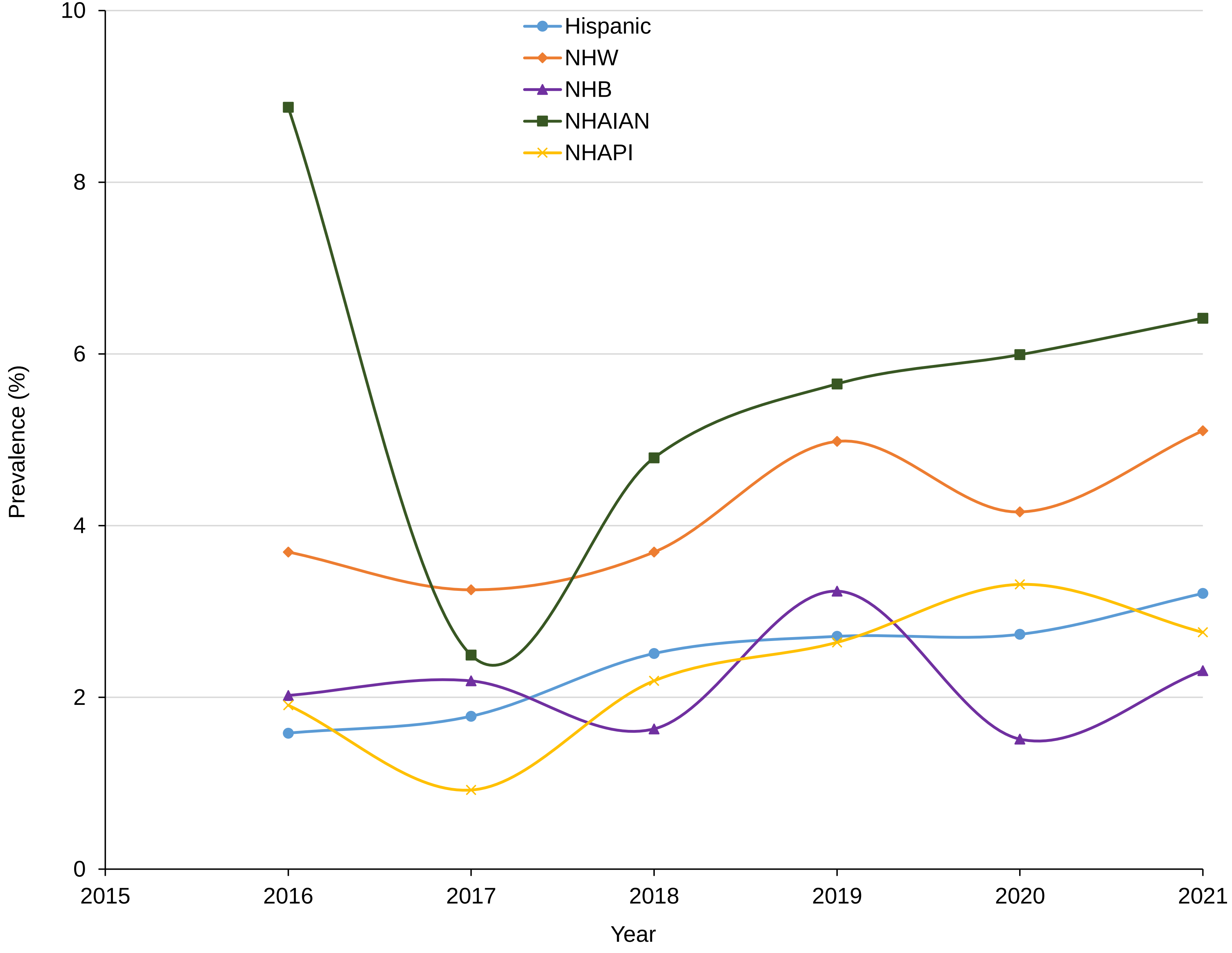
Hispanic
NHW
NHB
NHAIAN
NHAPI
10
8
6
4
2
0
Prevalence (%)
2015
2016
2017
2018
2019
2020
2021
Year

Figure S27. (A)

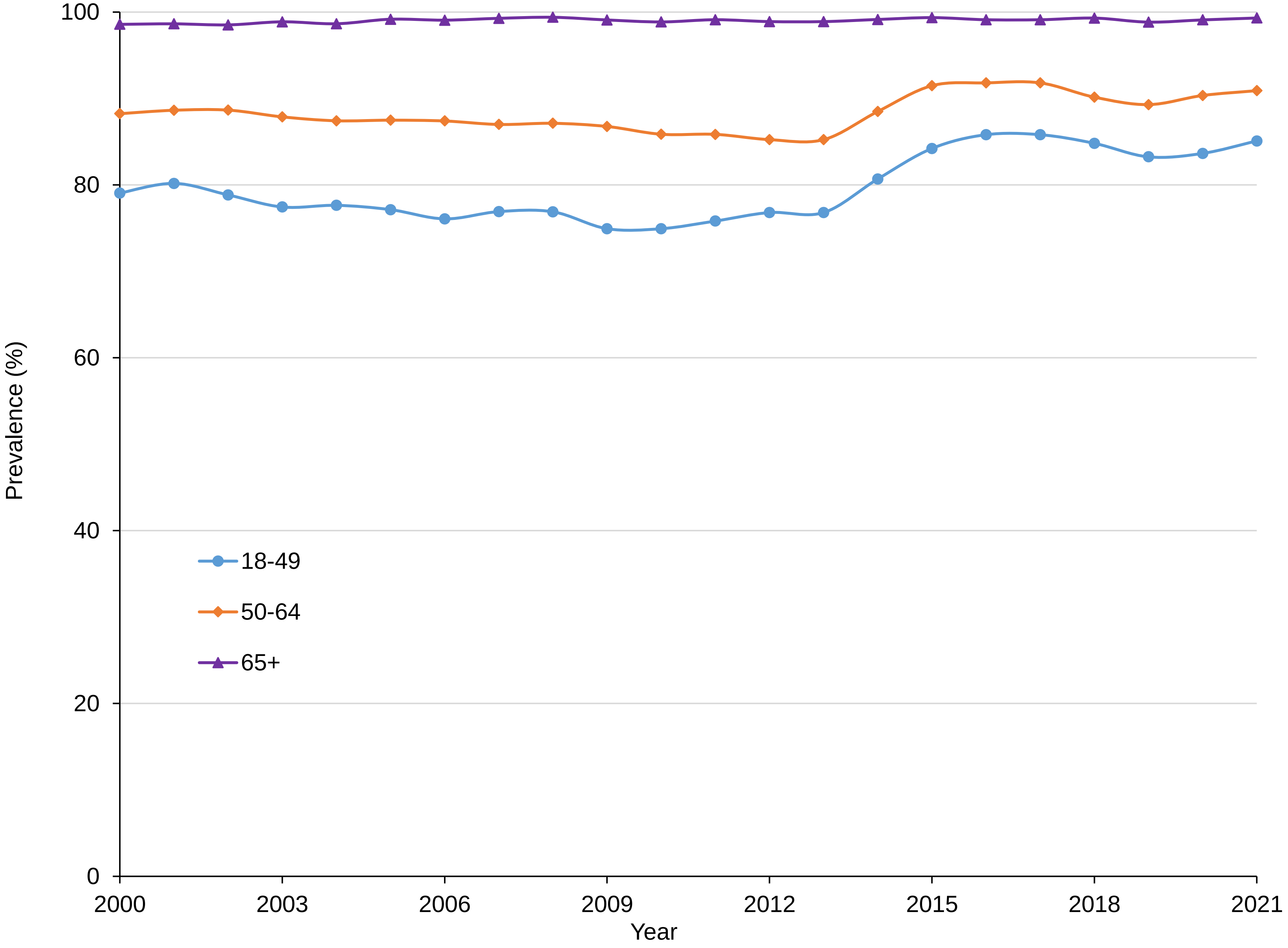

100
80
60
40
20
0
Prevalence (%)
2000
2003
2006
2009
2012
2015
2018
2021
Year
18-49
50-64
65+

Figure S27. (B)

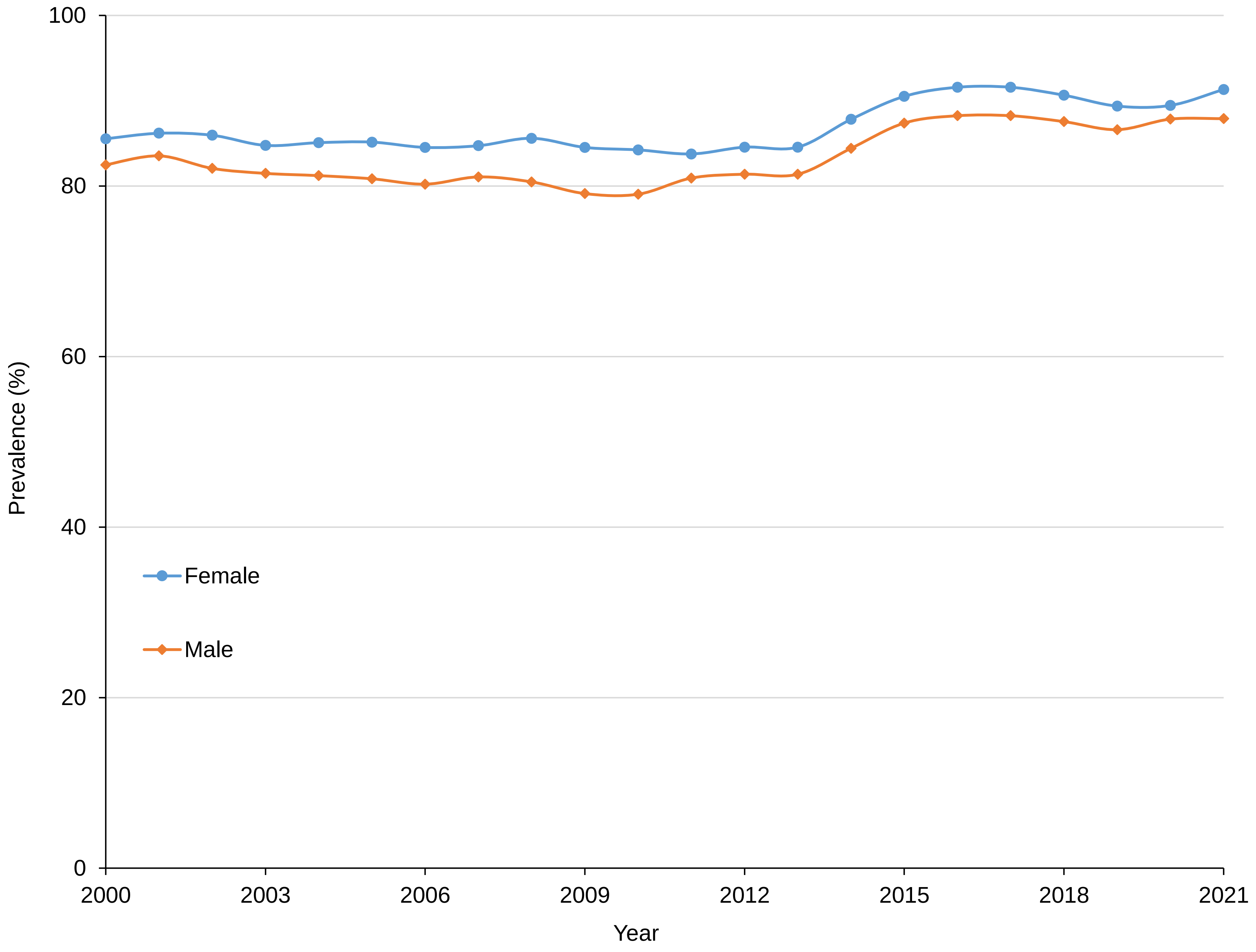

100
80
60
40
20
0
Prevalence (%)
Female
Male
2000
2003
2006
2009
2012
2015
2018
2021
Year

Figure S27. (C)

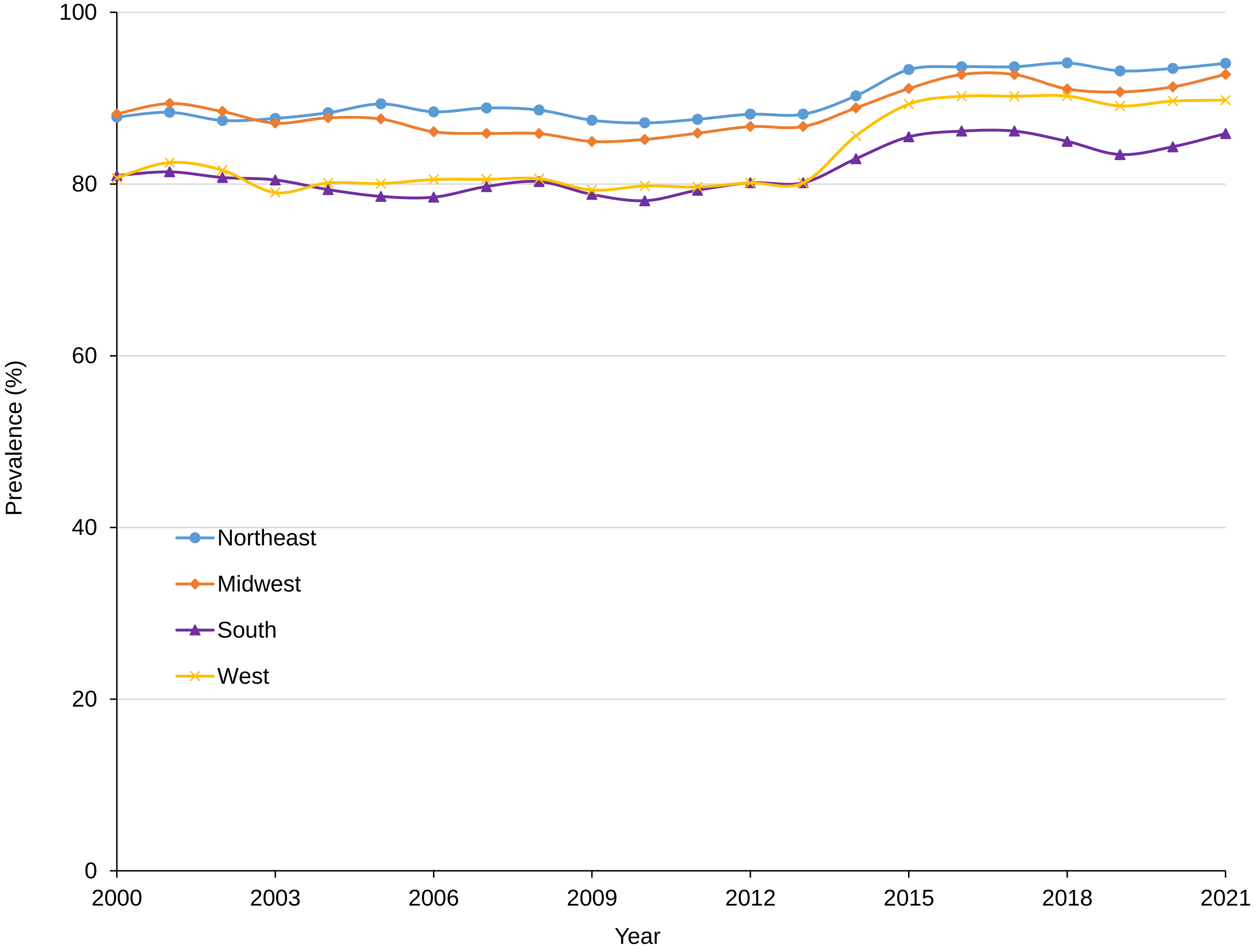

100
80
60
40
20
0
Prevalence (%)
2000
2003
2006
2009
2012
2015
2018
2021
Year
Northeast
Midwest
South
West

Figure S27. (D)

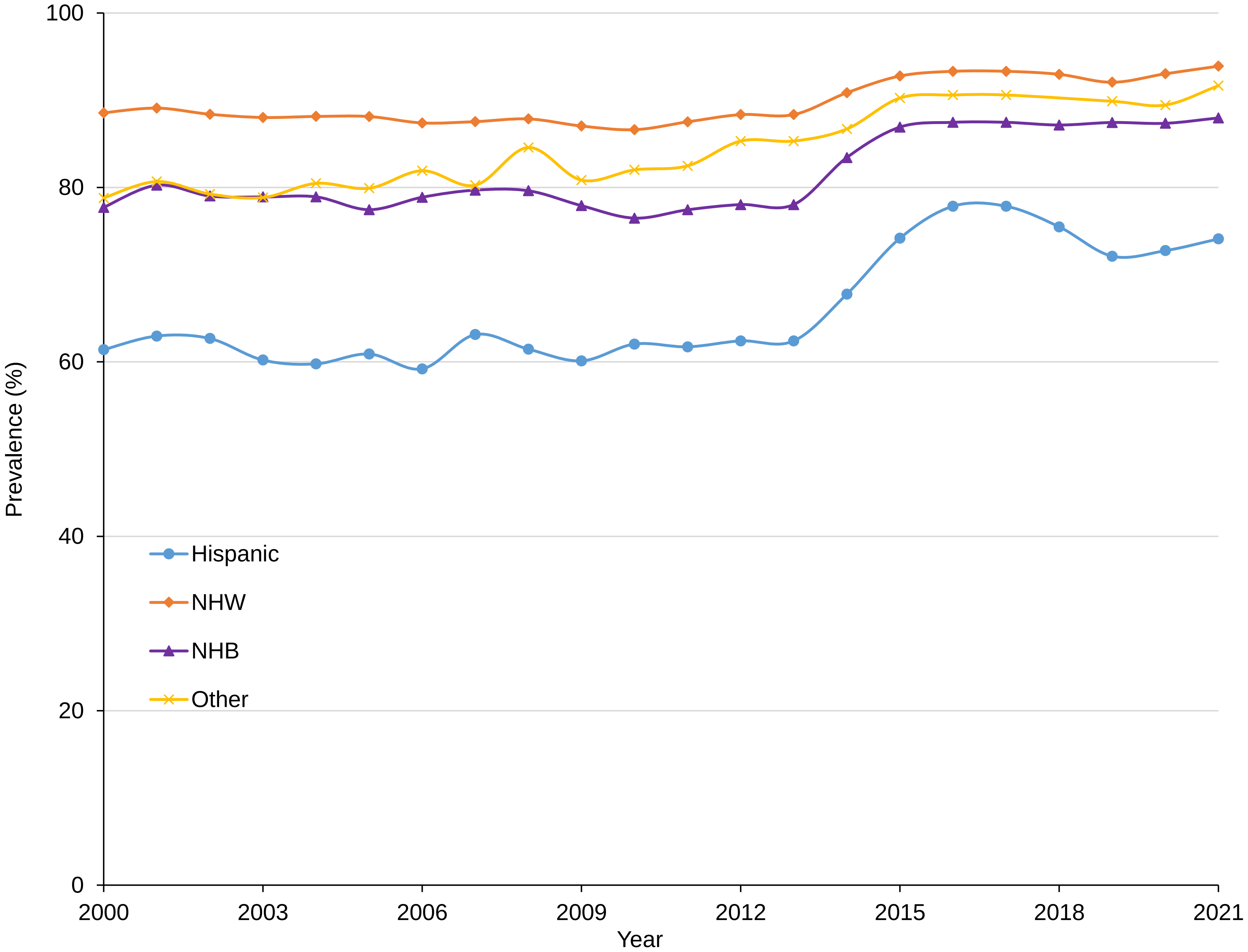

100
80
60
40
20
0
Prevalence (%)
2000
2003
2006
2009
2012
2015
2018
2021
Year
Hispanic
NHW
NHB
Other
